# Diet and sex inequities in ischemic heart disease mortality across Europe: findings from the global burden of disease study

**DOI:** 10.1093/cvr/cvaf176

**Published:** 2025-11-03

**Authors:** Raffaele Bugiardini, Tania Rahaman, Olivia Manfrini, Angela Maas, Maria Bergami, Lina Badimon, Guiomar Mendieta, Marija Vavlukis, Bela Merkely, Zorana Vasiljevic, Chris P Gale, Martha Gulati, Edina Cenko

**Affiliations:** Department of Medical and Surgical Sciences, University of Bologna, Via Giuseppe Massarenti 9, Bologna 40138, Italy; Department of Medical and Surgical Sciences, University of Bologna, Via Giuseppe Massarenti 9, Bologna 40138, Italy; Department of Medical and Surgical Sciences, University of Bologna, Via Giuseppe Massarenti 9, Bologna 40138, Italy; IRCCS Azienda Ospedaliero-Universitaria di Bologna Sant’Orsola Hospital, Bologna, Italy; Department of Women's Cardiac Health, Radboud University Medical Center, Nijmegen, Netherlands; Department of Medical and Surgical Sciences, University of Bologna, Via Giuseppe Massarenti 9, Bologna 40138, Italy; Cardiovascular Research Program ICCC, IR-IIB Sant Pau, University of Vic-UCC, CiberCV, Barcelona, Spain; Centro Nacional de Investigaciones Cardiovasculares Carlos III (CNIC), Instituto de Salud Carlos III, Madrid, Spain; University Clinic for Cardiology, Skopje 1000, Republic of North Macedonia; Faculty of Medicine, Ss. Cyril and Methodius University in Skopje, Skopje 1000, Republic of North Macedonia; Semmelweis University Heart and Vascular Center, Budapest, Hungary; Medical Faculty, University of Belgrade, Belgrade, Serbia; Leeds Institute of Cardiovascular and Metabolic Medicine, University of Leeds, Leeds, UK; Barbra Streisand Women's Heart Center Smidt Heart Institute, Cedars-Sinai Medical Center, Los Angeles, CA, USA; Department of Medical and Surgical Sciences, University of Bologna, Via Giuseppe Massarenti 9, Bologna 40138, Italy

**Keywords:** Sex differences, Ischemic heart disease, Deaths, Diet, Risk factors, Europe

## Abstract

**Aims:**

Sex differences in ischemic heart disease (IHD) mortality remain underexplored from a population-level case fatality perspective. This study evaluates sex-specific disparities in IHD mortality and risk-attributable causes across 27 European Union (EU) countries using Global Burden of Disease (GBD) 2021 data.

**Methods and results:**

We calculated age-standardized mortality rates (ASMRs), prevalence rates (ASPRs), and mortality-to-prevalence ratios (MPRs) as a proxy for population-level case fatality. To quantify mortality attributable to specific exposures among individuals with IHD, we derived a case fatality index (CFI) by normalizing risk-attributable ASMRs to ASPRs. *Z*-scores quantified the magnitude and statistical significance of sex differences in MPRs and CFIs (|*Z*| ≥ 1.96 = *P* < 0.05; |*Z*| ≥ 2.58 = *P* < 0.01). From 2011 to 2021, IHD ASMRs declined by 24.0% in men and 19.1% in women. In 2011, 12 countries showed significantly higher MPRs in women than men. By 2021, Austria (MPR 6.0% vs. 3.6%), Greece (9.4% vs. 5.3%), and Malta (9.3% vs. 4.2%) remained outliers, with *Z*-scores >2.58 (*P* < 0.01). CFIs showed that women in these countries faced 40 to 60% higher mortality burdens from hypertension, hyperglycemia, and poor dietary intake. Low intake of omega-3 fatty acids, fibers, vegetables, and nuts/seeds accounted for the largest dietary disparities.

**Conclusion:**

Despite declining IHD mortality rates, Austria, Greece, and Malta continue to exhibit significant sex disparities, with women experiencing disproportionately higher case fatality. These disparities are largely driven by modifiable cardiometabolic and dietary risks, underscoring the need for sex-specific, regionally tailored prevention strategies.

## Graphical Abstract

**Graphical Abstract:**
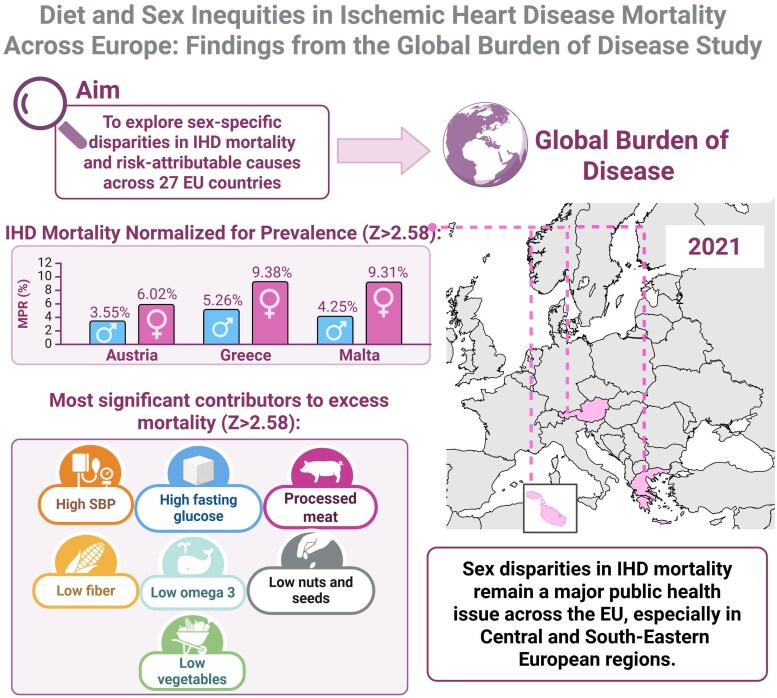

**Time of primary review: 10 days**

## 1. Introduction

Ischemic heart disease (IHD) remains one of the leading causes of mortality across European Union (EU) countries. According to the 2021 Global Burden of Disease (GBD) study, the age-standardized mortality rate (ASMR) for IHD in men is consistently higher than in women, a disparity primarily driven by the higher prevalence of IHD among men.^1^ However, despite having a lower prevalence of IHD, evidence suggests that once diagnosed, women may experience a higher relative mortality risk than men.^2^ The underlying mechanisms driving these sex-specific disparities remain unclear.

To date, comprehensive, population-based data on sex differences in IHD mortality and associated risk factors across EU countries are lacking. Existing research on cardiovascular sex disparities in Europe is largely limited to high-income Western European nations, with scant data available from middle-income countries, where IHD prevalence can be two to three times higher. This imbalance hinders a comprehensive understanding of how sex disparities in IHD outcomes may vary by socioeconomic context and geography.

The GBD study may circumvent this issue as it provides comprehensive assessments of global health and is therefore well-suited to achieve internationally comparable estimates of mortality and associated risk factors. Using GBD data, we examined sex-specific trends in IHD mortality across all 27 EU member states from 2011 to 2021, with attention to economic stratification, regional variation, and modifiable risk factors. Our goal was to identify patterns and drivers of sex disparities in IHD outcomes across the EU and inform strategies aimed at reducing inequities in cardiovascular health among European populations.

## 2. Methods

### 2.1 Data sources

This study is compliant with the Guidelines for Accurate and Transparent Health Estimates Reporting (GATHER) statement. We utilized mortality and population data from individual EU countries provided by the GBD study at two benchmark years: 2011 and 2021.^3^ These data were further complemented by economic classifications from the European Commission, Directorate-General for Research and Innovation, using data from the Transitions Performance Index (TPI) 2021.^4^ (see Supplementary material online, *Appendix Table S1*). The score identifies 5 ranking areas: (ⅰ) transition leader (75–100); (ⅱ) strong transition (65–74); (ⅲ) good transition (55–64); (ⅳ) moderate transition (45–54); and (ⅴ) weak transition (0–44). Since the data analysed are publicly available, with no personally identifiable information, ethical approval was not required for this study.

### 2.2 Risk factor estimation

We selected IHD risk factors based on established causal evidence, including high systolic blood pressure, elevated low-density lipoprotein (LDL) cholesterol, high fasting plasma glucose, tobacco use, and high body mass index (BMI). Definitions followed the GBD 2021 framework.^5^ Smoking exposure comprised current/former tobacco use plus secondhand smoke, all compared with a theoretical minimum risk level of zero. Theoretical minimum risk exposure levels (TMRELs) for other key risk factors were as follows: blood pressure, 110–115 mmHg; fasting plasma glucose, 4.8–5.4 mmol/L; LDL cholesterol, 0.7–1.3 mmol/L; and BMI, 20–25 kg/m². Additional modifiable exposures included low physical activity (TMRELs < 3000–4500 MET-min/week) and ambient/household air pollution (ozone, PM_2.5_, and solid fuel use).

### 2.3 Dietary risk-attributable mortality

Dietary risk-attributable mortality in the GBD is not based on direct dietary intake surveys from each country. Rather, it is modeled using a combination of nationally representative dietary data, food balance sheets, household consumption surveys, and covariates such as education, urbanization, and income. These inputs are synthesized through Bayesian meta-regression (DisMod-MR 2.1) to estimate population-level exposures, which are then linked to disease outcomes using risk-outcome relationships derived from large-scale cohort studies.

### 2.4 Health effects of IHD dietary risks

Based on GBD criteria, we evaluated 12 dietary components linked to IHD, including insufficient intake of whole grains, fruits, fiber, legumes, vegetables, nuts/seeds, seafood omega-3 s, and polyunsaturated fatty acids (PUFAs), and excessive intake of red meat, processed meat, sodium, and sugar-sweetened beverages. Trans-fat data were unavailable for 2021 and thus excluded. Where GBD estimates showed implausible negative values within 95% uncertainty intervals (UI), specifically for low omega-6 PUFA and legume intake, or high intake of sugar-sweetened beverages and red meat, only point estimates were reported. These were flagged as unstable and excluded from statistical interpretation. Optimal intake levels for each factor are provided in Supplementary material online, *Appendix Table S2*.

### 2.5 Statistical analysis

We analysed sex-specific IHD outcomes across 27 EU countries from 2011 to 2021 using data from the GBD 2021 study. Primary outcomes included ASMRs, age-standardized prevalence rates (ASPRs), and mortality-to-prevalence ratios (MPRs), all expressed per 100 000 population and standardized to the GBD reference population. MPRs, calculated as ASMR divided by ASPR, served as a proxy for population-level case fatality, enabling cross-country and sex-based comparisons. UI for MPRs were estimated using the extreme value method.

We also assessed GBD-derived ASMRs attributable to specific risk factors, modeled using TMRELs within the GBD and Burden of Proof frameworks. Full methodology is publicly available (https://github.com/ihmeuw-msca/burden-of-proof).

Sex differences were assessed using two-sample *Z*-tests for independent proportions. This approach was applied both to MPRs and to risk factor–attributable ASMRs, which were normalized for the prevalence. This derived measure, termed the case fatality index (CFI), approximates the mortality burden associated with specific risk factors among individuals living with IHD, providing a prevalence-adjusted perspective on sex-specific risk impact. Descriptive comparisons were also made using women-to-men rate ratios, where values >1.0 indicate relatively greater female burden.

We used Pearson’s correlation to assess trends (*P* < 0.05 considered significant), with Shapiro-Wilk tests verifying normality. Analyses were conducted in Stata v17.0 using publicly available GBD and World Bank data. Further details on statistical methods and adjustments are provided in the Supplementary material online, Appendix.

## 3. Results

Between 2011 and 2021, ASMRs for IHD declined significantly across EU countries in both men and women, with mean absolute reductions of 24.0% and 19.1%, respectively (*Table 1*). Men consistently exhibited higher ASPR and ASMR than women throughout the study period. In 2021, the ASMR for men in the EU was 1.8 times higher than that of women, and the ASPR for men was 2.3 times higher than the corresponding rate for women.

**Table 1:** Sex-stratified distribution of age-standardized prevalence and mortality rates for IHD across European countries (years 2011 vs. 2021)

Country	Age-standardized prevalence rate of IHD per 100 000 inhabitants				Age-standardized mortality rate of IHD per 100 000 inhabitants				Mortality to Prevalence Ratio %				Women to Men Ratio	
	2011		2021		2011		2021		2011		2021		2011	2021
	Men	Women	Men	Women	Men	Women	Men	Women	Men	Women	Men	Women		
Austria	2602.26	820.80	2600.33	818.64	125.84	71.60	92.35	49.30	4.84	8.72	3.55	6.02	1.80	1.70
Lower bound 95% CI	2338.41	739.69	2306.18	718.16	115.48	59.73	82.72	40.15	4.05	6.58	2.83	4.36		
Upper bound 95% CI	2849.00	907.78	2921.21	921.02	132.32	77.79	97.78	54.18	5.66	10.52	4.24	7.54		
Belgium	2181.26	751.25	2190.02	757.31	82.00	39.90	54.54	25.11	3.76	5.31	2.49	3.32	1.41	1.33
Lower bound 95% CI	1974.34	672.60	1916.93	659.03	75.20	33.05	49.10	20.48	3.12	3.94	1.96	2.36		
Upper bound 95% CI	2413.74	838.13	2499.83	866.79	86.48	43.90	58.26	28.04	4.38	6.53	3.04	4.25		
Bulgaria	4585.53	2574.92	4368.54	2520.10	299.10	183.91	267.20	155.85	6.52	7.14	6.12	6.18	1.10	1.01
Lower bound 95% CI	4136.13	2336.51	3864.79	2246.71	282.21	169.25	235.29	137.23	5.61	6.03	4.85	4.84		
Upper bound 95% CI	5034.79	2807.07	4853.16	2834.89	315.94	196.26	302.23	174.31	7.64	8.40	7.82	7.76		
Croatia	3685.50	1721.96	3827.09	1931.88	199.79	134.90	155.67	108.92	5.42	7.83	4.07	5.64	1.45	1.39
Lower bound 95% CI	3245.41	1515.44	3320.79	1696.65	188.01	120.29	138.90	94.00	4.47	6.10	3.19	4.25		
Upper bound 95% CI	4209.93	1972.31	4355.72	2212.51	208.81	144.91	169.81	120.60	6.43	9.56	5.11	7.11		
Cyprus	1554.59	494.34	1822.57	633.49	170.68	86.07	116.12	62.12	10.98	17.41	6.37	9.81	1.59	1.54
Lower bound 95% CI	1354.36	418.88	1563.62	538.85	152.81	72.12	101.63	51.72	8.58	12.23	4.84	6.96		
Upper bound 95% CI	1781.97	589.82	2101.86	742.68	187.80	96.80	131.13	71.34	13.87	23.11	8.39	13.24		
Czech Republic	4158.48	2311.59	4218.04	2634.26	224.47	138.29	164.68	97.17	5.40	5.98	3.90	3.69	1.11	0.94
Lower bound 95% CI	3758.55	2089.19	3730.13	2337.34	211.32	123.23	145.55	82.49	4.61	4.80	3.09	2.82		
Upper bound 95% CI	4584.39	2570.01	4713.62	2925.87	234.11	146.54	181.42	107.67	6.23	7.01	4.86	4.61		
Denmark	1554.40	583.22	1650.00	568.42	82.17	43.23	56.22	26.47	5.29	7.41	3.41	4.66	1.40	1.37
Lower bound 95% CI	1383.95	517.41	1438.23	494.91	74.63	36.78	50.40	21.75	4.22	5.58	2.69	3.31		
Upper bound 95% CI	1769.68	658.64	1873.94	656.14	86.77	47.16	60.04	29.12	6.27	9.11	4.17	5.88		
Estonia	4719.33	3021.20	5007.33	3208.47	262.22	131.92	139.32	65.18	5.56	4.37	2.78	2.03	0.79	0.73
Lower bound 95% CI	4345.16	2760.11	4452.66	2897.53	246.35	114.66	123.34	54.42	4.78	3.46	2.21	1.52		
Upper bound 95%CI	5149.91	3315.18	5577.95	3585.15	274.45	142.73	155.28	73.61	6.32	5.17	3.49	2.54		
Finland	2083.61	827.37	2420.89	909.29	155.75	75.99	109.27	52.05	7.47	9.18	4.51	5.72	1.23	1.27
Lower bound 95%CI	1869.26	737.64	2118.43	788.66	140.32	61.61	96.36	41.45	6.03	6.59	3.52	4.01		
Upper bound 95% CI	2326.06	935.32	2739.77	1034.80	163.46	83.59	116.79	57.84	8.74	11.33	5.51	7.33		
France	2604.53	824.35	2516.06	814.04	61.76	26.95	44.63	18.85	2.37	3.27	1.77	2.32	1.38	1.31
Lower bound 95% CI	2379.68	747.27	2244.40	723.91	56.98	22.45	39.92	15.09	1.99	2.45	1.40	1.63		
Upper bound 95% CI	2867.51	917.96	2843.48	924.51	64.99	29.54	47.85	20.87	2.73	3.95	2.13	2.88		
Germany	3047.30	965.42	2584.64	882.52	114.15	61.64	90.09	43.12	3.75	6.38	3.49	4.89	1.70	1.40
Lower bound 95% CI	2791.91	883.94	2292.02	777.51	105.63	51.51	81.33	34.99	3.18	4.83	2.80	3.46		
Upper bound 95% CI	3323.41	1066.53	2905.49	1010.92	119.53	67.47	95.37	47.93	4.28	7.63	4.16	6.16		
Greece	2006.09	584.63	1819.01	578.03	130.64	92.35	95.63	54.20	6.51	15.80	5.26	9.38	2.43	1.78
Lower bound 95% CI	1803.20	511.19	1604.99	493.92	121.65	79.72	89.26	45.72	5.42	11.88	4.32	6.68		
Upper bound 95% CI	2244.09	671.22	2065.31	684.98	136.43	99.00	100.54	58.51	7.57	19.37	6.26	11.85		
Hungary	4905.74	2778.89	4573.18	2684.32	258.07	159.46	199.87	122.78	5.26	5.74	4.37	4.57	1.09	1.05
Lower bound 95% CI	4480.24	2530.10	4094.42	2418.59	245.91	145.01	178.24	107.28	4.56	4.74	3.51	3.56		
Upper bound 95% CI	5395.03	3056.71	5074.73	3014.34	267.50	168.70	218.12	134.97	5.97	6.67	5.33	5.58		
Ireland	2188.89	735.96	2178.57	750.05	117.00	60.53	71.50	36.28	5.35	8.22	3.28	4.84	1.54	1.47
Lower bound 95% CI	1984.19	652.85	1898.02	656.22	108.13	50.81	63.37	28.78	4.42	6.13	2.57	3.35		
Upper bound 95% CI	2447.24	828.41	2467.32	859.44	122.90	66.08	77.20	40.73	6.19	10.12	4.07	6.21		
Italy	2442.56	904.50	2311.45	873.81	84.39	42.68	62.67	30.54	3.45	4.72	2.71	3.50	1.37	1.29
Lower bound 95% CI	2115.12	788.10	1929.84	736.94	76.24	33.67	56.25	23.66	2.72	3.23	2.04	2.29		
Upper bound 95% CI	2807.85	1042.82	2755.29	1034.08	88.45	47.31	66.04	34.14	4.18	6.00	3.42	4.63		
Latvia	3990.15	2020.97	4218.38	2363.87	342.39	179.90	231.22	117.63	8.58	8.90	5.48	4.98	1.04	0.91
Lower bound 95% CI	3646.19	1785.94	3745.79	2077.92	325.19	161.76	206.24	101.50	7.42	7.12	4.37	3.76		
Upper bound 95% CI	4379.69	2272.89	4716.06	2701.01	356.15	191.70	256.30	130.78	9.77	10.73	6.84	6.29		
Lithuania	4102.93	2014.47	4161.43	2307.65	382.01	208.53	272.55	150.43	9.31	10.35	6.55	6.52	1.11	1.00
Lower bound 95% CI	3825.23	1811.56	3710.65	2052.94	362.81	186.88	245.79	131.76	8.17	8.33	5.32	5.06		
Upper bound 95% CI	4438.85	2243.53	4623.86	2606.41	394.92	219.53	297.43	166.88	10.32	12.12	8.02	8.13		
Luxembourg	1576.93	611.91	1837.68	686.90	91.82	44.55	64.94	30.83	5.82	7.28	3.53	4.49	1.25	1.27
Lower bound 95% CI	1391.20	519.62	1602.90	585.00	84.87	39.40	58.56	25.94	4.77	5.43	2.80	3.28		
Upper bound 95% CI	1780.21	725.53	2091.87	790.69	97.46	48.02	71.27	34.44	7.01	9.24	4.45	5.89		
Malta	1846.86	586.14	1963.08	642.44	140.23	86.02	83.45	59.81	7.59	14.68	4.25	9.31	1.93	2.19
Lower bound 95% CI	1638.50	504.26	1690.74	548.99	127.86	73.51	74.29	49.31	6.07	10.76	3.33	6.67		
Upper bound 95% CI	2104.77	683.07	2232.93	739.14	149.08	94.00	91.33	67.23	9.10	18.64	5.40	12.25		
The Netherlands	2369.78	814.56	2259.59	770.04	69.73	33.43	53.62	25.18	2.94	4.10	2.37	3.27	1.39	1.38
Lower bound 95% CI	2146.71	726.82	1975.58	675.04	63.65	28.36	48.51	20.60	2.42	3.10	1.89	2.35		
Upper bound 95% CI	2633.54	913.38	2573.06	875.20	73.88	36.42	57.25	27.79	3.44	5.01	2.90	4.12		
Poland	3962.14	2136.90	3941.87	2128.66	188.37	100.98	153.84	84.03	4.75	4.73	3.90	3.95	0.99	1.01
Lower bound 95% CI	3434.32	1848.91	3351.71	1789.06	178.42	89.40	138.61	71.83	3.95	3.63	2.99	2.85		
Upper bound 95% CI	4515.47	2465.62	4633.79	2522.73	194.05	106.65	168.39	92.43	5.65	5.77	5.02	5.17		
Portugal	1466.11	486.64	1454.98	532.96	58.79	34.32	50.47	25.62	4.01	7.05	3.47	4.81	1.76	1.39
Lower bound 95% CI	1274.63	416.61	1251.73	433.62	54.19	28.71	46.47	20.93	3.23	5.04	2.76	3.22		
Upper bound 95% CI	1678.35	569.17	1683.91	649.06	61.90	37.27	53.36	28.22	4.86	8.94	4.26	6.51		
Romania	3866.03	2204.39	4133.15	2618.48	227.63	150.69	193.28	123.07	5.89	6.84	4.68	4.70	1.16	1.01
Lower bound 95% CI	3461.64	1989.83	3638.78	2345.76	216.99	137.14	175.08	110.27	5.00	5.61	3.77	3.78		
Upper bound 95% CI	4337.04	2445.44	4641.41	2918.78	234.97	158.21	212.41	135.86	6.79	7.95	5.84	5.79		
Slovakia	3703.14	1980.22	3867.17	2323.95	285.64	184.74	224.35	154.39	7.71	9.33	5.80	6.64	1.21	1.15
Lower bound 95% CI	3330.48	1781.59	3406.43	2076.56	267.87	165.38	199.85	131.22	6.44	7.47	4.64	5.03		
Upper bound 95% CI	4157.40	2212.79	4307.09	2610.02	300.36	199.50	250.47	174.39	9.02	11.20	7.35	8.40		
Slovenia	3433.70	1985.50	4061.45	2535.53	91.65	45.46	69.61	29.46	2.67	2.29	1.71	1.16	0.86	0.68
Lower bound 95% CI	3043.13	1747.10	3523.51	2227.00	84.42	38.76	61.34	23.50	2.16	1.71	1.34	0.82		
Upper bound 95% CI	3914.79	2262.18	4576.70	2855.64	97.97	49.57	77.44	33.82	3.22	2.84	2.20	1.52		
Spain	2106.33	723.44	1943.35	675.28	67.80	31.83	51.98	22.19	3.22	4.40	2.67	3.29	1.37	1.23
Lower bound 95% CI	1907.39	644.64	1720.30	592.69	62.18	26.05	47.04	17.35	2.68	3.21	2.18	2.26		
Upper bound 95% CI	2318.24	810.69	2160.39	766.67	71.62	35.09	54.94	24.99	3.75	5.44	3.19	4.22		
Sweden	1942.45	875.12	2062.33	890.68	112.12	61.10	68.12	37.80	5.77	6.98	3.30	4.24	1.21	1.28
Lower bound 95% CI	1683.86	755.82	1752.48	756.14	103.34	50.88	58.52	29.92	4.60	5.02	2.39	2.87		
Upper bound 95% CI	2245.91	1013.09	2443.82	1043.07	117.56	66.42	77.59	42.97	6.98	8.79	4.43	5.68		
European Union	2808.31	1152.41	2669.85	1159.08	114.88	63.31	87.28	46.62	4.09	5.49	3.27	4.02	1.34	1.23
Lower bound 95% CI	2534.62	1032.44	2361.33	1022.30	107.31	54.52	80.39	39.51	3.47	4.24	2.67	2.99		
Upper bound 95% CI	3089.45	1285.71	3006.43	1323.00	118.50	67.86	91.20	50.52	4.68	6.57	3.86	4.94		

ASMR, age-standardized mortality rate; ASPR, age-standardized prevalence rate; IHD, ischemic heart disease; MPR, mortality to prevalence ratio.

The GBD 2021 definition describes IHD as International Classification of Diseases (ICD) classes I20–25.9, namely: angina pectoris, acute myocardial infarction, subsequent ST elevation (STEMI) and non-ST elevation (NSTEMI) myocardial infarction, certain current complications following ST elevation (STEMI) and non-ST elevation (NSTEMI) myocardial infarction (within the 28 days), other acute ischemic heart disease, and chronic ischemic heart disease.

Data and definitions from Global Burden of Disease Database, 2021.

### 3.1 IHD mortality estimates

In 2021, ASMRs for IHD varied substantially across EU countries (*Table 1*, Supplementary material online, *Appendix Figure S1*). Among men, the highest ASMRs were in Lithuania (272.6 per 100 000), Bulgaria (267.2 per 100 000), and Latvia (231.2); among women, Bulgaria led with 155.9, followed by Slovakia (154.4) and Lithuania (150.4). In contrast, the lowest rates for men were observed in France (44.6), Portugal (50.5) and Spain (52.0); for women, in France (18.9), Spain (22.2), and Belgium (25.1) ASMRs were inversely correlated with TPI scores in both sexes (women: *r* = −0.64, *P* < 0.001; men: *r* = −0.61, *P* < 0.001) (see Supplementary material online, *Appendix Figure S2*).

### 3.2 IHD prevalence estimates

A similarly wide variation was observed in ASPRs across the EU in 2021 (*Table 1*, Supplementary material online, *Appendix Figure S3*). Estonia recorded the highest ASPR for both men (5007.3 per 100 000) and women (3208.5 per 100 000), followed by Hungary (men: 4573.2; women: 2684.3). Bulgaria ranked third for men (4368.5), and the Czech Republic for women (2634.3). The lowest ASPRs were seen in Portugal for both sexes (men: 1455.0; women: 533.0). ASPRs were inversely correlated with TPI scores in both men (*r* = −0.44, *P* = 0.02) and women (*r* = −0.46, *P* = 0.01) (see Supplementary material online, *Appendix Figure S4*).

### 3.3 Relationship between IHD prevalence and mortality

Pearson correlation analyses revealed a significant relationship between ASMR and ASPR in 2021 for both women (*r* = 0.74, *P* < 0.0001) and men (**r = 0.79*, *P** < 0.0001) (see Supplementary material online, *Appendix Figure S5*). This suggests that crude ASMRs may be more reflective of overall disease burden than of mortality risk per individual cases. Normalizing ASMR by ASPR (MPR) provided a more accurate measure of the probability of death among individuals diagnosed with IHD, offering a clinically relevant indicator of disease severity and/or healthcare system performance.

### 3.4 Mortality normalized for the prevalence of IHD

**Figure 1:**
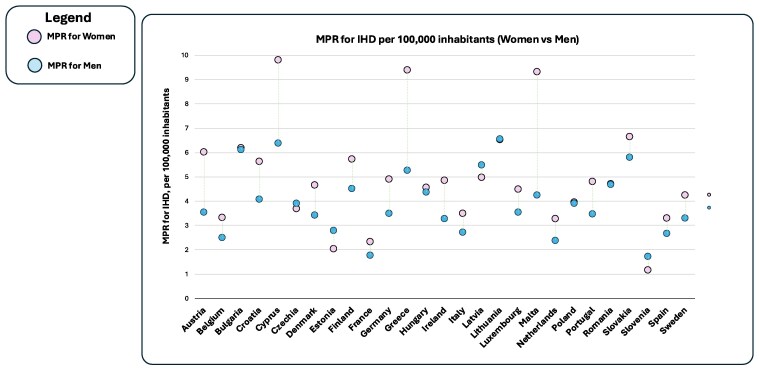
MPRs for IHD by sex across 27 EU countries in 2021. IHD, Ischemic Heart Disease; MPR, Mortality-Prevalence Ratio.

Between 2011 and 2021, MPRs declined across most EU countries, with the EU average falling from 4.09% to 3.27% in men and from 5.49% to 4.02% in women (*Table 1*). However, these averages obscure substantial country-specific differences. In 2021, the largest sex gaps were observed in Austria, Cyprus, Croatia, Greece, Ireland, and Malta, where IHD mortality among women exceeded that of men by 1.6% to 5.0% points. Smaller gaps (approximately 0.5% points or less) were noted in Bulgaria, Hungary, Poland, and Romania. Only five countries, the Czech Republic, Estonia, Latvia, Lithuania, and Slovenia, reported slightly higher MPRs in men. To visualize these differences, *Figure 1* shows the MPR in women vs. men for 27 EU countries in 2021. Cyprus, Malta, and Greece show marked excess in female IHD mortality, while Estonia, Slovenia, Latvia, Lithuania and the Czech Republic show near parity or reversed trends. Overall, these findings suggest that women in most EU countries experience a relatively higher IHD mortality burden than men. Wide UI, however, introduce variability in these estimates and warrant further analysis.

### 3.5 *Z*-score analysis for mortality normalized for the prevalence of IHD

**Figure 2:**
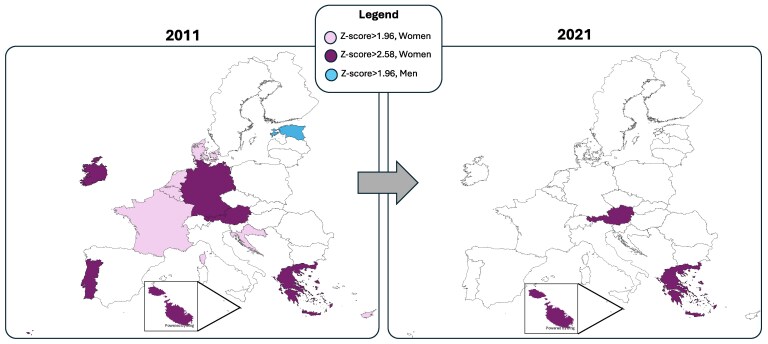
*Z*-Score analysis of sex differences in IHD MPRs, 2011–2021. IHD, Ischemic Heart Disease; MPR, Mortality-Prevalence Ratio; W/M, women-to-men.

In 2011, sex disparities in IHD outcomes were substantial, with women experiencing significantly worse outcomes in six countries (Austria, Germany, Greece, Ireland, Malta, and Portugal) with *Z*-scores exceeding 2.58, indicating statistically significant differences at the 99% confidence level. Eight other countries (Belgium, Croatia, Cyprus, Denmark, France and the Netherlands) exhibited *Z*-scores > 1.96, indicating statistically significant differences at the 95% confidence level (*Figure 2*, Supplementary material online, *Appendix Table S3*) Estonia was the only EU country where men had a significantly higher MPR than women, with significance at the 95% confidence level. By 2021, *Z*-score analysis suggested a narrowing of sex disparities in most countries. No country exhibited statistically significant differences in favor of men, as no *Z*-scores exceeded 1.96 (95% confidence). In contrast, disparities against women persisted. Austria, Greece, and Malta continued to show *Z*-scores above 2.58, maintaining statistical significance at the 99% confidence level, while other previously significant countries no longer retained statistically significant sex disparities (*Z*-scores < 1.96, 95% confidence level).

### 3.6 MPRs and TPI scores

Pearson correlation analyses indicated a non-significant relationship between the 2021 TPI and MPR for women (*r* = −0.35; *P* = 0.07), and a significant correlation in men (*r* = −0.57; *P* = 0.002), as shown in Supplementary material online, *Appendix Figure S6*. These findings suggest that economic transition, as measured by the TPI, does not strongly or consistently predict IHD outcome disparities by sex across European countries.

### 3.7 Proportion of IHD deaths attributable to risk factors

Across EU countries, most IHD deaths from modifiable risk factors occurred in men, reflecting their higher IHD prevalence. The exception was physical inactivity, which had a greater impact on women, contributing more to female IHD mortality (see Supplementary material online, *Appendix Tables S4*  to  *S23*). To complement this population-level analysis, we also assessed the CFI, a prevalence-adjusted metric estimating the mortality burden attributable to each risk factor among individuals with IHD. By accounting for disease burden, the CFI provides a disease-burden–weighted perspective, offering a clearer view of sex-specific vulnerability to risk factors.

### 3.8 Case fatality Index attributable to metabolic risk factors

**Figure 3:**
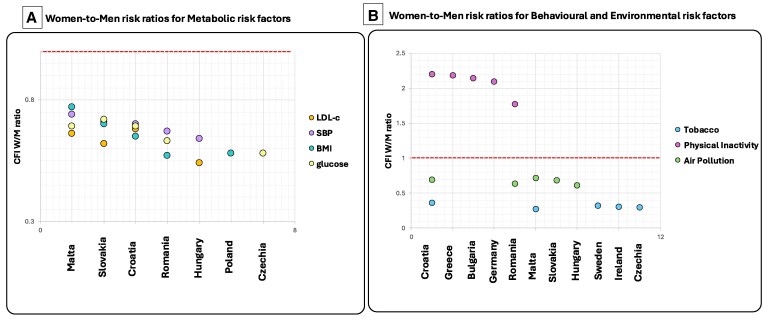
Top 5 countries with the highest 2021 female-to-male CFI ratios. (*A*) Metabolic; (*B*) Behavioral & Environmental risks. CFI, case-fatality index; W/M, women-to-men.

*Figure 3A* shows the top five EU countries with the highest women-to-men CFI ratios for IHD mortality attributable to key metabolic risk factors: high systolic blood pressure, fasting plasma glucose, LDL cholesterol, and BMI in 2021. Full country-level data are provided in Supplementary material online, *Appendix Tables S4*  to  *S7*. Women often exhibited higher CFI values than men (ratios >1.0), suggesting greater per-case vulnerability to metabolic risk factors. Malta stood out as a consistent outlier, with CFI ratios exceeding 2.0 for all four metabolic risks examined. Additional high female CFI ratios were observed in other Southern European countries, namely Greece and Cyprus, underscoring a regional pattern of elevated cardiometabolic burden among women.

### 3.9 Case fatality Index attributable to behavioral and environmental risk factors

*Figure 3B* presents the top five countries with the highest women-to-men CFI ratios for IHD mortality attributable to behavioral and environmental risk factors in 2021. Complete results are provided in Supplementary material online, *Appendix Tables S8*  to  *S10*. Malta and Greece led for air pollution–related mortality, with CFI ratios of 2.19 and 1.79, respectively, indicating a markedly higher per-case burden among women. Southern European women also bore a disproportionate burden from physical inactivity. In Greece, women with IHD experienced an almost 7-fold higher mortality burden from inactivity than men (CFI ratio = 6.88). In contrast, tobacco use posed significantly less risk for women, with most countries showing CFI ratios well below 1.0. An exception was Ireland, where the CFI ratio approached parity (CFI ratio = 0.91), suggesting narrowing sex differences in smoking-related IHD mortality.

### 3.10 Case fatality Index attributable to dietary risk factors

**Figure 4:**
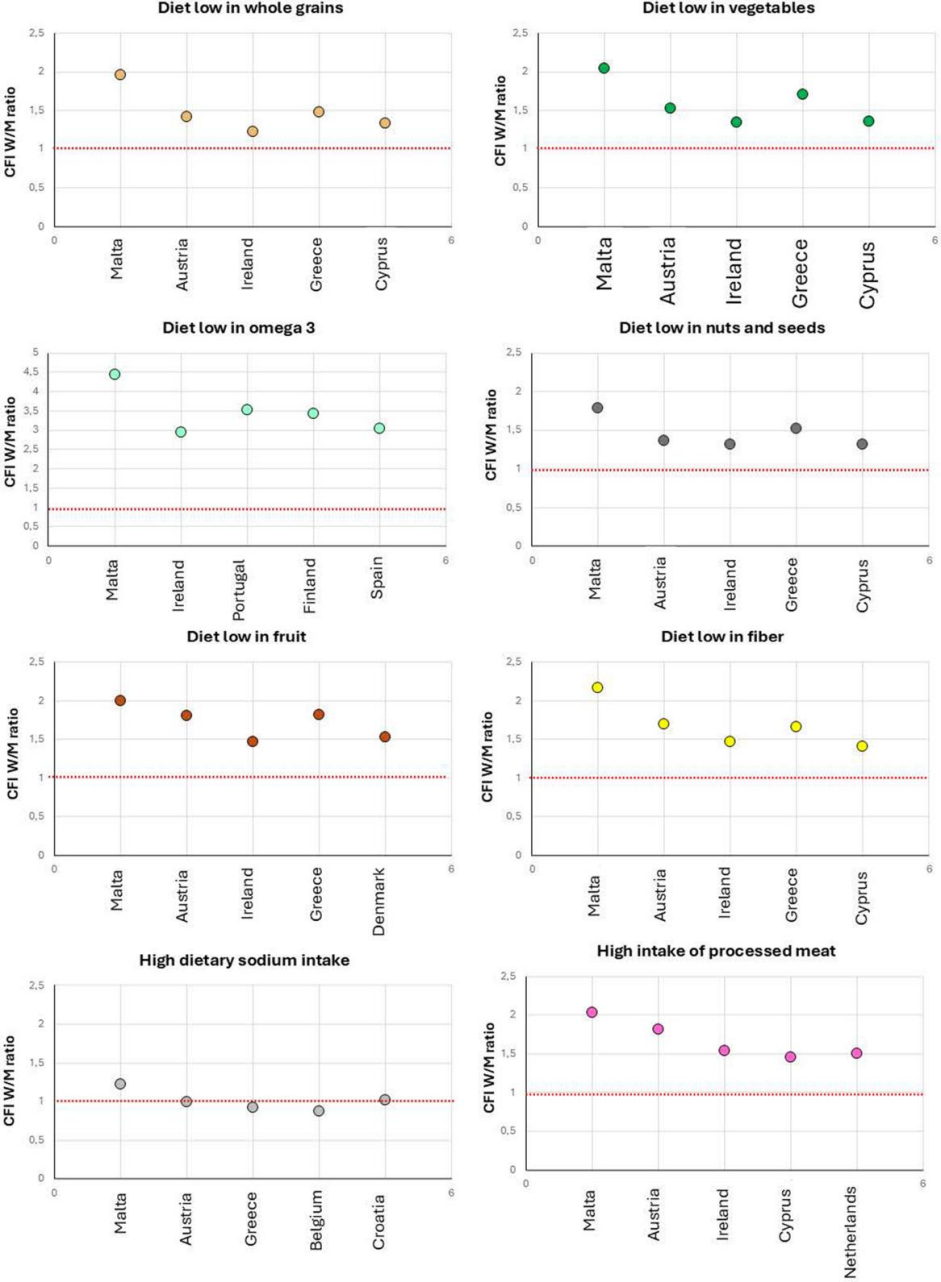
Top 5 countries with the highest female-to-male CFI ratios for diet-related IHD mortality (2021). CFI, case-fatality index; IHD, ischemic heart disease, W/M, women-to-men.

From 2011 to 2021, mortality rates attributable to most dietary risk factors declined across the EU, reflecting possible improvements in diet quality (see Supplementary material online, *Appendix Tables S11*  to  *S23*). However, among individuals with diagnosed IHD, women frequently exhibited higher mortality burdens than men for several dietary risks (*Figure 4*). Despite the region’s healthy diet reputation, Mediterranean women (Malta, Greece, Cyprus, Portugal) faced 40% to 60% higher mortality per IHD case from low fruit, fiber, vegetables, whole grains and nuts and seeds intake than men. In landlocked countries such as Hungary, Slovakia, and Austria, low seafood omega-3 intake was associated with disproportionate female mortality. High red and processed meat consumption harm women more in Central Europe with a 50% to 80% higher mortality per IHD case in the Netherlands and Austria. Eastern Europe (Bulgaria, Romania, Lithuania) leads in female IHD burden from vegetable-poor diets (CFI ratios 1.32–1.48). Ireland also showed marked sex disparities across several dietary risks, including a diet low in whole grains, vegetables, omega-3, nuts and seeds, fruit, fiber, as well as a diet high in processed meat.

### 3.11 *Z*-score analysis for IHD risk factors

Due to wide UI in the estimates of mortality attributable to risk factors, formal *Z*-score testing was used to assess statistically significant sex differences in risk for 2021.

### 3.12 *Z*-score analysis for metabolic risk factors

**Figure 5:**
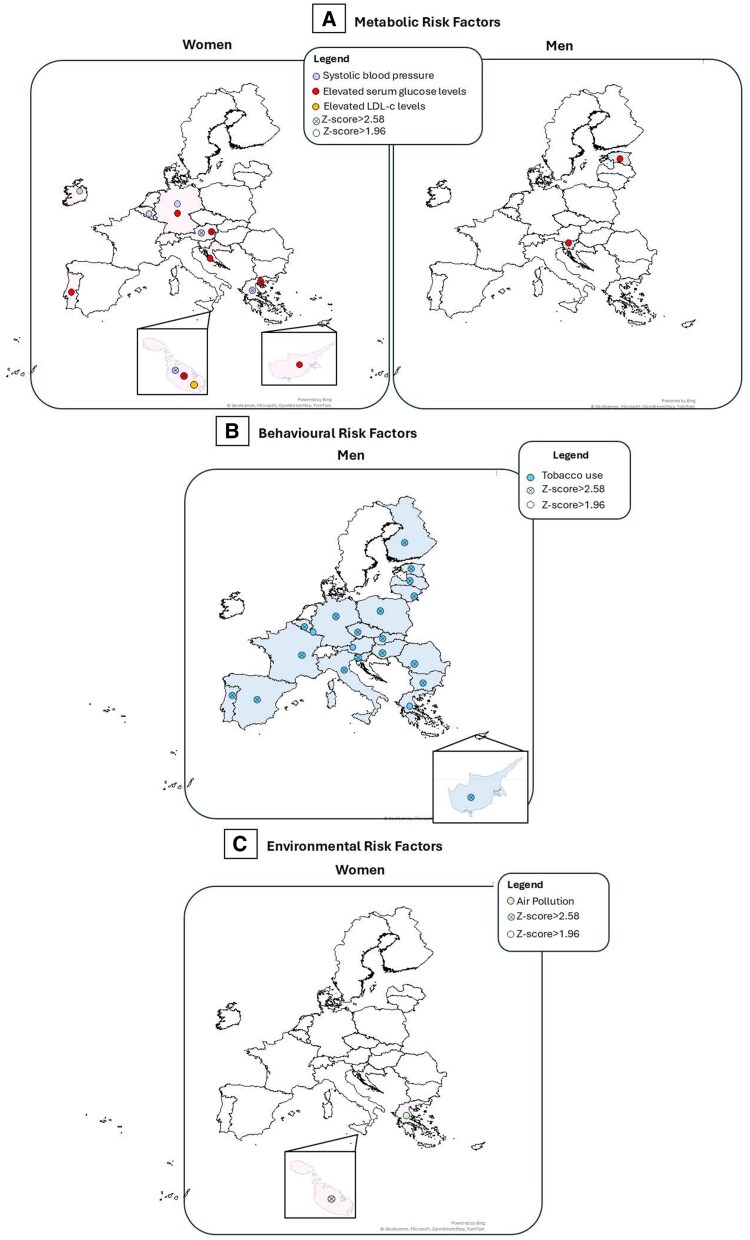
Z-score analysis of sex differences in IHD mortality in 2021: (*A*) metabolic, (*B*). Behavioral, (*C*). Environmental risk factors.

In 2021, Greece, Malta and Austria emerged as the only EU countries where women exhibited remarkably higher IHD mortality across multiple metabolic risk factors with *Z*-scores > 2.58 indicating strong statistical significance. These disparities were attributable to systolic blood pressure and fasting plasma glucose (*Figure 5A* and Supplementary material online, *Appendix Tables S24*  to  *S27*). Malta also showed a significant female disadvantage for high LDL cholesterol (|*Z*| > 1.96). Germany demonstrated a multi-risk sex disparity as well, with women showing excess mortality related to both elevated serum glucose levels and systolic blood pressure, though *Z*-scores ranged from 1.96 to 2.58, reflecting a moderate level of statistical significance. Other countries showing isolated female disadvantages (|*Z*| > 1.96) included Belgium, Ireland (systolic blood pressure), and Croatia, Cyprus, and Portugal (plasma fasting glucose). In contrast, Slovenia and Estonia displayed a reverse trend, with significantly higher IHD mortality in men due to fasting plasma glucose (|*Z*| > 1.96), suggesting a divergent regional pattern of sex disparities in metabolic risk. However, none of these countries showed the multi-factor clustering or magnitude of disparity seen in Greece, Malta, Austria, or Germany.

### 3.13 *Z*-score analysis for behavioral risk factors

Tobacco use–related IHD mortality was significantly lower in women across most EU countries (|*Z*| > 2.58), with the smallest gap in Sweden (4.8% vs. 6.3% per 10 000 individuals living with IHD) Physical inactivity showed less consistent sex disparities; countries like Austria and Malta showed higher female mortality, but differences were not statistically significant (|*Z*| < 1.96) (*Figure 5B*, Supplementary material online, *Appendix Tables S28* and *S29*).

### 3.14 *Z*-score analysis for environmental risk factors

*Figure 5C* displays countries exceeding the 95% confidence threshold (|*Z*| > 1.96) for sex disparities in IHD mortality attributable to air pollution in 2021. Greece and Malta exhibited the largest female disadvantage, with women experiencing 1.8 to 2.2 times higher mortality than men. (see Supplementary material online, *Appendix Table S30*).

### 3.15 *Z*-score analysis for diet-driven mortality

*Z*-score analysis identified significant geographic and sex-specific disparities in ischemic heart disease (IHD) mortality attributable to dietary factors across 27 EU countries in 2021. The analysis included dietary components with valid 95% UI; error bars represent these intervals. Risk factors with non-positive lower bounds, such as low intake of omega-6 polyunsaturated fats, legumes, sugar-sweetened beverages, and red meat, were excluded due to statistical instability. Seven key dietary risks reached strong significance (|*Z*| > 2.58), including low intake of whole grains, vegetables, nuts/seeds, seafood omega-3 s, and fiber, and high intake of processed meat and sodium (see Supplementary material online, *Appendix Tables S31*  to  *S39*).

Clustering of dietary risk factors was most pronounced among Mediterranean women. In Malta and Greece, markedly elevated IHD mortality was associated with low intake of omega-3 fatty acids (|*Z*| = 4.67 and |*Z*| = 3.56, respectively) and nuts/seeds (|*Z*| = 4.12 and |*Z*| = 2.89, respectively). Additional excess burden was linked to low vegetable intake in Malta (|*Z*| = 3.12) and Greece (|*Z*| = 2.89), fiber deficiency in Malta (|*Z*| = 4.92) and Greece (|*Z*| = 4.67), and high processed meat consumption in Greece (|*Z*| = 3.05). Outside the Mediterranean region, significant excess female IHD mortality was associated with low omega-3 intake in Hungary (|*Z*| = 3.56), France (|*Z*| = 3.29), and Austria (|*Z*| = 3.21); high processed meat intake in Denmark (|*Z*| = 3.12); and high sodium consumption in Bulgaria (|*Z*| = 2.89).

In contrast, diet-attributable mortality burdens among men were concentrated in the Baltic region. Processed meat consumption was associated with extreme risk in Lithuania (*Z* = 5.01), Latvia (|*Z*| = 4.87), and Estonia (|*Z*| = 5.92). Low whole grain intake contributed to elevated mortality in Lithuania (|*Z*| = 5.45), while low fiber intake was a major driver in Estonia (|*Z*| = 5.01), the Czech Republic (|*Z*| = 4.01), and Latvia (|*Z*| = 5.89).

## 4. Discussion

The principal finding of this analysis is that, despite a substantial decline in sex disparities in IHD mortality across EU countries from 2011 to 2021, significant regional inequities persist. By 2021, Austria, Greece, and Malta continued to show significantly higher MPRs among women, reflecting a persistent and disproportionate IHD mortality burden. In these countries, excess female mortality was largely attributable to modifiable metabolic risks, mainly elevated systolic blood pressure and fasting plasma glucose, alongside unfavorable dietary patterns, including low intake of vegetables, nuts/seeds, fibers, and omega-3 fatty acids, as well as high consumption of processed meat. No clear inverse association was observed between national income and sex disparities in IHD mortality, suggesting that economic status alone does not account for the differences in cardiovascular outcomes between women and men.

### 4.1 *Z*-score analysis and excess female mortality

Statistical significance was assessed using *Z*-score testing, a conservative approach designed to detect only the most robust differences. When applied across 27 EU countries in 2021, this approach identified significantly higher MPRs among women in Austria, Greece, and Malta (|*Z*| > 2.56). However, the strict reliance on this threshold may limit sensitivity for detecting clinically meaningful disparities. Other six countries exhibited MPRs more than 5% higher in women: Belgium, Croatia, Cyprus, Denmark, France, and the Netherlands, suggesting that sex inequities may be more widespread than those identified by the conservative statistical criteria. This interpretation is further supported by aggregated EU data. In 2021, the ASPR for IHD was approximately 2.3 times higher in men than in women, while the ASMR was only 1.8 times higher. This divergence implies that, relative to their disease burden, women may experience a higher case fatality than men, suggesting potential disparities in access to care, treatment effectiveness, health-related behaviors, or underlying biological vulnerability.

### 4.2 Metabolic risk factors: Major health threats for women

Persistent sex disparities in IHD mortality observed in parts of Central and Southern Europe, such as Austria, Greece, and Malta, appear closely linked to the burden of metabolic risk factors among women in these regions. These three countries stood out as the only EU member states where women exhibited significantly higher IHD mortality attributable to multiple metabolic risk factors (|*Z*| > 2.58) compared with men. Excess female mortality was strongly associated with elevated systolic blood pressure and high fasting plasma glucose, with Malta also demonstrating a pronounced female disadvantage related to elevated LDL cholesterol. These findings are consistent with prior research indicating that the coexistence of two or more metabolic risk factors significantly amplifies the overall cardiovascular risk.^6,7^

### 4.3 High fasting plasma glucose: a disproportionate burden on women

Beyond the sheer number of metabolic risk factors, the specific clustering and severity of these risks appear to underlie the excess IHD mortality observed among women in Austria, Malta, and Greece. In these countries, elevated fasting plasma glucose, a marker of diabetes and prediabetes, emerged as a particularly significant contributor to female IHD mortality (|*Z*|>2.58). This finding aligns with robust epidemiologic evidence showing that diabetes disproportionately increases the risk of fatal IHD in women, with meta-analyses indicating a > 40% higher relative risk of fatal coronary heart disease in diabetic women compared with men. This disparity begins even before diabetes is formally diagnosed. In the prediabetic state, women already exhibit a more adverse cardiometabolic profile, including higher blood pressure relative to men. Biological factors, including heightened endothelial dysfunction and impaired fibrinolysis during the prediabetic phase, may amplify sex differences in risk.^8^ In the present analysis, coexisting hypertension and hyperglycemia (both |*Z*|>2.58) was a defining feature among women in Austria, Malta, and Greece. These risk factors are known to act synergistically, raising IHD risk by 59% in men and 78% in women, further suggesting compounded vulnerability among women.

### 4.4 Regional dietary patterns and their impact on IHD

The striking sex disparity in metabolic risk observed in Austria, Malta, and Greece points to the influence of sex-specific modifiable lifestyle and environmental contributors. Among these, diet emerged as a critical factor. Poor dietary quality is a well-established upstream driver of both hypertension and type 2 diabetes and ultimately, IHD mortality.^9–11^ In our study, significant sex disparities were observed in relation to low intake of omega-3 fatty acids, nuts/seeds, fibers, and vegetables, as well as high consumption of processed meat. High processed meat intake was common among women in Greece, Malta, and Austria, while the impact of protective dietary factors varied regionally, reflecting diverse socio-cultural and economic contexts.

### 4.5 The paradox of Mediterranean women abandoning the Mediterranean diet

Despite a longstanding cultural association with the Mediterranean diet, women in Southern Europe, specifically in Greece and Malta, show a surprising deviation from this cardioprotective dietary pattern. This shift is reflected in elevated IHD mortality attributable to low intake of vegetables, nuts/seeds, fibers and seafood omega-3 fatty acids, all core components of the Mediterranean diet. This dietary pattern has consistently demonstrated benefits in reducing LDL cholesterol, blood pressure, obesity, and the incidence and severity of type 2 diabetes, as demonstrated in both observational and interventional studies.^12^ On this background, our analysis reveals a public health paradox: regions once exemplary for heart-healthy eating habits now face rising IHD mortality risks among women, linked in part by dietary deterioration. Re-engagement with the principles of Mediterranean nutrition, particularly among at-risk female populations is needed, not merely as a cultural remainder, but as a public health strategy.

### 4.6 Omega-3 deficiency and Austria’s IHD sex gap

Austria presents a distinct case in the European landscape of IHD sex disparities. Unlike Greece and Malta, where excess female mortality may be driven by a clustering of protective dietary risk factors influencing various metabolic pathways, Austria’s appears more narrowly associated with a single protective factor: low omega-3 intake. Austrian women showed significantly higher IHD mortality primarily attributable to low omega-3 intake (|*Z*|=3.21). This pattern suggests that even isolated but potent nutritional deficiencies may disproportionately affect women’s cardiovascular outcomes.^13^ While Austria may not require re-engagement with Mediterranean dietary principles per se, targeted strategies to increase omega-3 intake through diet or supplementation could offer an effective strategy to reduce sex disparities in IHD mortality.

### 4.7 Sex-specific biological vulnerabilities and nutritional gaps

The disproportionate burden of multiple dietary deficiencies among women likely reflects an intersection of biological, socio-cultural, and economic factors that affect women's nutritional status across their lifespan. From a biological perspective, women have elevated nutritional needs due to reproductive functions. Pregnancy and lactation increase omega-3 demands for fetal neurodevelopment and maternal cardiovascular health.^14^ During menopause, declining estrogen levels impair omega-3 incorporation into cell membranes, potentially increasing susceptibility to inflammation and endothelial dysfunction.^15^ Magnesium requirements increase in women during and after menopause, making magnesium-rich foods, such as nuts, seeds, and whole grains, critical for maintaining vascular tone, blood pressure regulation, and insulin sensitivity.^16^ Women’s higher body fat percentage increases oxidative stress, increasing their reliance on antioxidant-rich vegetables. Combined lack of vegetables and nuts/seeds disrupts sodium balance and gut microbiota, both of which are associated with elevated blood pressure and glucose dysregulation.^17^ These physiological differences mean that women require more nutrient-dense diets yet often receive fewer essential nutrients.

### 4.8 Intersecting inequities

Beyond biological mechanisms, broader social determinants of health substantially compound sex disparities in cardiovascular risk. Poverty, education gaps, and exclusion disproportionately affect women’s cardiovascular health in socioeconomically vulnerable regions. According to the 2021 EU Survey on Income and Living Conditions (EU-SILC), approximately 25% of the population in Malta and Greece is at risk of poverty or social exclusion, conditions strongly associated with food insecurity, poor dietary quality, and limited access to preventive care.^18^ Cultural stigmas may discourage women from consuming nutrient-rich, higher-cost foods, such as fish and nuts, which are often reserved for male household members. Addressing these disparities requires multifaceted interventions including targeted food assistance programs, nutrition education initiatives, workplace meal supports, and policies challenging gendered food distribution norms.

### 4.9 Additional consideration: underestimation of female IHD prevalence

An alternative explanation for Malta and Austria’s elevated female MPR is the potential underdiagnosis of IHD in women. If fewer women are formally diagnosed with IHD despite having the condition, the denominator in the MPR calculation (prevalence) would be artificially low, thus inflating the apparent mortality burden. Underdiagnosis is a well-documented issue in female cardiovascular care and is often linked to atypical symptom presentation, diagnostic bias, and delayed care-seeking among women. This issue may be amplified in Malta and Austria, both of which have among the highest proportions of foreign-born residents in the EU, approximately 30% and 22%, respectively (see Supplementary material online, *Appendix Table S40*).^19^ Linguistic, cultural, and health literacy barriers may limit access to diagnosis and care, particularly among immigrant women.^20^ This dynamic contrasts with Greece, where the foreign-born population is relatively small (approximately 7%), making underdiagnosis due to migration-related barriers a less likely explanation.

### 4.10 Air pollution and women’s cardiovascular risk

In addition to dietary and metabolic factors, environmental exposures may also play a role in the observed sex disparities in IHD mortality. In our study, exposure to air pollution emerged as a significant (|*Z*|>1.96) environmental risk factor for female IHD mortality in Greece and Malta. Women in these countries may bear a disproportionate burden due to their roles in household activities, including cooking and heating with polluting fuels. Household air pollution from solid fuels, such as wood, charcoal, and coal, remains a significant issue, especially in areas where access to clean fuel devices is limited by financial constraints.^21,22^

In the GBD framework, exposure to air pollution, including both ambient (outdoor) and household (indoor) pollution, is estimated using satellite-based measurements, chemical transport models, and ground-level monitoring data, integrated through data fusion techniques to produce geographically resolved exposure estimates.^5^ Exposure-response relationships are modeled using the TMREL, derived from epidemiological studies, to quantify risk-attributable mortality for each population subgroup.

While the GBD framework does not distinguish whether excess risk arises from differential exposure, increased biological susceptibility, or a combination of both, these findings underscore a need to better account for domestic environmental exposures in cardiovascular risk assessments. These exposures are often unaccounted for in conventional risk assessments but may contribute meaningfully to cardiovascular risk in women.

### 4.11 Limitations

This study has several limitations. First, it shares the inherent constraints of the GBD methodology. The absence of individual-level data on death certification practices may affect the accuracy of cause-of-death attribution. Second, estimates may be biased by data quality and completeness, particularly in regions where vital registration systems are limited. In such settings, GBD relies on predictive covariates to interpolate values from neighboring countries, which may introduce regional bias. Third, the conservative bounding method used to estimate uncertainty in sex ratios assumes independence between estimates, potentially underestimating true variability. Fourth, dietary risk factors are not mutually exclusive, raising the possibility of double-counting and inflating the diet-attributable burden of IHD. However, while individual-level diet–disease associations cannot be inferred, these modeled estimates allow for consistent cross-country comparisons of population-level dietary risk burdens. Fifth, several modifiable and non-modifiable risk factors, such as clinical management, medication adherence, healthcare access, and genetic predisposition, were not included, reflecting limitations in the GBD comparative risk framework. However, current. registries, although rich in clinical detail, often reflect selected patient populations, span heterogeneous timeframes, and vary in data completeness, Sixth, the analysis does not account for structural determinants of health, including social deprivation, rurality, and geographic barriers, which likely interact with sex to shape IHD outcomes. Finally, we could not differentiate IHD subtypes, despite evidence that sex disparities in outcomes are particularly pronounced in ST-elevation myocardial infarction.^1,2^

Translational perspective

- Sex disparities in IHD mortality are not solely explained by differences in disease prevalence; they reflect inequities in modifiable risk factor exposures and limited access to healthy nutrition.
- Targeted prevention strategies should integrate sex-sensitive dietary interventions and improved cardiometabolic risk screening, especially in high-burden regions.
- Public health policies should support equitable access to nutrient-rich foods and prioritize cardiovascular risk reduction in underserved women.
- Greater emphasis on nutrition and sex-specific care in clinical guidelines could contribute to reducing sex disparities in IHD mortality across Europe.

## 5. Conclusion

Sex disparities in IHD mortality remain a major public health issue across the Europe, with substantial impact observed in Central, and South-Eastern European regions where Austria, Greece and Malta, continue to experience the highest burden. This persistent inequality reflects a structural failure to address sex- and region-specific risk factors, though our ecological design precludes causal attribution.

Our analysis identifies strong associations between poor diet, elevated blood pressure, and hyperglycemia, on women’s cardiovascular outcomes. While affordable antihypertensive therapies are widely available and access to newer antidiabetic agents is improving, prevention remains the most effective strategy to reduce excess female IHD mortality. Prioritizing public health interventions in high-burden countries may reduce sex-based gaps and improve cardiovascular outcomes in Europe.

## Supplementary Material

cvaf176_Supplementary_Data

## Data Availability

To download the data used in these analyses, please visit the Global Health Data Exchange GBD 2021 website at https://ghdx.healthdata.org/gbd-2021.
