# Supplementary material for: Diet and sex inequities in ischemic heart disease mortality across Europe: findings from the global burden of disease study
Source: Cardiovasc Res. 2025 Nov 3;121(15):2432–46. doi: 10.1093/cvr/cvaf176 (PMC12687866; doi:10.1093/cvr/cvaf176)
Supplement: cvaf176_Supplementary_Data [file cvaf176_supplementary_data.docx]

**Appendix -****Supplementary Data-**

**Diet and Sex Inequities in Ischemic Heart Disease Mortality Across Europe:**

**Findings from the Global Burden of Disease Study**

**Address for Correspondence:**

Raffaele Bugiardini, MD, PHD, FESC, FACC, FAHA.

Department of Experimental, Diagnostic and Specialty Medicine, University of Bologna, Bologna, Italy.

Address: Via Giuseppe Massarenti 9, 40138, Bologna, Italy.

Phone and fax: +39 051347290. E-mail: raffaele.bugiardini@unibo.it

Contents

[Global Burden of Disease Overview 5](#_Toc205977468)

[Normalizing IHD Mortality by Prevalence. 6](#_Toc205977469)

[**Analytical Design and Temporal Scope 7**](#_Toc205977470)

[**Statistical Analysis. 7**](#_Toc205977472)

[1. Mortality-to-Prevalence Ratios (MPRs) 7](#_Toc205977473)

[2. Risk-Attributable Mortality Normalized by IHD Prevalence. 8](#_Toc205977474)

[3. Z-Test for Comparing Sex Ratios Across Populations 9](#_Toc205977475)

[**Ischemic heart disease (IHD) definition: 10**](#_Toc205977476)

[Case Definitions 10](#_Toc205977477)

[**Terminology 11**](#_Toc205977478)

[**Transition Performance Index (TPI) 11**](#_Toc205977479)

[**Appendix Figure 1. Trends in Age-Standardized Mortality Rates (ASMR) and Transition Performance Index (TPI) for IHD, Per 100,000 Inhabitants, Stratified by Country and Sex 13**](#_Toc205977480)

[**Appendix Figure 2: Correlation Between Age-Standardized Mortality Rates (ASMR) and Transition Performance Index (TPI) for Ischemic Heart Disease (IHD) in 2021. Regression line and 95%UI visualised. 14**](#_Toc205977481)

[***Data derived from Global Burden of Diseases, year 2021* 14**](#_Toc205977482)

[**Appendix Figure 3. Trends in Age-Standardized Prevalence Rates for IHD, Per 100,000 Inhabitants, Stratified by Country and Sex 15**](#_Toc205977483)

[**Appendix Figure 4: Correlation Between Age-Standardized Prevalence Rates (ASMR) and Transition Performance Index (TPI) for Ischemic Heart Disease (IHD) in 2021 16**](#_Toc205977484)

[**Appendix Figure 5: Correlation Between Age-Standardized Mortality Rates (ASMR) and Age-Standardized Prevalence Rates for Ischemic Heart Disease (IHD) in 2021, in women and in men. 17**](#_Toc205977485)

[**Appendix Figure 6. Correlation Between Mortality-Prevalence ratios (MPR) for Ischemic Heart Disease (IHD) and Transition Performance Index in 2021, in women and in men. 18**](#_Toc205977486)

[**Appendix Table 1: Economic Transition Index 2021 by Country 19**](#_Toc205977487)

[**Appendix Table 2: Dietary risk factor exposure GBD definitions and optimal level of exposure as defined by GBD 2021. 21**](#_Toc205977488)

[**Appendix Table 3. Sex-stratified distribution of Z scores based on MPR for IHD across European Countries (Years 2011 vs 2021). 23**](#_Toc205977489)

[**Appendix Table 4. Age-standardised mortality rates and case fatality indexes for IHD attributable to High LDL cholesterol. 2011 , 2021 24**](#_Toc205977490)

[**Appendix Table 5. Age-standardised mortality rates and case fatality indexes for IHD attributable to High systolic blood pressure.2011 , 2021 29**](#_Toc205977491)

[**Appendix Table 6. Age-standardised mortality rates and case fatality indexes for IHD attributable to High body-mass index. 34**](#_Toc205977492)

[**2011 , 2021 34**](#_Toc205977493)

[**Appendix Table 7. Age-standardised mortality rates and case fatality indexes for IHD attributable to High fasting plasma glucose. 2011 , 2021 39**](#_Toc205977494)

[**Appendix Table 8. Age-standardised mortality rates and case fatality indexes for IHD attributable to Tobacco. 2011 , 2021 46**](#_Toc205977495)

[**Appendix Table 9. Age-standardised mortality rates and case fatality indexes for IHD attributable to Air pollution. 2011 , 2021 51**](#_Toc205977496)

[**Appendix Table 10. Age-standardised mortality rates and case fatality indexes for IHD attributable to Low physical activity. 2011 , 2021 55**](#_Toc205977497)

[**Appendix Table 11. Age-standardised mortality rates and case fatality indexes for IHD attributable to Diet high in processed meat. 2011, 2021 58**](#_Toc205977498)

[**Appendix Table 12. Age-standardised mortality rates and case fatality indexes for IHD attributable to Diet high in red meat. 2011 , 2021 62**](#_Toc205977499)

[**Appendix Table 13. Age-standardised mortality rates and case fatality indexes for IHD attributable to Diet high in sodium. 2011 , 2021 65**](#_Toc205977500)

[**Appendix Table 14. Age-standardised mortality rates and case fatality indexes for IHD attributable to Diet high in sugar-sweetened beverages. 2011 , 2021 69**](#_Toc205977501)

[**Appendix Table 15. Age-standardised mortality rates and case fatality indexes for IHD attributable to Diet high in trans fatty acids. 2011 , 2021 74**](#_Toc205977502)

[**Appendix Table 16. Age-standardised mortality rates and case fatality indexes for IHD attributable to Diet low in fiber. 78**](#_Toc205977503)

[**2011 , 2021 78**](#_Toc205977504)

[**Appendix Table 17. Age-standardised mortality rates and case fatality indexes for IHD attributable to Diet low in fruits. 83**](#_Toc205977505)

[**2011 , 2021 83**](#_Toc205977506)

[**Appendix Table 18. Age-standardised mortality rates and case fatality indexes for IHD attributable to Diet low in legumes. 86**](#_Toc205977507)

[**2011 , 2021 86**](#_Toc205977508)

[**Appendix Table 19. Age-standardised mortality rates and case fatality indexes for IHD attributable to Diet low in nuts and seeds. 89**](#_Toc205977509)

[**2011 , 2021 89**](#_Toc205977510)

[**Appendix Table 20. Age-standardised mortality rates and case fatality indexes for IHD attributable to Diet low in omega-6 polyunsaturated fatty acids. 2011 , 2021 94**](#_Toc205977511)

[**Appendix Table 21. Age-standardised mortality rates and case fatality indexes for IHD attributable to Diet low in seafood omega-3 fatty acids. 98**](#_Toc205977512)

[**2011 , 2021 98**](#_Toc205977513)

[**Appendix Table 22. Age-standardised mortality rates and case fatality indexes for IHD attributable to Diet low in vegetables. 2011 , 2021 102**](#_Toc205977514)

[**Appendix Table 23. Age-standardised mortality rates and case fatality indexes for IHD attributable to Diet low in whole grains. 2011 , 2021 106**](#_Toc205977515)

[**Appendix Table 24. Z scores for Case Fatality Index attributable to High LDL cholesterol. 2021. 109**](#_Toc205977516)

[**Appendix Table 25. Z scores for Case Fatality Index attributable to High systolic blood pressure. 2021. 111**](#_Toc205977517)

[**Appendix Table 26. Z scores for Case Fatality Index attributable to High body-mass index. 2021. 113**](#_Toc205977518)

[**Appendix Table 27. Z scores for Case Fatality Index attributable to High fasting plasma glucose. 2021. 115**](#_Toc205977519)

[**Appendix Table 28. Z scores for Case Fatality Index attributable to Tobacco. 2021. 117**](#_Toc205977520)

[**Appendix Table 29. Z scores for Case Fatality Index attributable to Low physical activity. 2021. 119**](#_Toc205977521)

[**Appendix Table 30. Z scores for Case Fatality Index attributable to Air pollution. 2021. 121**](#_Toc205977522)

[**Appendix Table 32. Z values for Case Fatality Index attributable to Diet high in processed meat. 2021. 125**](#_Toc205977523)

[**Appendix Table 33. Z values for Case Fatality Index attributable to Diet low in fiber. 2021. 127**](#_Toc205977524)

[**Appendix Table 34. Z values for Case Fatality Index attributable to Diet low in whole grain. 2021. 129**](#_Toc205977525)

[**Appendix Table 35. Z values for Case Fatality Index attributable to Diet low in vegetables. 2021. 131**](#_Toc205977526)

[**Appendix Table 36. Z values for Case Fatality Index attributable to Diet low in nuts and seeds. 2021. 133**](#_Toc205977527)

[**Appendix Table 37. Z values for Case Fatality Index attributable to Diet low in seafood omega-3 fatty acids. 2021. 135**](#_Toc205977528)

[**Appendix Table 38. Z values for Case Fatality Index attributable to Diet low in fruits. 2021. 137**](#_Toc205977529)

[**Appendix Table 39. Z values for Case Fatality Index attributable to Diet high in sodium. 2021. 139**](#_Toc205977530)

[**Appendix Table 41: Detailed methodology and data sources: GATHER Statement^1^ 143**](#_Toc205977531)

[**References 144**](#_Toc205977532)

## **Global Burden of Disease Overview**

The Global Burden of Disease (GBD) Study 2021 provides a comprehensive evaluation of disease burden, covering 371 diseases across 204 countries and territories from 1990 to 2021. It generates various metrics, including prevalence rates and cause-specific mortality, adhering to the Guidelines for Accurate and Transparent Health Estimates Reporting (GATHER).^1^ See Appendix Table 41 for the GATHER checklist.

To expand available data and produce outcomes for the global population, GBD employs a range of modelling tools. This modelling is conducted at the 1000 draw level, with point estimates calculated as the mean of these draws, and 95% uncertainty intervals (UIs) derived from the 2.5th and 97.5th percentiles.^2^

Using the GBD 2021 global population age standard, age-standardized rates are calculated to facilitate comparisons across different locations and time periods with varying age distributions.^3,4^ In countries with robust vital registration systems characterized by high completeness and minimal errors, vital registration serves as the primary data source for causes of death. The majority of causes of death data is obtained from vital registration sources, including those submitted to the World Health Organization (WHO) Mortality Database, as well as country-specific mortality databases managed by national authorities.

Two types of risk factor estimation are possible within the GBD framework: attributable burden and avoidable burden. Attributable burden refers to the reduction in current disease burden that would have occurred if past exposure had matched an alternative (counterfactual) risk distribution. In contrast, avoidable burden reflects the potential future reduction in disease if current exposures are modified. Murray and Lopez identified four counterfactual exposure levels: (1) theoretical minimum risk, (2) plausible minimum risk, (3) feasible minimum risk, and (4) cost-effective minimum risk. The theoretical minimum risk exposure level (TMREL) is the exposure level associated with the lowest population risk and thus captures the maximum attributable burden of death. ^5^ Other benchmarks include plausible minimum risk (conceivably achievable), feasible minimum risk (the lowest observed in any population), and cost-effective minimum risk (achievable within economic constraints). Our study focuses on attributable burden, using the TMREL as the counterfactual standard.

## **Normalizing IHD Mortality by Prevalence.**

The age-standardized mortality-to-prevalence ratio (MPR) for IHD estimates the proportion of individuals with the disease who die annually, adjusted for age. It serves as a proxy for population-level case fatality and highlights disparities that crude death rates may obscure. Recent studies have applied this metric to assess outcome gaps. A 2024 GBD-based analysis in Europe found that although men have higher IHD prevalence and absolute mortality, women exhibited a higher MPR, suggesting greater per-case mortality risk in women.^6^ This normalized measure, therefore, uncovers sex-based differences in survival not visible through raw rates alone.

Beyond sex differences, MPR is increasingly used to evaluate health system performance. Populations with similar IHD prevalence but higher MPRs likely face poorer care or delayed treatment. The GBD framework and Gates Foundation’s Goalkeepers report use MPRs for chronic diseases as indicators of care quality, assuming improved management should reduce mortality relative to prevalence.

This approach is not unique to IHD. In oncology, the mortality-to-incidence ratio (MIR) serves a similar role. A recent global analysis of 36 cancers across 185 countries showed that low- and middle-income countries had higher MPRs and MIRs, reflecting worse survival.^7^ Likewise, in multiple myeloma, a falling MPR, from 78% in 2008 to 22.8% in 2017, paralleled rising survival rates, illustrating how declining MPRs can reflect therapeutic progress.^8^ In summary, MPR offers a valuable lens to assess disease outcomes across populations and track improvements in care quality.

**Case Fatality Index (CFI) as Risk-Attributable Lethality Metric**

CFI is an extension of the MPR logic, adapted to GBD-modeled estimates of risk factor–attributable IHD mortality. In other words, it approximates the proportion of mortality attributable to a given risk factor (e.g., hypertension or poor diet) among those living with IHD. It allows for comparative assessment across sexes and countries, identifying where exposure to specific risk factors is associated with disproportionately high death rates. While not a traditional epidemiologic construct, CFI is conceptually aligned with the burden of risk approach used in comparative risk assessment. Its reproducibility stems from GBD’s open-access methodology and codebase (DisMod-MR 2.1), which ensures that results can be regenerated using publicly available inputs and assumptions.

All underlying data and modeling assumptions are openly accessible through the Institute for Health Metrics and Evaluation (IHME) and the Burden of Proof framework (https://ghdx.healthdata.org; https://github.com/ihmeuw-msca/burden-of-proof). Therefore, while CFI is a novel application, it is methodologically reproducible, publicly documented, and designed to facilitate cross-country and sex-stratified comparisons in the absence of more granular surveillance data.

**GBD Multiple Data Sources**

The GBD integrates multiple data sources, including vital registration systems, verbal autopsies, health surveys, censuses, hospital records, and disease registries. For countries with limited or incomplete vital registration data, the GBD employs statistical adjustments using covariate-driven predictive modeling, including spatiotemporal Gaussian process regression and Bayesian meta-regression (DisMod-MR 2.1). These methods account for differences in data availability and quality across countries by borrowing strength from regional patterns and using covariates such as sociodemographic index (SDI), education, urbanicity, and health system access to inform estimates. All input data and modeling assumptions are transparently documented in the GBD metadata repository (http://ghdx.healthdata.org/gbd-2021).

**Analytical Design and Temporal Scope**

This study was designed to assess statistically significant and absolute differences in sex-specific IHD mortality burden across EU countries at two benchmark years: 2011 and 2021. The 10-year interval was selected to capture meaningful epidemiological shifts, particularly in regions undergoing health system transitions or socioeconomic change. While the inclusion of additional time points might increase temporal resolution, it would also introduce greater uncertainty due to the modeled nature of the Global Burden of Disease (GBD) estimates. GBD outputs are generated using the Bayesian meta-regression framework DisMod-MR, which integrates heterogeneous data sources and applies smoothing and imputation where empirical data are sparse or inconsistent. In settings with limited or poor-quality surveillance, year-to-year fluctuations may reflect modeling assumptions rather than observed trends, limiting interpretability.

# **Statistical Analysis.**

## **1. Mortality-to-Prevalence Ratios (MPRs)**

To evaluate the burden of IHD mortality relative to disease prevalence, we calculated Mortality-to-Prevalence Ratios (MPRs) using age-standardized rates derived from the GBD 2021 study. The MPR was computed as:

**MPR = Age-Standardized Mortality Rate (ASMR) / Age-Standardized Prevalence Rate (ASPR)**

This ratio reflects population-level case fatality and enables comparison of relative IHD mortality burden across sexes and countries, adjusted for disease prevalence and demographic differences.

To estimate uncertainty intervals for the MPR, we applied the **extreme value method**, a conservative bounding technique appropriate when joint distributions of ASMR and ASPR are unavailable. This method estimates the 95% uncertainty interval (UI) as

*Lower* ***Bound* = Lower UI of ASMR / Upper UI of ASPR**

*Upper* ***Bound* = Upper UI of ASMR / Lower UI of ASP**

This approach yields cautious but transparent bounds consistent with standard practices in GBD-based analyses^11,12^.

For ease of interpretation, MPRs were also expressed as percentages by multiplying by 100. For example, an ASMR of 150 per 100,000 (95% UI: 130–170) and an ASPR of 2,000 per 100,000 (95% UI: 1,800–2,200) would yield an MPR of 7.5% (UI: 5.9%–9.4%)

## **2. Risk-Attributable Mortality Normalized by IHD Prevalence.**

To align with the case-based interpretation of MPRs, we also analyzed IHD mortality attributable to specific risk factors, normalized by IHD prevalence:

Normalized mortality = Case Fatality Index (CFI) $=\frac{Risk-attributable ASMR}{ASPR}$

Where:

*Risk-attributable ASMR* = IHD mortality due to a specific risk factor (per 100,000) stratified by sex and country.

*ASPR* = prevalence of IHD (per 100,000) stratified by sex and country.

This Case Fatality Index (CFI) approximates the mortality impact of individual risk factors among individuals with IHD, offering a disease-burden–adjusted lens on risk exposure by sex. Risk-attributable ASMRs were derived from GBD 2021 estimates for each EU country, stratified by sex and risk factor.

## 3. **Z-Test for Comparing Sex Ratios** **Across Populations**

To assess statistical differences in sex ratios (either MPRs or risk-specific CFIs) across populations (e.g., across countries), we performed a Z-test for two independent ratios. The Z-score was calculated using the following formula:

$$Z=\frac{(RR_{1}-RR_{2})}{\surd(S{E_{1}}^{2}+S{E_{2}}^{2})}$$

Where:

RR_1_ and RR_2_ are the ratio estimates (MPR or CFI) for women and men, respectively, and SE₁ and SE₂ are their standard errors. Given that the Global Burden of Disease (GBD) Study reports 95% uncertainty intervals (UIs) rather than standard errors, SEs were approximated using the width of the uncertainty interval, assuming symmetry:

$$SE = \frac{UI Upper-UILower}{1.96\times2}$$

A Z-score ≥ |1.96| was considered statistically significant at the 95% confidence level. A threshold of |2.58| was used to denote highly significant differences (p < 0.01).

In cases where GBD 2021 estimates included negative values within the 95% uncertainty intervals (UIs), these were considered statistical artifacts, as negative mortality rates are not biologically plausible. For such instances, we reported only the point estimate of the risk ratio (RR), flagged the result as unstable, and omitted confidence bounds. These estimates were interpreted qualitatively and excluded from formal significance testing.

This method is widely accepted in secondary analyses of GBD data where full distributional parameters are not publicly available. Although this approach may slightly overestimate variance, it offers a transparent and practical solution for comparative population-level risk analysis.

# **Ischemic heart disease (IHD) definition:**

For the GBD 2021 framework, IHD was modelled as the aggregate of discrete sequelae, including acute myocardial infarction and chronic ischemic heart disease.^13^

## **Case Definitions**

1. **Acute Myocardial Infarction (MI):** Defined according to the fourth universal definition of MI,^14^ this condition includes:
   - Clinical evidence of myocardial necrosis in a setting consistent with ischemia.
   - Detection of rising or falling cardiac biomarkers, accompanied by at least one of the following: symptoms of ischemia, new electrocardiographic changes (e.g., ST-segment/T-wave alterations), development of pathological Q waves, imaging evidence of new loss of viable myocardium or regional wall motion abnormalities, or the identification of an intracoronary thrombus.
   - Sudden unexplained cardiac death, involving cardiac arrest without evidence of a non-coronary cause.

Unstable angina was also included when reported separately as specified in the fourth universal definition.

1. **Chronic IHD:**
   - **Stable Angina:** Diagnosed based on clinical symptoms, physician assessment, or the use of nitrates for chest pain relief.
   - **Asymptomatic Ischemic Heart Disease:** Includes cases surviving 28 days post-MI but excludes ECG-based estimates due to their limited specificity and sensitivity.

These precise case definitions ensure consistency in modelling and robust estimation of IHD’s burden within the GBD framework, enabling targeted interventions at regional and global level.

# **Terminology**

As sex and gender often interact, we consistently used the term "sex" throughout the manuscript to reflect biological differences under investigation.

# **Transition Performance Index (TPI)**

The European Commission, Directorate-General for Research and Innovation evaluates economic development and structural transformation using the Transition Performance Index (TPI). This index captures a country's progress toward a sustainable and resilient prosperity model by combining metrics such as wealth, education, ICT (information, communication, and technology) use and skills, labor productivity, R&D intensity, and industrial base performance. A successful economic transition reflects balanced strength across these dimensions, which are also closely linked to health system capacity and equity.

In this study, we used the TPI, rather than relying on GNI per capita, as it offers a more nuanced and policy-relevant measure of socio-economic development. While GNI per capita reflects income level, it does not adequately capture structural factors that shape access to healthcare, innovation in public health infrastructure, or long-term investments in education and productivity, all of which contribute to differences in IHD outcomes.

According to the TPI 2021 report, one EU country (Ireland) is classified as a leader in economic transition, and eight others as strong performers, mostly due to their combined strengths in wealth and education. Six countries are good performers, primarily driven by strengths in ICT and skills. However, twelve EU countries are categorized as moderate or weak in their economic transition, with concerning deficits in wealth generation, labor productivity, R&D intensity, and industrial base. These disparities may influence country-level capacity to reduce cardiovascular disease burden equitably, especially across sex.

# **Appendix Figure 1. Trends in Age-Standardized Mortality Rates (ASMR) and Transition Performance Index (TPI) for IHD, Per 100,000 Inhabitants, Stratified by Country and Sex**

***Data derived from Global Burden of Diseases, year 2021***

***
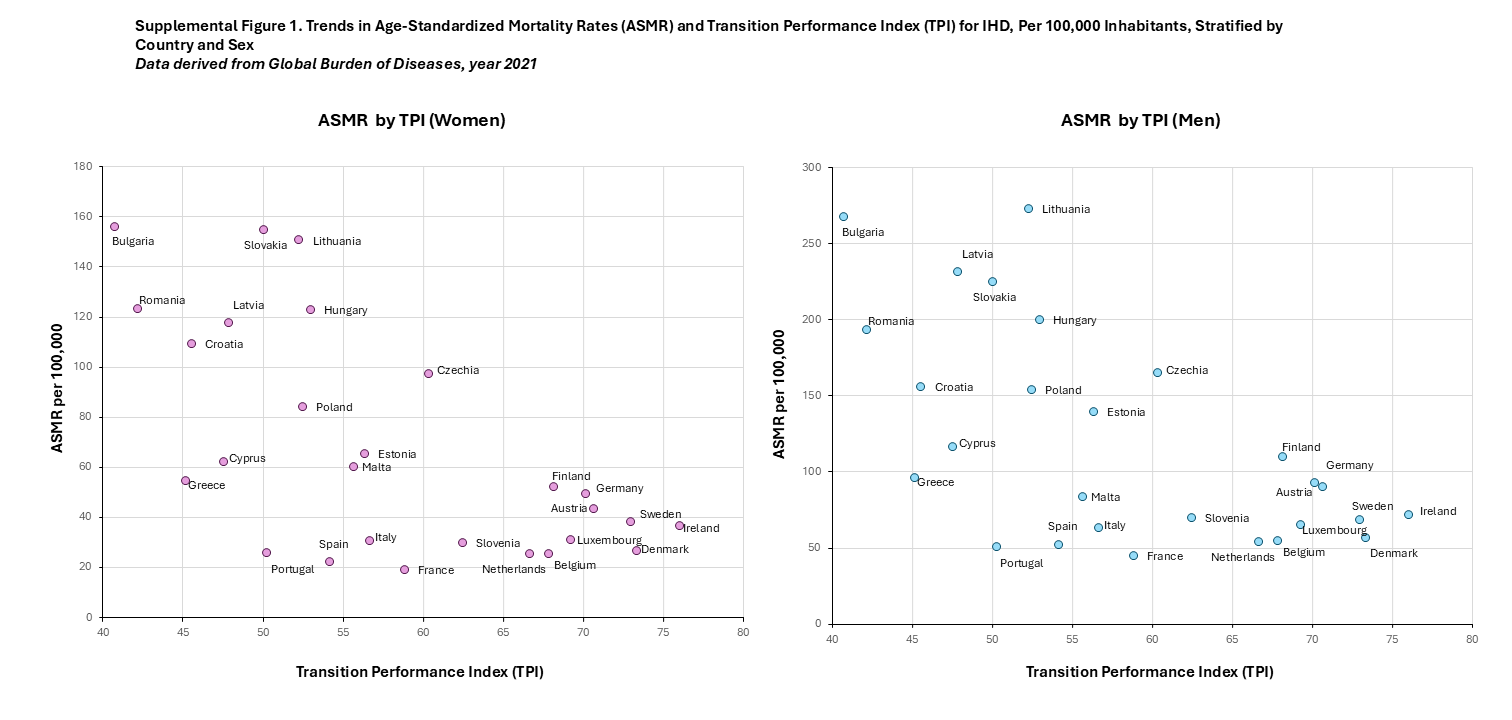
***

**Abbreviation:** ASMR=Age-standardized mortality rate

# **Appendix Figure 2: Correlation Between Age-Standardized Mortality Rates (ASMR) and Transition Performance Index (TPI) for Ischemic Heart Disease (IHD) in 2021. Regression line and 95%UI visualised.**

***Data derived from Global Burden of Diseases, year 2021***

**
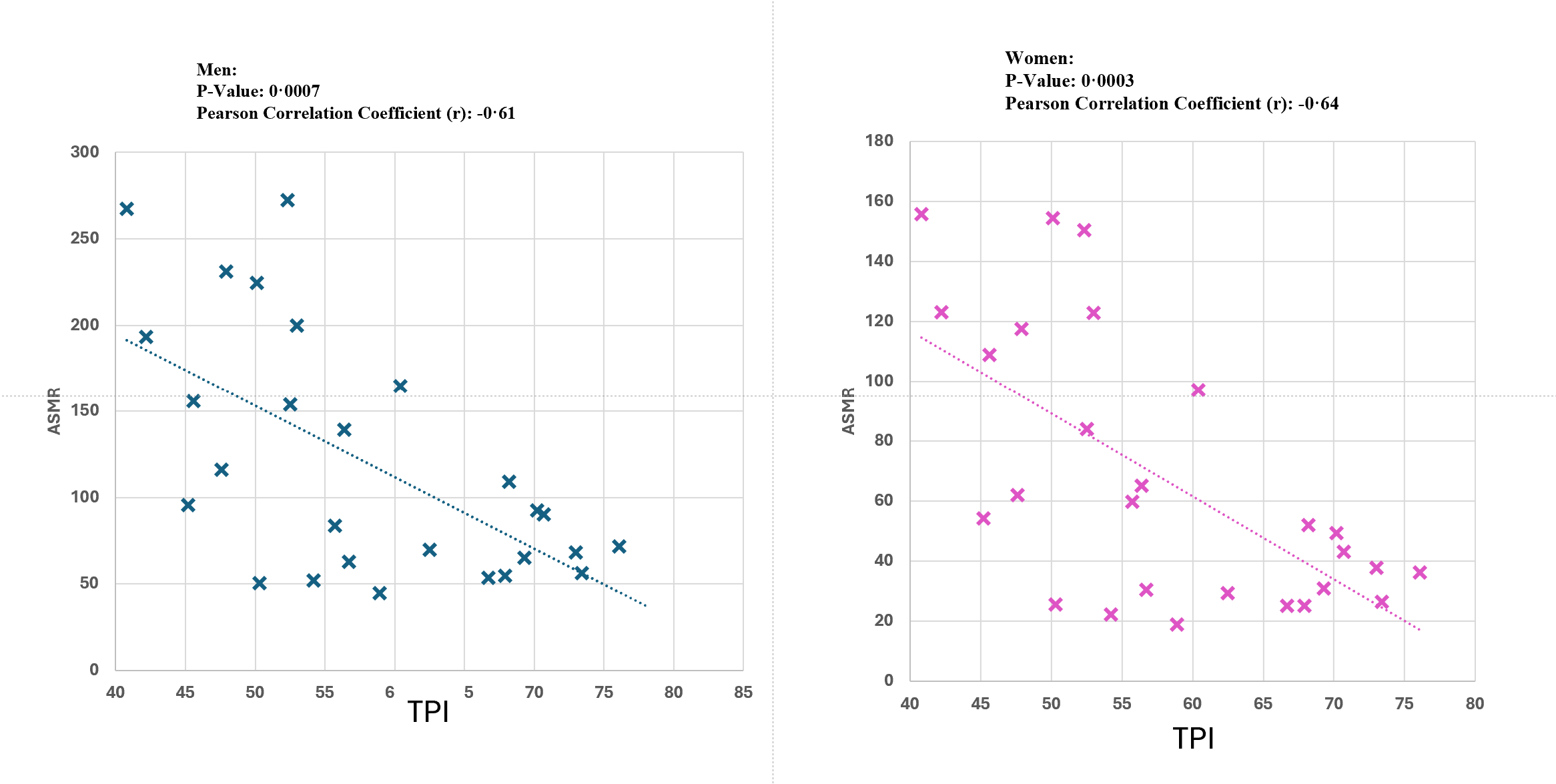
**

# **Appendix Figure 3. Trends in Age-Standardized Prevalence Rates for IHD, Per 100,000 Inhabitants, Stratified by Country and Sex**

***Data derived from Global Burden of Diseases, year 2021.***


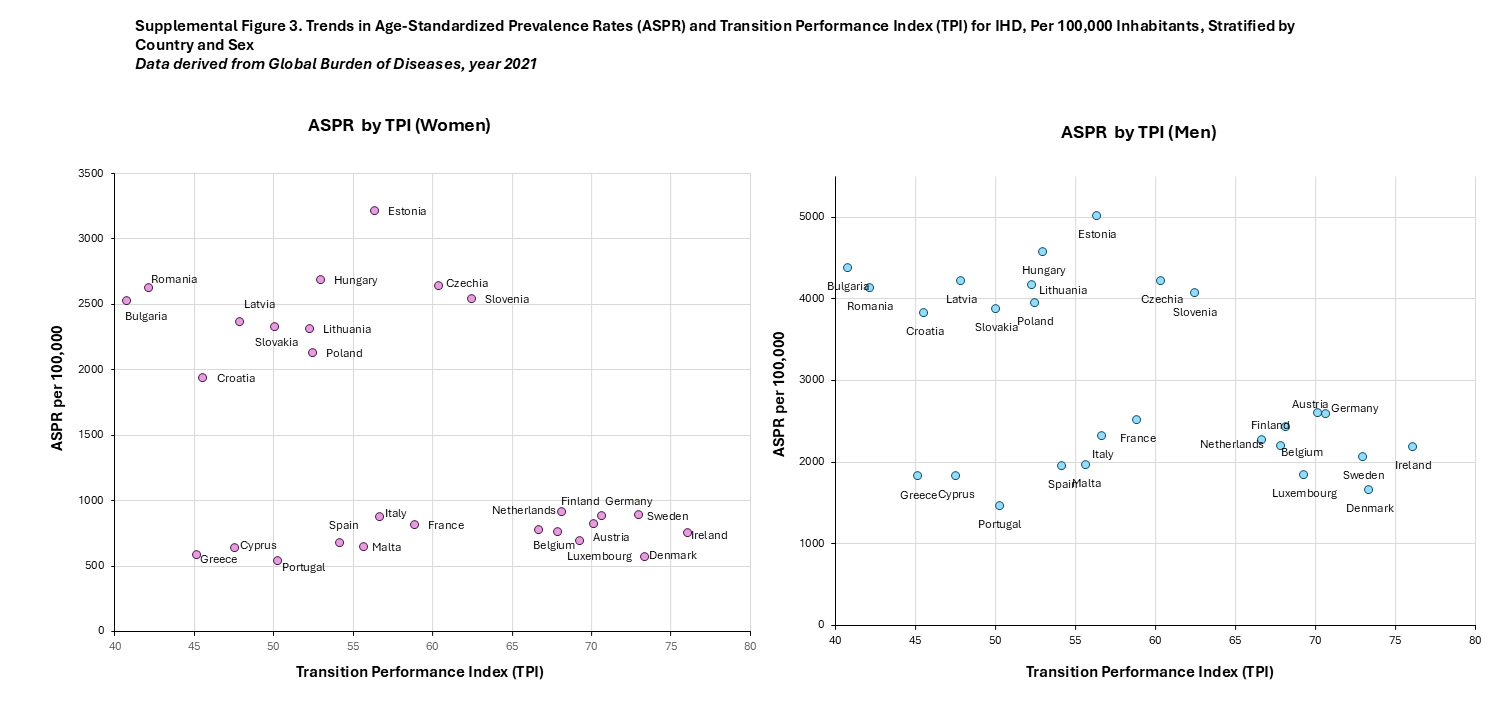


**Abbreviation:** ASPR=Age-standardized prevalence rate

# **Appendix Figure 4: Correlation Between Age-Standardized Prevalence Rates (ASMR) and Transition Performance Index (TPI) for Ischemic Heart Disease (IHD) in 2021**

***Data derived from Global Burden of Diseases, year 2021***


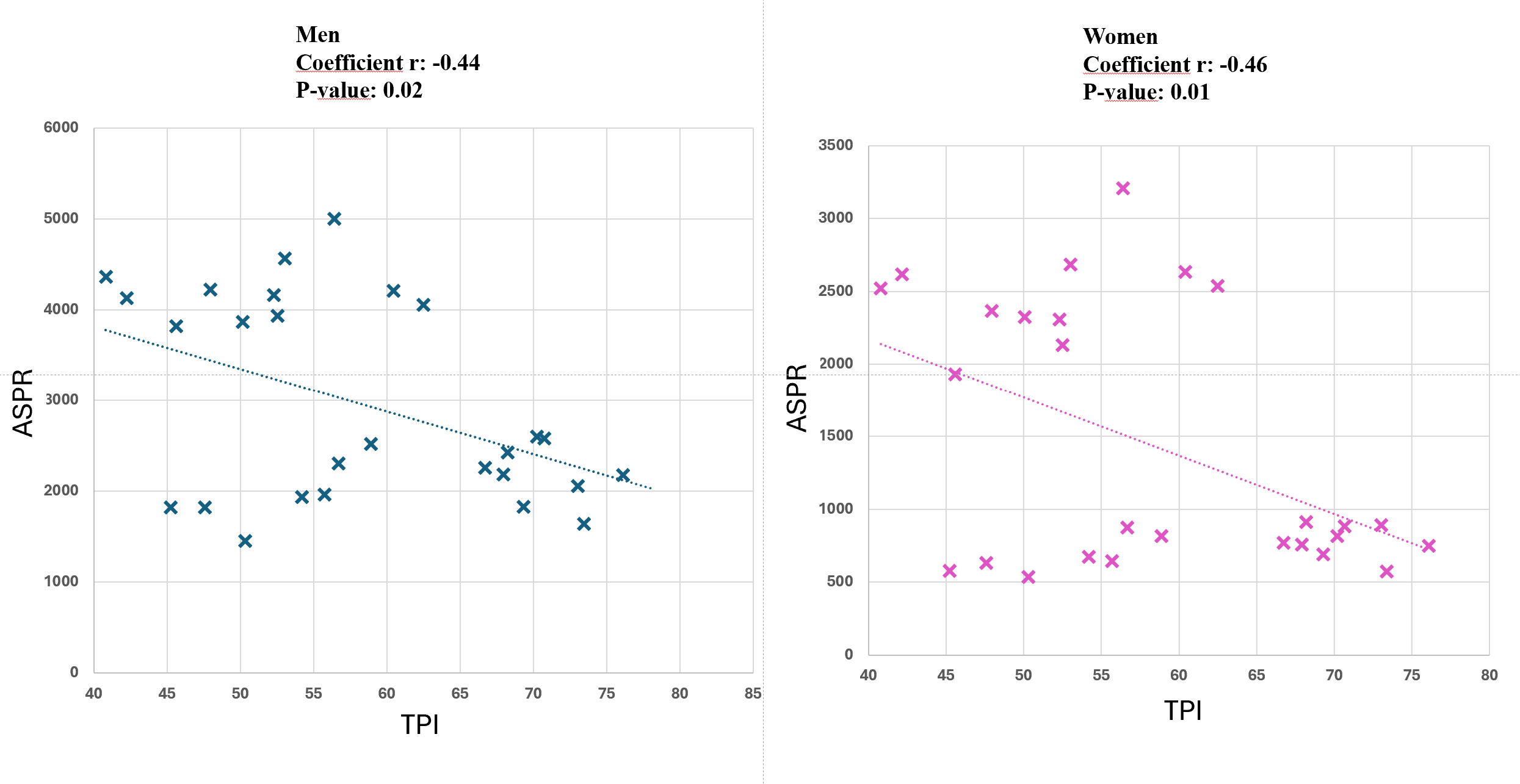


# **Appendix Figure 5: Correlation Between Age-Standardized Mortality Rates (ASMR) and Age-Standardized Prevalence Rates for Ischemic Heart Disease (IHD) in 2021, in women and in men.**

**. *Data derived from Global Burden of Diseases, year 2021***


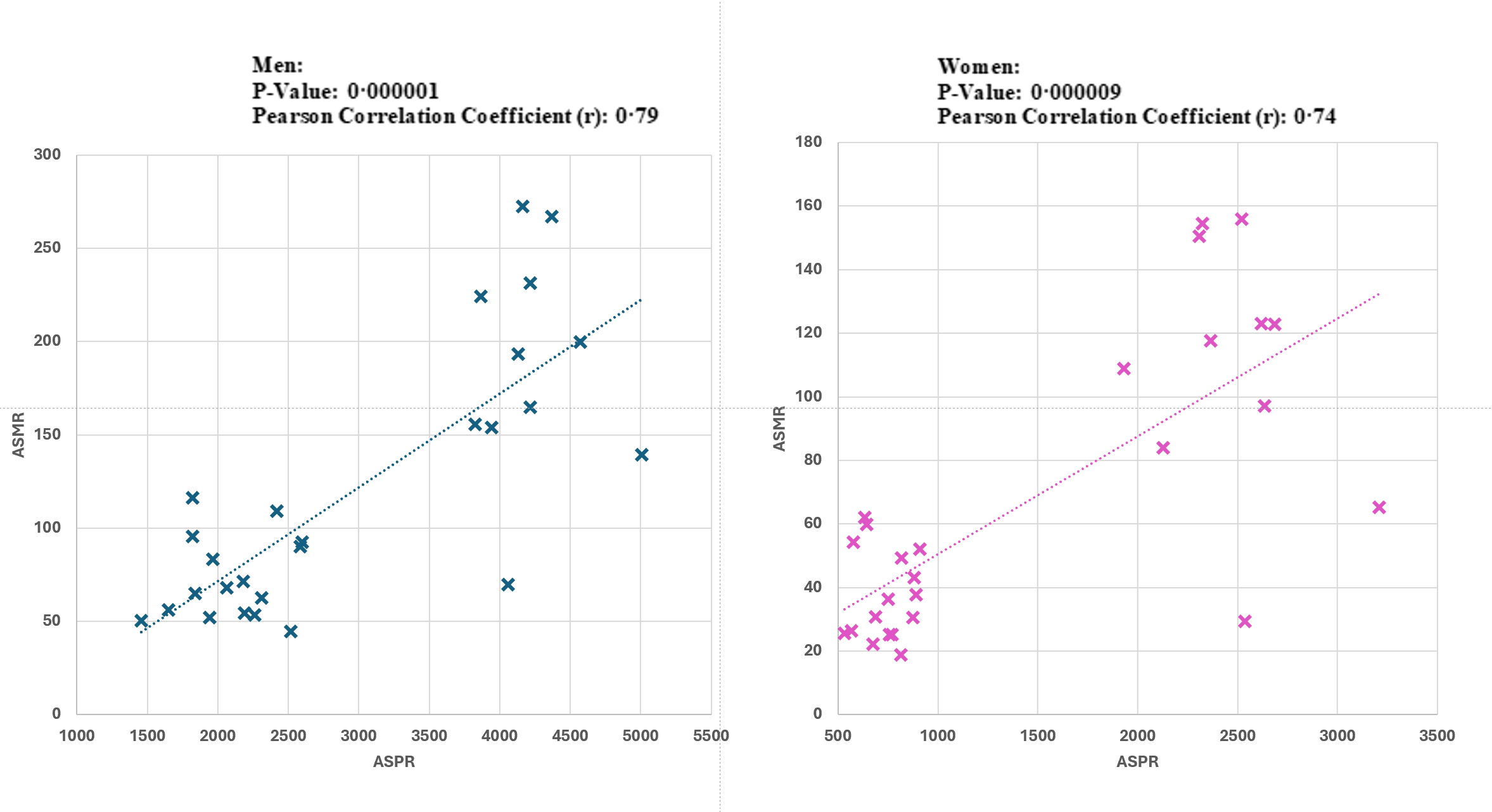


Abbreviation: ASMR= Age-standardized mortality rate; ASPR: Age-standardized prevalence rate

# **Appendix Figure 6. Correlation Between Mortality-Prevalence ratios (MPR) for Ischemic Heart Disease (IHD) and Transition Performance Index in 2021, in women and in men.**

***Data derived from Global Burden of Diseases, year 2021***


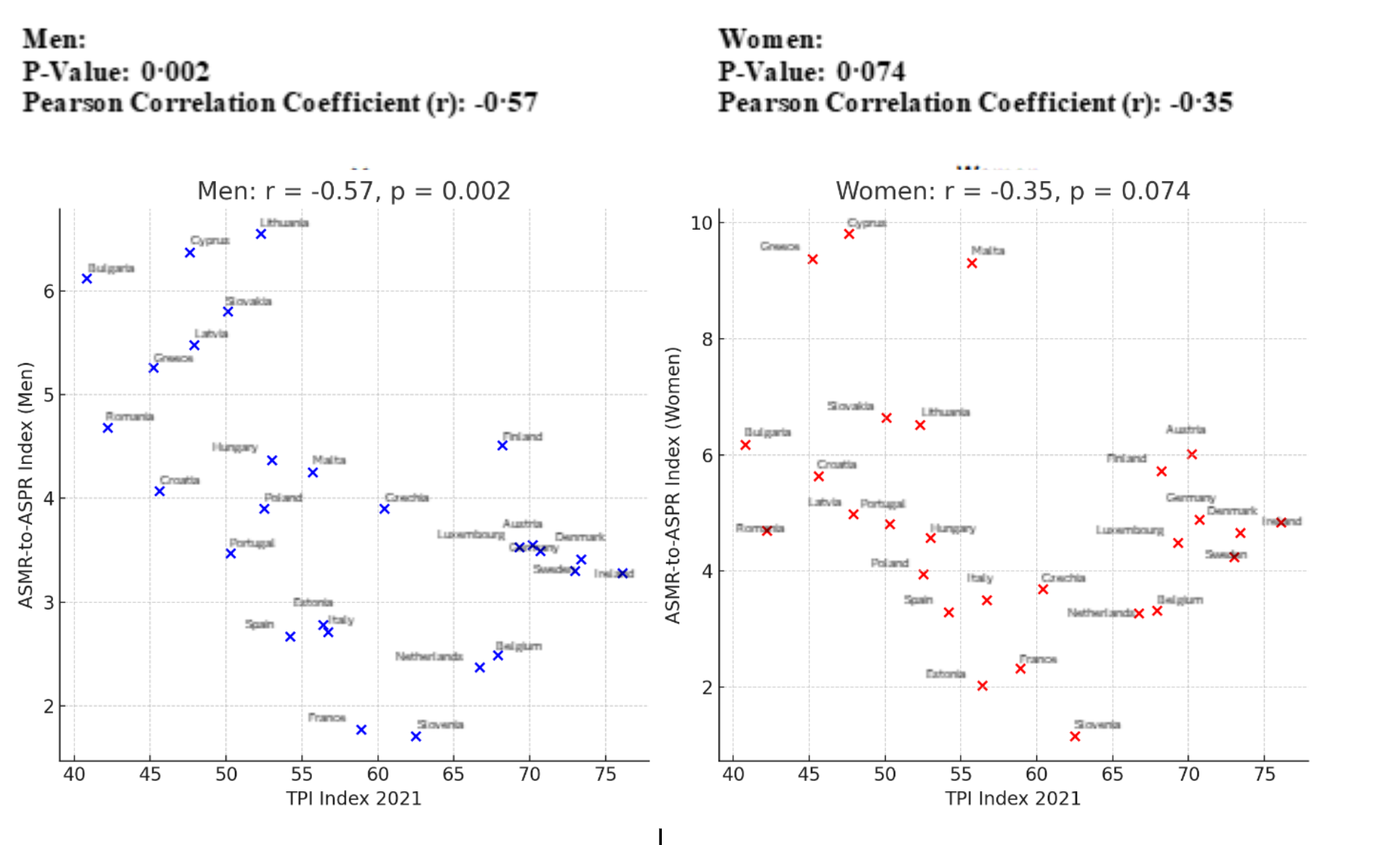


**Abbreviations:** MPR= Mortality-Prevalence ratio; TPI= transition performance index

# **Appendix Table 1: Economic Transition Index 2021 by Country**

| **Country** | **2021 Score** | **Category** |
| --- | --- | --- |
| Ireland | 76.1 | Transition leader |
| Denmark | 73.4 | Strong transition |
| Sweden | 73 | Strong transition |
| Germany | 70.7 | Strong transition |
| Austria | 70.2 | Strong transition |
| Luxembourg | 69.3 | Strong transition |
| Finland | 68.2 | Strong transition |
| Belgium | 67.9 | Strong transition |
| Netherlands | 66.7 | Strong transition |
| Slovenia | 62.5 | Good transition |
| Czechia | 60.4 | Good transition |
| France | 58.9 | Good transition |
| Italy | 56.7 | Good transition |
| Estonia | 56.4 | Good transition |
| Malta | 55.7 | Good transition |
| Spain | 54.2 | Moderate transition |
| Hungary | 53 | Moderate transition |
| Poland | 52.5 | Moderate transition |
| Lithuania | 52.3 | Moderate transition |
| Portugal | 50.3 | Moderate transition |
| Slovakia | 50.1 | Moderate transition |
| Latvia | 47.9 | Moderate transition |
| Cyprus | 47.6 | Moderate transition |
| Croatia | 45.6 | Moderate transition |
| Greece | 45.2 | Moderate transition |
| Romania | 42.2 | Weak transition |
| Bulgaria | 40.8 | Weak transition |

*GDP per capita is based on the de facto definition of population, which counts all residents regardless of legal status or citizenship. The values reported are midyear estimates. These data were complemented by the economic classifications from the Bureau of Economic Analysis (BEA) and the United States Census Bureau (USCB). The BEA provided gross domestic product (GDP) per capita by state.

The World Bank classification from 2022 was used as it is the closest available to the 2021 data and served as a relevant baseline for the analysis.

| **Appendix Table 2: Dietary risk factor exposure GBD definitions and optimal level of exposure as defined by GBD 2021.** | | |
| --- | --- | --- |
| **Dietary Risk Factor** | **Definition of Exposure** | **Optimal level or range of intake** |
| **Diet low in fruit** | Average daily consumption (in grams per day) of fruit including fresh, frozen, cooked, canned, or dried fruit, excluding fruit juices and salted or pickled fruits | 340–350 g/day |
| **Diet low in vegetables** | Average daily consumption (in grams per day) of vegetables, including fresh, frozen, cooked, canned, or dried vegetables and excluding legumes and salted or pickled vegetables, juices, nuts and seeds, and starchy vegetables such as potatoes or corn | 306–372 g/day |
| **Diet low in whole grains** | Average daily consumption (in grams per  day) of whole grains (bran, germ, and endosperm in their natural proportion) from breakfast cereals, bread, rice, pasta, biscuits, muffins, tortillas, pancakes, and other sources | 160–210 g/day |
| **Diet low in nuts and seeds** | Average daily consumption (in grams per day) of nuts and seeds, including tree nuts and seeds and peanuts | 19–24 g/day |
| **Diet low in fibre** | Average daily consumption (in grams per day) of fibre from all sources including fruits, vegetables, grains, legumes, and pulses | 22–25 g/day |
| **Diet low in seafood omega-3 fatty acids** | Average daily consumption (in milligrams per day) of eicosapentaenoic acid (EPA) and docosahexaenoic acid (DHA) | 470–660 mg/day |
| **Diet low in omega- 6 polyunsaturated fatty acids** | Average daily consumption (in % daily energy) from omega-6 polyunsaturated fatty acids (PUFA) (specifically linoleic acid, γ- linolenic acid, eicosadienoic acid, dihomo-γ- linolenic acid, arachidonic acid) | 9–10% of total daily energy |
| **Diet low in legumes** | Average daily consumption (in grams per day) of legumes and pulses, including fresh, frozen, cooked, canned, or dried legumes | 100–110 g/day |
| **Diet high in red meat** | Average daily consumption (in grams per day) of unprocessed red meat including pork and bovine meats such as beef, pork, lamb, and goat, but excluding all processed meats, poultry, fish, and eggs | 0–200 g/day |
| **Diet high in processed meat** | Average daily consumption (in grams per day) of meat preserved by smoking, curing, salting, or addition of chemical preservatives | 0 g/day |
| **Diet high in sugar- sweetened beverages (SSBs)** | Average daily consumption (in grams per day) of beverages with ≥50 kcal per 226.8 gram serving, including carbonated beverages, sodas, energy drinks, and fruit drinks, but excluding | 0 g/day |
| **Diet high in trans fatty acids** | Average daily consumption (in percent daily energy) of trans fat from all sources, mainly partially hydrogenated vegetable oils and ruminant products | 0–1.1% of total daily energy |
| **Diet high in sodium** | Average 24-hour urinary sodium excretion (in grams per day) | 1–5 g/day |
| Note that GBD dietary risk factor modelling is for adult populations only (ages 25+). | | |
|  | | |

| **Appendix Table 3. Sex-stratified distribution of Z scores based on MPR for IHD across European Countries (Years 2011 vs 2021).** | | | | | | |
| --- | --- | --- | --- | --- | --- | --- |
| **Country** | **2011** | | **2021** | | **Z score 2011** | **Z score 2021** |
|  | **SE 2011 men** | **SE 2011 women** | **SE 2021 men** | **SE 2021 women** |  |  |
| **Austria** | 0.41 | 1.00 | 0.36 | 0.81 | 3.58 | 2.78 |
| **Belgium** | 0.32 | 0.66 | 0.27 | 0.48 | 2.11 | 1.49 |
| **Bulgaria** | 0.52 | 0.60 | 0.76 | 0.74 | 0.78 | 0.06 |
| **Croatia** | 0.50 | 0.88 | 0.49 | 0.73 | 2.37 | 1.79 |
| **Cyprus** | 1.35 | 2.78 | 0.91 | 1.60 | 2.08 | 1.87 |
| **Czechia** | 0.41 | 0.57 | 0.45 | 0.46 | 0.83 | -0.34 |
| **Denmark** | 0.52 | 0.90 | 0.38 | 0.66 | 2.04 | 1.65 |
| **Estonia** | 0.39 | 0.44 | 0.33 | 0.26 | -2.03 | -1.80 |
| **Finland** | 0.69 | 1.21 | 0.51 | 0.85 | 1.23 | 1.22 |
| **France** | 0.19 | 0.38 | 0.19 | 0.32 | 2.09 | 1.47 |
| **Germany** | 0.28 | 0.72 | 0.35 | 0.69 | 3.43 | 1.81 |
| **Greece** | 0.55 | 1.91 | 0.50 | 1.32 | 4.67 | 2.92 |
| **Hungary** | 0.36 | 0.49 | 0.46 | 0.52 | 0.78 | 0.29 |
| **Ireland** | 0.45 | 1.02 | 0.38 | 0.73 | 2.59 | 1.89 |
| **Italy** | 0.37 | 0.71 | 0.35 | 0.60 | 1.58 | 1.13 |
| **Latvia** | 0.60 | 0.92 | 0.63 | 0.65 | 0.29 | -0.56 |
| **Lithuania** | 0.55 | 0.97 | 0.69 | 0.78 | 0.94 | -0.03 |
| **Luxembourg** | 0.57 | 0.97 | 0.42 | 0.67 | 1.29 | 1.21 |
| **Malta** | 0.77 | 2.01 | 0.53 | 1.42 | 3.29 | 3.33 |
| **Netherlands** | 0.26 | 0.49 | 0.26 | 0.45 | 2.10 | 1.73 |
| **Poland** | 0.43 | 0.55 | 0.52 | 0.59 | -0.04 | 0.06 |
| **Portugal** | 0.42 | 1.00 | 0.38 | 0.84 | 2.82 | 1.45 |
| **Romania** | 0.46 | 0.60 | 0.53 | 0.51 | 1.26 | 0.03 |
| **Slovakia** | 0.66 | 0.95 | 0.69 | 0.86 | 1.40 | 0.76 |
| **Slovenia** | 0.27 | 0.29 | 0.22 | 0.18 | -0.96 | -1.96 |
| **Spain** | 0.27 | 0.57 | 0.26 | 0.50 | 1.87 | 1.09 |
| **Sweden** | 0.61 | 0.96 | 0.52 | 0.72 | 1.06 | 1.06 |
| **European Union** | 0.31 | 0.59 | 0.30 | 0.50 | 2.10 | 1.29 |
| **Abbreviation:** IHD= ischemic heart disease; MPR= Mortality-Prevalence ratio; SE=standard error | | | | | | |

| **Appendix Table 4. Age-standardised mortality rates and case fatality indexes for IHD attributable to High LDL cholesterol. 2011 , 2021** | | | | | | | | | | |
| --- | --- | --- | --- | --- | --- | --- | --- | --- | --- | --- |
| **Country** | **2011** | | | | | **2021** | | | | |
|  | **Men** | | **Women** | | **CFI Ratio** | **Men** | | **Women** |  | **CFI Ratio** |
|  | **Mortality rate** | **CFI** | **Mortality rate** | **CFI** |  | **Mortality rate** | **CFI** | **Mortality rate** | **CFI** |  |
| **Austria** | 43.48 | 1.67 | 22.46 | 2.74 | 1.64 | 31.70 | 1.22 | 15.50 | 1.89 | 1.55 |
| Lower bound 95%CI | 28.82 | 1.01 | 13.87 | 1.53 |  | 21.28 | 0.73 | 9.55 | 1.04 |  |
| Upper bound 95%CI | 58.65 | 2.51 | 31.63 | 4.28 |  | 42.62 | 1.85 | 21.77 | 3.03 |  |
| **Belgium** | 25.78 | 1.18 | 12.22 | 1.63 | 1.38 | 17.17 | 0.78 | 7.79 | 1.03 | 1.31 |
| Lower bound 95%CI | 17.17 | 0.71 | 7.72 | 0.92 |  | 11.46 | 0.46 | 4.92 | 0.57 |  |
| Upper bound 95%CI | 35.49 | 1.80 | 17.08 | 2.54 |  | 23.31 | 1.22 | 10.84 | 1.64 |  |
| **Bulgaria** | 104.43 | 2.28 | 53.73 | 2.09 | 0.92 | 93.38 | 2.14 | 46.29 | 1.84 | 0.86 |
| Lower bound 95%CI | 70.84 | 1.41 | 34.09 | 1.21 |  | 61.45 | 1.27 | 29.55 | 1.04 |  |
| Upper bound 95%CI | 137.14 | 3.32 | 73.90 | 3.16 |  | 124.43 | 3.22 | 64.31 | 2.86 |  |
| **Croatia** | 61.48 | 1.67 | 40.23 | 2.34 | 1.40 | 47.75 | 1.25 | 32.25 | 1.67 | 1.34 |
| Lower bound 95%CI | 40.70 | 0.97 | 24.86 | 1.26 |  | 30.45 | 0.70 | 19.30 | 0.87 |  |
| Upper bound 95%CI | 82.95 | 2.56 | 56.43 | 3.72 |  | 65.51 | 1.97 | 45.63 | 2.69 |  |
| **Cyprus** | 56.15 | 3.61 | 25.45 | 5.15 | 1.43 | 39.21 | 2.15 | 18.41 | 2.91 | 1.35 |
| Lower bound 95%CI | 36.51 | 2.05 | 15.54 | 2.63 |  | 25.77 | 1.23 | 10.99 | 1.48 |  |
| Upper bound 95%CI | 77.23 | 5.70 | 37.43 | 8.94 |  | 54.61 | 3.49 | 27.62 | 5.13 |  |
| **Czechia** | 77.11 | 1.85 | 42.25 | 1.83 | 0.99 | 55.85 | 1.32 | 29.62 | 1.12 | 0.85 |
| Lower bound 95%CI | 51.75 | 1.13 | 26.37 | 1.03 |  | 35.90 | 0.76 | 18.45 | 0.63 |  |
| Upper bound 95%CI | 102.94 | 2.74 | 58.81 | 2.81 |  | 76.20 | 2.04 | 41.57 | 1.78 |  |
| **Denmark** | 28.21 | 1.81 | 13.99 | 2.40 | 1.32 | 19.43 | 1.18 | 8.61 | 1.51 | 1.29 |
| Lower bound 95%CI | 19.04 | 1.08 | 8.98 | 1.36 |  | 13.00 | 0.69 | 5.50 | 0.84 |  |
| Upper bound 95%CI | 37.22 | 2.69 | 19.32 | 3.73 |  | 26.19 | 1.82 | 11.99 | 2.42 |  |
| **Estonia** | 94.86 | 2.01 | 41.93 | 1.39 | 0.69 | 50.05 | 1.00 | 20.64 | 0.64 | 0.64 |
| Lower bound 95%CI | 63.72 | 1.24 | 26.02 | 0.78 |  | 32.85 | 0.59 | 12.56 | 0.35 |  |
| Upper bound 95%CI | 126.76 | 2.92 | 58.61 | 2.12 |  | 67.08 | 1.51 | 29.70 | 1.03 |  |
| **Finland** | 52.23 | 2.51 | 21.90 | 2.65 | 1.06 | 36.29 | 1.50 | 14.98 | 1.65 | 1.10 |
| Lower bound 95%CI | 35.25 | 1.52 | 13.60 | 1.45 |  | 24.07 | 0.88 | 9.09 | 0.88 |  |
| Upper bound 95%CI | 70.49 | 3.77 | 31.09 | 4.21 |  | 49.32 | 2.33 | 21.39 | 2.71 |  |
| **France** | 21.93 | 0.84 | 8.39 | 1.02 | 1.21 | 15.83 | 0.63 | 5.91 | 0.73 | 1.15 |
| Lower bound 95%CI | 14.85 | 0.52 | 5.23 | 0.57 |  | 10.59 | 0.37 | 3.69 | 0.40 |  |
| Upper bound 95%CI | 29.37 | 1.23 | 11.78 | 1.58 |  | 21.19 | 0.94 | 8.34 | 1.15 |  |
| **Germany** | 39.50 | 1.30 | 19.50 | 2.02 | 1.56 | 31.33 | 1.21 | 13.76 | 1.56 | 1.29 |
| Lower bound 95%CI | 26.71 | 0.80 | 12.14 | 1.14 |  | 21.20 | 0.73 | 8.54 | 0.84 |  |
| Upper bound 95%CI | 53.66 | 1.92 | 27.24 | 3.08 |  | 42.25 | 1.84 | 19.23 | 2.47 |  |
| **Greece** | 44.97 | 2.24 | 26.52 | 4.54 | 2.02 | 33.78 | 1.86 | 16.05 | 2.78 | 1.50 |
| Lower bound 95%CI | 31.11 | 1.39 | 16.47 | 2.45 |  | 23.31 | 1.13 | 10.28 | 1.50 |  |
| Upper bound 95%CI | 59.32 | 3.29 | 37.30 | 7.30 |  | 44.73 | 2.79 | 22.14 | 4.48 |  |
| **Hungary** | 95.48 | 1.95 | 51.22 | 1.84 | 0.95 | 73.61 | 1.61 | 39.53 | 1.47 | 0.91 |
| Lower bound 95%CI | 65.15 | 1.21 | 32.63 | 1.07 |  | 48.38 | 0.95 | 25.64 | 0.85 |  |
| Upper bound 95%CI | 126.32 | 2.82 | 70.60 | 2.79 |  | 97.58 | 2.38 | 54.65 | 2.26 |  |
| **Ireland** | 35.26 | 1.61 | 16.73 | 2.27 | 1.41 | 21.67 | 0.99 | 10.11 | 1.35 | 1.35 |
| Lower bound 95%CI | 23.62 | 0.97 | 10.73 | 1.30 |  | 14.39 | 0.58 | 6.18 | 0.72 |  |
| Upper bound 95%CI | 47.62 | 2.40 | 23.38 | 3.58 |  | 30.04 | 1.58 | 14.61 | 2.23 |  |
| **Italy** | 26.50 | 1.08 | 12.65 | 1.40 | 1.29 | 19.68 | 0.85 | 9.00 | 1.03 | 1.21 |
| Lower bound 95%CI | 17.62 | 0.63 | 7.76 | 0.74 |  | 13.16 | 0.48 | 5.42 | 0.52 |  |
| Upper bound 95%CI | 36.05 | 1.70 | 18.14 | 2.30 |  | 26.94 | 1.40 | 12.86 | 1.75 |  |
| **Latvia** | 121.33 | 3.04 | 57.97 | 2.87 | 0.94 | 80.57 | 1.91 | 37.08 | 1.57 | 0.82 |
| Lower bound 95%CI | 83.00 | 1.90 | 37.56 | 1.65 |  | 53.75 | 1.14 | 23.27 | 0.86 |  |
| Upper bound 95%CI | 160.80 | 4.41 | 79.03 | 4.43 |  | 108.71 | 2.90 | 51.79 | 2.49 |  |
| **Lithuania** | 139.79 | 3.41 | 67.43 | 3.35 | 0.98 | 98.28 | 2.36 | 48.04 | 2.08 | 0.88 |
| Lower bound 95%CI | 95.37 | 2.15 | 43.10 | 1.92 |  | 65.29 | 1.41 | 29.63 | 1.14 |  |
| Upper bound 95%CI | 186.06 | 4.86 | 92.96 | 5.13 |  | 132.85 | 3.58 | 67.96 | 3.31 |  |
| **Luxembourg** | 29.74 | 1.89 | 13.47 | 2.20 | 1.17 | 21.11 | 1.15 | 9.35 | 1.36 | 1.18 |
| Lower bound 95%CI | 19.62 | 1.10 | 8.40 | 1.16 |  | 13.71 | 0.66 | 5.73 | 0.72 |  |
| Upper bound 95%CI | 40.87 | 2.94 | 19.02 | 3.66 |  | 28.83 | 1.80 | 13.32 | 2.28 |  |
| **Malta** | 48.10 | 2.60 | 27.27 | 4.65 | 1.79 | 29.31 | 1.49 | 19.32 | 3.01 | 2.01 |
| Lower bound 95%CI | 32.36 | 1.54 | 17.34 | 2.54 |  | 19.97 | 0.89 | 12.46 | 1.69 |  |
| Upper bound 95%CI | 64.37 | 3.93 | 38.54 | 7.64 |  | 39.28 | 2.32 | 27.21 | 4.96 |  |
| **Netherlands** | 23.44 | 0.99 | 11.14 | 1.37 | 1.38 | 17.30 | 0.77 | 8.18 | 1.06 | 1.39 |
| Lower bound 95%CI | 15.61 | 0.59 | 7.11 | 0.78 |  | 11.26 | 0.44 | 5.10 | 0.58 |  |
| Upper bound 95%CI | 31.69 | 1.48 | 15.40 | 2.12 |  | 23.88 | 1.21 | 11.47 | 1.70 |  |
| **Poland** | 60.03 | 1.52 | 29.06 | 1.36 | 0.90 | 47.79 | 1.21 | 23.66 | 1.11 | 0.92 |
| Lower bound 95%CI | 40.47 | 0.90 | 18.37 | 0.75 |  | 31.08 | 0.67 | 15.19 | 0.60 |  |
| Upper bound 95%CI | 80.24 | 2.34 | 40.47 | 2.19 |  | 65.39 | 1.95 | 34.33 | 1.92 |  |
| **Portugal** | 20.93 | 1.43 | 10.56 | 2.17 | 1.52 | 18.76 | 1.29 | 8.08 | 1.52 | 1.18 |
| Lower bound 95%CI | 14.40 | 0.86 | 6.66 | 1.17 |  | 13.14 | 0.78 | 5.20 | 0.80 |  |
| Upper bound 95%CI | 27.76 | 2.18 | 14.82 | 3.56 |  | 24.48 | 1.96 | 11.27 | 2.60 |  |
| **Romania** | 79.69 | 2.06 | 47.19 | 2.14 | 1.04 | 68.33 | 1.65 | 39.06 | 1.49 | 0.90 |
| Lower bound 95%CI | 53.88 | 1.24 | 30.00 | 1.23 |  | 45.75 | 0.99 | 24.68 | 0.85 |  |
| Upper bound 95%CI | 106.17 | 3.07 | 65.36 | 3.28 |  | 92.36 | 2.54 | 54.97 | 2.34 |  |
| **Slovakia** | 95.24 | 2.57 | 55.04 | 2.78 | 1.08 | 73.23 | 1.89 | 45.64 | 1.96 | 1.04 |
| Lower bound 95%CI | 63.52 | 1.53 | 34.52 | 1.56 |  | 48.30 | 1.12 | 28.22 | 1.08 |  |
| Upper bound 95%CI | 128.90 | 3.87 | 77.33 | 4.34 |  | 101.70 | 2.99 | 64.55 | 3.11 |  |
| **Slovenia** | 30.31 | 0.88 | 13.44 | 0.68 | 0.77 | 22.47 | 0.55 | 8.57 | 0.34 | 0.61 |
| Lower bound 95%CI | 20.24 | 0.52 | 8.27 | 0.37 |  | 14.45 | 0.32 | 5.11 | 0.18 |  |
| Upper bound 95%CI | 41.16 | 1.35 | 19.19 | 1.10 |  | 30.78 | 0.87 | 12.43 | 0.56 |  |
| **Spain** | 22.08 | 1.05 | 9.34 | 1.29 | 1.23 | 17.06 | 0.88 | 6.63 | 0.98 | 1.12 |
| Lower bound 95%CI | 14.97 | 0.65 | 5.78 | 0.71 |  | 11.59 | 0.54 | 4.14 | 0.54 |  |
| Upper bound 95%CI | 29.45 | 1.54 | 13.24 | 2.05 |  | 22.81 | 1.33 | 9.33 | 1.57 |  |
| **Sweden** | 37.25 | 1.92 | 18.81 | 2.15 | 1.12 | 22.42 | 1.09 | 11.59 | 1.30 | 1.20 |
| Lower bound 95%CI | 24.83 | 1.11 | 11.51 | 1.14 |  | 14.34 | 0.59 | 7.10 | 0.68 |  |
| Upper bound 95%CI | 50.58 | 3.00 | 26.52 | 3.51 |  | 30.95 | 1.77 | 16.44 | 2.17 |  |
| **European Union** | 39.36 | 1.40 | 19.56 | 1.70 | 1.21 | 29.84 | 1.12 | 14.37 | 1.24 | 1.11 |
| Lower bound 95%CI | 26.85 | 0.87 | 12.37 | 0.96 |  | 20.19 | 0.67 | 9.08 | 0.69 |  |
| Upper bound 95%CI | 52.73 | 2.08 | 27.13 | 2.63 |  | 40.05 | 1.70 | 20.01 | 1.96 |  |
|  | | | | | | | | | | |
| Data and definitions from Global Burden of Disease Database. 2021 | | | | | | | | | | |
| *Each value corresponds to mortality rates per 100.000 inhabitants (95% Uncertainty Interval) | | | | | | | | | | |
| Abbreviations: CFI, case-fatality index | | | | | | | | | | |

| **Appendix Table 5. Age-standardised mortality rates and case fatality indexes for IHD attributable to High systolic blood pressure.2011 , 2021** | | | | | | | | | | |
| --- | --- | --- | --- | --- | --- | --- | --- | --- | --- | --- |
| **Country** | **2011** | | | | | **2021** | | | | |
|  | **Men** | | **Women** | | **CFI Ratio** | **Men** | | **Women** | | **CFI Ratio** |
|  | **Mortality rate** | **CFI** | **Mortality rate** | **CFI** |  | **Mortality rate** | **CFI** | **Mortality rate** | **CFI** |  |
| **Austria** | 68.21 | 2.62 | 39.16 | 4.77 | 1.82 | 49.88 | 1.92 | 26.97 | 3.29 | 1.72 |
| Lower bound 95%CI | 52.13 | 1.83 | 28.30 | 3.12 |  | 37.73 | 1.29 | 18.92 | 2.05 |  |
| Upper bound 95%CI | 82.44 | 3.53 | 49.27 | 6.66 |  | 60.29 | 2.61 | 34.20 | 4.76 |  |
| **Belgium** | 44.64 | 2.05 | 23.30 | 3.10 | 1.52 | 29.89 | 1.36 | 14.77 | 1.95 | 1.43 |
| Lower bound 95%CI | 36.08 | 1.49 | 17.93 | 2.14 |  | 23.73 | 0.95 | 11.06 | 1.28 |  |
| Upper bound 95%CI | 52.53 | 2.66 | 27.82 | 4.14 |  | 35.77 | 1.87 | 18.04 | 2.74 |  |
| **Bulgaria** | 172.27 | 3.76 | 108.06 | 4.20 | 1.12 | 150.94 | 3.46 | 89.80 | 3.56 | 1.03 |
| Lower bound 95%CI | 136.66 | 2.71 | 82.79 | 2.95 |  | 119.17 | 2.46 | 68.27 | 2.41 |  |
| Upper bound 95%CI | 202.47 | 4.90 | 130.43 | 5.58 |  | 181.86 | 4.71 | 110.76 | 4.93 |  |
| **Croatia** | 117.60 | 3.19 | 81.28 | 4.72 | 1.48 | 90.05 | 2.35 | 64.04 | 3.31 | 1.41 |
| Lower bound 95%CI | 93.65 | 2.22 | 61.44 | 3.12 |  | 68.43 | 1.57 | 48.31 | 2.18 |  |
| Upper bound 95%CI | 138.02 | 4.25 | 97.08 | 6.41 |  | 107.68 | 3.24 | 79.38 | 4.68 |  |
| **Cyprus** | 92.06 | 5.92 | 46.17 | 9.34 | 1.58 | 62.52 | 3.43 | 33.00 | 5.21 | 1.52 |
| Lower bound 95%CI | 71.29 | 4.00 | 32.47 | 5.51 |  | 47.34 | 2.25 | 22.53 | 3.03 |  |
| Upper bound 95%CI | 113.40 | 8.37 | 59.16 | 14.12 |  | 76.96 | 4.92 | 42.78 | 7.94 |  |
| **Czechia** | 127.25 | 3.06 | 79.77 | 3.45 | 1.13 | 92.08 | 2.18 | 55.53 | 2.11 | 0.97 |
| Lower bound 95%CI | 99.94 | 2.18 | 60.46 | 2.35 |  | 71.76 | 1.52 | 40.13 | 1.37 |  |
| Upper bound 95%CI | 150.80 | 4.01 | 97.33 | 4.66 |  | 111.76 | 3.00 | 69.85 | 2.99 |  |
| **Denmark** | 46.17 | 2.97 | 25.00 | 4.29 | 1.44 | 31.21 | 1.89 | 15.10 | 2.66 | 1.40 |
| Lower bound 95%CI | 36.40 | 2.06 | 18.74 | 2.85 |  | 24.00 | 1.28 | 11.13 | 1.70 |  |
| Upper bound 95%CI | 55.23 | 3.99 | 30.64 | 5.92 |  | 37.66 | 2.62 | 19.07 | 3.85 |  |
| **Estonia** | 151.25 | 3.20 | 79.15 | 2.62 | 0.82 | 80.59 | 1.61 | 39.44 | 1.23 | 0.76 |
| Lower bound 95%CI | 121.83 | 2.37 | 59.81 | 1.80 |  | 63.40 | 1.14 | 29.52 | 0.82 |  |
| Upper bound 95%CI | 178.81 | 4.12 | 96.52 | 3.50 |  | 97.32 | 2.19 | 48.77 | 1.68 |  |
| **Finland** | 87.72 | 4.21 | 43.00 | 5.20 | 1.23 | 60.29 | 2.49 | 29.03 | 3.19 | 1.28 |
| Lower bound 95%CI | 69.09 | 2.97 | 31.63 | 3.38 |  | 46.64 | 1.70 | 20.91 | 2.02 |  |
| Upper bound 95%CI | 104.76 | 5.60 | 53.73 | 7.28 |  | 73.33 | 3.46 | 36.62 | 4.64 |  |
| **France** | 33.25 | 1.28 | 14.21 | 1.72 | 1.35 | 24.18 | 0.96 | 9.99 | 1.23 | 1.28 |
| Lower bound 95%CI | 26.29 | 0.92 | 10.42 | 1.14 |  | 18.91 | 0.67 | 7.09 | 0.77 |  |
| Upper bound 95%CI | 39.40 | 1.66 | 17.57 | 2.35 |  | 29.50 | 1.31 | 12.80 | 1.77 |  |
| **Germany** | 61.97 | 2.03 | 37.37 | 3.87 | 1.90 | 49.17 | 1.90 | 26.56 | 3.01 | 1.58 |
| Lower bound 95%CI | 49.98 | 1.50 | 28.44 | 2.67 |  | 38.10 | 1.31 | 19.20 | 1.90 |  |
| Upper bound 95%CI | 73.00 | 2.61 | 44.70 | 5.06 |  | 59.23 | 2.58 | 32.18 | 4.14 |  |
| **Greece** | 62.54 | 3.12 | 46.30 | 7.92 | 2.54 | 43.29 | 2.38 | 25.84 | 4.47 | 1.88 |
| Lower bound 95%CI | 49.26 | 2.20 | 35.33 | 5.26 |  | 33.10 | 1.60 | 18.63 | 2.72 |  |
| Upper bound 95%CI | 76.28 | 4.23 | 56.66 | 11.08 |  | 53.21 | 3.32 | 32.17 | 6.51 |  |
| **Hungary** | 166.45 | 3.39 | 106.88 | 3.85 | 1.13 | 127.77 | 2.79 | 81.33 | 3.03 | 1.08 |
| Lower bound 95%CI | 140.64 | 2.61 | 87.86 | 2.87 |  | 102.73 | 2.02 | 64.72 | 2.15 |  |
| Upper bound 95%CI | 191.09 | 4.27 | 124.01 | 4.90 |  | 151.35 | 3.70 | 97.69 | 4.04 |  |
| **Ireland** | 65.85 | 3.01 | 33.53 | 4.56 | 1.51 | 39.42 | 1.81 | 19.85 | 2.65 | 1.46 |
| Lower bound 95%CI | 51.26 | 2.09 | 24.89 | 3.00 |  | 30.01 | 1.22 | 14.33 | 1.67 |  |
| Upper bound 95%CI | 77.85 | 3.92 | 41.08 | 6.29 |  | 47.59 | 2.51 | 25.04 | 3.82 |  |
| **Italy** | 39.54 | 1.62 | 20.54 | 2.27 | 1.40 | 28.33 | 1.23 | 14.02 | 1.60 | 1.31 |
| Lower bound 95%CI | 30.18 | 1.07 | 14.76 | 1.42 |  | 20.85 | 0.76 | 9.49 | 0.92 |  |
| Upper bound 95%CI | 47.40 | 2.24 | 25.13 | 3.19 |  | 35.00 | 1.81 | 18.35 | 2.49 |  |
| **Latvia** | 199.04 | 4.99 | 109.31 | 5.41 | 1.08 | 134.79 | 3.20 | 71.79 | 3.04 | 0.95 |
| Lower bound 95%CI | 157.62 | 3.60 | 84.19 | 3.70 |  | 104.04 | 2.21 | 54.33 | 2.01 |  |
| Upper bound 95%CI | 234.44 | 6.43 | 131.58 | 7.37 |  | 164.09 | 4.38 | 88.00 | 4.23 |  |
| **Lithuania** | 227.64 | 5.55 | 126.03 | 6.26 | 1.13 | 162.60 | 3.91 | 91.25 | 3.95 | 1.01 |
| Lower bound 95%CI | 184.59 | 4.16 | 97.36 | 4.34 |  | 130.58 | 2.82 | 69.75 | 2.68 |  |
| Upper bound 95%CI | 264.44 | 6.91 | 150.04 | 8.28 |  | 194.34 | 5.24 | 112.42 | 5.48 |  |
| **Luxembourg** | 51.75 | 3.28 | 24.49 | 4.00 | 1.22 | 36.32 | 1.98 | 16.93 | 2.47 | 1.25 |
| Lower bound 95%CI | 41.10 | 2.31 | 17.68 | 2.44 |  | 28.78 | 1.38 | 12.54 | 1.59 |  |
| Upper bound 95%CI | 61.47 | 4.42 | 30.21 | 5.81 |  | 43.75 | 2.73 | 21.45 | 3.67 |  |
| **Malta** | 74.62 | 4.04 | 46.87 | 8.00 | 1.98 | 44.01 | 2.24 | 32.44 | 5.05 | 2.25 |
| Lower bound 95%CI | 57.63 | 2.74 | 32.88 | 4.81 |  | 33.35 | 1.49 | 22.05 | 2.98 |  |
| Upper bound 95%CI | 90.00 | 5.49 | 58.96 | 11.69 |  | 53.18 | 3.15 | 41.27 | 7.52 |  |
| **Netherlands** | 38.41 | 1.62 | 16.28 | 2.00 | 1.23 | 29.66 | 1.31 | 13.29 | 1.73 | 1.31 |
| Lower bound 95%CI | 30.02 | 1.14 | 11.62 | 1.27 |  | 23.19 | 0.90 | 9.69 | 1.11 |  |
| Upper bound 95%CI | 45.96 | 2.14 | 20.90 | 2.87 |  | 35.98 | 1.82 | 17.01 | 2.52 |  |
| **Poland** | 106.39 | 2.69 | 57.30 | 2.68 | 1.00 | 76.62 | 1.94 | 41.69 | 1.96 | 1.01 |
| Lower bound 95%CI | 86.57 | 1.92 | 44.75 | 1.81 |  | 59.87 | 1.29 | 29.68 | 1.18 |  |
| Upper bound 95%CI | 123.16 | 3.59 | 67.08 | 3.63 |  | 92.72 | 2.77 | 53.16 | 2.97 |  |
| **Portugal** | 29.60 | 2.02 | 16.27 | 3.34 | 1.66 | 25.64 | 1.76 | 12.09 | 2.27 | 1.29 |
| Lower bound 95%CI | 23.07 | 1.37 | 12.16 | 2.14 |  | 20.13 | 1.20 | 8.70 | 1.34 |  |
| Upper bound 95%CI | 35.25 | 2.77 | 19.93 | 4.78 |  | 30.87 | 2.47 | 15.45 | 3.56 |  |
| **Romania** | 138.18 | 3.57 | 95.71 | 4.34 | 1.21 | 115.25 | 2.79 | 77.14 | 2.95 | 1.06 |
| Lower bound 95%CI | 115.00 | 2.65 | 79.65 | 3.26 |  | 90.72 | 1.95 | 61.04 | 2.09 |  |
| Upper bound 95%CI | 157.92 | 4.56 | 109.75 | 5.52 |  | 137.04 | 3.77 | 93.02 | 3.97 |  |
| **Slovakia** | 168.18 | 4.54 | 112.02 | 5.66 | 1.25 | 129.12 | 3.34 | 91.57 | 3.94 | 1.18 |
| Lower bound 95%CI | 134.04 | 3.22 | 84.96 | 3.84 |  | 100.29 | 2.33 | 68.44 | 2.62 |  |
| Upper bound 95%CI | 199.66 | 5.99 | 135.07 | 7.58 |  | 158.45 | 4.65 | 113.48 | 5.46 |  |
| **Slovenia** | 53.66 | 1.56 | 27.37 | 1.38 | 0.88 | 39.81 | 0.98 | 17.37 | 0.69 | 0.70 |
| Lower bound 95%CI | 42.11 | 1.08 | 20.33 | 0.90 |  | 30.53 | 0.67 | 12.66 | 0.44 |  |
| Upper bound 95%CI | 63.46 | 2.09 | 33.33 | 1.91 |  | 48.67 | 1.38 | 21.64 | 0.97 |  |
| **Spain** | 37.76 | 1.79 | 17.12 | 2.37 | 1.32 | 26.74 | 1.38 | 11.21 | 1.66 | 1.21 |
| Lower bound 95%CI | 30.42 | 1.31 | 12.69 | 1.57 |  | 20.51 | 0.95 | 7.81 | 1.02 |  |
| Upper bound 95%CI | 44.46 | 2.33 | 20.89 | 3.24 |  | 32.40 | 1.88 | 14.50 | 2.45 |  |
| **Sweden** | 61.27 | 3.15 | 34.69 | 3.96 | 1.26 | 36.36 | 1.76 | 20.72 | 2.33 | 1.32 |
| Lower bound 95%CI | 47.81 | 2.13 | 25.78 | 2.54 |  | 27.20 | 1.11 | 14.77 | 1.42 |  |
| Upper bound 95%CI | 73.64 | 4.37 | 42.71 | 5.65 |  | 45.29 | 2.58 | 26.46 | 3.50 |  |
| **European Union** | 63.15 | 2.25 | 36.29 | 3.15 | 1.40 | 46.94 | 1.76 | 26.10 | 2.25 | 1.28 |
| Lower bound 95%CI | 51.60 | 1.67 | 28.37 | 2.21 |  | 37.56 | 1.25 | 20.06 | 1.52 |  |
| Upper bound 95%CI | 73.12 | 2.88 | 42.52 | 4.12 |  | 55.03 | 2.33 | 31.07 | 3.04 |  |
| Data and definitions from Global Burden of Disease Database. 2021  *Each value corresponds to mortality rates per 100.000 inhabitants (95% Uncertainty Interval)  Abbreviations: CFI, case-fatality index | | | | | | | | | | |

| Appendix Table 6. Age-standardised mortality rates and case fatality indexes for IHD attributable to High body-mass index.  2011 , 2021 | | | | | | | | | | |
| --- | --- | --- | --- | --- | --- | --- | --- | --- | --- | --- |
|  | **2011** | | | | | **2021** | | | | |
| **Country** | **Men** | | **Women** | | **CFI Ratio** | **Men** | | **Women** | | **CFI Ratio** |
|  | **Mortality rate** | **CFI** | **Mortality rate** | **CFI** |  | **Mortality rate** | **CFI** | **Mortality rate** | **CFI** |  |
| **Austria** | 13.36 | 0.51 | 7.78 | 0.95 | 1.85 | 10.41 | 0.40 | 5.75 | 0.70 | 1.75 |
| Lower bound 95%CI | 5.06 | 0.18 | 2.92 | 0.32 |  | 4.10 | 0.14 | 2.11 | 0.23 |  |
| Upper bound 95%CI | 22.86 | 0.98 | 13.37 | 1.81 |  | 18.03 | 0.78 | 10.11 | 1.41 |  |
| **Belgium** | 8.73 | 0.40 | 4.38 | 0.58 | 1.46 | 6.31 | 0.29 | 3.06 | 0.40 | 1.40 |
| Lower bound 95%CI | 3.34 | 0.14 | 1.58 | 0.19 |  | 2.40 | 0.10 | 1.13 | 0.13 |  |
| Upper bound 95%CI | 14.56 | 0.74 | 7.51 | 1.12 |  | 10.79 | 0.56 | 5.14 | 0.78 |  |
| **Bulgaria** | 44.22 | 0.96 | 25.06 | 0.97 | 1.01 | 41.10 | 0.94 | 22.61 | 0.90 | 0.95 |
| Lower bound 95%CI | 17.60 | 0.35 | 9.53 | 0.34 |  | 16.10 | 0.33 | 8.99 | 0.32 |  |
| Upper bound 95%CI | 72.70 | 1.76 | 42.33 | 1.81 |  | 69.28 | 1.79 | 39.06 | 1.74 |  |
| **Croatia** | 28.38 | 0.77 | 17.81 | 1.03 | 1.34 | 23.45 | 0.61 | 15.36 | 0.79 | 1.30 |
| Lower bound 95%CI | 11.04 | 0.26 | 6.70 | 0.34 |  | 9.22 | 0.21 | 5.93 | 0.27 |  |
| Upper bound 95%CI | 47.03 | 1.45 | 29.99 | 1.98 |  | 38.94 | 1.17 | 26.40 | 1.56 |  |
| **Cyprus** | 17.03 | 1.10 | 8.16 | 1.65 | 1.51 | 13.45 | 0.74 | 6.68 | 1.05 | 1.43 |
| Lower bound 95%CI | 6.31 | 0.35 | 3.01 | 0.51 |  | 5.44 | 0.26 | 2.47 | 0.33 |  |
| Upper bound 95%CI | 28.29 | 2.09 | 13.93 | 3.33 |  | 23.46 | 1.50 | 11.81 | 2.19 |  |
| **Czechia** | 33.66 | 0.81 | 18.92 | 0.82 | 1.01 | 25.44 | 0.60 | 13.97 | 0.53 | 0.88 |
| Lower bound 95%CI | 12.98 | 0.28 | 7.03 | 0.27 |  | 10.08 | 0.21 | 5.18 | 0.18 |  |
| Upper bound 95%CI | 56.01 | 1.49 | 32.14 | 1.54 |  | 42.60 | 1.14 | 24.13 | 1.03 |  |
| **Denmark** | 8.37 | 0.54 | 4.18 | 0.72 | 1.33 | 6.24 | 0.38 | 2.80 | 0.49 | 1.30 |
| Lower bound 95%CI | 3.15 | 0.18 | 1.50 | 0.23 |  | 2.44 | 0.13 | 1.04 | 0.16 |  |
| Upper bound 95%CI | 13.90 | 1.00 | 7.15 | 1.38 |  | 10.60 | 0.74 | 4.80 | 0.97 |  |
| **Estonia** | 34.73 | 0.74 | 17.61 | 0.58 | 0.79 | 19.59 | 0.39 | 9.07 | 0.28 | 0.72 |
| Lower bound 95%CI | 13.41 | 0.26 | 6.66 | 0.20 |  | 7.62 | 0.14 | 3.37 | 0.09 |  |
| Upper bound 95%CI | 56.07 | 1.29 | 29.90 | 1.08 |  | 32.54 | 0.73 | 16.00 | 0.55 |  |
| **Finland** | 18.45 | 0.89 | 8.77 | 1.06 | 1.20 | 13.70 | 0.57 | 6.36 | 0.70 | 1.24 |
| Lower bound 95%CI | 7.20 | 0.31 | 3.23 | 0.35 |  | 5.37 | 0.20 | 2.39 | 0.23 |  |
| Upper bound 95%CI | 30.87 | 1.65 | 15.07 | 2.04 |  | 23.11 | 1.09 | 10.86 | 1.38 |  |
| **France** | 6.90 | 0.27 | 2.67 | 0.32 | 1.22 | 5.44 | 0.22 | 2.06 | 0.25 | 1.17 |
| Lower bound 95%CI | 2.63 | 0.09 | 1.00 | 0.11 |  | 2.10 | 0.07 | 0.77 | 0.08 |  |
| Upper bound 95%CI | 11.66 | 0.49 | 4.59 | 0.61 |  | 9.06 | 0.40 | 3.52 | 0.49 |  |
| **Germany** | 14.21 | 0.47 | 7.10 | 0.73 | 1.58 | 11.78 | 0.46 | 5.33 | 0.60 | 1.33 |
| Lower bound 95%CI | 5.43 | 0.16 | 2.65 | 0.25 |  | 4.53 | 0.16 | 2.01 | 0.20 |  |
| Upper bound 95%CI | 24.29 | 0.87 | 11.95 | 1.35 |  | 20.02 | 0.87 | 8.71 | 1.12 |  |
| **Greece** | 18.23 | 0.91 | 10.56 | 1.81 | 1.99 | 14.97 | 0.82 | 7.15 | 1.24 | 1.50 |
| Lower bound 95%CI | 7.24 | 0.32 | 3.96 | 0.59 |  | 6.08 | 0.29 | 2.70 | 0.39 |  |
| Upper bound 95%CI | 30.47 | 1.69 | 17.56 | 3.44 |  | 24.79 | 1.54 | 12.02 | 2.43 |  |
| **Hungary** | 41.56 | 0.85 | 22.44 | 0.81 | 0.95 | 34.08 | 0.75 | 18.58 | 0.69 | 0.93 |
| Lower bound 95%CI | 16.53 | 0.31 | 8.58 | 0.28 |  | 13.66 | 0.27 | 7.28 | 0.24 |  |
| Upper bound 95%CI | 67.71 | 1.51 | 37.00 | 1.46 |  | 56.31 | 1.38 | 30.82 | 1.27 |  |
| **Ireland** | 14.81 | 0.68 | 6.86 | 0.93 | 1.38 | 9.64 | 0.44 | 4.40 | 0.59 | 1.33 |
| Lower bound 95%CI | 5.65 | 0.23 | 2.53 | 0.31 |  | 3.77 | 0.15 | 1.68 | 0.20 |  |
| Upper bound 95%CI | 24.25 | 1.22 | 11.59 | 1.78 |  | 16.22 | 0.85 | 7.45 | 1.14 |  |
| **Italy** | 8.96 | 0.37 | 4.37 | 0.48 | 1.32 | 7.27 | 0.31 | 3.44 | 0.39 | 1.25 |
| Lower bound 95%CI | 3.36 | 0.12 | 1.60 | 0.15 |  | 2.70 | 0.10 | 1.27 | 0.12 |  |
| Upper bound 95%CI | 15.28 | 0.72 | 7.46 | 0.95 |  | 12.41 | 0.64 | 5.97 | 0.81 |  |
| **Latvia** | 47.29 | 1.19 | 28.22 | 1.40 | 1.18 | 33.66 | 0.80 | 18.97 | 0.80 | 1.01 |
| Lower bound 95%CI | 18.57 | 0.42 | 11.44 | 0.50 |  | 13.08 | 0.28 | 7.34 | 0.27 |  |
| Upper bound 95%CI | 78.05 | 2.14 | 47.15 | 2.64 |  | 57.59 | 1.54 | 32.29 | 1.55 |  |
| **Lithuania** | 54.71 | 1.33 | 29.84 | 1.48 | 1.11 | 41.43 | 1.00 | 22.37 | 0.97 | 0.97 |
| Lower bound 95%CI | 21.99 | 0.50 | 11.42 | 0.51 |  | 16.35 | 0.35 | 8.71 | 0.33 |  |
| Upper bound 95%CI | 89.74 | 2.35 | 50.96 | 2.81 |  | 69.83 | 1.88 | 38.28 | 1.86 |  |
| **Luxembourg** | 10.78 | 0.68 | 5.24 | 0.86 | 1.25 | 8.26 | 0.45 | 3.88 | 0.56 | 1.26 |
| Lower bound 95%CI | 4.13 | 0.23 | 1.99 | 0.27 |  | 3.17 | 0.15 | 1.49 | 0.19 |  |
| Upper bound 95%CI | 18.09 | 1.30 | 8.90 | 1.71 |  | 13.88 | 0.87 | 6.75 | 1.15 |  |
| **Malta** | 14.49 | 0.78 | 9.73 | 1.66 | 2.12 | 9.75 | 0.50 | 7.48 | 1.16 | 2.34 |
| Lower bound 95%CI | 5.38 | 0.26 | 3.67 | 0.54 |  | 3.81 | 0.17 | 2.86 | 0.39 |  |
| Upper bound 95%CI | 24.11 | 1.47 | 16.64 | 3.30 |  | 16.60 | 0.98 | 12.59 | 2.29 |  |
| **Netherlands** | 7.14 | 0.30 | 3.63 | 0.45 | 1.48 | 5.55 | 0.25 | 2.88 | 0.37 | 1.52 |
| Lower bound 95%CI | 2.75 | 0.10 | 1.38 | 0.15 |  | 2.07 | 0.08 | 1.07 | 0.12 |  |
| Upper bound 95%CI | 11.83 | 0.55 | 6.17 | 0.85 |  | 9.47 | 0.48 | 4.89 | 0.72 |  |
| **Poland** | 24.21 | 0.61 | 13.90 | 0.65 | 1.06 | 20.63 | 0.52 | 12.01 | 0.56 | 1.08 |
| Lower bound 95%CI | 9.53 | 0.21 | 5.52 | 0.22 |  | 7.90 | 0.17 | 4.73 | 0.19 |  |
| Upper bound 95%CI | 41.48 | 1.21 | 23.24 | 1.26 |  | 34.78 | 1.04 | 20.29 | 1.13 |  |
| **Portugal** | 6.70 | 0.46 | 3.91 | 0.80 | 1.76 | 6.62 | 0.45 | 3.28 | 0.62 | 1.35 |
| Lower bound 95%CI | 2.53 | 0.15 | 1.49 | 0.26 |  | 2.53 | 0.15 | 1.24 | 0.19 |  |
| Upper bound 95%CI | 11.26 | 0.88 | 6.71 | 1.61 |  | 11.02 | 0.88 | 5.72 | 1.32 |  |
| **Romania** | 32.88 | 0.85 | 19.48 | 0.88 | 1.04 | 30.02 | 0.73 | 17.08 | 0.65 | 0.90 |
| Lower bound 95%CI | 12.92 | 0.30 | 7.38 | 0.30 |  | 11.47 | 0.25 | 6.56 | 0.22 |  |
| Upper bound 95%CI | 53.25 | 1.54 | 31.87 | 1.60 |  | 49.59 | 1.36 | 29.34 | 1.25 |  |
| **Slovakia** | 42.16 | 1.14 | 26.94 | 1.36 | 1.20 | 34.03 | 0.88 | 23.68 | 1.02 | 1.16 |
| Lower bound 95%CI | 16.91 | 0.41 | 10.67 | 0.48 |  | 14.31 | 0.33 | 9.43 | 0.36 |  |
| Upper bound 95%CI | 69.77 | 2.09 | 44.28 | 2.49 |  | 57.35 | 1.68 | 40.25 | 1.94 |  |
| **Slovenia** | 14.09 | 0.41 | 6.09 | 0.31 | 0.75 | 11.05 | 0.27 | 4.17 | 0.16 | 0.60 |
| Lower bound 95%CI | 5.69 | 0.15 | 2.32 | 0.10 |  | 4.31 | 0.09 | 1.58 | 0.06 |  |
| Upper bound 95%CI | 23.07 | 0.76 | 10.35 | 0.59 |  | 18.72 | 0.53 | 7.22 | 0.32 |  |
| **Spain** | 9.70 | 0.46 | 4.12 | 0.57 | 1.24 | 7.97 | 0.41 | 3.19 | 0.47 | 1.15 |
| Lower bound 95%CI | 3.78 | 0.16 | 1.54 | 0.19 |  | 3.11 | 0.14 | 1.24 | 0.16 |  |
| Upper bound 95%CI | 16.10 | 0.84 | 7.10 | 1.10 |  | 13.51 | 0.79 | 5.47 | 0.92 |  |
| **Sweden** | 11.41 | 0.59 | 6.20 | 0.71 | 1.21 | 7.34 | 0.36 | 4.15 | 0.47 | 1.31 |
| Lower bound 95%CI | 4.30 | 0.19 | 2.32 | 0.23 |  | 2.92 | 0.12 | 1.57 | 0.15 |  |
| Upper bound 95%CI | 19.37 | 1.15 | 10.63 | 1.41 |  | 12.56 | 0.72 | 7.26 | 0.96 |  |
| **European Union** | 14.90 | 0.53 | 7.84 | 0.68 | 1.28 | 12.06 | 0.45 | 6.22 | 0.54 | 1.19 |
| Lower bound 95%CI | 5.78 | 0.19 | 3.00 | 0.23 |  | 4.71 | 0.16 | 2.39 | 0.18 |  |
| Upper bound 95%CI | 24.52 | 0.97 | 13.24 | 1.28 |  | 20.20 | 0.86 | 10.51 | 1.03 |  |

Data and definitions from Global Burden of Disease Database. 2021

*Each value corresponds to mortality rates per 100.000 inhabitants (95% Uncertainty Interval)

Abbreviations: CFI, case-fatality index

| Appendix Table 7. Age-standardised mortality rates and case fatality indexes for IHD attributable to High fasting plasma glucose. 2011 , 2021 | | | | | | | | | | |
| --- | --- | --- | --- | --- | --- | --- | --- | --- | --- | --- |
| **Country** | **2011** | | | | | **2021** | | | | |
|  | **Men** | | **Women** | | **CFI Ratio** | **Men** | | **Women** | | **CFI Ratio** |
|  | **Mortality rate** | **CFI** | **Mortality rate** | **CFI** |  | **Mortality rate** | **CFI** | **Mortality rate** | **CFI** |  |
| **Austria** | 10.70 | 0.41 | 6.27 | 0.76 | 1.86 | 8.77 | 0.34 | 4.67 | 0.57 | 1.69 |
| Lower bound 95%CI | 8.80 | 0.31 | 4.90 | 0.54 |  | 7.16 | 0.25 | 3.47 | 0.38 |  |
| Upper bound 95%CI | 12.86 | 0.55 | 7.60 | 1.03 |  | 10.53 | 0.46 | 5.74 | 0.80 |  |
| **Belgium** | 10.14 | 0.46 | 4.47 | 0.60 | 1.28 | 7.68 | 0.35 | 3.25 | 0.43 | 1.22 |
| Lower bound 95%CI | 8.26 | 0.34 | 3.44 | 0.41 |  | 6.24 | 0.25 | 2.49 | 0.29 |  |
| Upper bound 95%CI | 11.99 | 0.61 | 5.47 | 0.81 |  | 9.14 | 0.48 | 3.99 | 0.61 |  |
| **Bulgaria** | 48.79 | 1.06 | 29.88 | 1.16 | 1.09 | 45.72 | 1.05 | 26.18 | 1.04 | 0.99 |
| Lower bound 95%CI | 41.92 | 0.83 | 25.05 | 0.89 |  | 37.72 | 0.78 | 21.45 | 0.76 |  |
| Upper bound 95%CI | 56.16 | 1.36 | 34.93 | 1.49 |  | 54.62 | 1.41 | 31.06 | 1.38 |  |
| **Croatia** | 35.23 | 0.96 | 23.23 | 1.35 | 1.41 | 28.91 | 0.76 | 19.83 | 1.03 | 1.36 |
| Lower bound 95%CI | 29.98 | 0.71 | 19.24 | 0.98 |  | 24.33 | 0.56 | 16.30 | 0.74 |  |
| Upper bound 95%CI | 40.76 | 1.26 | 27.52 | 1.82 |  | 34.57 | 1.04 | 23.71 | 1.40 |  |
| **Cyprus** | 27.59 | 1.77 | 13.36 | 2.70 | 1.52 | 18.78 | 1.03 | 9.37 | 1.48 | 1.44 |
| Lower bound 95%CI | 22.79 | 1.28 | 10.68 | 1.81 |  | 15.38 | 0.73 | 7.22 | 0.97 |  |
| Upper bound 95%CI | 32.84 | 2.42 | 16.34 | 3.90 |  | 22.58 | 1.44 | 11.47 | 2.13 |  |
| **Czechia** | 44.06 | 1.06 | 25.40 | 1.10 | 1.04 | 34.67 | 0.82 | 19.94 | 0.76 | 0.92 |
| Lower bound 95%CI | 37.84 | 0.83 | 21.10 | 0.82 |  | 28.74 | 0.61 | 16.35 | 0.56 |  |
| Upper bound 95%CI | 51.09 | 1.36 | 29.64 | 1.42 |  | 41.52 | 1.11 | 24.05 | 1.03 |  |
| **Denmark** | 8.48 | 0.55 | 3.54 | 0.61 | 1.11 | 6.61 | 0.40 | 2.58 | 0.45 | 1.13 |
| Lower bound 95%CI | 6.99 | 0.39 | 2.78 | 0.42 |  | 5.40 | 0.29 | 1.99 | 0.30 |  |
| Upper bound 95%CI | 10.13 | 0.73 | 4.40 | 0.85 |  | 7.89 | 0.55 | 3.22 | 0.65 |  |
| **Estonia** | 31.15 | 0.66 | 16.56 | 0.55 | 0.83 | 18.51 | 0.37 | 8.47 | 0.26 | 0.71 |
| Lower bound 95%CI | 26.13 | 0.51 | 13.27 | 0.40 |  | 15.20 | 0.27 | 6.64 | 0.19 |  |
| Upper bound 95%CI | 37.27 | 0.86 | 19.96 | 0.72 |  | 22.29 | 0.50 | 10.43 | 0.36 |  |
| **Finland** | 24.85 | 1.19 | 12.13 | 1.47 | 1.23 | 18.75 | 0.77 | 9.04 | 0.99 | 1.28 |
| Lower bound 95%CI | 20.85 | 0.90 | 9.50 | 1.02 |  | 15.46 | 0.56 | 7.02 | 0.68 |  |
| Upper bound 95%CI | 28.84 | 1.54 | 14.69 | 1.99 |  | 21.81 | 1.03 | 10.76 | 1.36 |  |
| **France** | 6.79 | 0.26 | 2.21 | 0.27 | 1.03 | 5.41 | 0.22 | 1.71 | 0.21 | 0.98 |
| Lower bound 95%CI | 5.64 | 0.20 | 1.69 | 0.18 |  | 4.41 | 0.16 | 1.28 | 0.14 |  |
| Upper bound 95%CI | 8.12 | 0.34 | 2.72 | 0.36 |  | 6.48 | 0.29 | 2.12 | 0.29 |  |
| **Germany** | 16.60 | 0.54 | 8.93 | 0.92 | 1.70 | 14.42 | 0.56 | 7.06 | 0.80 | 1.43 |
| Lower bound 95%CI | 14.15 | 0.43 | 7.12 | 0.67 |  | 12.17 | 0.42 | 5.42 | 0.54 |  |
| Upper bound 95%CI | 19.27 | 0.69 | 10.62 | 1.20 |  | 16.84 | 0.73 | 8.51 | 1.09 |  |
| **Greece** | 17.23 | 0.86 | 11.50 | 1.97 | 2.29 | 13.37 | 0.74 | 7.02 | 1.21 | 1.65 |
| Lower bound 95%CI | 14.48 | 0.65 | 9.35 | 1.39 |  | 11.11 | 0.54 | 5.61 | 0.82 |  |
| Upper bound 95%CI | 20.11 | 1.12 | 13.86 | 2.71 |  | 15.46 | 0.96 | 8.54 | 1.73 |  |
| **Hungary** | 45.90 | 0.94 | 25.69 | 0.92 | 0.99 | 36.35 | 0.79 | 20.42 | 0.76 | 0.96 |
| Lower bound 95%CI | 39.21 | 0.73 | 21.75 | 0.71 |  | 30.09 | 0.59 | 17.01 | 0.56 |  |
| Upper bound 95%CI | 52.66 | 1.18 | 30.16 | 1.19 |  | 43.53 | 1.06 | 24.20 | 1.00 |  |
| **Ireland** | 14.99 | 0.68 | 6.65 | 0.90 | 1.32 | 10.37 | 0.48 | 4.65 | 0.62 | 1.30 |
| Lower bound 95%CI | 12.61 | 0.52 | 5.22 | 0.63 |  | 8.44 | 0.34 | 3.53 | 0.41 |  |
| Upper bound 95%CI | 17.63 | 0.89 | 8.16 | 1.25 |  | 12.31 | 0.65 | 5.77 | 0.88 |  |
| **Italy** | 11.64 | 0.48 | 5.91 | 0.65 | 1.37 | 9.16 | 0.40 | 4.50 | 0.52 | 1.30 |
| Lower bound 95%CI | 9.68 | 0.34 | 4.43 | 0.42 |  | 7.56 | 0.27 | 3.35 | 0.32 |  |
| Upper bound 95%CI | 13.54 | 0.64 | 7.24 | 0.92 |  | 10.73 | 0.56 | 5.51 | 0.75 |  |
| **Latvia** | 36.29 | 0.91 | 21.65 | 1.07 | 1.18 | 27.83 | 0.66 | 15.07 | 0.64 | 0.97 |
| Lower bound 95%CI | 30.09 | 0.69 | 18.16 | 0.80 |  | 22.18 | 0.47 | 12.02 | 0.45 |  |
| Upper bound 95%CI | 43.11 | 1.18 | 25.59 | 1.43 |  | 33.52 | 0.89 | 18.40 | 0.89 |  |
| **Lithuania** | 37.11 | 0.90 | 21.76 | 1.08 | 1.19 | 29.83 | 0.72 | 16.76 | 0.73 | 1.01 |
| Lower bound 95%CI | 30.66 | 0.69 | 17.30 | 0.77 |  | 24.46 | 0.53 | 13.17 | 0.51 |  |
| Upper bound 95%CI | 43.95 | 1.15 | 26.00 | 1.43 |  | 35.41 | 0.95 | 20.49 | 1.00 |  |
| **Luxembourg** | 14.92 | 0.95 | 5.40 | 0.88 | 0.93 | 11.74 | 0.64 | 5.26 | 0.77 | 1.20 |
| Lower bound 95%CI | 12.58 | 0.71 | 4.32 | 0.60 |  | 9.80 | 0.47 | 4.22 | 0.53 |  |
| Upper bound 95%CI | 17.16 | 1.23 | 6.48 | 1.25 |  | 13.75 | 0.86 | 6.21 | 1.06 |  |
| **Malta** | 19.28 | 1.04 | 11.48 | 1.96 | 1.88 | 13.70 | 0.70 | 9.41 | 1.46 | 2.10 |
| Lower bound 95%CI | 16.02 | 0.76 | 9.22 | 1.35 |  | 11.54 | 0.52 | 7.46 | 1.01 |  |
| Upper bound 95%CI | 22.59 | 1.38 | 13.63 | 2.70 |  | 16.11 | 0.95 | 11.30 | 2.06 |  |
| **Netherlands** | 8.81 | 0.37 | 3.53 | 0.43 | 1.17 | 7.30 | 0.32 | 2.89 | 0.38 | 1.16 |
| Lower bound 95%CI | 7.29 | 0.28 | 2.72 | 0.30 |  | 6.07 | 0.24 | 2.18 | 0.25 |  |
| Upper bound 95%CI | 10.37 | 0.48 | 4.23 | 0.58 |  | 8.66 | 0.44 | 3.55 | 0.53 |  |
| **Poland** | 30.47 | 0.77 | 17.44 | 0.82 | 1.06 | 28.57 | 0.72 | 16.14 | 0.76 | 1.05 |
| Lower bound 95%CI | 26.06 | 0.58 | 14.36 | 0.58 |  | 24.14 | 0.52 | 13.11 | 0.52 |  |
| Upper bound 95%CI | 35.15 | 1.02 | 20.46 | 1.11 |  | 33.56 | 1.00 | 19.27 | 1.08 |  |
| **Portugal** | 10.35 | 0.71 | 6.01 | 1.24 | 1.75 | 9.02 | 0.62 | 4.73 | 0.89 | 1.43 |
| Lower bound 95%CI | 8.85 | 0.53 | 4.78 | 0.84 |  | 7.77 | 0.46 | 3.70 | 0.57 |  |
| Upper bound 95%CI | 12.00 | 0.94 | 7.12 | 1.71 |  | 10.41 | 0.83 | 5.62 | 1.30 |  |
| **Romania** | 31.12 | 0.80 | 19.83 | 0.90 | 1.12 | 29.06 | 0.70 | 18.31 | 0.70 | 0.99 |
| Lower bound 95%CI | 26.01 | 0.60 | 16.64 | 0.68 |  | 23.92 | 0.52 | 14.91 | 0.51 |  |
| Upper bound 95%CI | 36.68 | 1.06 | 23.88 | 1.20 |  | 35.06 | 0.96 | 22.19 | 0.95 |  |
| **Slovakia** | 43.71 | 1.18 | 28.68 | 1.45 | 1.23 | 36.10 | 0.93 | 25.89 | 1.11 | 1.19 |
| Lower bound 95%CI | 36.35 | 0.87 | 23.38 | 1.06 |  | 29.16 | 0.68 | 19.95 | 0.76 |  |
| Upper bound 95%CI | 51.27 | 1.54 | 34.25 | 1.92 |  | 43.45 | 1.28 | 31.58 | 1.52 |  |
| **Slovenia** | 15.72 | 0.46 | 7.47 | 0.38 | 0.82 | 12.24 | 0.30 | 5.03 | 0.20 | 0.66 |
| Lower bound 95%CI | 13.20 | 0.34 | 6.06 | 0.27 |  | 10.07 | 0.22 | 3.86 | 0.14 |  |
| Upper bound 95%CI | 18.23 | 0.60 | 8.88 | 0.51 |  | 14.76 | 0.42 | 6.17 | 0.28 |  |
| **Spain** | 11.80 | 0.56 | 5.43 | 0.75 | 1.34 | 8.89 | 0.46 | 3.73 | 0.55 | 1.21 |
| Lower bound 95%CI | 10.20 | 0.44 | 4.23 | 0.52 |  | 7.53 | 0.35 | 2.80 | 0.37 |  |
| Upper bound 95%CI | 13.65 | 0.72 | 6.54 | 1.01 |  | 10.30 | 0.60 | 4.53 | 0.76 |  |
| **Sweden** | 16.25 | 0.84 | 7.60 | 0.87 | 1.04 | 10.67 | 0.52 | 5.07 | 0.57 | 1.10 |
| Lower bound 95%CI | 13.48 | 0.60 | 6.12 | 0.60 |  | 8.72 | 0.36 | 3.76 | 0.36 |  |
| Upper bound 95%CI | 19.17 | 1.14 | 9.17 | 1.21 |  | 13.13 | 0.75 | 6.37 | 0.84 |  |
| **European Union** | 16.66 | 0.59 | 9.01 | 0.78 | 1.32 | 13.76 | 0.52 | 7.29 | 0.63 | 1.22 |
| Lower bound 95%CI | 14.33 | 0.46 | 7.41 | 0.58 |  | 11.79 | 0.39 | 5.94 | 0.45 |  |
| Upper bound 95%CI | 19.05 | 0.75 | 10.45 | 1.01 |  | 15.76 | 0.67 | 8.54 | 0.84 |  |

Data and definitions from Global Burden of Disease Database. 2021

*Each value corresponds to mortality rates per 100.000 inhabitants (95% Uncertainty Interval)

| Appendix Table 8. Age-standardised mortality rates and case fatality indexes for IHD attributable to Tobacco. 2011 , 2021 | | | | | | | | | | |
| --- | --- | --- | --- | --- | --- | --- | --- | --- | --- | --- |
| **Country** | **2011** | | | | | **2021** | | | | |
|  | **Men** | | **Women** | | **CFI Ratio** | **Men** | | **Women** | | **CFI Ratio** |
|  | **Mortality rates** | **CFI** | **Mortality rates** | **CFI** |  | **Mortality rates** | **CFI** | **Mortality rates** | **CFI** |  |
| **Austria** | 28.99 | 1.11 | 6.87 | 0.84 | 0.75 | 19.81 | 0.76 | 4.05 | 0.49 | 0.65 |
| Lower bound 95%CI | 24.13 | 0.85 | 5.24 | 0.58 |  | 16.11 | 0.55 | 2.99 | 0.32 |  |
| Upper bound 95%CI | 34.51 | 1.48 | 8.66 | 1.17 |  | 23.96 | 1.04 | 5.28 | 0.74 |  |
| **Belgium** | 21.26 | 0.97 | 4.76 | 0.63 | 0.65 | 12.18 | 0.56 | 2.56 | 0.34 | 0.61 |
| Lower bound 95%CI | 17.62 | 0.73 | 3.69 | 0.44 |  | 9.84 | 0.39 | 1.90 | 0.22 |  |
| Upper bound 95%CI | 25.33 | 1.28 | 5.90 | 0.88 |  | 14.75 | 0.77 | 3.30 | 0.50 |  |
| **Bulgaria** | 77.89 | 1.70 | 19.69 | 0.76 | 0.45 | 62.03 | 1.42 | 15.26 | 0.61 | 0.43 |
| Lower bound 95%CI | 65.34 | 1.30 | 14.42 | 0.51 |  | 48.98 | 1.01 | 10.94 | 0.39 |  |
| Upper bound 95%CI | 92.58 | 2.24 | 25.32 | 1.08 |  | 77.23 | 2.00 | 20.09 | 0.89 |  |
| **Croatia** | 61.04 | 1.66 | 22.78 | 1.32 | 0.80 | 42.34 | 1.11 | 15.82 | 0.82 | 0.74 |
| Lower bound 95%CI | 49.86 | 1.18 | 16.20 | 0.82 |  | 33.28 | 0.76 | 11.14 | 0.50 |  |
| Upper bound 95%CI | 73.72 | 2.27 | 29.88 | 1.97 |  | 53.19 | 1.60 | 21.86 | 1.29 |  |
| **Cyprus** | 45.30 | 2.91 | 7.41 | 1.50 | 0.51 | 31.00 | 1.70 | 4.71 | 0.74 | 0.44 |
| Lower bound 95%CI | 36.12 | 2.03 | 5.23 | 0.89 |  | 24.38 | 1.16 | 3.23 | 0.43 |  |
| Upper bound 95%CI | 55.64 | 4.11 | 10.00 | 2.39 |  | 38.87 | 2.49 | 6.45 | 1.20 |  |
| **Czechia** | 51.50 | 1.24 | 16.73 | 0.72 | 0.58 | 36.00 | 0.85 | 10.80 | 0.41 | 0.48 |
| Lower bound 95%CI | 42.29 | 0.92 | 12.13 | 0.47 |  | 27.85 | 0.59 | 7.59 | 0.26 |  |
| Upper bound 95%CI | 61.57 | 1.64 | 21.61 | 1.03 |  | 44.57 | 1.19 | 14.47 | 0.62 |  |
| **Denmark** | 23.96 | 1.54 | 7.07 | 1.21 | 0.79 | 14.11 | 0.86 | 3.71 | 0.65 | 0.76 |
| Lower bound 95%CI | 19.61 | 1.11 | 5.52 | 0.84 |  | 11.32 | 0.60 | 2.82 | 0.43 |  |
| Upper bound 95%CI | 28.38 | 2.05 | 8.85 | 1.71 |  | 17.26 | 1.20 | 4.77 | 0.96 |  |
| **Estonia** | 61.65 | 1.31 | 11.11 | 0.37 | 0.28 | 28.27 | 0.56 | 4.31 | 0.13 | 0.24 |
| Lower bound 95%CI | 50.39 | 0.98 | 8.09 | 0.24 |  | 22.13 | 0.40 | 3.08 | 0.09 |  |
| Upper bound 95%CI | 72.58 | 1.67 | 14.60 | 0.53 |  | 34.71 | 0.78 | 5.71 | 0.20 |  |
| **Finland** | 26.89 | 1.29 | 4.74 | 0.57 | 0.44 | 16.17 | 0.67 | 2.68 | 0.29 | 0.44 |
| Lower bound 95%CI | 21.77 | 0.94 | 3.56 | 0.38 |  | 12.92 | 0.47 | 1.99 | 0.19 |  |
| Upper bound 95%CI | 32.38 | 1.73 | 6.04 | 0.82 |  | 19.98 | 0.94 | 3.51 | 0.45 |  |
| **France** | 12.55 | 0.48 | 2.13 | 0.26 | 0.54 | 8.37 | 0.33 | 1.36 | 0.17 | 0.50 |
| Lower bound 95%CI | 10.46 | 0.36 | 1.60 | 0.17 |  | 6.74 | 0.24 | 1.01 | 0.11 |  |
| Upper bound 95%CI | 15.27 | 0.64 | 2.77 | 0.37 |  | 10.13 | 0.45 | 1.73 | 0.24 |  |
| **Germany** | 23.64 | 0.78 | 5.35 | 0.55 | 0.71 | 17.40 | 0.67 | 3.62 | 0.41 | 0.61 |
| Lower bound 95%CI | 19.19 | 0.58 | 3.97 | 0.37 |  | 13.79 | 0.47 | 2.68 | 0.27 |  |
| Upper bound 95%CI | 28.19 | 1.01 | 6.74 | 0.76 |  | 21.41 | 0.93 | 4.69 | 0.60 |  |
| **Greece** | 44.81 | 2.23 | 12.21 | 2.09 | 0.94 | 31.08 | 1.71 | 6.83 | 1.18 | 0.69 |
| Lower bound 95%CI | 37.97 | 1.69 | 9.07 | 1.35 |  | 26.16 | 1.27 | 5.13 | 0.75 |  |
| Upper bound 95%CI | 51.86 | 2.88 | 15.77 | 3.08 |  | 36.81 | 2.29 | 8.71 | 1.76 |  |
| **Hungary** | 59.10 | 1.20 | 16.79 | 0.60 | 0.50 | 41.18 | 0.90 | 11.00 | 0.41 | 0.46 |
| Lower bound 95%CI | 49.67 | 0.92 | 12.42 | 0.41 |  | 33.09 | 0.65 | 8.17 | 0.27 |  |
| Upper bound 95%CI | 70.08 | 1.56 | 21.52 | 0.85 |  | 50.17 | 1.23 | 14.49 | 0.60 |  |
| **Ireland** | 25.31 | 1.16 | 7.81 | 1.06 | 0.92 | 13.03 | 0.60 | 4.06 | 0.54 | 0.91 |
| Lower bound 95%CI | 20.42 | 0.83 | 5.85 | 0.71 |  | 10.26 | 0.42 | 2.79 | 0.32 |  |
| Upper bound 95%CI | 30.32 | 1.53 | 9.95 | 1.52 |  | 16.04 | 0.85 | 5.42 | 0.83 |  |
| **Italy** | 17.42 | 0.71 | 3.54 | 0.39 | 0.55 | 11.85 | 0.51 | 2.11 | 0.24 | 0.47 |
| Lower bound 95%CI | 14.32 | 0.51 | 2.69 | 0.26 |  | 9.49 | 0.34 | 1.57 | 0.15 |  |
| Upper bound 95%CI | 20.89 | 0.99 | 4.53 | 0.57 |  | 14.44 | 0.75 | 2.80 | 0.38 |  |
| **Latvia** | 86.07 | 2.16 | 13.92 | 0.69 | 0.32 | 53.19 | 1.26 | 7.47 | 0.32 | 0.25 |
| Lower bound 95%CI | 72.35 | 1.65 | 9.77 | 0.43 |  | 42.41 | 0.90 | 5.12 | 0.19 |  |
| Upper bound 95%CI | 101.29 | 2.78 | 20.10 | 1.13 |  | 64.07 | 1.71 | 10.90 | 0.52 |  |
| **Lithuania** | 99.48 | 2.42 | 10.96 | 0.54 | 0.22 | 63.36 | 1.52 | 6.58 | 0.29 | 0.19 |
| Lower bound 95%CI | 82.63 | 1.86 | 7.70 | 0.34 |  | 50.88 | 1.10 | 4.55 | 0.17 |  |
| Upper bound 95%CI | 119.01 | 3.11 | 15.50 | 0.86 |  | 77.74 | 2.09 | 9.70 | 0.47 |  |
| **Luxembourg** | 18.74 | 1.19 | 4.65 | 0.76 | 0.64 | 12.64 | 0.69 | 2.75 | 0.40 | 0.58 |
| Lower bound 95%CI | 14.56 | 0.82 | 3.27 | 0.45 |  | 9.61 | 0.46 | 1.86 | 0.24 |  |
| Upper bound 95%CI | 23.49 | 1.69 | 6.25 | 1.20 |  | 16.27 | 1.02 | 3.71 | 0.63 |  |
| **Malta** | 28.42 | 1.54 | 6.96 | 1.19 | 0.77 | 16.24 | 0.83 | 4.49 | 0.70 | 0.85 |
| Lower bound 95%CI | 23.38 | 1.11 | 5.10 | 0.75 |  | 13.01 | 0.58 | 3.14 | 0.42 |  |
| Upper bound 95%CI | 34.23 | 2.09 | 9.15 | 1.81 |  | 20.06 | 1.19 | 5.99 | 1.09 |  |
| **Netherlands** | 15.79 | 0.67 | 4.41 | 0.54 | 0.81 | 10.13 | 0.45 | 2.77 | 0.36 | 0.80 |
| Lower bound 95%CI | 12.91 | 0.49 | 3.45 | 0.38 |  | 8.09 | 0.31 | 2.04 | 0.23 |  |
| Upper bound 95%CI | 19.05 | 0.89 | 5.47 | 0.75 |  | 12.56 | 0.64 | 3.61 | 0.54 |  |
| **Poland** | 45.44 | 1.15 | 9.78 | 0.46 | 0.40 | 32.05 | 0.81 | 6.73 | 0.32 | 0.39 |
| Lower bound 95%CI | 38.00 | 0.84 | 7.56 | 0.31 |  | 25.87 | 0.56 | 5.06 | 0.20 |  |
| Upper bound 95%CI | 52.97 | 1.54 | 12.27 | 0.66 |  | 39.00 | 1.16 | 8.80 | 0.49 |  |
| **Portugal** | 10.89 | 0.74 | 1.67 | 0.34 | 0.46 | 9.40 | 0.65 | 1.24 | 0.23 | 0.36 |
| Lower bound 95%CI | 8.90 | 0.53 | 1.15 | 0.20 |  | 7.73 | 0.46 | 0.90 | 0.14 |  |
| Upper bound 95%CI | 13.13 | 1.03 | 2.39 | 0.57 |  | 11.10 | 0.89 | 1.72 | 0.40 |  |
| **Romania** | 49.20 | 1.27 | 12.02 | 0.55 | 0.43 | 39.37 | 0.95 | 8.90 | 0.34 | 0.36 |
| Lower bound 95%CI | 40.92 | 0.94 | 8.87 | 0.36 |  | 31.75 | 0.68 | 6.32 | 0.22 |  |
| Upper bound 95%CI | 58.36 | 1.69 | 16.27 | 0.82 |  | 48.39 | 1.33 | 12.38 | 0.53 |  |
| **Slovakia** | 67.12 | 1.81 | 13.64 | 0.69 | 0.38 | 51.05 | 1.32 | 11.10 | 0.48 | 0.36 |
| Lower bound 95%CI | 54.20 | 1.30 | 9.30 | 0.42 |  | 39.83 | 0.92 | 7.34 | 0.28 |  |
| Upper bound 95%CI | 83.51 | 2.51 | 19.58 | 1.10 |  | 65.37 | 1.92 | 16.42 | 0.79 |  |
| **Slovenia** | 20.96 | 0.61 | 5.22 | 0.26 | 0.43 | 13.30 | 0.33 | 2.66 | 0.10 | 0.32 |
| Lower bound 95%CI | 17.05 | 0.44 | 3.79 | 0.17 |  | 10.43 | 0.23 | 1.85 | 0.06 |  |
| Upper bound 95%CI | 25.45 | 0.84 | 6.67 | 0.38 |  | 16.77 | 0.48 | 3.51 | 0.16 |  |
| **Spain** | 17.43 | 0.83 | 2.59 | 0.36 | 0.43 | 11.89 | 0.61 | 1.51 | 0.22 | 0.37 |
| Lower bound 95%CI | 14.42 | 0.62 | 1.84 | 0.23 |  | 9.62 | 0.45 | 1.07 | 0.14 |  |
| Upper bound 95%CI | 20.60 | 1.08 | 3.53 | 0.55 |  | 14.36 | 0.83 | 2.04 | 0.34 |  |
| **Sweden** | 23.10 | 1.19 | 7.56 | 0.86 | 0.73 | 13.05 | 0.63 | 4.25 | 0.48 | 0.75 |
| Lower bound 95%CI | 18.27 | 0.81 | 5.73 | 0.57 |  | 9.81 | 0.40 | 3.03 | 0.29 |  |
| Upper bound 95%CI | 28.20 | 1.67 | 9.78 | 1.29 |  | 16.73 | 0.95 | 5.75 | 0.76 |  |
| **European Union** | 26.80 | 0.95 | 6.07 | 0.53 | 0.55 | 18.59 | 0.70 | 3.95 | 0.34 | 0.49 |
| Lower bound 95%CI | 22.67 | 0.73 | 4.87 | 0.38 |  | 15.63 | 0.52 | 3.11 | 0.23 |  |
| Upper bound 95%CI | 31.25 | 1.23 | 7.45 | 0.72 |  | 22.08 | 0.94 | 4.85 | 0.47 |  |

Data and definitions from Global Burden of Disease Database. 2021

*Each value corresponds to mortality rates per 100.000 inhabitants (95% Uncertainty Interval)

Abbreviation: CFI, case-fatality index

| Appendix Table 9. Age-standardised mortality rates and case fatality indexes for IHD attributable to Air pollution. 2011 , 2021 | | | | | | | | | | |
| --- | --- | --- | --- | --- | --- | --- | --- | --- | --- | --- |
| **Country** | **2011** | | | | | **2021** | | | | |
|  | **Men** | | **Women** | |  | **Men** | | **Women** | |  |
|  | **Mortality Rates** | **CFI** | **Mortality Rates** | **CFI** | **CFI Ratio** | **Mortality Rates** | **CFI** | **Mortality Rates** | **CFI** | **CFI Ratio** |
| **Austria** | 19.27 | 0.74 | 10.97 | 1.34 | 1.80 | 9.70 | 0.37 | 5.18 | 0.63 | 1.70 |
| Lower bound 95%CI | 13.81 | 0.48 | 7.35 | 0.81 |  | 6.42 | 0.22 | 3.29 | 0.36 |  |
| Upper bound 95%CI | 24.94 | 1.07 | 14.55 | 1.97 |  | 13.16 | 0.57 | 7.19 | 1.00 |  |
| **Belgium** | 13.51 | 0.62 | 6.57 | 0.87 | 1.41 | 5.56 | 0.25 | 2.56 | 0.34 | 1.33 |
| Lower bound 95%CI | 9.69 | 0.40 | 4.49 | 0.54 |  | 3.60 | 0.14 | 1.58 | 0.18 |  |
| Upper bound 95%CI | 17.38 | 0.88 | 8.57 | 1.27 |  | 7.53 | 0.39 | 3.58 | 0.54 |  |
| **Bulgaria** | 70.84 | 1.54 | 44.49 | 1.73 | 1.12 | 48.91 | 1.12 | 28.99 | 1.15 | 1.03 |
| Lower bound 95%CI | 51.82 | 1.03 | 32.21 | 1.15 |  | 34.99 | 0.72 | 20.65 | 0.73 |  |
| Upper bound 95%CI | 92.50 | 2.24 | 58.21 | 2.49 |  | 69.91 | 1.81 | 41.46 | 1.85 |  |
| **Croatia** | 42.30 | 1.15 | 28.69 | 1.67 | 1.45 | 24.69 | 0.65 | 17.34 | 0.90 | 1.39 |
| Lower bound 95%CI | 31.18 | 0.74 | 20.90 | 1.06 |  | 17.53 | 0.40 | 12.01 | 0.54 |  |
| Upper bound 95%CI | 53.46 | 1.65 | 36.82 | 2.43 |  | 32.37 | 0.97 | 23.24 | 1.37 |  |
| **Cyprus** | 31.06 | 2.00 | 15.67 | 3.17 | 1.59 | 17.11 | 0.94 | 9.16 | 1.45 | 1.54 |
| Lower bound 95%CI | 22.26 | 1.25 | 10.79 | 1.83 |  | 11.58 | 0.55 | 6.15 | 0.83 |  |
| Upper bound 95%CI | 40.55 | 2.99 | 20.86 | 4.98 |  | 23.30 | 1.49 | 12.77 | 2.37 |  |
| **Czechia** | 45.59 | 1.10 | 28.11 | 1.22 | 1.11 | 22.83 | 0.54 | 13.48 | 0.51 | 0.95 |
| Lower bound 95%CI | 34.20 | 0.75 | 20.43 | 0.79 |  | 16.01 | 0.34 | 9.34 | 0.32 |  |
| Upper bound 95%CI | 57.61 | 1.53 | 35.53 | 1.70 |  | 30.37 | 0.81 | 17.86 | 0.76 |  |
| **Denmark** | 9.81 | 0.63 | 5.16 | 0.89 | 1.40 | 4.54 | 0.28 | 2.14 | 0.38 | 1.37 |
| Lower bound 95%CI | 6.57 | 0.37 | 3.33 | 0.51 |  | 2.64 | 0.14 | 1.19 | 0.18 |  |
| Upper bound 95%CI | 13.24 | 0.96 | 7.09 | 1.37 |  | 6.69 | 0.47 | 3.23 | 0.65 |  |
| **Estonia** | 19.92 | 0.42 | 10.26 | 0.34 | 0.80 | 6.28 | 0.13 | 3.00 | 0.09 | 0.74 |
| Lower bound 95%CI | 9.84 | 0.19 | 5.12 | 0.15 |  | 1.91 | 0.03 | 0.89 | 0.02 |  |
| Upper bound 95%CI | 34.83 | 0.80 | 19.17 | 0.69 |  | 12.42 | 0.28 | 6.01 | 0.21 |  |
| **Finland** | 6.41 | 0.31 | 3.13 | 0.38 | 1.23 | 2.40 | 0.10 | 1.14 | 0.13 | 1.26 |
| Lower bound 95%CI | 2.38 | 0.10 | 1.18 | 0.13 |  | 0.31 | 0.01 | 0.14 | 0.01 |  |
| Upper bound 95%CI | 11.15 | 0.60 | 5.57 | 0.76 |  | 5.23 | 0.25 | 2.53 | 0.32 |  |
| **France** | 7.95 | 0.31 | 3.47 | 0.42 | 1.38 | 3.73 | 0.15 | 1.58 | 0.19 | 1.31 |
| Lower bound 95%CI | 5.60 | 0.20 | 2.28 | 0.25 |  | 2.31 | 0.08 | 0.95 | 0.10 |  |
| Upper bound 95%CI | 10.49 | 0.44 | 4.64 | 0.62 |  | 5.30 | 0.24 | 2.31 | 0.32 |  |
| **Germany** | 17.90 | 0.59 | 9.67 | 1.00 | 1.71 | 8.50 | 0.33 | 4.07 | 0.46 | 1.40 |
| Lower bound 95%CI | 12.87 | 0.39 | 6.54 | 0.61 |  | 5.51 | 0.19 | 2.47 | 0.24 |  |
| Upper bound 95%CI | 23.12 | 0.83 | 12.66 | 1.43 |  | 11.93 | 0.52 | 5.73 | 0.74 |  |
| **Greece** | 25.66 | 1.28 | 18.16 | 3.11 | 2.43 | 14.03 | 0.77 | 7.96 | 1.38 | 1.79 |
| Lower bound 95%CI | 18.81 | 0.84 | 12.88 | 1.92 |  | 10.02 | 0.49 | 5.47 | 0.80 |  |
| Upper bound 95%CI | 32.61 | 1.81 | 23.32 | 4.56 |  | 18.35 | 1.14 | 10.36 | 2.10 |  |
| **Hungary** | 53.70 | 1.09 | 33.59 | 1.21 | 1.10 | 29.69 | 0.65 | 18.44 | 0.69 | 1.06 |
| Lower bound 95%CI | 38.55 | 0.71 | 23.61 | 0.77 |  | 20.49 | 0.40 | 12.52 | 0.42 |  |
| Upper bound 95%CI | 72.66 | 1.62 | 47.02 | 1.86 |  | 43.18 | 1.05 | 27.55 | 1.14 |  |
| **Ireland** | 10.11 | 0.46 | 5.23 | 0.71 | 1.54 | 4.07 | 0.19 | 2.07 | 0.28 | 1.48 |
| Lower bound 95%CI | 6.34 | 0.26 | 3.22 | 0.39 |  | 2.00 | 0.08 | 0.98 | 0.11 |  |
| Upper bound 95%CI | 14.65 | 0.74 | 7.55 | 1.16 |  | 6.48 | 0.34 | 3.29 | 0.50 |  |
| **Italy** | 15.72 | 0.64 | 7.98 | 0.88 | 1.37 | 8.68 | 0.38 | 4.20 | 0.48 | 1.28 |
| Lower bound 95%CI | 11.47 | 0.41 | 5.50 | 0.53 |  | 6.03 | 0.22 | 2.74 | 0.26 |  |
| Upper bound 95%CI | 20.00 | 0.95 | 10.39 | 1.32 |  | 11.42 | 0.59 | 5.69 | 0.77 |  |
| **Latvia** | 61.42 | 1.54 | 32.84 | 1.62 | 1.06 | 28.21 | 0.67 | 14.54 | 0.62 | 0.92 |
| Lower bound 95%CI | 42.56 | 0.97 | 22.54 | 0.99 |  | 17.66 | 0.37 | 9.06 | 0.34 |  |
| Upper bound 95%CI | 86.41 | 2.37 | 46.12 | 2.58 |  | 40.97 | 1.09 | 21.87 | 1.05 |  |
| **Lithuania** | 50.51 | 1.23 | 27.73 | 1.38 | 1.12 | 24.58 | 0.59 | 13.61 | 0.59 | 1.00 |
| Lower bound 95%CI | 34.10 | 0.77 | 18.33 | 0.82 |  | 14.34 | 0.31 | 8.06 | 0.31 |  |
| Upper bound 95%CI | 68.22 | 1.78 | 37.95 | 2.09 |  | 36.09 | 0.97 | 20.16 | 0.98 |  |
| **Luxembourg** | 11.08 | 0.70 | 5.37 | 0.88 | 1.25 | 4.22 | 0.23 | 2.00 | 0.29 | 1.27 |
| Lower bound 95%CI | 7.73 | 0.43 | 3.71 | 0.51 |  | 2.29 | 0.11 | 1.04 | 0.13 |  |
| Upper bound 95%CI | 14.90 | 1.07 | 7.31 | 1.41 |  | 6.50 | 0.41 | 3.02 | 0.52 |  |
| **Malta** | 23.38 | 1.27 | 14.34 | 2.45 | 1.93 | 9.93 | 0.51 | 7.12 | 1.11 | 2.19 |
| Lower bound 95%CI | 17.04 | 0.81 | 10.05 | 1.47 |  | 6.65 | 0.30 | 4.55 | 0.62 |  |
| Upper bound 95%CI | 30.81 | 1.88 | 18.87 | 3.74 |  | 13.36 | 0.79 | 9.75 | 1.78 |  |
| **Netherlands** | 10.94 | 0.46 | 5.25 | 0.64 | 1.40 | 5.57 | 0.25 | 2.61 | 0.34 | 1.37 |
| Lower bound 95%CI | 7.87 | 0.30 | 3.52 | 0.39 |  | 3.72 | 0.14 | 1.65 | 0.19 |  |
| Upper bound 95%CI | 14.08 | 0.66 | 6.88 | 0.95 |  | 7.59 | 0.38 | 3.62 | 0.54 |  |
| **Poland** | 49.39 | 1.25 | 26.83 | 1.26 | 1.01 | 29.29 | 0.74 | 16.13 | 0.76 | 1.02 |
| Lower bound 95%CI | 37.14 | 0.82 | 19.73 | 0.80 |  | 20.91 | 0.45 | 11.31 | 0.45 |  |
| Upper bound 95%CI | 61.44 | 1.79 | 33.79 | 1.83 |  | 38.76 | 1.16 | 21.61 | 1.21 |  |
| **Portugal** | 5.48 | 0.37 | 3.20 | 0.66 | 1.76 | 2.95 | 0.20 | 1.50 | 0.28 | 1.39 |
| Lower bound 95%CI | 3.50 | 0.21 | 1.96 | 0.34 |  | 1.53 | 0.09 | 0.77 | 0.12 |  |
| Upper bound 95%CI | 7.74 | 0.61 | 4.54 | 1.09 |  | 4.62 | 0.37 | 2.41 | 0.56 |  |
| **Romania** | 46.93 | 1.21 | 31.66 | 1.44 | 1.18 | 29.48 | 0.71 | 18.99 | 0.73 | 1.02 |
| Lower bound 95%CI | 34.12 | 0.79 | 22.79 | 0.93 |  | 19.77 | 0.43 | 12.77 | 0.44 |  |
| Upper bound 95%CI | 61.90 | 1.79 | 41.81 | 2.10 |  | 41.80 | 1.15 | 27.18 | 1.16 |  |
| **Slovakia** | 59.87 | 1.62 | 38.76 | 1.96 | 1.21 | 35.23 | 0.91 | 24.24 | 1.04 | 1.14 |
| Lower bound 95%CI | 43.90 | 1.06 | 28.76 | 1.30 |  | 24.96 | 0.58 | 16.79 | 0.64 |  |
| Upper bound 95%CI | 74.73 | 2.24 | 49.71 | 2.79 |  | 47.44 | 1.39 | 32.66 | 1.57 |  |
| **Slovenia** | 17.26 | 0.50 | 8.59 | 0.43 | 0.86 | 9.50 | 0.23 | 4.03 | 0.16 | 0.68 |
| Lower bound 95%CI | 12.61 | 0.32 | 6.13 | 0.27 |  | 6.43 | 0.14 | 2.66 | 0.09 |  |
| Upper bound 95%CI | 22.21 | 0.73 | 11.08 | 0.63 |  | 12.87 | 0.37 | 5.59 | 0.25 |  |
| **Spain** | 7.72 | 0.37 | 3.63 | 0.50 | 1.37 | 4.02 | 0.21 | 1.72 | 0.25 | 1.23 |
| Lower bound 95%CI | 5.32 | 0.23 | 2.32 | 0.29 |  | 2.47 | 0.11 | 0.98 | 0.13 |  |
| Upper bound 95%CI | 10.59 | 0.56 | 4.95 | 0.77 |  | 5.84 | 0.34 | 2.56 | 0.43 |  |
| **Sweden** | 5.94 | 0.31 | 3.24 | 0.37 | 1.21 | 2.14 | 0.10 | 1.19 | 0.13 | 1.29 |
| Lower bound 95%CI | 2.96 | 0.13 | 1.59 | 0.16 |  | 0.63 | 0.03 | 0.32 | 0.03 |  |
| Upper bound 95%CI | 9.54 | 0.57 | 5.32 | 0.70 |  | 4.18 | 0.24 | 2.32 | 0.31 |  |
| **European Union** | 19.91 | 0.71 | 11.24 | 0.98 | 1.38 | 10.61 | 0.40 | 5.92 | 0.51 | 1.29 |
| Lower bound 95%CI | 14.55 | 0.47 | 8.01 | 0.62 |  | 7.35 | 0.24 | 3.89 | 0.29 |  |
| Upper bound 95%CI | 25.56 | 1.01 | 14.46 | 1.40 |  | 14.21 | 0.60 | 8.04 | 0.79 |  |

Data and definitions from Global Burden of Disease Database. 2021

*Each value corresponds to mortality rates per 100.000 inhabitants (95% Uncertainty Interval) Abbreviation: CFI, case-fatality index

| Appendix Table 10. Age-standardised mortality rates and case fatality indexes for IHD attributable to Low physical activity. 2011 , 2021 | | | | | | | | | | |
| --- | --- | --- | --- | --- | --- | --- | --- | --- | --- | --- |
| **Country** | **2011** | | | | | **2021** | | | | |
|  | **Men** | | **Women** | | **CFI Ratio** | **Men** | | **Women** | | **CFI Ratio** |
|  | **Mortality rate** | **CFI** | **Mortality rate** | **CFI** |  | **Mortality rate** | **CFI** | **Mortality rate** | **CFI** |  |
| **Austria** | 1.50 | 0.06 | 2.99 | 0.36 |  | 1.15 | 0.04 | 2.00 | 0.24 |  |
| Lower bound 95%CI | 0.42 | 0.01 | 1.03 | 0.11 | 6.32 | 0.28 | 0.01 | 0.67 | 0.07 | 5.52 |
| Upper bound 95%CI | 3.60 | 0.15 | 5.80 | 0.78 |  | 2.80 | 0.12 | 4.35 | 0.61 |  |
| **Belgium** | 1.29 | 0.06 | 1.69 | 0.22 | 3.80 | 0.88 | 0.04 | 1.03 | 0.14 | 3.38 |
| Lower bound 95%CI | 0.42 | 0.02 | 0.59 | 0.07 |  | 0.26 | 0.01 | 0.37 | 0.04 |  |
| Upper bound 95%CI | 2.96 | 0.15 | 3.19 | 0.47 |  | 2.08 | 0.11 | 2.21 | 0.33 |  |
| **Bulgaria** | 2.30 | 0.05 | 5.63 | 0.22 | 4.36 | 2.20 | 0.05 | 4.74 | 0.19 | 3.74 |
| Lower bound 95%CI | 0.75 | 0.01 | 2.15 | 0.08 |  | 0.64 | 0.01 | 1.63 | 0.06 |  |
| Upper bound 95%CI | 4.80 | 0.12 | 10.29 | 0.44 |  | 4.87 | 0.13 | 9.04 | 0.40 |  |
| **Croatia** | 2.85 | 0.08 | 6.13 | 0.36 | 4.60 | 2.27 | 0.06 | 5.00 | 0.26 | 4.37 |
| Lower bound 95%CI | 0.89 | 0.02 | 2.28 | 0.12 |  | 0.62 | 0.01 | 1.77 | 0.08 |  |
| Upper bound 95%CI | 5.79 | 0.18 | 11.25 | 0.74 |  | 5.32 | 0.16 | 9.83 | 0.58 |  |
| **Cyprus** | 3.30 | 0.21 | 3.00 | 0.61 | 2.85 | 2.07 | 0.11 | 2.20 | 0.35 | 3.06 |
| Lower bound 95%CI | 0.97 | 0.05 | 0.89 | 0.15 |  | 0.52 | 0.02 | 0.55 | 0.07 |  |
| Upper bound 95%CI | 6.98 | 0.52 | 6.30 | 1.50 |  | 4.94 | 0.32 | 5.01 | 0.93 |  |
| **Czechia** | 3.21 | 0.08 | 6.60 | 0.29 | 3.70 | 2.73 | 0.06 | 4.60 | 0.17 | 2.70 |
| Lower bound 95%CI | 1.05 | 0.02 | 2.57 | 0.10 |  | 0.83 | 0.02 | 1.85 | 0.06 |  |
| Upper bound 95%CI | 6.42 | 0.17 | 11.74 | 0.56 |  | 5.72 | 0.15 | 8.45 | 0.36 |  |
| **Denmark** | 1.01 | 0.06 | 1.62 | 0.28 | 4.27 | 0.71 | 0.04 | 0.98 | 0.17 | 3.99 |
| Lower bound 95%CI | 0.32 | 0.02 | 0.59 | 0.09 |  | 0.21 | 0.01 | 0.29 | 0.04 |  |
| Upper bound 95%CI | 2.32 | 0.17 | 3.11 | 0.60 |  | 1.77 | 0.12 | 2.08 | 0.42 |  |
| **Estonia** | 2.98 | 0.06 | 4.86 | 0.16 | 2.55 | 1.69 | 0.03 | 2.42 | 0.08 | 2.23 |
| Lower bound 95%CI | 0.88 | 0.02 | 1.73 | 0.05 |  | 0.50 | 0.01 | 0.81 | 0.02 |  |
| Upper bound 95%CI | 6.56 | 0.15 | 9.54 | 0.35 |  | 3.88 | 0.09 | 4.71 | 0.16 |  |
| **Finland** | 2.42 | 0.12 | 2.66 | 0.32 | 2.77 | 1.72 | 0.07 | 1.80 | 0.20 | 2.79 |
| Lower bound 95%CI | 0.78 | 0.03 | 1.00 | 0.11 |  | 0.49 | 0.02 | 0.57 | 0.06 |  |
| Upper bound 95%CI | 5.18 | 0.28 | 5.26 | 0.71 |  | 3.99 | 0.19 | 4.11 | 0.52 |  |
| **France** | 1.11 | 0.04 | 1.17 | 0.14 | 3.33 | 0.81 | 0.03 | 0.78 | 0.10 | 2.97 |
| Lower bound 95%CI | 0.33 | 0.01 | 0.43 | 0.05 |  | 0.23 | 0.01 | 0.26 | 0.03 |  |
| Upper bound 95%CI | 2.45 | 0.10 | 2.21 | 0.30 |  | 1.87 | 0.08 | 1.57 | 0.22 |  |
| **Germany** | 0.83 | 0.03 | 2.19 | 0.23 | 8.33 | 0.72 | 0.03 | 1.51 | 0.17 | 6.14 |
| Lower bound 95%CI | 0.24 | 0.01 | 0.78 | 0.07 |  | 0.20 | 0.01 | 0.46 | 0.05 |  |
| Upper bound 95%CI | 1.92 | 0.07 | 4.49 | 0.51 |  | 1.80 | 0.08 | 3.20 | 0.41 |  |
| **Greece** | 1.13 | 0.06 | 3.19 | 0.54 | 9.67 | 0.82 | 0.05 | 1.79 | 0.31 | 6.88 |
| Lower bound 95%CI | 0.31 | 0.01 | 1.08 | 0.16 |  | 0.24 | 0.01 | 0.59 | 0.09 |  |
| Upper bound 95%CI | 2.58 | 0.14 | 6.32 | 1.24 |  | 1.98 | 0.12 | 3.80 | 0.77 |  |
| **Hungary** | 3.54 | 0.07 | 6.18 | 0.22 | 3.08 | 2.80 | 0.06 | 4.71 | 0.18 | 2.87 |
| Lower bound 95%CI | 1.13 | 0.02 | 2.38 | 0.08 |  | 0.84 | 0.02 | 1.74 | 0.06 |  |
| Upper bound 95%CI | 7.21 | 0.16 | 11.30 | 0.45 |  | 5.90 | 0.14 | 9.00 | 0.37 |  |
| **Ireland** | 2.66 | 0.12 | 3.03 | 0.41 | 3.39 | 1.64 | 0.08 | 1.76 | 0.23 | 3.11 |
| Lower bound 95%CI | 0.83 | 0.03 | 1.18 | 0.14 |  | 0.52 | 0.02 | 0.62 | 0.07 |  |
| Upper bound 95%CI | 5.56 | 0.28 | 5.75 | 0.88 |  | 3.72 | 0.20 | 3.48 | 0.53 |  |
| **Italy** | 1.63 | 0.07 | 2.25 | 0.25 | 3.73 | 1.23 | 0.05 | 1.55 | 0.18 | 3.33 |
| Lower bound 95%CI | 0.48 | 0.02 | 0.87 | 0.08 |  | 0.39 | 0.01 | 0.60 | 0.06 |  |
| Upper bound 95%CI | 3.49 | 0.17 | 4.24 | 0.54 |  | 2.86 | 0.15 | 3.05 | 0.41 |  |
| **Latvia** | 2.97 | 0.07 | 4.34 | 0.21 | 2.89 | 2.13 | 0.05 | 2.91 | 0.12 | 2.44 |
| Lower bound 95%CI | 1.03 | 0.02 | 1.38 | 0.06 |  | 0.75 | 0.02 | 1.00 | 0.04 |  |
| Upper bound 95%CI | 6.39 | 0.18 | 8.71 | 0.49 |  | 4.43 | 0.12 | 5.73 | 0.28 |  |
| **Lithuania** | 7.20 | 0.18 | 8.43 | 0.42 | 2.39 | 5.58 | 0.13 | 6.27 | 0.27 | 2.03 |
| Lower bound 95%CI | 2.70 | 0.06 | 3.11 | 0.14 |  | 1.91 | 0.04 | 2.27 | 0.09 |  |
| Upper bound 95%CI | 14.01 | 0.37 | 16.33 | 0.90 |  | 11.55 | 0.31 | 12.29 | 0.60 |  |
| **Luxembourg** | 1.36 | 0.09 | 1.60 | 0.26 | 3.04 | 0.95 | 0.05 | 1.13 | 0.16 | 3.18 |
| Lower bound 95%CI | 0.40 | 0.02 | 0.59 | 0.08 |  | 0.25 | 0.01 | 0.36 | 0.05 |  |
| Upper bound 95%CI | 3.01 | 0.22 | 3.22 | 0.62 |  | 2.33 | 0.15 | 2.47 | 0.42 |  |
| **Malta** | 2.83 | 0.15 | 4.29 | 0.73 | 4.78 | 1.74 | 0.09 | 2.91 | 0.45 | 5.11 |
| Lower bound 95%CI | 1.00 | 0.05 | 1.67 | 0.24 |  | 0.48 | 0.02 | 1.06 | 0.14 |  |
| Upper bound 95%CI | 6.00 | 0.37 | 8.00 | 1.59 |  | 3.94 | 0.23 | 5.67 | 1.03 |  |
| **Netherlands** | 0.53 | 0.02 | 0.75 | 0.09 | 4.12 | 0.47 | 0.02 | 0.60 | 0.08 | 3.75 |
| Lower bound 95%CI | 0.17 | 0.01 | 0.24 | 0.03 |  | 0.11 | 0.00 | 0.18 | 0.02 |  |
| Upper bound 95%CI | 1.24 | 0.06 | 1.61 | 0.22 |  | 1.28 | 0.06 | 1.43 | 0.21 |  |
| **Poland** | 3.07 | 0.08 | 4.41 | 0.21 | 2.67 | 2.76 | 0.07 | 3.66 | 0.17 | 2.46 |
| Lower bound 95%CI | 1.11 | 0.02 | 1.79 | 0.07 |  | 0.93 | 0.02 | 1.38 | 0.05 |  |
| Upper bound 95%CI | 6.04 | 0.18 | 8.11 | 0.44 |  | 5.82 | 0.17 | 6.91 | 0.39 |  |
| **Portugal** | 1.07 | 0.07 | 1.38 | 0.28 | 3.89 | 0.89 | 0.06 | 1.01 | 0.19 | 3.10 |
| Lower bound 95%CI | 0.36 | 0.02 | 0.46 | 0.08 |  | 0.27 | 0.02 | 0.32 | 0.05 |  |
| Upper bound 95%CI | 2.33 | 0.18 | 2.65 | 0.64 |  | 1.86 | 0.15 | 2.11 | 0.49 |  |
| **Romania** | 2.61 | 0.07 | 5.10 | 0.23 | 3.43 | 2.37 | 0.06 | 4.22 | 0.16 | 2.81 |
| Lower bound 95%CI | 0.86 | 0.02 | 1.98 | 0.08 |  | 0.69 | 0.01 | 1.59 | 0.05 |  |
| Upper bound 95%CI | 5.13 | 0.15 | 8.84 | 0.44 |  | 4.76 | 0.13 | 7.98 | 0.34 |  |
| **Slovakia** | 3.02 | 0.08 | 5.31 | 0.27 | 3.29 | 2.53 | 0.07 | 4.49 | 0.19 | 2.95 |
| Lower bound 95%CI | 0.98 | 0.02 | 1.86 | 0.08 |  | 0.76 | 0.02 | 1.47 | 0.06 |  |
| Upper bound 95%CI | 6.49 | 0.19 | 10.40 | 0.58 |  | 5.49 | 0.16 | 9.13 | 0.44 |  |
| **Slovenia** | 1.15 | 0.03 | 1.56 | 0.08 | 2.35 | 0.92 | 0.02 | 1.03 | 0.04 | 1.80 |
| Lower bound 95%CI | 0.36 | 0.01 | 0.58 | 0.03 |  | 0.26 | 0.01 | 0.34 | 0.01 |  |
| Upper bound 95%CI | 2.44 | 0.08 | 3.15 | 0.18 |  | 1.97 | 0.06 | 2.06 | 0.09 |  |
| **Spain** | 1.12 | 0.05 | 1.20 | 0.17 | 3.11 | 0.88 | 0.05 | 0.81 | 0.12 | 2.65 |
| Lower bound 95%CI | 0.37 | 0.02 | 0.41 | 0.05 |  | 0.26 | 0.01 | 0.24 | 0.03 |  |
| Upper bound 95%CI | 2.18 | 0.11 | 2.44 | 0.38 |  | 1.88 | 0.11 | 1.76 | 0.30 |  |
| **Sweden** | 0.95 | 0.05 | 1.56 | 0.18 | 3.63 | 0.70 | 0.03 | 0.99 | 0.11 | 3.27 |
| Lower bound 95%CI | 0.35 | 0.02 | 0.63 | 0.06 |  | 0.21 | 0.01 | 0.33 | 0.03 |  |
| Upper bound 95%CI | 1.91 | 0.11 | 2.78 | 0.37 |  | 1.66 | 0.10 | 2.12 | 0.28 |  |
| **European Union** | 1.49 | 0.05 | 2.45 | 0.21 | 4.01 | 1.19 | 0.04 | 1.79 | 0.15 | 3.46 |
| Lower bound 95%CI | 0.61 | 0.02 | 1.03 | 0.08 |  | 0.50 | 0.02 | 0.75 | 0.06 |  |
| Upper bound 95%CI | 2.45 | 0.10 | 4.02 | 0.39 |  | 1.98 | 0.08 | 2.97 | 0.29 |  |

Data and definitions from Global Burden of Disease Database. 2021

*Each value corresponds to mortality rates per 100.000 inhabitants (95% Uncertainty Interval)

Abbreviation: CFI, case-fatality index

| Appendix Table 11. Age-standardised mortality rates and case fatality indexes for IHD attributable to Diet high in processed meat. 2011, 2021 | | | | | | | | | | |
| --- | --- | --- | --- | --- | --- | --- | --- | --- | --- | --- |
| **Country** | **2011** | | | | | **2021** | | | | |
|  | **Men** | | **Women** | | **CFI Ratio** | **Men** | | **Women** | | **CFI Ratio** |
|  | **Mortality rate** | **CFI** | **Mortality rate** | **CFI** |  | **Mortality rate** | **CFI** | **Mortality rate** | **CFI** |  |
| **Austria** | 2.12 | 0.08 | 1.21 | 0.15 | 1.81 | 1.39 | 0.05 | 0.79 | 0.10 | 1.81 |
| Lower bound 95%CI | 3.43 | 0.12 | 2.05 | 0.23 |  | 2.33 | 0.08 | 1.37 | 0.15 |  |
| Upper bound 95%CI | 0.80 | 0.03 | 0.41 | 0.06 |  | 0.49 | 0.02 | 0.25 | 0.03 |  |
| **Belgium** | 2.48 | 0.11 | 1.22 | 0.16 | 1.43 | 1.73 | 0.08 | 0.83 | 0.11 | 1.39 |
| Lower bound 95%CI | 3.92 | 0.16 | 1.99 | 0.24 |  | 2.84 | 0.11 | 1.38 | 0.16 |  |
| Upper bound 95%CI | 0.93 | 0.05 | 0.46 | 0.07 |  | 0.69 | 0.04 | 0.32 | 0.05 |  |
| **Bulgaria** | 3.28 | 0.07 | 1.86 | 0.07 | 1.01 | 3.64 | 0.08 | 1.99 | 0.08 | 0.95 |
| Lower bound 95%CI | 5.35 | 0.11 | 3.12 | 0.11 |  | 6.25 | 0.13 | 3.41 | 0.12 |  |
| Upper bound 95%CI | 1.24 | 0.03 | 0.73 | 0.03 |  | 1.44 | 0.04 | 0.77 | 0.03 |  |
| **Croatia** | 0.12 | 0.00 | 0.09 | 0.01 | 1.61 | 0.11 | 0.00 | 0.08 | 0.00 | 1.44 |
| Lower bound 95%CI | 0.20 | 0.00 | 0.15 | 0.01 |  | 0.18 | 0.00 | 0.13 | 0.01 |  |
| Upper bound 95%CI | 0.05 | 0.00 | 0.03 | 0.00 |  | 0.04 | 0.00 | 0.03 | 0.00 |  |
| **Cyprus** | 1.23 | 0.08 | 0.63 | 0.13 | 1.61 | 1.05 | 0.06 | 0.53 | 0.08 | 1.45 |
| Lower bound 95%CI | 2.12 | 0.12 | 1.18 | 0.20 |  | 1.80 | 0.09 | 0.99 | 0.13 |  |
| Upper bound 95%CI | 0.43 | 0.03 | 0.22 | 0.05 |  | 0.40 | 0.03 | 0.17 | 0.03 |  |
| **Czechia** | 1.61 | 0.04 | 1.01 | 0.04 | 1.13 | 1.19 | 0.03 | 0.74 | 0.03 | 1.00 |
| Lower bound 95%CI | 2.88 | 0.06 | 1.79 | 0.07 |  | 2.27 | 0.05 | 1.33 | 0.05 |  |
| Upper bound 95%CI | 0.60 | 0.02 | 0.38 | 0.02 |  | 0.41 | 0.01 | 0.27 | 0.01 |  |
| **Denmark** | 2.34 | 0.15 | 1.22 | 0.21 | 1.39 | 1.65 | 0.10 | 0.80 | 0.14 | 1.41 |
| Lower bound 95%CI | 3.68 | 0.21 | 2.02 | 0.31 |  | 2.63 | 0.14 | 1.31 | 0.20 |  |
| Upper bound 95%CI | 0.89 | 0.06 | 0.45 | 0.09 |  | 0.65 | 0.05 | 0.30 | 0.06 |  |
| **Estonia** | 21.70 | 0.46 | 8.96 | 0.30 | 0.64 | 10.49 | 0.21 | 4.09 | 0.13 | 0.61 |
| Lower bound 95%CI | 34.23 | 0.66 | 14.49 | 0.44 |  | 17.23 | 0.31 | 6.69 | 0.19 |  |
| Upper bound 95%CI | 8.76 | 0.20 | 3.54 | 0.13 |  | 4.00 | 0.09 | 1.54 | 0.05 |  |
| **Finland** | 3.87 | 0.19 | 1.68 | 0.20 | 1.09 | 1.84 | 0.08 | 0.90 | 0.10 | 1.30 |
| Lower bound 95%CI | 6.32 | 0.27 | 2.72 | 0.29 |  | 3.05 | 0.11 | 1.57 | 0.15 |  |
| Upper bound 95%CI | 1.48 | 0.08 | 0.61 | 0.08 |  | 0.67 | 0.03 | 0.33 | 0.04 |  |
| **France** | 1.98 | 0.08 | 0.79 | 0.10 | 1.26 | 1.43 | 0.06 | 0.57 | 0.07 | 1.23 |
| Lower bound 95%CI | 3.17 | 0.11 | 1.33 | 0.14 |  | 2.35 | 0.08 | 0.95 | 0.10 |  |
| Upper bound 95%CI | 0.75 | 0.03 | 0.28 | 0.04 |  | 0.54 | 0.02 | 0.21 | 0.03 |  |
| **Germany** | 5.74 | 0.19 | 2.89 | 0.30 | 1.59 | 4.09 | 0.16 | 1.93 | 0.22 | 1.38 |
| Lower bound 95%CI | 8.94 | 0.27 | 4.68 | 0.44 |  | 6.75 | 0.23 | 3.24 | 0.32 |  |
| Upper bound 95%CI | 2.25 | 0.08 | 1.12 | 0.13 |  | 1.65 | 0.07 | 0.72 | 0.09 |  |
| **Greece** | 1.41 | 0.07 | 0.79 | 0.14 | 1.92 | 1.11 | 0.06 | 0.50 | 0.09 | 1.42 |
| Lower bound 95%CI | 2.23 | 0.10 | 1.32 | 0.20 |  | 1.85 | 0.09 | 0.85 | 0.12 |  |
| Upper bound 95%CI | 0.53 | 0.03 | 0.27 | 0.05 |  | 0.43 | 0.03 | 0.18 | 0.04 |  |
| **Hungary** | 2.42 | 0.05 | 1.48 | 0.05 | 1.08 | 2.07 | 0.05 | 1.27 | 0.05 | 1.05 |
| Lower bound 95%CI | 3.89 | 0.07 | 2.35 | 0.08 |  | 3.43 | 0.07 | 2.14 | 0.07 |  |
| Upper bound 95%CI | 0.93 | 0.02 | 0.54 | 0.02 |  | 0.79 | 0.02 | 0.50 | 0.02 |  |
| **Ireland** | 2.11 | 0.10 | 1.06 | 0.14 | 1.49 | 1.19 | 0.05 | 0.63 | 0.08 | 1.54 |
| Lower bound 95%CI | 3.27 | 0.13 | 1.73 | 0.21 |  | 1.95 | 0.08 | 1.07 | 0.12 |  |
| Upper bound 95%CI | 0.80 | 0.04 | 0.39 | 0.06 |  | 0.48 | 0.03 | 0.24 | 0.04 |  |
| **Italy** | 1.84 | 0.08 | 0.91 | 0.10 | 1.34 | 1.37 | 0.06 | 0.65 | 0.07 | 1.26 |
| Lower bound 95%CI | 2.95 | 0.11 | 1.53 | 0.15 |  | 2.27 | 0.08 | 1.05 | 0.10 |  |
| Upper bound 95%CI | 0.72 | 0.03 | 0.34 | 0.04 |  | 0.54 | 0.03 | 0.23 | 0.03 |  |
| **Latvia** | 35.75 | 0.90 | 15.47 | 0.77 | 0.85 | 22.15 | 0.53 | 9.29 | 0.39 | 0.75 |
| Lower bound 95%CI | 54.98 | 1.26 | 24.47 | 1.08 |  | 35.33 | 0.75 | 14.82 | 0.55 |  |
| Upper bound 95%CI | 14.69 | 0.40 | 6.17 | 0.35 |  | 8.75 | 0.23 | 3.49 | 0.17 |  |
| **Lithuania** | 41.27 | 1.01 | 17.56 | 0.87 | 0.87 | 26.37 | 0.63 | 11.61 | 0.50 | 0.79 |
| Lower bound 95%CI | 62.93 | 1.42 | 27.35 | 1.22 |  | 41.60 | 0.90 | 18.97 | 0.73 |  |
| Upper bound 95%CI | 16.96 | 0.44 | 6.67 | 0.37 |  | 10.26 | 0.28 | 4.38 | 0.21 |  |
| **Luxembourg** | 4.98 | 0.32 | 2.15 | 0.35 | 1.11 | 2.96 | 0.16 | 1.28 | 0.19 | 1.16 |
| Lower bound 95%CI | 7.90 | 0.44 | 3.47 | 0.48 |  | 5.04 | 0.24 | 2.13 | 0.27 |  |
| Upper bound 95%CI | 1.85 | 0.13 | 0.87 | 0.17 |  | 1.08 | 0.07 | 0.48 | 0.08 |  |
| **Malta** | 7.04 | 0.38 | 3.91 | 0.67 | 1.75 | 3.89 | 0.20 | 2.59 | 0.40 | 2.03 |
| Lower bound 95%CI | 11.04 | 0.52 | 6.24 | 0.91 |  | 6.09 | 0.27 | 4.25 | 0.57 |  |
| Upper bound 95%CI | 2.72 | 0.17 | 1.47 | 0.29 |  | 1.49 | 0.09 | 0.90 | 0.16 |  |
| **Netherlands** | 1.04 | 0.04 | 0.54 | 0.07 | 1.51 | 0.80 | 0.04 | 0.41 | 0.05 | 1.50 |
| Lower bound 95%CI | 1.67 | 0.06 | 0.92 | 0.10 |  | 1.32 | 0.05 | 0.72 | 0.08 |  |
| Upper bound 95%CI | 0.40 | 0.02 | 0.20 | 0.03 |  | 0.30 | 0.02 | 0.16 | 0.02 |  |
| **Poland** | 0.88 | 0.02 | 0.44 | 0.02 | 0.93 | 0.70 | 0.02 | 0.38 | 0.02 | 1.01 |
| Lower bound 95%CI | 1.43 | 0.03 | 0.74 | 0.03 |  | 1.21 | 0.03 | 0.62 | 0.02 |  |
| Upper bound 95%CI | 0.35 | 0.01 | 0.17 | 0.01 |  | 0.26 | 0.01 | 0.14 | 0.01 |  |
| **Portugal** | 0.31 | 0.02 | 0.17 | 0.03 | 1.65 | 0.29 | 0.02 | 0.14 | 0.03 | 1.32 |
| Lower bound 95%CI | 0.50 | 0.03 | 0.30 | 0.05 |  | 0.49 | 0.03 | 0.25 | 0.04 |  |
| Upper bound 95%CI | 0.12 | 0.01 | 0.06 | 0.01 |  | 0.11 | 0.01 | 0.05 | 0.01 |  |
| **Romania** | 2.54 | 0.07 | 1.60 | 0.07 | 1.10 | 2.69 | 0.07 | 1.63 | 0.06 | 0.96 |
| Lower bound 95%CI | 3.98 | 0.09 | 2.62 | 0.11 |  | 4.53 | 0.10 | 2.71 | 0.09 |  |
| Upper bound 95%CI | 0.95 | 0.03 | 0.61 | 0.03 |  | 1.00 | 0.03 | 0.60 | 0.03 |  |
| **Slovakia** | 2.68 | 0.07 | 1.64 | 0.08 | 1.14 | 2.37 | 0.06 | 1.58 | 0.07 | 1.11 |
| Lower bound 95%CI | 4.23 | 0.10 | 2.68 | 0.12 |  | 3.98 | 0.09 | 2.78 | 0.11 |  |
| Upper bound 95%CI | 0.99 | 0.03 | 0.57 | 0.03 |  | 0.86 | 0.03 | 0.53 | 0.03 |  |
| **Slovenia** | 4.10 | 0.12 | 1.73 | 0.09 | 0.73 | 2.51 | 0.06 | 0.95 | 0.04 | 0.61 |
| Lower bound 95%CI | 6.39 | 0.16 | 2.88 | 0.13 |  | 4.08 | 0.09 | 1.64 | 0.06 |  |
| Upper bound 95%CI | 1.52 | 0.05 | 0.66 | 0.04 |  | 0.86 | 0.02 | 0.36 | 0.02 |  |
| **Spain** | 1.87 | 0.09 | 0.79 | 0.11 | 1.23 | 1.57 | 0.08 | 0.62 | 0.09 | 1.14 |
| Lower bound 95%CI | 2.94 | 0.13 | 1.32 | 0.16 |  | 2.58 | 0.12 | 1.05 | 0.14 |  |
| Upper bound 95%CI | 0.70 | 0.04 | 0.28 | 0.04 |  | 0.59 | 0.03 | 0.24 | 0.04 |  |
| **Sweden** | 5.47 | 0.28 | 2.87 | 0.33 | 1.16 | 3.42 | 0.17 | 1.82 | 0.20 | 1.23 |
| Lower bound 95%CI | 8.57 | 0.38 | 4.76 | 0.47 |  | 5.48 | 0.22 | 3.04 | 0.29 |  |
| Upper bound 95%CI | 2.15 | 0.13 | 1.10 | 0.15 |  | 1.32 | 0.08 | 0.70 | 0.09 |  |
| **European Union** | 9.78 | 0.35 | 5.64 | 0.49 | 1.41 | 4.64 | 0.17 | 3.02 | 0.26 | 1.50 |
| Lower bound 95%CI | 15.35 | 0.50 | 9.01 | 0.70 |  | 7.38 | 0.25 | 4.94 | 0.37 |  |
| Upper bound 95%CI | 3.97 | 0.16 | 2.18 | 0.21 |  | 1.84 | 0.08 | 1.13 | 0.11 |  |

Data and definitions from Global Burden of Disease Database. 2021

*Each value corresponds to mortality rates per 100.000 inhabitants (95% Uncertainty Interval)

Abbreviation: CFI, case-fatality index

| Appendix Table 12. Age-standardised mortality rates and case fatality indexes for IHD attributable to Diet high in red meat. 2011 , 2021 | | | | | | | | | | |
| --- | --- | --- | --- | --- | --- | --- | --- | --- | --- | --- |
| **Country** | **2011** | | | | | **2021** | | | | |
|  | **Men** | | **Women** | | **CFI Ratio** | **Men** | | **Women** | | **CFI Ratio** |
|  | **Mortality rate** | **CFI** | **Mortality rate** | **CFI** |  | **Mortality rate** | **CFI** | **Mortality rate** | **CFI** |  |
| **Austria** | 5.85 | 0.22 | 2.84 | 0.35 | 1.54 | 4.27 | 0.16 | 1.97 | 0.24 | 1.47 |
| Lower bound 95%CI | -0.30 | -0.01 | -0.13 | -0.01 |  | -0.23 | -0.01 | -0.09 | -0.01 |  |
| Upper bound 95%CI | 12.11 | 0.52 | 6.01 | 0.81 |  | 8.99 | 0.39 | 4.24 | 0.59 |  |
| **Belgium** | 4.10 | 0.19 | 1.68 | 0.22 | 1.19 | 2.64 | 0.12 | 1.05 | 0.14 | 1.15 |
| Lower bound 95%CI | -0.21 | -0.01 | -0.08 | -0.01 |  | -0.14 | -0.01 | -0.05 | -0.01 |  |
| Upper bound 95%CI | 8.49 | 0.43 | 3.53 | 0.52 |  | 5.52 | 0.29 | 2.27 | 0.34 |  |
| **Bulgaria** | 14.35 | 0.31 | 6.63 | 0.26 | 0.82 | 13.05 | 0.30 | 5.90 | 0.23 | 0.78 |
| Lower bound 95%CI | -0.75 | -0.01 | -0.33 | -0.01 |  | -0.69 | -0.01 | -0.32 | -0.01 |  |
| Upper bound 95%CI | 30.28 | 0.73 | 14.61 | 0.63 |  | 27.64 | 0.72 | 12.63 | 0.56 |  |
| **Croatia** | 5.22 | 0.14 | 2.39 | 0.14 | 0.98 | 3.31 | 0.09 | 1.60 | 0.08 | 0.96 |
| Lower bound 95%CI | -0.13 | 0.00 | -0.07 | 0.00 |  | -0.11 | 0.00 | -0.03 | 0.00 |  |
| Upper bound 95%CI | 11.25 | 0.35 | 5.38 | 0.36 |  | 7.25 | 0.22 | 3.99 | 0.24 |  |
| **Cyprus** | 5.79 | 0.37 | 2.26 | 0.46 | 1.23 | 3.68 | 0.20 | 1.51 | 0.24 | 1.18 |
| Lower bound 95%CI | -0.19 | -0.01 | -0.09 | -0.02 |  | -0.16 | -0.01 | -0.05 | -0.01 |  |
| Upper bound 95%CI | 12.22 | 0.90 | 5.35 | 1.28 |  | 8.24 | 0.53 | 3.54 | 0.66 |  |
| **Czechia** | 8.37 | 0.20 | 4.10 | 0.18 | 0.88 | 6.13 | 0.15 | 2.98 | 0.11 | 0.78 |
| Lower bound 95%CI | -0.40 | -0.01 | -0.11 | 0.00 |  | -0.32 | -0.01 | -0.10 | 0.00 |  |
| Upper bound 95%CI | 17.72 | 0.47 | 9.09 | 0.44 |  | 13.68 | 0.37 | 6.55 | 0.28 |  |
| **Denmark** | 3.88 | 0.25 | 1.73 | 0.30 | 1.19 | 2.66 | 0.16 | 1.08 | 0.19 | 1.18 |
| Lower bound 95%CI | -0.18 | -0.01 | -0.09 | -0.01 |  | -0.13 | -0.01 | -0.05 | -0.01 |  |
| Upper bound 95%CI | 7.93 | 0.57 | 3.76 | 0.73 |  | 5.49 | 0.38 | 2.31 | 0.47 |  |
| **Estonia** | 6.64 | 0.14 | 2.37 | 0.08 | 0.56 | 3.78 | 0.08 | 1.28 | 0.04 | 0.53 |
| Lower bound 95%CI | -0.19 | 0.00 | -0.06 | 0.00 |  | -0.13 | 0.00 | -0.03 | 0.00 |  |
| Upper bound 95%CI | 14.24 | 0.33 | 5.42 | 0.20 |  | 8.11 | 0.18 | 3.04 | 0.10 |  |
| **Finland** | 6.32 | 0.30 | 2.44 | 0.29 | 0.97 | 4.43 | 0.18 | 1.72 | 0.19 | 1.03 |
| Lower bound 95%CI | -0.27 | -0.01 | -0.10 | -0.01 |  | -0.20 | -0.01 | -0.07 | -0.01 |  |
| Upper bound 95%CI | 13.09 | 0.70 | 5.56 | 0.75 |  | 9.24 | 0.44 | 3.82 | 0.48 |  |
| **France** | 3.33 | 0.13 | 1.18 | 0.14 | 1.12 | 2.37 | 0.09 | 0.83 | 0.10 | 1.08 |
| Lower bound 95%CI | -0.17 | -0.01 | -0.06 | -0.01 |  | -0.12 | 0.00 | -0.04 | 0.00 |  |
| Upper bound 95%CI | 6.70 | 0.28 | 2.51 | 0.34 |  | 4.84 | 0.22 | 1.75 | 0.24 |  |
| **Germany** | 5.33 | 0.17 | 2.39 | 0.25 | 1.42 | 4.22 | 0.16 | 1.71 | 0.19 | 1.19 |
| Lower bound 95%CI | -0.27 | -0.01 | -0.11 | -0.01 |  | -0.21 | -0.01 | -0.08 | -0.01 |  |
| Upper bound 95%CI | 10.95 | 0.39 | 5.25 | 0.59 |  | 8.60 | 0.38 | 3.69 | 0.47 |  |
| **Greece** | 7.70 | 0.38 | 3.96 | 0.68 | 1.76 | 5.59 | 0.31 | 2.33 | 0.40 | 1.31 |
| Lower bound 95%CI | -0.42 | -0.02 | -0.17 | -0.03 |  | -0.29 | -0.01 | -0.11 | -0.02 |  |
| Upper bound 95%CI | 15.52 | 0.86 | 8.41 | 1.65 |  | 11.16 | 0.70 | 4.89 | 0.99 |  |
| **Hungary** | 8.39 | 0.17 | 3.88 | 0.14 | 0.82 | 6.47 | 0.14 | 3.07 | 0.11 | 0.81 |
| Lower bound 95%CI | -0.23 | 0.00 | -0.08 | 0.00 |  | -0.24 | 0.00 | -0.07 | 0.00 |  |
| Upper bound 95%CI | 18.64 | 0.42 | 8.23 | 0.33 |  | 14.21 | 0.35 | 6.92 | 0.29 |  |
| **Ireland** | 6.53 | 0.30 | 2.83 | 0.38 | 1.29 | 4.00 | 0.18 | 1.72 | 0.23 | 1.25 |
| Lower bound 95%CI | -0.35 | -0.01 | -0.14 | -0.02 |  | -0.21 | -0.01 | -0.09 | -0.01 |  |
| Upper bound 95%CI | 13.52 | 0.68 | 5.90 | 0.90 |  | 8.30 | 0.44 | 3.64 | 0.55 |  |
| **Italy** | 3.50 | 0.14 | 1.48 | 0.16 | 1.14 | 2.51 | 0.11 | 1.03 | 0.12 | 1.09 |
| Lower bound 95%CI | -0.19 | -0.01 | -0.07 | -0.01 |  | -0.13 | 0.00 | -0.05 | 0.00 |  |
| Upper bound 95%CI | 7.38 | 0.35 | 3.16 | 0.40 |  | 5.44 | 0.28 | 2.18 | 0.30 |  |
| **Latvia** | 9.65 | 0.24 | 3.67 | 0.18 | 0.75 | 6.83 | 0.16 | 2.55 | 0.11 | 0.67 |
| Lower bound 95%CI | -0.24 | -0.01 | -0.14 | -0.01 |  | -0.33 | -0.01 | -0.08 | 0.00 |  |
| Upper bound 95%CI | 20.75 | 0.57 | 8.42 | 0.47 |  | 16.04 | 0.43 | 5.84 | 0.28 |  |
| **Lithuania** | 16.48 | 0.40 | 6.79 | 0.34 | 0.84 | 11.15 | 0.27 | 4.73 | 0.20 | 0.76 |
| Lower bound 95%CI | -0.82 | -0.02 | -0.28 | -0.01 |  | -0.48 | -0.01 | -0.17 | -0.01 |  |
| Upper bound 95%CI | 34.72 | 0.91 | 14.82 | 0.82 |  | 23.65 | 0.64 | 10.10 | 0.49 |  |
| **Luxembourg** | 4.61 | 0.29 | 1.93 | 0.32 | 1.08 | 3.22 | 0.18 | 1.33 | 0.19 | 1.11 |
| Lower bound 95%CI | -0.23 | -0.01 | -0.10 | -0.01 |  | -0.17 | -0.01 | -0.06 | -0.01 |  |
| Upper bound 95%CI | 9.55 | 0.69 | 4.05 | 0.78 |  | 6.59 | 0.41 | 2.78 | 0.48 |  |
| **Malta** | 5.25 | 0.28 | 2.58 | 0.44 | 1.55 | 3.21 | 0.16 | 1.88 | 0.29 | 1.79 |
| Lower bound 95%CI | -0.22 | -0.01 | -0.07 | -0.01 |  | -0.16 | -0.01 | -0.07 | -0.01 |  |
| Upper bound 95%CI | 10.95 | 0.67 | 5.69 | 1.13 |  | 6.89 | 0.41 | 4.14 | 0.75 |  |
| **Netherlands** | 3.38 | 0.14 | 1.40 | 0.17 | 1.21 | 2.42 | 0.11 | 1.00 | 0.13 | 1.21 |
| Lower bound 95%CI | -0.17 | -0.01 | -0.07 | -0.01 |  | -0.12 | 0.00 | -0.05 | -0.01 |  |
| Upper bound 95%CI | 6.81 | 0.32 | 2.94 | 0.40 |  | 5.02 | 0.25 | 2.19 | 0.32 |  |
| **Poland** | 8.05 | 0.20 | 3.32 | 0.16 | 0.76 | 6.66 | 0.17 | 2.88 | 0.14 | 0.80 |
| Lower bound 95%CI | -0.39 | -0.01 | -0.16 | -0.01 |  | -0.33 | -0.01 | -0.14 | -0.01 |  |
| Upper bound 95%CI | 16.64 | 0.48 | 7.23 | 0.39 |  | 13.97 | 0.42 | 6.28 | 0.35 |  |
| **Portugal** | 3.34 | 0.23 | 1.52 | 0.31 | 1.37 | 2.96 | 0.20 | 1.15 | 0.22 | 1.06 |
| Lower bound 95%CI | -0.19 | -0.01 | -0.07 | -0.01 |  | -0.16 | -0.01 | -0.06 | -0.01 |  |
| Upper bound 95%CI | 6.78 | 0.53 | 3.22 | 0.77 |  | 5.98 | 0.48 | 2.47 | 0.57 |  |
| **Romania** | 10.75 | 0.28 | 5.55 | 0.25 | 0.91 | 9.32 | 0.23 | 4.68 | 0.18 | 0.79 |
| Lower bound 95%CI | -0.56 | -0.01 | -0.26 | -0.01 |  | -0.48 | -0.01 | -0.22 | -0.01 |  |
| Upper bound 95%CI | 22.21 | 0.64 | 11.70 | 0.59 |  | 19.80 | 0.54 | 9.85 | 0.42 |  |
| **Slovakia** | 9.15 | 0.25 | 4.36 | 0.22 | 0.89 | 7.40 | 0.19 | 3.93 | 0.17 | 0.88 |
| Lower bound 95%CI | -0.30 | -0.01 | -0.14 | -0.01 |  | -0.28 | -0.01 | -0.15 | -0.01 |  |
| Upper bound 95%CI | 19.36 | 0.58 | 10.18 | 0.57 |  | 15.91 | 0.47 | 8.92 | 0.43 |  |
| **Slovenia** | 3.99 | 0.12 | 1.58 | 0.08 | 0.68 | 2.80 | 0.07 | 0.97 | 0.04 | 0.55 |
| Lower bound 95%CI | -0.17 | 0.00 | -0.08 | 0.00 |  | -0.15 | 0.00 | -0.05 | 0.00 |  |
| Upper bound 95%CI | 8.34 | 0.27 | 3.49 | 0.20 |  | 5.95 | 0.17 | 2.18 | 0.10 |  |
| **Spain** | 3.31 | 0.16 | 1.21 | 0.17 | 1.06 | 2.54 | 0.13 | 0.87 | 0.13 | 0.99 |
| Lower bound 95%CI | -0.18 | -0.01 | -0.06 | -0.01 |  | -0.13 | -0.01 | -0.04 | -0.01 |  |
| Upper bound 95%CI | 6.82 | 0.36 | 2.62 | 0.41 |  | 5.36 | 0.31 | 1.88 | 0.32 |  |
| **Sweden** | 4.57 | 0.24 | 2.10 | 0.24 | 1.02 | 2.77 | 0.13 | 1.32 | 0.15 | 1.10 |
| Lower bound 95%CI | -0.24 | -0.01 | -0.10 | -0.01 |  | -0.14 | -0.01 | -0.05 | 0.00 |  |
| Upper bound 95%CI | 9.66 | 0.57 | 4.52 | 0.60 |  | 5.92 | 0.34 | 2.92 | 0.39 |  |
| **European Union** | 11.54 | 0.41 | 5.39 | 0.47 | 1.14 | 8.56 | 0.32 | 4.23 | 0.36 | 1.14 |
| Lower bound 95%CI | -0.53 | -0.02 | -0.22 | -0.02 |  | -0.41 | -0.01 | -0.18 | -0.01 |  |
| Upper bound 95%CI | 23.89 | 0.94 | 11.31 | 1.10 |  | 17.73 | 0.75 | 8.73 | 0.85 |  |

Data and definitions from Global Burden of Disease Database. 2021

*Each value corresponds to mortality rates per 100.000 inhabitants (95% Uncertainty Interval)

Abbreviation: CFI, case-fatality index

| Appendix Table 13. Age-standardised mortality rates and case fatality indexes for IHD attributable to Diet high in sodium. 2011 , 2021 | | | | | | | | | | |
| --- | --- | --- | --- | --- | --- | --- | --- | --- | --- | --- |
| **Country** | **2011** | | | | | **2021** | | | | |
|  | **Men** | | **Women** | |  | **Men** | | **Women** | |  |
|  | **Mortality rate** | **CFI** | **Mortality rate** | **CFI** | **CFI Ratio** | **Mortality rate** | **CFI** | **Mortality rate** | **CFI** | **CFI Ratio** |
| **Austria** | 10.81 | 0.42 | 3.60 | 0.44 | 1.06 | 7.98 | 0.31 | 2.49 | 0.30 | 0.99 |
| Lower bound 95%CI | 0.50 | 0.02 | 0.02 | 0.00 |  | 0.41 | 0.01 | 0.01 | 0.00 |  |
| Upper bound 95%CI | 26.49 | 1.13 | 11.58 | 1.57 |  | 19.85 | 0.86 | 7.74 | 1.08 |  |
| **Belgium** | 5.75 | 0.26 | 1.87 | 0.25 | 0.94 | 3.79 | 0.17 | 1.14 | 0.15 | 0.87 |
| Lower bound 95%CI | 0.17 | 0.01 | 0.01 | 0.00 |  | 0.10 | 0.00 | 0.00 | 0.00 |  |
| Upper bound 95%CI | 15.40 | 0.78 | 6.22 | 0.92 |  | 10.11 | 0.53 | 3.85 | 0.58 |  |
| **Bulgaria** | 52.70 | 1.15 | 23.47 | 0.91 | 0.79 | 46.67 | 1.07 | 19.40 | 0.77 | 0.72 |
| Lower bound 95%CI | 17.96 | 0.36 | 4.38 | 0.16 |  | 16.24 | 0.33 | 3.56 | 0.13 |  |
| Upper bound 95%CI | 94.70 | 2.29 | 47.59 | 2.04 |  | 84.25 | 2.18 | 39.73 | 1.77 |  |
| **Croatia** | 35.54 | 0.96 | 17.59 | 1.02 | 1.06 | 27.63 | 0.72 | 14.08 | 0.73 | 1.01 |
| Lower bound 95%CI | 12.22 | 0.29 | 3.28 | 0.17 |  | 9.60 | 0.22 | 2.68 | 0.12 |  |
| Upper bound 95%CI | 62.77 | 1.93 | 34.81 | 2.30 |  | 49.27 | 1.48 | 28.31 | 1.67 |  |
| **Cyprus** | 8.19 | 0.53 | 2.26 | 0.46 | 0.87 | 5.79 | 0.32 | 1.64 | 0.26 | 0.81 |
| Lower bound 95%CI | 0.06 | 0.00 | 0.00 | 0.00 |  | 0.06 | 0.00 | 0.00 | 0.00 |  |
| Upper bound 95%CI | 26.46 | 1.95 | 9.42 | 2.25 |  | 18.47 | 1.18 | 6.87 | 1.27 |  |
| **Czechia** | 39.79 | 0.96 | 17.88 | 0.77 | 0.81 | 29.08 | 0.69 | 12.39 | 0.47 | 0.68 |
| Lower bound 95%CI | 13.76 | 0.30 | 3.24 | 0.13 |  | 9.99 | 0.21 | 2.09 | 0.07 |  |
| Upper bound 95%CI | 70.96 | 1.89 | 37.13 | 1.78 |  | 53.48 | 1.43 | 25.97 | 1.11 |  |
| **Denmark** | 4.17 | 0.27 | 1.19 | 0.20 | 0.76 | 2.94 | 0.18 | 0.74 | 0.13 | 0.73 |
| Lower bound 95%CI | 0.04 | 0.00 | 0.00 | 0.00 |  | 0.02 | 0.00 | 0.00 | 0.00 |  |
| Upper bound 95%CI | 12.89 | 0.93 | 5.04 | 0.97 |  | 8.97 | 0.62 | 3.05 | 0.62 |  |
| **Estonia** | 7.12 | 0.15 | 1.16 | 0.04 | 0.25 | 3.83 | 0.08 | 0.57 | 0.02 | 0.23 |
| Lower bound 95%CI | 0.00 | 0.00 | 0.00 | 0.00 |  | 0.00 | 0.00 | 0.00 | 0.00 |  |
| Upper bound 95%CI | 29.00 | 0.67 | 7.70 | 0.28 |  | 16.38 | 0.37 | 3.85 | 0.13 |  |
| **Finland** | 8.61 | 0.41 | 1.65 | 0.20 | 0.48 | 6.13 | 0.25 | 1.15 | 0.13 | 0.50 |
| Lower bound 95%CI | 0.11 | 0.00 | 0.00 | 0.00 |  | 0.08 | 0.00 | 0.00 | 0.00 |  |
| Upper bound 95%CI | 25.79 | 1.38 | 7.75 | 1.05 |  | 18.84 | 0.89 | 5.25 | 0.67 |  |
| **France** | 2.67 | 0.10 | 0.69 | 0.08 | 0.82 | 1.98 | 0.08 | 0.48 | 0.06 | 0.75 |
| Lower bound 95%CI | 0.01 | 0.00 | 0.00 | 0.00 |  | 0.01 | 0.00 | 0.00 | 0.00 |  |
| Upper bound 95%CI | 8.88 | 0.37 | 3.05 | 0.41 |  | 6.62 | 0.29 | 2.10 | 0.29 |  |
| **Germany** | 6.18 | 0.20 | 1.51 | 0.16 | 0.77 | 5.00 | 0.19 | 1.07 | 0.12 | 0.63 |
| Lower bound 95%CI | 0.10 | 0.00 | 0.00 | 0.00 |  | 0.09 | 0.00 | 0.00 | 0.00 |  |
| Upper bound 95%CI | 19.47 | 0.70 | 6.61 | 0.75 |  | 15.18 | 0.66 | 4.64 | 0.60 |  |
| **Greece** | 6.25 | 0.31 | 2.38 | 0.41 | 1.31 | 4.80 | 0.26 | 1.41 | 0.24 | 0.92 |
| Lower bound 95%CI | 0.06 | 0.00 | 0.00 | 0.00 |  | 0.05 | 0.00 | 0.00 | 0.00 |  |
| Upper bound 95%CI | 19.70 | 1.09 | 10.10 | 1.98 |  | 15.07 | 0.94 | 5.97 | 1.21 |  |
| **Hungary** | 50.29 | 1.03 | 21.47 | 0.77 | 0.75 | 38.89 | 0.85 | 16.29 | 0.61 | 0.71 |
| Lower bound 95%CI | 20.96 | 0.39 | 4.51 | 0.15 |  | 15.94 | 0.31 | 3.46 | 0.11 |  |
| Upper bound 95%CI | 85.02 | 1.90 | 41.42 | 1.64 |  | 66.72 | 1.63 | 31.85 | 1.32 |  |
| **Ireland** | 4.51 | 0.21 | 0.90 | 0.12 | 0.59 | 2.76 | 0.13 | 0.54 | 0.07 | 0.57 |
| Lower bound 95%CI | 0.01 | 0.00 | 0.00 | 0.00 |  | 0.01 | 0.00 | 0.00 | 0.00 |  |
| Upper bound 95%CI | 16.12 | 0.81 | 4.80 | 0.74 |  | 9.79 | 0.52 | 2.86 | 0.44 |  |
| **Italy** | 5.03 | 0.21 | 1.36 | 0.15 | 0.73 | 3.82 | 0.17 | 1.00 | 0.11 | 0.69 |
| Lower bound 95%CI | 0.25 | 0.01 | 0.00 | 0.00 |  | 0.18 | 0.01 | 0.00 | 0.00 |  |
| Upper bound 95%CI | 14.43 | 0.68 | 5.35 | 0.68 |  | 10.46 | 0.54 | 3.85 | 0.52 |  |
| **Latvia** | 23.22 | 0.58 | 3.14 | 0.16 | 0.27 | 15.37 | 0.36 | 2.03 | 0.09 | 0.24 |
| Lower bound 95%CI | 0.57 | 0.01 | 0.00 | 0.00 |  | 0.32 | 0.01 | 0.00 | 0.00 |  |
| Upper bound 95%CI | 62.82 | 1.72 | 15.78 | 0.88 |  | 42.47 | 1.13 | 10.43 | 0.50 |  |
| **Lithuania** | 16.64 | 0.41 | 7.81 | 0.39 | 0.96 | 11.75 | 0.28 | 5.64 | 0.24 | 0.87 |
| Lower bound 95%CI | 0.04 | 0.00 | 0.00 | 0.00 |  | 0.02 | 0.00 | 0.00 | 0.00 |  |
| Upper bound 95%CI | 57.09 | 1.49 | 28.33 | 1.56 |  | 39.51 | 1.06 | 20.40 | 0.99 |  |
| **Luxembourg** | 4.41 | 0.28 | 1.16 | 0.19 | 0.68 | 3.18 | 0.17 | 0.81 | 0.12 | 0.68 |
| Lower bound 95%CI | 0.03 | 0.00 | 0.00 | 0.00 |  | 0.02 | 0.00 | 0.00 | 0.00 |  |
| Upper bound 95%CI | 14.16 | 1.02 | 4.89 | 0.94 |  | 10.31 | 0.64 | 3.51 | 0.60 |  |
| **Malta** | 8.17 | 0.44 | 2.73 | 0.47 | 1.05 | 5.18 | 0.26 | 2.06 | 0.32 | 1.22 |
| Lower bound 95%CI | 0.27 | 0.01 | 0.01 | 0.00 |  | 0.22 | 0.01 | 0.01 | 0.00 |  |
| Upper bound 95%CI | 24.01 | 1.47 | 10.97 | 2.18 |  | 14.35 | 0.85 | 7.72 | 1.41 |  |
| **Netherlands** | 3.07 | 0.13 | 0.81 | 0.10 | 0.77 | 2.39 | 0.11 | 0.62 | 0.08 | 0.76 |
| Lower bound 95%CI | 0.02 | 0.00 | 0.00 | 0.00 |  | 0.02 | 0.00 | 0.00 | 0.00 |  |
| Upper bound 95%CI | 10.17 | 0.47 | 3.58 | 0.49 |  | 8.12 | 0.41 | 2.67 | 0.40 |  |
| **Poland** | 24.70 | 0.62 | 9.64 | 0.45 | 0.72 | 20.94 | 0.53 | 7.85 | 0.37 | 0.69 |
| Lower bound 95%CI | 5.07 | 0.11 | 0.92 | 0.04 |  | 4.67 | 0.10 | 0.70 | 0.03 |  |
| Upper bound 95%CI | 50.39 | 1.47 | 22.50 | 1.22 |  | 41.66 | 1.24 | 18.61 | 1.04 |  |
| **Portugal** | 2.56 | 0.17 | 0.63 | 0.13 | 0.74 | 2.55 | 0.18 | 0.52 | 0.10 | 0.56 |
| Lower bound 95%CI | 0.06 | 0.00 | 0.00 | 0.00 |  | 0.08 | 0.00 | 0.00 | 0.00 |  |
| Upper bound 95%CI | 8.29 | 0.65 | 3.12 | 0.75 |  | 7.79 | 0.62 | 2.41 | 0.56 |  |
| **Romania** | 41.02 | 1.06 | 19.66 | 0.89 | 0.84 | 34.41 | 0.83 | 15.43 | 0.59 | 0.71 |
| Lower bound 95%CI | 14.54 | 0.34 | 3.78 | 0.15 |  | 12.06 | 0.26 | 2.88 | 0.10 |  |
| Upper bound 95%CI | 72.56 | 2.10 | 39.83 | 2.00 |  | 61.02 | 1.68 | 32.27 | 1.38 |  |
| **Slovakia** | 51.32 | 1.39 | 24.00 | 1.21 | 0.87 | 40.11 | 1.04 | 19.82 | 0.85 | 0.82 |
| Lower bound 95%CI | 17.60 | 0.42 | 4.86 | 0.22 |  | 14.02 | 0.33 | 3.95 | 0.15 |  |
| Upper bound 95%CI | 91.28 | 2.74 | 48.13 | 2.70 |  | 72.50 | 2.13 | 41.22 | 1.99 |  |
| **Slovenia** | 16.41 | 0.48 | 5.94 | 0.30 | 0.63 | 12.40 | 0.31 | 3.82 | 0.15 | 0.49 |
| Lower bound 95%CI | 5.71 | 0.15 | 1.12 | 0.05 |  | 4.25 | 0.09 | 0.71 | 0.02 |  |
| Upper bound 95%CI | 29.19 | 0.96 | 12.26 | 0.70 |  | 22.04 | 0.63 | 7.89 | 0.35 |  |
| **Spain** | 1.54 | 0.07 | 0.42 | 0.06 | 0.79 | 1.30 | 0.07 | 0.30 | 0.04 | 0.66 |
| Lower bound 95%CI | 0.02 | 0.00 | 0.00 | 0.00 |  | 0.02 | 0.00 | 0.00 | 0.00 |  |
| Upper bound 95%CI | 6.42 | 0.34 | 2.40 | 0.37 |  | 4.99 | 0.29 | 1.59 | 0.27 |  |
| **Sweden** | 5.91 | 0.30 | 1.59 | 0.18 | 0.60 | 3.64 | 0.18 | 0.98 | 0.11 | 0.62 |
| Lower bound 95%CI | 0.04 | 0.00 | 0.00 | 0.00 |  | 0.03 | 0.00 | 0.00 | 0.00 |  |
| Upper bound 95%CI | 17.86 | 1.06 | 6.85 | 0.91 |  | 11.22 | 0.64 | 4.19 | 0.55 |  |
| **European Union** | 33.37 | 1.19 | 11.24 | 0.98 | 0.82 | 25.23 | 0.94 | 8.83 | 0.76 | 0.81 |
| Lower bound 95%CI | 6.55 | 0.21 | 1.11 | 0.09 |  | 5.12 | 0.17 | 0.88 | 0.07 |  |
| Upper bound 95%CI | 75.55 | 2.98 | 32.60 | 3.16 |  | 57.14 | 2.42 | 25.58 | 2.50 |  |

Data and definitions from Global Burden of Disease Database. 2021

*Each value corresponds to mortality rates per 100.000 inhabitants (95% Uncertainty Interval)

Abbreviation: CFI, case-fatality index

| Appendix Table 14. Age-standardised mortality rates and case fatality indexes for IHD attributable to Diet high in sugar-sweetened beverages. 2011 , 2021 | | | | | | | | | | |
| --- | --- | --- | --- | --- | --- | --- | --- | --- | --- | --- |
| **Country** | **2011** | | | | | **2021** | | | | |
|  | **Men** | | **Women** | | **CFI Ratio** | **Men** | | **Women** | | **CFI Ratio** |
|  | **Mortality rate** | **CFI** | **Mortality rate** | **CFI** |  | **Mortality rate** | **CFI** | **Mortality rate** | **CFI** |  |
| **Austria** | 0.14 | 0.01 | 0.08 | 0.01 | 1.81 | 0.10 | 0.00 | 0.05 | 0.01 | 1.59 |
| Lower bound 95%CI | -0.04 | 0.00 | -0.02 | 0.00 |  | -0.03 | 0.00 | -0.01 | 0.00 |  |
| Upper bound 95%CI | 0.31 | 0.01 | 0.17 | 0.02 |  | 0.23 | 0.01 | 0.12 | 0.02 |  |
| **Belgium** | 0.12 | 0.01 | 0.05 | 0.01 | 1.21 | 0.08 | 0.00 | 0.04 | 0.01 | 1.45 |
| Lower bound 95%CI | -0.03 | 0.00 | -0.01 | 0.00 |  | -0.02 | 0.00 | -0.01 | 0.00 |  |
| Upper bound 95%CI | 0.26 | 0.01 | 0.12 | 0.02 |  | 0.19 | 0.01 | 0.08 | 0.01 |  |
| **Bulgaria** | 0.36 | 0.01 | 0.19 | 0.01 | 0.94 | 0.41 | 0.01 | 0.20 | 0.01 | 0.85 |
| Lower bound 95%CI | -0.09 | 0.00 | -0.05 | 0.00 |  | -0.11 | 0.00 | -0.05 | 0.00 |  |
| Upper bound 95%CI | 0.79 | 0.02 | 0.40 | 0.02 |  | 0.93 | 0.02 | 0.44 | 0.02 |  |
| **Croatia** | 0.29 | 0.01 | 0.16 | 0.01 | 1.18 | 0.21 | 0.01 | 0.12 | 0.01 | 1.13 |
| Lower bound 95%CI | -0.07 | 0.00 | -0.04 | 0.00 |  | -0.05 | 0.00 | -0.03 | 0.00 |  |
| Upper bound 95%CI | 0.64 | 0.02 | 0.35 | 0.02 |  | 0.47 | 0.01 | 0.26 | 0.02 |  |
| **Cyprus** | 0.15 | 0.01 | 0.06 | 0.01 | 1.26 | 0.10 | 0.01 | 0.04 | 0.01 | 1.15 |
| Lower bound 95%CI | -0.03 | 0.00 | -0.01 | 0.00 |  | -0.02 | 0.00 | -0.01 | 0.00 |  |
| Upper bound 95%CI | 0.32 | 0.02 | 0.14 | 0.03 |  | 0.23 | 0.01 | 0.10 | 0.02 |  |
| **Czechia** | 0.25 | 0.01 | 0.14 | 0.01 | 1.01 | 0.19 | 0.00 | 0.10 | 0.00 | 0.84 |
| Lower bound 95%CI | -0.06 | 0.00 | -0.03 | 0.00 |  | -0.04 | 0.00 | -0.02 | 0.00 |  |
| Upper bound 95%CI | 0.56 | 0.01 | 0.30 | 0.01 |  | 0.43 | 0.01 | 0.23 | 0.01 |  |
| **Denmark** | 0.09 | 0.01 | 0.05 | 0.01 | 1.48 | 0.07 | 0.00 | 0.03 | 0.01 | 1.24 |
| Lower bound 95%CI | -0.02 | 0.00 | -0.01 | 0.00 |  | -0.02 | 0.00 | -0.01 | 0.00 |  |
| Upper bound 95%CI | 0.20 | 0.01 | 0.10 | 0.02 |  | 0.15 | 0.01 | 0.07 | 0.01 |  |
| **Estonia** | 0.20 | 0.00 | 0.09 | 0.00 | 0.70 | 0.11 | 0.00 | 0.05 | 0.00 | 0.71 |
| Lower bound 95%CI | -0.05 | 0.00 | -0.02 | 0.00 |  | -0.03 | 0.00 | -0.01 | 0.00 |  |
| Upper bound 95%CI | 0.44 | 0.01 | 0.21 | 0.01 |  | 0.25 | 0.01 | 0.11 | 0.00 |  |
| **Finland** | 0.16 | 0.01 | 0.07 | 0.01 | 1.10 | 0.12 | 0.00 | 0.05 | 0.01 | 1.11 |
| Lower bound 95%CI | -0.04 | 0.00 | -0.02 | 0.00 |  | -0.03 | 0.00 | -0.01 | 0.00 |  |
| Upper bound 95%CI | 0.35 | 0.02 | 0.16 | 0.02 |  | 0.26 | 0.01 | 0.11 | 0.01 |  |
| **France** | 0.06 | 0.00 | 0.03 | 0.00 | 1.58 | 0.05 | 0.00 | 0.02 | 0.00 | 1.24 |
| Lower bound 95%CI | -0.02 | 0.00 | -0.01 | 0.00 |  | -0.01 | 0.00 | 0.00 | 0.00 |  |
| Upper bound 95%CI | 0.14 | 0.01 | 0.05 | 0.01 |  | 0.11 | 0.00 | 0.04 | 0.01 |  |
| **Germany** | 0.15 | 0.00 | 0.07 | 0.01 | 1.47 | 0.12 | 0.00 | 0.05 | 0.01 | 1.22 |
| Lower bound 95%CI | -0.04 | 0.00 | -0.02 | 0.00 |  | -0.03 | 0.00 | -0.01 | 0.00 |  |
| Upper bound 95%CI | 0.33 | 0.01 | 0.16 | 0.02 |  | 0.26 | 0.01 | 0.12 | 0.02 |  |
| **Greece** | 0.16 | 0.01 | 0.09 | 0.02 | 1.93 | 0.12 | 0.01 | 0.05 | 0.01 | 1.31 |
| Lower bound 95%CI | -0.04 | 0.00 | -0.02 | 0.00 |  | -0.03 | 0.00 | -0.01 | 0.00 |  |
| Upper bound 95%CI | 0.36 | 0.02 | 0.20 | 0.04 |  | 0.27 | 0.02 | 0.12 | 0.02 |  |
| **Hungary** | 0.29 | 0.01 | 0.16 | 0.01 | 0.97 | 0.25 | 0.01 | 0.13 | 0.00 | 0.89 |
| Lower bound 95%CI | -0.08 | 0.00 | -0.04 | 0.00 |  | -0.06 | 0.00 | -0.03 | 0.00 |  |
| Upper bound 95%CI | 0.63 | 0.01 | 0.35 | 0.01 |  | 0.57 | 0.01 | 0.30 | 0.01 |  |
| **Ireland** | 0.17 | 0.01 | 0.08 | 0.01 | 1.40 | 0.13 | 0.01 | 0.06 | 0.01 | 1.34 |
| Lower bound 95%CI | -0.04 | 0.00 | -0.02 | 0.00 |  | -0.03 | 0.00 | -0.01 | 0.00 |  |
| Upper bound 95%CI | 0.37 | 0.02 | 0.17 | 0.03 |  | 0.28 | 0.01 | 0.13 | 0.02 |  |
| **Italy** | 0.08 | 0.00 | 0.03 | 0.00 | 1.01 | 0.06 | 0.00 | 0.02 | 0.00 | 0.88 |
| Lower bound 95%CI | -0.02 | 0.00 | -0.01 | 0.00 |  | -0.01 | 0.00 | -0.01 | 0.00 |  |
| Upper bound 95%CI | 0.17 | 0.01 | 0.08 | 0.01 |  | 0.13 | 0.01 | 0.06 | 0.01 |  |
| **Latvia** | 0.18 | 0.00 | 0.08 | 0.00 | 0.88 | 0.14 | 0.00 | 0.06 | 0.00 | 0.76 |
| Lower bound 95%CI | -0.04 | 0.00 | -0.02 | 0.00 |  | -0.03 | 0.00 | -0.01 | 0.00 |  |
| Upper bound 95%CI | 0.39 | 0.01 | 0.20 | 0.01 |  | 0.31 | 0.01 | 0.15 | 0.01 |  |
| **Lithuania** | 0.30 | 0.01 | 0.14 | 0.01 | 0.95 | 0.23 | 0.01 | 0.11 | 0.00 | 0.86 |
| Lower bound 95%CI | -0.08 | 0.00 | -0.03 | 0.00 |  | -0.06 | 0.00 | -0.02 | 0.00 |  |
| Upper bound 95%CI | 0.65 | 0.02 | 0.31 | 0.02 |  | 0.53 | 0.01 | 0.25 | 0.01 |  |
| **Luxembourg** | 0.11 | 0.01 | 0.05 | 0.01 | 1.17 | 0.08 | 0.00 | 0.03 | 0.00 | 1.00 |
| Lower bound 95%CI | -0.03 | 0.00 | -0.01 | 0.00 |  | -0.02 | 0.00 | -0.01 | 0.00 |  |
| Upper bound 95%CI | 0.25 | 0.02 | 0.11 | 0.02 |  | 0.17 | 0.01 | 0.07 | 0.01 |  |
| **Malta** | 0.26 | 0.01 | 0.13 | 0.02 | 1.58 | 0.16 | 0.01 | 0.09 | 0.01 | 1.72 |
| Lower bound 95%CI | -0.06 | 0.00 | -0.03 | 0.00 |  | -0.04 | 0.00 | -0.02 | 0.00 |  |
| Upper bound 95%CI | 0.57 | 0.03 | 0.29 | 0.06 |  | 0.39 | 0.02 | 0.22 | 0.04 |  |
| **Netherlands** | 0.10 | 0.00 | 0.04 | 0.00 | 1.16 | 0.07 | 0.00 | 0.03 | 0.00 | 1.26 |
| Lower bound 95%CI | -0.02 | 0.00 | -0.01 | 0.00 |  | -0.02 | 0.00 | -0.01 | 0.00 |  |
| Upper bound 95%CI | 0.21 | 0.01 | 0.09 | 0.01 |  | 0.16 | 0.01 | 0.07 | 0.01 |  |
| **Poland** | 0.22 | 0.01 | 0.10 | 0.00 | 0.84 | 0.21 | 0.01 | 0.09 | 0.00 | 0.79 |
| Lower bound 95%CI | -0.05 | 0.00 | -0.02 | 0.00 |  | -0.05 | 0.00 | -0.02 | 0.00 |  |
| Upper bound 95%CI | 0.47 | 0.01 | 0.21 | 0.01 |  | 0.48 | 0.01 | 0.21 | 0.01 |  |
| **Portugal** | 0.06 | 0.00 | 0.03 | 0.01 | 1.51 | 0.06 | 0.00 | 0.03 | 0.01 | 1.36 |
| Lower bound 95%CI | -0.02 | 0.00 | -0.01 | 0.00 |  | -0.02 | 0.00 | -0.01 | 0.00 |  |
| Upper bound 95%CI | 0.14 | 0.01 | 0.07 | 0.02 |  | 0.14 | 0.01 | 0.06 | 0.01 |  |
| **Romania** | 0.33 | 0.01 | 0.19 | 0.01 | 1.01 | 0.34 | 0.01 | 0.18 | 0.01 | 0.84 |
| Lower bound 95%CI | -0.09 | 0.00 | -0.05 | 0.00 |  | -0.08 | 0.00 | -0.05 | 0.00 |  |
| Upper bound 95%CI | 0.70 | 0.02 | 0.41 | 0.02 |  | 0.75 | 0.02 | 0.39 | 0.02 |  |
| **Slovakia** | 0.29 | 0.01 | 0.17 | 0.01 | 1.10 | 0.20 | 0.01 | 0.13 | 0.01 | 1.08 |
| Lower bound 95%CI | -0.07 | 0.00 | -0.04 | 0.00 |  | -0.05 | 0.00 | -0.03 | 0.00 |  |
| Upper bound 95%CI | 0.62 | 0.02 | 0.37 | 0.02 |  | 0.45 | 0.01 | 0.29 | 0.01 |  |
| **Slovenia** | 0.11 | 0.00 | 0.05 | 0.00 | 0.79 | 0.08 | 0.00 | 0.03 | 0.00 | 0.60 |
| Lower bound 95%CI | -0.03 | 0.00 | -0.01 | 0.00 |  | -0.02 | 0.00 | -0.01 | 0.00 |  |
| Upper bound 95%CI | 0.23 | 0.01 | 0.10 | 0.01 |  | 0.18 | 0.01 | 0.07 | 0.00 |  |
| **Spain** | 0.10 | 0.00 | 0.04 | 0.01 | 1.16 | 0.09 | 0.00 | 0.03 | 0.00 | 0.96 |
| Lower bound 95%CI | -0.03 | 0.00 | -0.01 | 0.00 |  | -0.02 | 0.00 | -0.01 | 0.00 |  |
| Upper bound 95%CI | 0.23 | 0.01 | 0.09 | 0.01 |  | 0.20 | 0.01 | 0.07 | 0.01 |  |
| **Sweden** | 0.11 | 0.01 | 0.06 | 0.01 | 1.21 | 0.07 | 0.00 | 0.04 | 0.00 | 1.32 |
| Lower bound 95%CI | -0.03 | 0.00 | -0.01 | 0.00 |  | -0.02 | 0.00 | -0.01 | 0.00 |  |
| Upper bound 95%CI | 0.23 | 0.01 | 0.12 | 0.02 |  | 0.15 | 0.01 | 0.08 | 0.01 |  |
| **European Union** | 0.25 | 0.01 | 0.13 | 0.01 | 1.27 | 0.21 | 0.01 | 0.11 | 0.01 | 1.21 |
| Lower bound 95%CI | -0.06 | 0.00 | -0.03 | 0.00 |  | -0.05 | 0.00 | -0.03 | 0.00 |  |
| Upper bound 95%CI | 0.54 | 0.02 | 0.28 | 0.03 |  | 0.46 | 0.02 | 0.24 | 0.02 |  |

Data and definitions from Global Burden of Disease Database. 2021

*Each value corresponds to mortality rates per 100.000 inhabitants (95% Uncertainty Interval)

Abbreviation: CFI, case-fatality index

| Appendix Table 15. Age-standardised mortality rates and case fatality indexes for IHD attributable to Diet high in trans fatty acids. 2011 , 2021 | | | | | | | | | | |
| --- | --- | --- | --- | --- | --- | --- | --- | --- | --- | --- |
| **Country** | **2011** | | | | | **2021** | | | | |
|  | **Men** | | **Women** | | **CFI Ratio** | **Men** | | **Women** | | **CFI Ratio** |
|  | **Mortality rate** | **CFI** | **Mortality rate** | **CFI** |  | **Mortality rate** | **CFI** | **Mortality rate** | **CFI** |  |
| **Austria** | 0.00 | 0.00 | 0.00 | 0.00 | - | 0.00 | 0.00 | 0.00 | 0.00 | - |
| Lower bound 95%CI | 0.00 | 0.00 | 0.00 | 0.00 |  | 0.00 | 0.00 | 0.00 | 0.00 |  |
| Upper bound 95%CI | 0.00 | 0.00 | 0.00 | 0.00 |  | 0.00 | 0.00 | 0.00 | 0.00 |  |
| **Belgium** | 1.01 | 0.05 | 0.47 | 0.06 | 1.35 | 0.63 | 0.03 | 0.28 | 0.04 | 1.29 |
| Lower bound 95%CI | 0.10 | 0.00 | 0.04 | 0.00 |  | 0.07 | 0.00 | 0.03 | 0.00 |  |
| Upper bound 95%CI | 2.16 | 0.11 | 1.01 | 0.15 |  | 1.34 | 0.07 | 0.61 | 0.09 |  |
| **Bulgaria** | 0.21 | 0.00 | 0.14 | 0.01 | 1.19 | 0.20 | 0.00 | 0.12 | 0.00 | 1.04 |
| Lower bound 95%CI | 0.02 | 0.00 | 0.01 | 0.00 |  | 0.02 | 0.00 | 0.01 | 0.00 |  |
| Upper bound 95%CI | 0.54 | 0.01 | 0.36 | 0.02 |  | 0.49 | 0.01 | 0.31 | 0.01 |  |
| **Croatia** | 0.17 | 0.00 | 0.12 | 0.01 | 1.51 | 0.14 | 0.00 | 0.10 | 0.01 | 1.42 |
| Lower bound 95%CI | 0.01 | 0.00 | 0.01 | 0.00 |  | 0.01 | 0.00 | 0.01 | 0.00 |  |
| Upper bound 95%CI | 0.42 | 0.01 | 0.30 | 0.02 |  | 0.37 | 0.01 | 0.26 | 0.02 |  |
| **Cyprus** | 0.50 | 0.03 | 0.25 | 0.05 | 1.57 | 0.32 | 0.02 | 0.17 | 0.03 | 1.53 |
| Lower bound 95%CI | 0.05 | 0.00 | 0.02 | 0.00 |  | 0.03 | 0.00 | 0.02 | 0.00 |  |
| Upper bound 95%CI | 1.19 | 0.09 | 0.61 | 0.15 |  | 0.74 | 0.05 | 0.43 | 0.08 |  |
| **Czechia** | 0.75 | 0.02 | 0.47 | 0.02 | 1.13 | 0.57 | 0.01 | 0.33 | 0.01 | 0.93 |
| Lower bound 95%CI | 0.08 | 0.00 | 0.04 | 0.00 |  | 0.06 | 0.00 | 0.03 | 0.00 |  |
| Upper bound 95%CI | 1.81 | 0.05 | 1.17 | 0.06 |  | 1.32 | 0.04 | 0.78 | 0.03 |  |
| **Denmark** | 0.00 | 0.00 | 0.00 | 0.00 | - | 0.00 | 0.00 | 0.00 | 0.00 | - |
| Lower bound 95%CI | 0.00 | 0.00 | 0.00 | 0.00 |  | 0.00 | 0.00 | 0.00 | 0.00 |  |
| Upper bound 95%CI | 0.00 | 0.00 | 0.00 | 0.00 |  | 0.00 | 0.00 | 0.00 | 0.00 |  |
| **Estonia** | 4.70 | 0.10 | 2.31 | 0.08 | 0.77 | 1.59 | 0.03 | 0.86 | 0.03 | 0.84 |
| Lower bound 95%CI | 0.43 | 0.01 | 0.20 | 0.01 |  | 0.13 | 0.00 | 0.09 | 0.00 |  |
| Upper bound 95%CI | 9.86 | 0.23 | 4.79 | 0.17 |  | 3.44 | 0.08 | 1.98 | 0.07 |  |
| **Finland** | 0.36 | 0.02 | 0.18 | 0.02 | 1.26 | 0.26 | 0.01 | 0.12 | 0.01 | 1.23 |
| Lower bound 95%CI | 0.04 | 0.00 | 0.02 | 0.00 |  | 0.02 | 0.00 | 0.01 | 0.00 |  |
| Upper bound 95%CI | 0.86 | 0.05 | 0.44 | 0.06 |  | 0.61 | 0.03 | 0.31 | 0.04 |  |
| **France** | 0.36 | 0.01 | 0.15 | 0.02 | 1.32 | 0.26 | 0.01 | 0.10 | 0.01 | 1.19 |
| Lower bound 95%CI | 0.03 | 0.00 | 0.01 | 0.00 |  | 0.02 | 0.00 | 0.01 | 0.00 |  |
| Upper bound 95%CI | 0.75 | 0.03 | 0.34 | 0.05 |  | 0.57 | 0.03 | 0.24 | 0.03 |  |
| **Germany** | 1.71 | 0.06 | 0.87 | 0.09 | 1.61 | 1.31 | 0.05 | 0.58 | 0.07 | 1.30 |
| Lower bound 95%CI | 0.13 | 0.00 | 0.10 | 0.01 |  | 0.12 | 0.00 | 0.06 | 0.01 |  |
| Upper bound 95%CI | 3.57 | 0.13 | 1.90 | 0.21 |  | 2.72 | 0.12 | 1.24 | 0.16 |  |
| **Greece** | 0.31 | 0.02 | 0.22 | 0.04 | 2.44 | 0.25 | 0.01 | 0.13 | 0.02 | 1.64 |
| Lower bound 95%CI | 0.03 | 0.00 | 0.02 | 0.00 |  | 0.03 | 0.00 | 0.01 | 0.00 |  |
| Upper bound 95%CI | 0.73 | 0.04 | 0.60 | 0.12 |  | 0.61 | 0.04 | 0.31 | 0.06 |  |
| **Hungary** | 0.68 | 0.01 | 0.41 | 0.01 | 1.06 | 0.00 | 0.00 | 0.00 | 0.00 | - |
| Lower bound 95%CI | 0.06 | 0.00 | 0.04 | 0.00 |  | 0.00 | 0.00 | 0.00 | 0.00 |  |
| Upper bound 95%CI | 1.68 | 0.04 | 1.06 | 0.04 |  | 0.00 | 0.00 | 0.00 | 0.00 |  |
| **Ireland** | 0.10 | 0.00 | 0.05 | 0.01 | 1.49 | 0.07 | 0.00 | 0.03 | 0.00 | 1.24 |
| Lower bound 95%CI | 0.01 | 0.00 | 0.00 | 0.00 |  | 0.01 | 0.00 | 0.00 | 0.00 |  |
| Upper bound 95%CI | 0.24 | 0.01 | 0.13 | 0.02 |  | 0.18 | 0.01 | 0.10 | 0.02 |  |
| **Italy** | 0.01 | 0.00 | 0.01 | 0.00 | 2.70 | 0.01 | 0.00 | 0.00 | 0.00 | 0.00 |
| Lower bound 95%CI | 0.00 | 0.00 | 0.00 | 0.00 |  | 0.00 | 0.00 | 0.00 | 0.00 |  |
| Upper bound 95%CI | 0.03 | 0.00 | 0.02 | 0.00 |  | 0.03 | 0.00 | 0.01 | 0.00 |  |
| **Latvia** | 21.84 | 0.55 | 10.22 | 0.51 | 0.92 | 0.00 | 0.00 | 0.00 | 0.00 | - |
| Lower bound 95%CI | 1.79 | 0.04 | 0.72 | 0.03 |  | 0.00 | 0.00 | 0.00 | 0.00 |  |
| Upper bound 95%CI | 40.96 | 1.12 | 20.06 | 1.12 |  | 0.00 | 0.00 | 0.00 | 0.00 |  |
| **Lithuania** | 3.84 | 0.09 | 2.01 | 0.10 | 1.07 | 0.00 | 0.00 | 0.00 | 0.00 | - |
| Lower bound 95%CI | 0.42 | 0.01 | 0.22 | 0.01 |  | 0.00 | 0.00 | 0.00 | 0.00 |  |
| Upper bound 95%CI | 8.02 | 0.21 | 4.10 | 0.23 |  | 0.00 | 0.00 | 0.00 | 0.00 |  |
| **Luxembourg** | 0.27 | 0.02 | 0.13 | 0.02 | 1.24 | 0.18 | 0.01 | 0.09 | 0.01 | 1.34 |
| Lower bound 95%CI | 0.04 | 0.00 | 0.01 | 0.00 |  | 0.02 | 0.00 | 0.01 | 0.00 |  |
| Upper bound 95%CI | 0.63 | 0.05 | 0.34 | 0.07 |  | 0.43 | 0.03 | 0.20 | 0.03 |  |
| **Malta** | 0.41 | 0.02 | 0.24 | 0.04 | 1.84 | 0.23 | 0.01 | 0.17 | 0.03 | 2.26 |
| Lower bound 95%CI | 0.04 | 0.00 | 0.03 | 0.00 |  | 0.02 | 0.00 | 0.02 | 0.00 |  |
| Upper bound 95%CI | 0.97 | 0.06 | 0.61 | 0.12 |  | 0.56 | 0.03 | 0.40 | 0.07 |  |
| **Netherlands** | 1.59 | 0.07 | 0.72 | 0.09 | 1.32 | 1.10 | 0.05 | 0.50 | 0.06 | 1.33 |
| Lower bound 95%CI | 0.14 | 0.01 | 0.07 | 0.01 |  | 0.08 | 0.00 | 0.04 | 0.00 |  |
| Upper bound 95%CI | 3.22 | 0.15 | 1.45 | 0.20 |  | 2.26 | 0.11 | 1.06 | 0.16 |  |
| **Poland** | 0.19 | 0.00 | 0.10 | 0.00 | 0.98 | 0.16 | 0.00 | 0.09 | 0.00 | 1.04 |
| Lower bound 95%CI | 0.02 | 0.00 | 0.01 | 0.00 |  | 0.02 | 0.00 | 0.01 | 0.00 |  |
| Upper bound 95%CI | 0.47 | 0.01 | 0.25 | 0.01 |  | 0.40 | 0.01 | 0.22 | 0.01 |  |
| **Portugal** | 0.16 | 0.01 | 0.09 | 0.02 | 1.69 | 0.13 | 0.01 | 0.07 | 0.01 | 1.47 |
| Lower bound 95%CI | 0.01 | 0.00 | 0.01 | 0.00 |  | 0.01 | 0.00 | 0.01 | 0.00 |  |
| Upper bound 95%CI | 0.36 | 0.03 | 0.22 | 0.05 |  | 0.30 | 0.02 | 0.16 | 0.04 |  |
| **Romania** | 0.42 | 0.01 | 0.27 | 0.01 | 1.13 | 0.32 | 0.01 | 0.21 | 0.01 | 1.04 |
| Lower bound 95%CI | 0.04 | 0.00 | 0.02 | 0.00 |  | 0.03 | 0.00 | 0.02 | 0.00 |  |
| Upper bound 95%CI | 1.03 | 0.03 | 0.70 | 0.04 |  | 0.82 | 0.02 | 0.53 | 0.02 |  |
| **Slovakia** | 1.28 | 0.03 | 0.81 | 0.04 | 1.18 | 1.00 | 0.03 | 0.65 | 0.03 | 1.08 |
| Lower bound 95%CI | 0.13 | 0.00 | 0.08 | 0.00 |  | 0.09 | 0.00 | 0.04 | 0.00 |  |
| Upper bound 95%CI | 2.86 | 0.09 | 1.92 | 0.11 |  | 2.33 | 0.07 | 1.66 | 0.08 |  |
| **Slovenia** | 3.02 | 0.09 | 1.36 | 0.07 | 0.78 | 0.00 | 0.00 | 0.00 | 0.00 | - |
| Lower bound 95%CI | 0.26 | 0.01 | 0.12 | 0.01 |  | 0.00 | 0.00 | 0.00 | 0.00 |  |
| Upper bound 95%CI | 5.78 | 0.19 | 2.78 | 0.16 |  | 0.00 | 0.00 | 0.00 | 0.00 |  |
| **Spain** | 0.00 | 0.00 | 0.00 | 0.00 | - | 0.01 | 0.00 | 0.00 | 0.00 | 0.00 |
| Lower bound 95%CI | 0.00 | 0.00 | 0.00 | 0.00 |  | 0.00 | 0.00 | 0.00 | 0.00 |  |
| Upper bound 95%CI | 0.02 | 0.00 | 0.01 | 0.00 |  | 0.02 | 0.00 | 0.01 | 0.00 |  |
| **Sweden** | 0.04 | 0.00 | 0.03 | 0.00 | 1.66 | 0.03 | 0.00 | 0.02 | 0.00 | 1.54 |
| Lower bound 95%CI | 0.00 | 0.00 | 0.00 | 0.00 |  | 0.00 | 0.00 | 0.00 | 0.00 |  |
| Upper bound 95%CI | 0.11 | 0.01 | 0.07 | 0.01 |  | 0.07 | 0.00 | 0.04 | 0.01 |  |
| **European Union** | 0.81 | 0.03 | 0.44 | 0.04 | 1.32 | 0.46 | 0.02 | 0.25 | 0.02 | 1.25 |
| Lower bound 95%CI | 0.08 | 0.00 | 0.05 | 0.00 |  | 0.05 | 0.00 | 0.03 | 0.00 |  |
| Upper bound 95%CI | 1.63 | 0.06 | 0.91 | 0.09 |  | 0.94 | 0.04 | 0.50 | 0.05 |  |

Data and definitions from Global Burden of Disease Database. 2021

*Each value corresponds to mortality rates per 100.000 inhabitants (95% Uncertainty Interval)

Abbreviation: CFI, case-fatality index

| Appendix Table 16. Age-standardised mortality rates and case fatality indexes for IHD attributable to Diet low in fiber.  2011 , 2021 | | | | | | | | | | |
| --- | --- | --- | --- | --- | --- | --- | --- | --- | --- | --- |
| **Country** | **2011** | | | | | **2021** | | | | |
|  | **Men** | | **Women** | | **CFI Ratio** | **Men** | | **Women** | | **CFI Ratio** |
|  | **Mortality rate** | **CFI** | **Mortality rate** | **CFI** |  | **Mortality rate** | **CFI** | **Mortality rate** | **CFI** |  |
| **Austria** | 4.57 | 0.18 | 2.64 | 0.32 | 1.83 | 3.28 | 0.13 | 1.75 | 0.21 | 1.69 |
| Lower bound 95%CI | 2.26 | 0.08 | 1.15 | 0.13 |  | 1.60 | 0.05 | 0.75 | 0.08 |  |
| Upper bound 95%CI | 7.30 | 0.31 | 4.36 | 0.59 |  | 5.61 | 0.24 | 2.93 | 0.41 |  |
| **Belgium** | 5.08 | 0.23 | 2.39 | 0.32 | 1.37 | 3.08 | 0.14 | 1.41 | 0.19 | 1.32 |
| Lower bound 95%CI | 2.51 | 0.10 | 1.17 | 0.14 |  | 1.57 | 0.06 | 0.69 | 0.08 |  |
| Upper bound 95%CI | 7.52 | 0.38 | 3.83 | 0.57 |  | 4.65 | 0.24 | 2.27 | 0.34 |  |
| **Bulgaria** | 27.41 | 0.60 | 15.48 | 0.60 | 1.01 | 21.15 | 0.48 | 11.52 | 0.46 | 0.94 |
| Lower bound 95%CI | 13.99 | 0.28 | 7.85 | 0.28 |  | 10.55 | 0.22 | 5.69 | 0.20 |  |
| Upper bound 95%CI | 40.54 | 0.98 | 23.75 | 1.02 |  | 32.36 | 0.84 | 18.32 | 0.82 |  |
| **Croatia** | 16.42 | 0.45 | 10.11 | 0.59 | 1.32 | 12.30 | 0.32 | 7.81 | 0.40 | 1.26 |
| Lower bound 95%CI | 8.52 | 0.20 | 5.05 | 0.26 |  | 6.10 | 0.14 | 3.74 | 0.17 |  |
| Upper bound 95%CI | 24.55 | 0.76 | 15.51 | 1.02 |  | 18.33 | 0.55 | 12.40 | 0.73 |  |
| **Cyprus** | 16.58 | 1.07 | 7.75 | 1.57 | 1.47 | 11.17 | 0.61 | 5.48 | 0.87 | 1.41 |
| Lower bound 95%CI | 8.34 | 0.47 | 3.77 | 0.64 |  | 5.33 | 0.25 | 2.48 | 0.33 |  |
| Upper bound 95%CI | 24.74 | 1.83 | 11.61 | 2.77 |  | 17.04 | 1.09 | 8.40 | 1.56 |  |
| **Czechia** | 16.58 | 0.40 | 9.62 | 0.42 | 1.04 | 10.91 | 0.26 | 6.15 | 0.23 | 0.90 |
| Lower bound 95%CI | 8.14 | 0.18 | 4.65 | 0.18 |  | 5.23 | 0.11 | 2.88 | 0.10 |  |
| Upper bound 95%CI | 24.83 | 0.66 | 15.32 | 0.73 |  | 16.61 | 0.45 | 9.88 | 0.42 |  |
| **Denmark** | 4.13 | 0.27 | 2.14 | 0.37 | 1.38 | 2.74 | 0.17 | 1.28 | 0.23 | 1.36 |
| Lower bound 95%CI | 2.06 | 0.12 | 1.00 | 0.15 |  | 1.36 | 0.07 | 0.57 | 0.09 |  |
| Upper bound 95%CI | 6.25 | 0.45 | 3.42 | 0.66 |  | 4.42 | 0.31 | 2.15 | 0.43 |  |
| **Estonia** | 6.44 | 0.14 | 3.50 | 0.12 | 0.85 | 2.89 | 0.06 | 1.53 | 0.05 | 0.83 |
| Lower bound 95%CI | 3.09 | 0.06 | 1.51 | 0.05 |  | 1.28 | 0.02 | 0.60 | 0.02 |  |
| Upper bound 95%CI | 10.36 | 0.24 | 6.08 | 0.22 |  | 4.89 | 0.11 | 2.85 | 0.10 |  |
| **Finland** | 8.59 | 0.41 | 4.34 | 0.52 | 1.27 | 5.66 | 0.23 | 2.86 | 0.31 | 1.35 |
| Lower bound 95%CI | 4.21 | 0.18 | 2.06 | 0.22 |  | 2.78 | 0.10 | 1.32 | 0.13 |  |
| Upper bound 95%CI | 13.32 | 0.71 | 6.99 | 0.95 |  | 8.92 | 0.42 | 4.66 | 0.59 |  |
| **France** | 4.73 | 0.18 | 1.89 | 0.23 | 1.26 | 3.23 | 0.13 | 1.27 | 0.16 | 1.22 |
| Lower bound 95%CI | 2.41 | 0.08 | 0.90 | 0.10 |  | 1.58 | 0.06 | 0.60 | 0.06 |  |
| Upper bound 95%CI | 7.00 | 0.29 | 2.94 | 0.39 |  | 5.08 | 0.23 | 1.98 | 0.27 |  |
| **Germany** | 6.31 | 0.21 | 3.30 | 0.34 | 1.65 | 4.80 | 0.19 | 2.19 | 0.25 | 1.34 |
| Lower bound 95%CI | 3.14 | 0.09 | 1.55 | 0.15 |  | 2.37 | 0.08 | 0.99 | 0.10 |  |
| Upper bound 95%CI | 9.52 | 0.34 | 5.29 | 0.60 |  | 7.26 | 0.32 | 3.41 | 0.44 |  |
| **Greece** | 4.34 | 0.22 | 2.95 | 0.50 | 2.33 | 3.84 | 0.21 | 2.02 | 0.35 | 1.66 |
| Lower bound 95%CI | 2.15 | 0.10 | 1.35 | 0.20 |  | 1.99 | 0.10 | 0.91 | 0.13 |  |
| Upper bound 95%CI | 6.73 | 0.37 | 4.99 | 0.98 |  | 5.84 | 0.36 | 3.38 | 0.68 |  |
| **Hungary** | 18.29 | 0.37 | 10.71 | 0.39 | 1.03 | 12.99 | 0.28 | 7.57 | 0.28 | 0.99 |
| Lower bound 95%CI | 9.29 | 0.17 | 5.25 | 0.17 |  | 6.41 | 0.13 | 3.70 | 0.12 |  |
| Upper bound 95%CI | 27.58 | 0.62 | 16.85 | 0.67 |  | 19.80 | 0.48 | 11.99 | 0.50 |  |
| **Ireland** | 5.29 | 0.24 | 2.70 | 0.37 | 1.52 | 2.61 | 0.12 | 1.31 | 0.17 | 1.46 |
| Lower bound 95%CI | 2.73 | 0.11 | 1.27 | 0.15 |  | 1.25 | 0.05 | 0.60 | 0.07 |  |
| Upper bound 95%CI | 8.22 | 0.41 | 4.38 | 0.67 |  | 4.36 | 0.23 | 2.24 | 0.34 |  |
| **Italy** | 3.73 | 0.15 | 1.83 | 0.20 | 1.32 | 3.05 | 0.13 | 1.41 | 0.16 | 1.22 |
| Lower bound 95%CI | 1.91 | 0.07 | 0.83 | 0.08 |  | 1.47 | 0.05 | 0.62 | 0.06 |  |
| Upper bound 95%CI | 5.93 | 0.28 | 3.16 | 0.40 |  | 4.83 | 0.25 | 2.41 | 0.33 |  |
| **Latvia** | 20.11 | 0.50 | 10.15 | 0.50 | 1.00 | 11.89 | 0.28 | 5.79 | 0.24 | 0.87 |
| Lower bound 95%CI | 9.92 | 0.23 | 4.72 | 0.21 |  | 5.59 | 0.12 | 2.67 | 0.10 |  |
| Upper bound 95%CI | 29.96 | 0.82 | 16.39 | 0.92 |  | 18.75 | 0.50 | 9.23 | 0.44 |  |
| **Lithuania** | 9.78 | 0.24 | 5.06 | 0.25 | 1.05 | 5.44 | 0.13 | 2.82 | 0.12 | 0.93 |
| Lower bound 95%CI | 4.53 | 0.10 | 2.22 | 0.10 |  | 2.34 | 0.05 | 1.12 | 0.04 |  |
| Upper bound 95%CI | 15.28 | 0.40 | 8.61 | 0.48 |  | 9.16 | 0.25 | 5.29 | 0.26 |  |
| **Luxembourg** | 5.94 | 0.38 | 2.82 | 0.46 | 1.22 | 3.74 | 0.20 | 1.76 | 0.26 | 1.26 |
| Lower bound 95%CI | 2.86 | 0.16 | 1.44 | 0.20 |  | 1.78 | 0.09 | 0.84 | 0.11 |  |
| Upper bound 95%CI | 9.20 | 0.66 | 4.31 | 0.83 |  | 5.71 | 0.36 | 2.82 | 0.48 |  |
| **Malta** | 5.86 | 0.32 | 3.66 | 0.62 | 1.97 | 3.27 | 0.17 | 2.31 | 0.36 | 2.16 |
| Lower bound 95%CI | 2.67 | 0.13 | 1.60 | 0.23 |  | 1.48 | 0.07 | 1.05 | 0.14 |  |
| Upper bound 95%CI | 8.87 | 0.54 | 5.87 | 1.16 |  | 4.99 | 0.30 | 3.91 | 0.71 |  |
| **Netherlands** | 4.78 | 0.20 | 2.26 | 0.28 | 1.38 | 3.28 | 0.15 | 1.54 | 0.20 | 1.38 |
| Lower bound 95%CI | 2.34 | 0.09 | 1.10 | 0.12 |  | 1.56 | 0.06 | 0.72 | 0.08 |  |
| Upper bound 95%CI | 7.08 | 0.33 | 3.46 | 0.48 |  | 5.19 | 0.26 | 2.49 | 0.37 |  |
| **Poland** | 5.55 | 0.14 | 2.89 | 0.14 | 0.97 | 3.59 | 0.09 | 1.91 | 0.09 | 0.99 |
| Lower bound 95%CI | 2.69 | 0.06 | 1.22 | 0.05 |  | 1.66 | 0.04 | 0.82 | 0.03 |  |
| Upper bound 95%CI | 8.60 | 0.25 | 4.99 | 0.27 |  | 6.07 | 0.18 | 3.45 | 0.19 |  |
| **Portugal** | 2.87 | 0.20 | 1.59 | 0.33 | 1.67 | 2.31 | 0.16 | 1.13 | 0.21 | 1.34 |
| Lower bound 95%CI | 1.42 | 0.08 | 0.74 | 0.13 |  | 1.13 | 0.07 | 0.50 | 0.08 |  |
| Upper bound 95%CI | 4.48 | 0.35 | 2.61 | 0.63 |  | 3.63 | 0.29 | 1.88 | 0.43 |  |
| **Romania** | 5.04 | 0.13 | 3.35 | 0.15 | 1.17 | 3.69 | 0.09 | 2.42 | 0.09 | 1.04 |
| Lower bound 95%CI | 2.29 | 0.05 | 1.54 | 0.06 |  | 1.56 | 0.03 | 1.05 | 0.04 |  |
| Upper bound 95%CI | 8.27 | 0.24 | 5.81 | 0.29 |  | 6.11 | 0.17 | 4.32 | 0.18 |  |
| **Slovakia** | 27.63 | 0.75 | 16.42 | 0.83 | 1.11 | 18.89 | 0.49 | 12.13 | 0.52 | 1.07 |
| Lower bound 95%CI | 14.23 | 0.34 | 8.07 | 0.36 |  | 9.83 | 0.23 | 5.94 | 0.23 |  |
| Upper bound 95%CI | 40.33 | 1.21 | 25.07 | 1.41 |  | 28.98 | 0.85 | 18.27 | 0.88 |  |
| **Slovenia** | 4.14 | 0.12 | 2.00 | 0.10 | 0.84 | 2.98 | 0.07 | 1.22 | 0.05 | 0.66 |
| Lower bound 95%CI | 1.98 | 0.05 | 0.99 | 0.04 |  | 1.43 | 0.03 | 0.52 | 0.02 |  |
| Upper bound 95%CI | 6.52 | 0.21 | 3.21 | 0.18 |  | 4.80 | 0.14 | 2.08 | 0.09 |  |
| **Spain** | 4.49 | 0.21 | 1.88 | 0.26 | 1.22 | 3.28 | 0.17 | 1.29 | 0.19 | 1.13 |
| Lower bound 95%CI | 2.29 | 0.10 | 0.92 | 0.11 |  | 1.58 | 0.07 | 0.62 | 0.08 |  |
| Upper bound 95%CI | 6.69 | 0.35 | 3.01 | 0.47 |  | 5.02 | 0.29 | 2.05 | 0.35 |  |
| **Sweden** | 6.40 | 0.33 | 3.76 | 0.43 | 1.30 | 3.63 | 0.18 | 2.16 | 0.24 | 1.38 |
| Lower bound 95%CI | 3.07 | 0.14 | 1.85 | 0.18 |  | 1.68 | 0.07 | 0.98 | 0.09 |  |
| Upper bound 95%CI | 9.97 | 0.59 | 5.90 | 0.78 |  | 5.81 | 0.33 | 3.62 | 0.48 |  |
| **European Union** | 17.71 | 0.63 | 10.08 | 0.87 | 1.39 | 12.04 | 0.45 | 7.23 | 0.62 | 1.38 |
| Lower bound 95%CI | 9.20 | 0.30 | 5.06 | 0.39 |  | 6.15 | 0.20 | 3.63 | 0.27 |  |
| Upper bound 95%CI | 25.77 | 1.02 | 15.13 | 1.47 |  | 17.95 | 0.76 | 10.99 | 1.08 |  |

Data and definitions from Global Burden of Disease Database. 2021

*Each value corresponds to mortality rates per 100.000 inhabitants (95% Uncertainty Interval)

Abbreviation: CFI, case-fatality index

| Appendix Table 17. Age-standardised mortality rates and case fatality indexes for IHD attributable to Diet low in fruits.  2011 , 2021 | | | | | | | | | | |
| --- | --- | --- | --- | --- | --- | --- | --- | --- | --- | --- |
| **Country** | **2011** | | | | | **2021** | | | | |
|  | **Men** | | **Women** | | **CFI Ratio** | **Men** | | **Women** | | **CFI Ratio** |
|  | **Mortality rate** | **CFI** | **Mortality rate** | **CFI** |  | **Mortality rate** | **CFI** | **Mortality rate** | **CFI** |  |
| **Austria** | 1.93 | 0.07 | 1.17 | 0.14 | 1.92 | 1.34 | 0.05 | 0.76 | 0.09 | 1.80 |
| Lower bound 95%CI | 0.38 | 0.01 | 0.24 | 0.03 |  | 0.26 | 0.01 | 0.14 | 0.02 |  |
| Upper bound 95%CI | 3.75 | 0.16 | 2.51 | 0.34 |  | 2.74 | 0.12 | 1.61 | 0.22 |  |
| **Belgium** | 2.77 | 0.13 | 1.38 | 0.18 | 1.45 | 1.86 | 0.08 | 0.88 | 0.12 | 1.37 |
| Lower bound 95%CI | 0.56 | 0.02 | 0.24 | 0.03 |  | 0.41 | 0.02 | 0.16 | 0.02 |  |
| Upper bound 95%CI | 5.03 | 0.25 | 2.54 | 0.38 |  | 3.36 | 0.18 | 1.69 | 0.26 |  |
| **Bulgaria** | 20.97 | 0.46 | 11.35 | 0.44 | 0.96 | 16.01 | 0.37 | 8.39 | 0.33 | 0.91 |
| Lower bound 95%CI | 4.29 | 0.09 | 2.14 | 0.08 |  | 3.22 | 0.07 | 1.59 | 0.06 |  |
| Upper bound 95%CI | 35.49 | 0.86 | 20.11 | 0.86 |  | 27.54 | 0.71 | 15.05 | 0.67 |  |
| **Croatia** | 5.51 | 0.15 | 3.78 | 0.22 | 1.47 | 4.03 | 0.11 | 2.81 | 0.15 | 1.38 |
| Lower bound 95%CI | 1.15 | 0.03 | 0.67 | 0.03 |  | 0.84 | 0.02 | 0.45 | 0.02 |  |
| Upper bound 95%CI | 10.19 | 0.31 | 7.34 | 0.48 |  | 7.54 | 0.23 | 5.76 | 0.34 |  |
| **Cyprus** | 3.57 | 0.23 | 1.74 | 0.35 | 1.53 | 2.70 | 0.15 | 1.33 | 0.21 | 1.42 |
| Lower bound 95%CI | 0.79 | 0.04 | 0.35 | 0.06 |  | 0.58 | 0.03 | 0.23 | 0.03 |  |
| Upper bound 95%CI | 6.67 | 0.49 | 3.52 | 0.84 |  | 5.13 | 0.33 | 2.75 | 0.51 |  |
| **Czechia** | 13.02 | 0.31 | 7.26 | 0.31 | 1.00 | 8.38 | 0.20 | 4.55 | 0.17 | 0.87 |
| Lower bound 95%CI | 2.48 | 0.05 | 1.42 | 0.06 |  | 1.74 | 0.04 | 0.97 | 0.03 |  |
| Upper bound 95%CI | 23.39 | 0.62 | 13.32 | 0.64 |  | 14.78 | 0.40 | 8.37 | 0.36 |  |
| **Denmark** | 1.46 | 0.09 | 0.89 | 0.15 | 1.62 | 1.16 | 0.07 | 0.61 | 0.11 | 1.53 |
| Lower bound 95%CI | 0.29 | 0.02 | 0.18 | 0.03 |  | 0.23 | 0.01 | 0.11 | 0.02 |  |
| Upper bound 95%CI | 2.91 | 0.21 | 1.84 | 0.36 |  | 2.37 | 0.16 | 1.23 | 0.25 |  |
| **Estonia** | 15.15 | 0.32 | 7.08 | 0.23 | 0.73 | 7.52 | 0.15 | 3.23 | 0.10 | 0.67 |
| Lower bound 95%CI | 3.05 | 0.06 | 1.41 | 0.04 |  | 1.45 | 0.03 | 0.59 | 0.02 |  |
| Upper bound 95%CI | 26.54 | 0.61 | 12.81 | 0.46 |  | 13.44 | 0.30 | 6.12 | 0.21 |  |
| **Finland** | 7.93 | 0.38 | 3.62 | 0.44 | 1.15 | 5.60 | 0.23 | 2.49 | 0.27 | 1.18 |
| Lower bound 95%CI | 1.62 | 0.07 | 0.80 | 0.09 |  | 1.19 | 0.04 | 0.53 | 0.05 |  |
| Upper bound 95%CI | 13.94 | 0.75 | 6.57 | 0.89 |  | 10.21 | 0.48 | 4.54 | 0.58 |  |
| **France** | 3.41 | 0.13 | 1.34 | 0.16 | 1.24 | 2.31 | 0.09 | 0.88 | 0.11 | 1.18 |
| Lower bound 95%CI | 0.70 | 0.02 | 0.28 | 0.03 |  | 0.49 | 0.02 | 0.19 | 0.02 |  |
| Upper bound 95%CI | 6.01 | 0.25 | 2.47 | 0.33 |  | 4.08 | 0.18 | 1.57 | 0.22 |  |
| **Germany** | 4.45 | 0.15 | 2.26 | 0.23 | 1.60 | 3.35 | 0.13 | 1.49 | 0.17 | 1.30 |
| Lower bound 95%CI | 0.86 | 0.03 | 0.46 | 0.04 |  | 0.66 | 0.02 | 0.31 | 0.03 |  |
| Upper bound 95%CI | 7.79 | 0.28 | 4.24 | 0.48 |  | 6.20 | 0.27 | 2.76 | 0.35 |  |
| **Greece** | 0.64 | 0.03 | 0.52 | 0.09 | 2.79 | 0.66 | 0.04 | 0.38 | 0.07 | 1.81 |
| Lower bound 95%CI | 0.13 | 0.01 | 0.09 | 0.01 |  | 0.15 | 0.01 | 0.06 | 0.01 |  |
| Upper bound 95%CI | 1.25 | 0.07 | 1.23 | 0.24 |  | 1.29 | 0.08 | 0.89 | 0.18 |  |
| **Hungary** | 15.11 | 0.31 | 8.54 | 0.31 | 1.00 | 10.34 | 0.23 | 5.87 | 0.22 | 0.97 |
| Lower bound 95%CI | 3.01 | 0.06 | 1.78 | 0.06 |  | 2.09 | 0.04 | 1.18 | 0.04 |  |
| Upper bound 95%CI | 26.37 | 0.59 | 15.34 | 0.61 |  | 18.32 | 0.45 | 10.61 | 0.44 |  |
| **Ireland** | 4.89 | 0.22 | 2.52 | 0.34 | 1.53 | 2.60 | 0.12 | 1.32 | 0.18 | 1.47 |
| Lower bound 95%CI | 0.94 | 0.04 | 0.49 | 0.06 |  | 0.53 | 0.02 | 0.26 | 0.03 |  |
| Upper bound 95%CI | 8.77 | 0.44 | 4.64 | 0.71 |  | 4.67 | 0.25 | 2.44 | 0.37 |  |
| **Italy** | 0.99 | 0.04 | 0.54 | 0.06 | 1.47 | 0.81 | 0.04 | 0.41 | 0.05 | 1.34 |
| Lower bound 95%CI | 0.21 | 0.01 | 0.11 | 0.01 |  | 0.16 | 0.01 | 0.08 | 0.01 |  |
| Upper bound 95%CI | 1.90 | 0.09 | 1.11 | 0.14 |  | 1.63 | 0.08 | 0.89 | 0.12 |  |
| **Latvia** | 28.08 | 0.70 | 13.14 | 0.65 | 0.92 | 18.19 | 0.43 | 8.03 | 0.34 | 0.79 |
| Lower bound 95%CI | 5.49 | 0.13 | 2.56 | 0.11 |  | 3.86 | 0.08 | 1.51 | 0.06 |  |
| Upper bound 95%CI | 48.32 | 1.33 | 22.89 | 1.28 |  | 32.23 | 0.86 | 14.15 | 0.68 |  |
| **Lithuania** | 24.42 | 0.60 | 11.98 | 0.59 | 1.00 | 15.31 | 0.37 | 7.52 | 0.33 | 0.89 |
| Lower bound 95%CI | 4.92 | 0.11 | 2.45 | 0.11 |  | 3.01 | 0.07 | 1.47 | 0.06 |  |
| Upper bound 95%CI | 41.94 | 1.10 | 21.36 | 1.18 |  | 27.57 | 0.74 | 13.94 | 0.68 |  |
| **Luxembourg** | 2.90 | 0.18 | 1.36 | 0.22 | 1.21 | 2.01 | 0.11 | 0.91 | 0.13 | 1.21 |
| Lower bound 95%CI | 0.61 | 0.03 | 0.28 | 0.04 |  | 0.46 | 0.02 | 0.19 | 0.02 |  |
| Upper bound 95%CI | 5.20 | 0.37 | 2.53 | 0.49 |  | 3.71 | 0.23 | 1.72 | 0.29 |  |
| **Malta** | 5.10 | 0.28 | 2.96 | 0.51 | 1.83 | 2.90 | 0.15 | 1.90 | 0.30 | 2.00 |
| Lower bound 95%CI | 1.04 | 0.05 | 0.57 | 0.08 |  | 0.64 | 0.03 | 0.38 | 0.05 |  |
| Upper bound 95%CI | 9.06 | 0.55 | 5.42 | 1.07 |  | 5.09 | 0.30 | 3.62 | 0.66 |  |
| **Netherlands** | 2.19 | 0.09 | 1.05 | 0.13 | 1.39 | 1.53 | 0.07 | 0.72 | 0.09 | 1.38 |
| Lower bound 95%CI | 0.46 | 0.02 | 0.20 | 0.02 |  | 0.35 | 0.01 | 0.13 | 0.01 |  |
| Upper bound 95%CI | 3.94 | 0.18 | 1.97 | 0.27 |  | 2.89 | 0.15 | 1.35 | 0.20 |  |
| **Poland** | 12.46 | 0.31 | 6.04 | 0.28 | 0.90 | 8.60 | 0.22 | 4.38 | 0.21 | 0.94 |
| Lower bound 95%CI | 2.60 | 0.06 | 1.29 | 0.05 |  | 1.96 | 0.04 | 0.98 | 0.04 |  |
| Upper bound 95%CI | 21.49 | 0.63 | 10.84 | 0.59 |  | 15.40 | 0.46 | 8.01 | 0.45 |  |
| **Portugal** | 1.35 | 0.09 | 0.77 | 0.16 | 1.72 | 1.19 | 0.08 | 0.57 | 0.11 | 1.31 |
| Lower bound 95%CI | 0.27 | 0.02 | 0.15 | 0.03 |  | 0.26 | 0.02 | 0.12 | 0.02 |  |
| Upper bound 95%CI | 2.44 | 0.19 | 1.45 | 0.35 |  | 2.17 | 0.17 | 1.11 | 0.26 |  |
| **Romania** | 9.61 | 0.25 | 5.98 | 0.27 | 1.09 | 6.56 | 0.16 | 3.97 | 0.15 | 0.96 |
| Lower bound 95%CI | 1.86 | 0.04 | 1.18 | 0.05 |  | 1.34 | 0.03 | 0.77 | 0.03 |  |
| Upper bound 95%CI | 17.12 | 0.49 | 11.24 | 0.56 |  | 11.98 | 0.33 | 7.55 | 0.32 |  |
| **Slovakia** | 17.57 | 0.47 | 10.56 | 0.53 | 1.12 | 13.00 | 0.34 | 8.31 | 0.36 | 1.06 |
| Lower bound 95%CI | 3.50 | 0.08 | 1.99 | 0.09 |  | 2.44 | 0.06 | 1.56 | 0.06 |  |
| Upper bound 95%CI | 30.98 | 0.93 | 19.34 | 1.09 |  | 23.36 | 0.69 | 14.96 | 0.72 |  |
| **Slovenia** | 1.47 | 0.04 | 0.76 | 0.04 | 0.89 | 1.21 | 0.03 | 0.49 | 0.02 | 0.65 |
| Lower bound 95%CI | 0.26 | 0.01 | 0.16 | 0.01 |  | 0.27 | 0.01 | 0.09 | 0.00 |  |
| Upper bound 95%CI | 2.75 | 0.09 | 1.60 | 0.09 |  | 2.30 | 0.07 | 1.03 | 0.05 |  |
| **Spain** | 0.68 | 0.03 | 0.35 | 0.05 | 1.50 | 0.58 | 0.03 | 0.25 | 0.04 | 1.24 |
| Lower bound 95%CI | 0.14 | 0.01 | 0.07 | 0.01 |  | 0.12 | 0.01 | 0.05 | 0.01 |  |
| Upper bound 95%CI | 1.35 | 0.07 | 0.75 | 0.12 |  | 1.15 | 0.07 | 0.55 | 0.09 |  |
| **Sweden** | 3.81 | 0.20 | 2.10 | 0.24 | 1.22 | 2.22 | 0.11 | 1.23 | 0.14 | 1.28 |
| Lower bound 95%CI | 0.78 | 0.03 | 0.42 | 0.04 |  | 0.51 | 0.02 | 0.28 | 0.03 |  |
| Upper bound 95%CI | 6.90 | 0.41 | 3.86 | 0.51 |  | 4.18 | 0.24 | 2.29 | 0.30 |  |
| **European Union** | 23.86 | 0.85 | 13.56 | 1.18 | 1.38 | 16.04 | 0.60 | 9.65 | 0.83 | 1.39 |
| Lower bound 95%CI | 4.84 | 0.16 | 2.80 | 0.22 |  | 3.33 | 0.11 | 2.06 | 0.16 |  |
| Upper bound 95%CI | 40.53 | 1.60 | 23.53 | 2.28 |  | 27.80 | 1.18 | 16.88 | 1.65 |  |

Data and definitions from Global Burden of Disease Database. 2021

*Each value corresponds to mortality rates per 100.000 inhabitants (95% Uncertainty Interval)

Abbreviation: CFI, case-fatality index

| Appendix Table 18. Age-standardised mortality rates and case fatality indexes for IHD attributable to Diet low in legumes.  2011 , 2021 | | | | | | | | | | |
| --- | --- | --- | --- | --- | --- | --- | --- | --- | --- | --- |
| **Country** | **2011** | | | | | **2021** | | | | |
|  | **Men** | | **Women** | | **CFI Ratio** | **Men** | | **Women** | | **CFI Ratio** |
|  | **Mortality rate** | **CFI** | **Mortality rate** | **CFI** |  | **Mortality rate** | **CFI** | **Mortality rate** | **CFI** |  |
| **Austria** | 9.56 | 0.37 | 4.68 | 0.57 | 1.55 | 6.72 | 0.26 | 3.11 | 0.38 | 1.47 |
| Lower bound 95%CI | -8.15 | -0.29 | -3.75 | -0.41 |  | -5.76 | -0.20 | -2.51 | -0.27 |  |
| Upper bound 95%CI | 24.80 | 1.06 | 12.57 | 1.70 |  | 17.49 | 0.76 | 8.36 | 1.16 |  |
| **Belgium** | 4.02 | 0.18 | 1.67 | 0.22 | 1.21 | 2.67 | 0.12 | 1.05 | 0.14 | 1.14 |
| Lower bound 95%CI | -3.17 | -0.13 | -1.31 | -0.16 |  | -2.16 | -0.09 | -0.83 | -0.10 |  |
| Upper bound 95%CI | 10.44 | 0.53 | 4.54 | 0.67 |  | 7.13 | 0.37 | 2.87 | 0.44 |  |
| **Bulgaria** | 13.35 | 0.29 | 7.14 | 0.28 | 0.95 | 12.87 | 0.29 | 6.37 | 0.25 | 0.86 |
| Lower bound 95%CI | -10.86 | -0.22 | -5.56 | -0.20 |  | -10.52 | -0.22 | -4.87 | -0.17 |  |
| Upper bound 95%CI | 34.52 | 0.83 | 19.65 | 0.84 |  | 34.67 | 0.90 | 17.43 | 0.78 |  |
| **Croatia** | 15.05 | 0.41 | 8.47 | 0.49 | 1.20 | 11.21 | 0.29 | 6.50 | 0.34 | 1.15 |
| Lower bound 95%CI | -12.75 | -0.30 | -6.70 | -0.34 |  | -9.10 | -0.21 | -5.15 | -0.23 |  |
| Upper bound 95%CI | 38.71 | 1.19 | 22.79 | 1.50 |  | 28.68 | 0.86 | 16.91 | 1.00 |  |
| **Cyprus** | 9.37 | 0.60 | 3.99 | 0.81 | 1.34 | 6.50 | 0.36 | 2.91 | 0.46 | 1.29 |
| Lower bound 95%CI | -7.83 | -0.44 | -2.95 | -0.50 |  | -4.98 | -0.24 | -2.09 | -0.28 |  |
| Upper bound 95%CI | 24.73 | 1.83 | 10.54 | 2.52 |  | 16.94 | 1.08 | 8.15 | 1.51 |  |
| **Czechia** | 17.54 | 0.42 | 9.29 | 0.40 | 0.95 | 12.38 | 0.29 | 6.31 | 0.24 | 0.82 |
| Lower bound 95%CI | -14.84 | -0.32 | -7.62 | -0.30 |  | -10.10 | -0.21 | -4.99 | -0.17 |  |
| Upper bound 95%CI | 45.33 | 1.21 | 24.54 | 1.17 |  | 31.73 | 0.85 | 16.81 | 0.72 |  |
| **Denmark** | 6.90 | 0.44 | 3.18 | 0.55 | 1.23 | 4.61 | 0.28 | 1.91 | 0.34 | 1.20 |
| Lower bound 95%CI | -6.04 | -0.34 | -2.71 | -0.41 |  | -3.89 | -0.21 | -1.61 | -0.25 |  |
| Upper bound 95%CI | 17.43 | 1.26 | 8.39 | 1.62 |  | 11.64 | 0.81 | 5.06 | 1.02 |  |
| **Estonia** | 21.53 | 0.46 | 9.46 | 0.31 | 0.69 | 11.39 | 0.23 | 4.62 | 0.14 | 0.63 |
| Lower bound 95%CI | -18.43 | -0.36 | -7.81 | -0.24 |  | -9.64 | -0.17 | -3.72 | -0.10 |  |
| Upper bound 95%CI | 55.65 | 1.28 | 25.47 | 0.92 |  | 30.39 | 0.68 | 12.33 | 0.43 |  |
| **Finland** | 14.12 | 0.68 | 5.98 | 0.72 | 1.07 | 9.52 | 0.39 | 3.98 | 0.44 | 1.11 |
| Lower bound 95%CI | -12.59 | -0.54 | -5.06 | -0.54 |  | -8.34 | -0.30 | -3.27 | -0.32 |  |
| Upper bound 95%CI | 36.28 | 1.94 | 15.85 | 2.15 |  | 24.88 | 1.17 | 10.73 | 1.36 |  |
| **France** | 3.19 | 0.12 | 1.15 | 0.14 | 1.14 | 2.29 | 0.09 | 0.80 | 0.10 | 1.08 |
| Lower bound 95%CI | -2.61 | -0.09 | -0.92 | -0.10 |  | -1.84 | -0.06 | -0.60 | -0.06 |  |
| Upper bound 95%CI | 8.50 | 0.36 | 3.11 | 0.42 |  | 5.97 | 0.27 | 2.17 | 0.30 |  |
| **Germany** | 9.85 | 0.32 | 4.62 | 0.48 | 1.48 | 7.52 | 0.29 | 3.16 | 0.36 | 1.23 |
| Lower bound 95%CI | -8.57 | -0.26 | -3.87 | -0.36 |  | -6.60 | -0.23 | -2.65 | -0.26 |  |
| Upper bound 95%CI | 25.13 | 0.90 | 12.19 | 1.38 |  | 19.25 | 0.84 | 8.14 | 1.05 |  |
| **Greece** | 4.98 | 0.25 | 2.90 | 0.50 | 2.00 | 4.09 | 0.22 | 1.86 | 0.32 | 1.43 |
| Lower bound 95%CI | -3.93 | -0.18 | -2.26 | -0.34 |  | -3.26 | -0.16 | -1.44 | -0.21 |  |
| Upper bound 95%CI | 13.10 | 0.73 | 7.84 | 1.53 |  | 10.70 | 0.67 | 5.16 | 1.04 |  |
| **Hungary** | 9.74 | 0.20 | 5.25 | 0.19 | 0.95 | 8.08 | 0.18 | 4.26 | 0.16 | 0.90 |
| Lower bound 95%CI | -7.86 | -0.15 | -4.14 | -0.14 |  | -6.88 | -0.14 | -3.29 | -0.11 |  |
| Upper bound 95%CI | 25.62 | 0.57 | 14.52 | 0.57 |  | 20.77 | 0.51 | 11.51 | 0.48 |  |
| **Ireland** | 7.22 | 0.33 | 3.17 | 0.43 | 1.31 | 4.21 | 0.19 | 1.81 | 0.24 | 1.25 |
| Lower bound 95%CI | -5.99 | -0.24 | -2.53 | -0.31 |  | -3.51 | -0.14 | -1.44 | -0.17 |  |
| Upper bound 95%CI | 19.20 | 0.97 | 8.63 | 1.32 |  | 10.90 | 0.57 | 4.83 | 0.74 |  |
| **Italy** | 2.76 | 0.11 | 1.21 | 0.13 | 1.18 | 2.28 | 0.10 | 0.94 | 0.11 | 1.09 |
| Lower bound 95%CI | -2.14 | -0.08 | -0.88 | -0.08 |  | -1.70 | -0.06 | -0.70 | -0.07 |  |
| Upper bound 95%CI | 7.59 | 0.36 | 3.31 | 0.42 |  | 6.13 | 0.32 | 2.66 | 0.36 |  |
| **Latvia** | 31.67 | 0.79 | 14.45 | 0.72 | 0.90 | 21.01 | 0.50 | 9.16 | 0.39 | 0.78 |
| Lower bound 95%CI | -28.06 | -0.64 | -12.09 | -0.53 |  | -17.42 | -0.37 | -7.40 | -0.27 |  |
| Upper bound 95%CI | 79.69 | 2.19 | 38.48 | 2.15 |  | 54.21 | 1.45 | 23.88 | 1.15 |  |
| **Lithuania** | 27.84 | 0.68 | 12.59 | 0.62 | 0.92 | 18.67 | 0.45 | 8.54 | 0.37 | 0.82 |
| Lower bound 95%CI | -23.87 | -0.54 | -10.31 | -0.46 |  | -15.42 | -0.33 | -6.81 | -0.26 |  |
| Upper bound 95%CI | 71.32 | 1.86 | 32.69 | 1.80 |  | 48.72 | 1.31 | 23.22 | 1.13 |  |
| **Luxembourg** | 7.84 | 0.50 | 3.38 | 0.55 | 1.11 | 5.33 | 0.29 | 2.24 | 0.33 | 1.12 |
| Lower bound 95%CI | -6.72 | -0.38 | -2.77 | -0.38 |  | -4.70 | -0.22 | -1.82 | -0.23 |  |
| Upper bound 95%CI | 20.18 | 1.45 | 8.79 | 1.69 |  | 13.56 | 0.85 | 5.96 | 1.02 |  |
| **Malta** | 5.95 | 0.32 | 3.14 | 0.54 | 1.66 | 3.66 | 0.19 | 2.22 | 0.35 | 1.85 |
| Lower bound 95%CI | -4.69 | -0.22 | -2.47 | -0.36 |  | -2.94 | -0.13 | -1.71 | -0.23 |  |
| Upper bound 95%CI | 15.46 | 0.94 | 8.68 | 1.72 |  | 9.52 | 0.56 | 6.01 | 1.09 |  |
| **Netherlands** | 4.56 | 0.19 | 1.87 | 0.23 | 1.19 | 3.27 | 0.14 | 1.33 | 0.17 | 1.19 |
| Lower bound 95%CI | -3.83 | -0.15 | -1.44 | -0.16 |  | -2.66 | -0.10 | -1.05 | -0.12 |  |
| Upper bound 95%CI | 11.93 | 0.56 | 4.97 | 0.68 |  | 8.70 | 0.44 | 3.55 | 0.53 |  |
| **Poland** | 11.27 | 0.28 | 5.10 | 0.24 | 0.84 | 8.92 | 0.23 | 4.11 | 0.19 | 0.85 |
| Lower bound 95%CI | -9.15 | -0.20 | -3.89 | -0.16 |  | -7.48 | -0.16 | -3.10 | -0.12 |  |
| Upper bound 95%CI | 29.13 | 0.85 | 13.71 | 0.74 |  | 23.54 | 0.70 | 10.78 | 0.60 |  |
| **Portugal** | 2.73 | 0.19 | 1.32 | 0.27 | 1.46 | 2.44 | 0.17 | 1.00 | 0.19 | 1.12 |
| Lower bound 95%CI | -2.24 | -0.13 | -1.00 | -0.18 |  | -1.97 | -0.12 | -0.76 | -0.12 |  |
| Upper bound 95%CI | 7.12 | 0.56 | 3.63 | 0.87 |  | 6.34 | 0.51 | 2.76 | 0.64 |  |
| **Romania** | 14.86 | 0.38 | 8.19 | 0.37 | 0.97 | 12.29 | 0.30 | 6.53 | 0.25 | 0.84 |
| Lower bound 95%CI | -12.14 | -0.28 | -6.59 | -0.27 |  | -10.14 | -0.22 | -5.36 | -0.18 |  |
| Upper bound 95%CI | 38.16 | 1.10 | 22.13 | 1.11 |  | 32.54 | 0.89 | 18.07 | 0.77 |  |
| **Slovakia** | 14.06 | 0.38 | 8.21 | 0.41 | 1.09 | 12.34 | 0.32 | 7.43 | 0.32 | 1.00 |
| Lower bound 95%CI | -11.58 | -0.28 | -6.18 | -0.28 |  | -10.18 | -0.24 | -6.11 | -0.23 |  |
| Upper bound 95%CI | 37.12 | 1.11 | 22.86 | 1.28 |  | 32.68 | 0.96 | 19.94 | 0.96 |  |
| **Slovenia** | 7.06 | 0.21 | 2.96 | 0.15 | 0.73 | 5.07 | 0.12 | 1.84 | 0.07 | 0.58 |
| Lower bound 95%CI | -6.17 | -0.16 | -2.30 | -0.10 |  | -4.30 | -0.09 | -1.40 | -0.05 |  |
| Upper bound 95%CI | 18.11 | 0.60 | 8.23 | 0.47 |  | 12.96 | 0.37 | 4.83 | 0.22 |  |
| **Spain** | 2.32 | 0.11 | 0.92 | 0.13 | 1.15 | 1.90 | 0.10 | 0.68 | 0.10 | 1.03 |
| Lower bound 95%CI | -1.83 | -0.08 | -0.70 | -0.09 |  | -1.43 | -0.07 | -0.51 | -0.07 |  |
| Upper bound 95%CI | 6.25 | 0.33 | 2.54 | 0.39 |  | 5.06 | 0.29 | 1.88 | 0.32 |  |
| **Sweden** | 6.80 | 0.35 | 3.24 | 0.37 | 1.06 | 4.14 | 0.20 | 2.01 | 0.23 | 1.12 |
| Lower bound 95%CI | -5.41 | -0.24 | -2.58 | -0.25 |  | -3.23 | -0.13 | -1.64 | -0.16 |  |
| Upper bound 95%CI | 18.04 | 1.07 | 8.61 | 1.14 |  | 11.04 | 0.63 | 5.42 | 0.72 |  |
| **European Union** | 24.96 | 0.89 | 13.15 | 1.14 | 1.28 | 19.89 | 0.74 | 10.91 | 0.94 | 1.26 |
| Lower bound 95%CI | -21.28 | -0.69 | -10.69 | -0.83 |  | -17.46 | -0.58 | -8.87 | -0.67 |  |
| Upper bound 95%CI | 64.17 | 2.53 | 35.00 | 3.39 |  | 51.22 | 2.17 | 28.61 | 2.80 |  |

Data and definitions from Global Burden of Disease Database. 2021

*Each value corresponds to mortality rates per 100.000 inhabitants (95% Uncertainty Interval)

Abbreviation: CFI, case-fatality index

| Appendix Table 19. Age-standardised mortality rates and case fatality indexes for IHD attributable to Diet low in nuts and seeds.  2011 , 2021 | | | | | | | | | | |
| --- | --- | --- | --- | --- | --- | --- | --- | --- | --- | --- |
| **Country** | **2011** | | | | | **2021** | | | | |
|  | **Men** | | **Women** | | **CFI Ratio** | **Men** | | **Women** | | **CFI Ratio** |
|  | **Mortality rate** | **CFI** | **Mortality rate** | **CFI** |  | **Mortality rate** | **CFI** | **Mortality rate** | **CFI** |  |
| **Austria** | 4.58 | 0.18 | 2.12 | 0.26 | 1.47 | 3.17 | 0.12 | 1.36 | 0.17 | 1.36 |
| Lower bound 95%CI | 1.17 | 0.04 | 0.51 | 0.06 |  | 0.82 | 0.03 | 0.33 | 0.04 |  |
| Upper bound 95%CI | 8.28 | 0.35 | 4.04 | 0.55 |  | 5.97 | 0.26 | 2.77 | 0.39 |  |
| **Belgium** | 3.45 | 0.16 | 1.38 | 0.18 | 1.16 | 2.24 | 0.10 | 0.85 | 0.11 | 1.10 |
| Lower bound 95%CI | 0.90 | 0.04 | 0.36 | 0.04 |  | 0.61 | 0.02 | 0.23 | 0.03 |  |
| Upper bound 95%CI | 6.13 | 0.31 | 2.64 | 0.39 |  | 4.13 | 0.22 | 1.59 | 0.24 |  |
| **Bulgaria** | 27.66 | 0.60 | 14.85 | 0.58 | 0.96 | 22.78 | 0.52 | 11.54 | 0.46 | 0.88 |
| Lower bound 95%CI | 7.83 | 0.16 | 4.01 | 0.14 |  | 6.39 | 0.13 | 3.02 | 0.11 |  |
| Upper bound 95%CI | 45.99 | 1.11 | 25.35 | 1.08 |  | 37.79 | 0.98 | 19.94 | 0.89 |  |
| **Croatia** | 15.58 | 0.42 | 9.09 | 0.53 | 1.25 | 12.11 | 0.32 | 7.32 | 0.38 | 1.20 |
| Lower bound 95%CI | 4.22 | 0.10 | 2.37 | 0.12 |  | 3.17 | 0.07 | 1.86 | 0.08 |  |
| Upper bound 95%CI | 26.24 | 0.81 | 15.84 | 1.05 |  | 20.53 | 0.62 | 12.81 | 0.76 |  |
| **Cyprus** | 4.71 | 0.30 | 2.03 | 0.41 | 1.36 | 3.26 | 0.18 | 1.50 | 0.24 | 1.32 |
| Lower bound 95%CI | 1.27 | 0.07 | 0.54 | 0.09 |  | 0.82 | 0.04 | 0.38 | 0.05 |  |
| Upper bound 95%CI | 9.18 | 0.68 | 3.90 | 0.93 |  | 6.30 | 0.40 | 3.16 | 0.59 |  |
| **Czechia** | 17.65 | 0.42 | 9.40 | 0.41 | 0.96 | 12.25 | 0.29 | 6.24 | 0.24 | 0.82 |
| Lower bound 95%CI | 4.95 | 0.11 | 2.42 | 0.09 |  | 3.37 | 0.07 | 1.55 | 0.05 |  |
| Upper bound 95%CI | 29.16 | 0.78 | 16.34 | 0.78 |  | 21.38 | 0.57 | 10.99 | 0.47 |  |
| **Denmark** | 5.23 | 0.34 | 2.37 | 0.41 | 1.21 | 3.41 | 0.21 | 1.40 | 0.25 | 1.19 |
| Lower bound 95%CI | 1.39 | 0.08 | 0.64 | 0.10 |  | 0.89 | 0.05 | 0.36 | 0.05 |  |
| Upper bound 95%CI | 8.85 | 0.64 | 4.21 | 0.81 |  | 5.80 | 0.40 | 2.53 | 0.51 |  |
| **Estonia** | 27.63 | 0.59 | 12.35 | 0.41 | 0.70 | 13.83 | 0.28 | 5.79 | 0.18 | 0.65 |
| Lower bound 95%CI | 7.34 | 0.14 | 3.22 | 0.10 |  | 3.50 | 0.06 | 1.50 | 0.04 |  |
| Upper bound 95%CI | 45.62 | 1.05 | 21.65 | 0.78 |  | 23.39 | 0.53 | 10.02 | 0.35 |  |
| **Finland** | 14.25 | 0.68 | 6.17 | 0.75 | 1.09 | 9.43 | 0.39 | 4.01 | 0.44 | 1.13 |
| Lower bound 95%CI | 4.04 | 0.17 | 1.62 | 0.17 |  | 2.67 | 0.10 | 1.02 | 0.10 |  |
| Upper bound 95%CI | 23.98 | 1.28 | 10.43 | 1.41 |  | 15.91 | 0.75 | 6.90 | 0.87 |  |
| **France** | 4.28 | 0.16 | 1.52 | 0.18 | 1.12 | 2.86 | 0.11 | 1.00 | 0.12 | 1.08 |
| Lower bound 95%CI | 1.21 | 0.04 | 0.41 | 0.04 |  | 0.84 | 0.03 | 0.26 | 0.03 |  |
| Upper bound 95%CI | 7.20 | 0.30 | 2.73 | 0.37 |  | 4.98 | 0.22 | 1.80 | 0.25 |  |
| **Germany** | 4.11 | 0.13 | 1.81 | 0.19 | 1.39 | 3.18 | 0.12 | 1.22 | 0.14 | 1.12 |
| Lower bound 95%CI | 1.13 | 0.03 | 0.43 | 0.04 |  | 0.86 | 0.03 | 0.32 | 0.03 |  |
| Upper bound 95%CI | 7.57 | 0.27 | 3.30 | 0.37 |  | 5.87 | 0.26 | 2.20 | 0.28 |  |
| **Greece** | 3.12 | 0.16 | 1.96 | 0.34 | 2.16 | 2.85 | 0.16 | 1.38 | 0.24 | 1.52 |
| Lower bound 95%CI | 0.94 | 0.04 | 0.52 | 0.08 |  | 0.79 | 0.04 | 0.36 | 0.05 |  |
| Upper bound 95%CI | 5.39 | 0.30 | 3.91 | 0.76 |  | 5.09 | 0.32 | 2.72 | 0.55 |  |
| **Hungary** | 31.67 | 0.65 | 17.01 | 0.61 | 0.95 | 23.65 | 0.52 | 12.64 | 0.47 | 0.91 |
| Lower bound 95%CI | 8.99 | 0.17 | 4.79 | 0.16 |  | 6.60 | 0.13 | 3.49 | 0.12 |  |
| Upper bound 95%CI | 52.48 | 1.17 | 28.60 | 1.13 |  | 38.82 | 0.95 | 21.17 | 0.88 |  |
| **Ireland** | 8.84 | 0.40 | 4.04 | 0.55 | 1.36 | 4.48 | 0.21 | 2.04 | 0.27 | 1.32 |
| Lower bound 95%CI | 2.47 | 0.10 | 1.06 | 0.13 |  | 1.22 | 0.05 | 0.55 | 0.06 |  |
| Upper bound 95%CI | 14.70 | 0.74 | 7.20 | 1.10 |  | 7.74 | 0.41 | 3.65 | 0.56 |  |
| **Italy** | 5.41 | 0.22 | 2.23 | 0.25 | 1.11 | 4.09 | 0.18 | 1.64 | 0.19 | 1.06 |
| Lower bound 95%CI | 1.51 | 0.05 | 0.62 | 0.06 |  | 1.15 | 0.04 | 0.44 | 0.04 |  |
| Upper bound 95%CI | 9.13 | 0.43 | 4.08 | 0.52 |  | 7.03 | 0.36 | 3.01 | 0.41 |  |
| **Latvia** | 32.30 | 0.81 | 15.13 | 0.75 | 0.92 | 21.35 | 0.51 | 9.55 | 0.40 | 0.80 |
| Lower bound 95%CI | 8.90 | 0.20 | 4.13 | 0.18 |  | 5.67 | 0.12 | 2.55 | 0.09 |  |
| Upper bound 95%CI | 53.83 | 1.48 | 25.93 | 1.45 |  | 36.08 | 0.96 | 16.62 | 0.80 |  |
| **Lithuania** | 36.22 | 0.88 | 17.31 | 0.86 | 0.97 | 24.37 | 0.59 | 11.74 | 0.51 | 0.87 |
| Lower bound 95%CI | 10.03 | 0.23 | 4.81 | 0.21 |  | 6.40 | 0.14 | 3.06 | 0.12 |  |
| Upper bound 95%CI | 59.10 | 1.55 | 29.33 | 1.62 |  | 41.51 | 1.12 | 20.21 | 0.98 |  |
| **Luxembourg** | 9.60 | 0.61 | 4.11 | 0.67 | 1.10 | 6.38 | 0.35 | 2.65 | 0.39 | 1.11 |
| Lower bound 95%CI | 2.66 | 0.15 | 1.14 | 0.16 |  | 1.78 | 0.09 | 0.70 | 0.09 |  |
| Upper bound 95%CI | 15.69 | 1.13 | 6.91 | 1.33 |  | 10.80 | 0.67 | 4.58 | 0.78 |  |
| **Malta** | 6.83 | 0.37 | 3.54 | 0.60 | 1.63 | 3.61 | 0.18 | 2.10 | 0.33 | 1.78 |
| Lower bound 95%CI | 1.78 | 0.08 | 0.91 | 0.13 |  | 0.95 | 0.04 | 0.55 | 0.07 |  |
| Upper bound 95%CI | 11.44 | 0.70 | 6.30 | 1.25 |  | 6.26 | 0.37 | 3.89 | 0.71 |  |
| **Netherlands** | 2.89 | 0.12 | 1.18 | 0.14 | 1.19 | 2.06 | 0.09 | 0.81 | 0.11 | 1.15 |
| Lower bound 95%CI | 0.73 | 0.03 | 0.32 | 0.04 |  | 0.51 | 0.02 | 0.20 | 0.02 |  |
| Upper bound 95%CI | 4.98 | 0.23 | 2.18 | 0.30 |  | 3.72 | 0.19 | 1.61 | 0.24 |  |
| **Poland** | 22.61 | 0.57 | 10.75 | 0.50 | 0.88 | 17.71 | 0.45 | 8.62 | 0.40 | 0.90 |
| Lower bound 95%CI | 6.57 | 0.15 | 3.00 | 0.12 |  | 5.25 | 0.11 | 2.30 | 0.09 |  |
| Upper bound 95%CI | 36.73 | 1.07 | 17.86 | 0.97 |  | 29.58 | 0.88 | 14.55 | 0.81 |  |
| **Portugal** | 4.29 | 0.29 | 2.06 | 0.42 | 1.45 | 3.81 | 0.26 | 1.55 | 0.29 | 1.11 |
| Lower bound 95%CI | 1.16 | 0.07 | 0.54 | 0.09 |  | 1.05 | 0.06 | 0.41 | 0.06 |  |
| Upper bound 95%CI | 7.22 | 0.57 | 3.52 | 0.84 |  | 6.33 | 0.51 | 2.76 | 0.64 |  |
| **Romania** | 27.42 | 0.71 | 15.70 | 0.71 | 1.00 | 22.27 | 0.54 | 12.25 | 0.47 | 0.87 |
| Lower bound 95%CI | 7.78 | 0.18 | 4.35 | 0.18 |  | 6.42 | 0.14 | 3.52 | 0.12 |  |
| Upper bound 95%CI | 44.65 | 1.29 | 26.61 | 1.34 |  | 36.88 | 1.01 | 20.44 | 0.87 |  |
| **Slovakia** | 21.30 | 0.58 | 12.14 | 0.61 | 1.07 | 15.10 | 0.39 | 9.20 | 0.40 | 1.01 |
| Lower bound 95%CI | 5.95 | 0.14 | 3.16 | 0.14 |  | 4.33 | 0.10 | 2.50 | 0.10 |  |
| Upper bound 95%CI | 35.74 | 1.07 | 21.09 | 1.18 |  | 26.42 | 0.78 | 16.77 | 0.81 |  |
| **Slovenia** | 5.98 | 0.17 | 2.54 | 0.13 | 0.73 | 4.40 | 0.11 | 1.59 | 0.06 | 0.58 |
| Lower bound 95%CI | 1.63 | 0.04 | 0.67 | 0.03 |  | 1.18 | 0.03 | 0.44 | 0.02 |  |
| Upper bound 95%CI | 10.25 | 0.34 | 4.55 | 0.26 |  | 7.62 | 0.22 | 2.80 | 0.13 |  |
| **Spain** | 3.03 | 0.14 | 1.19 | 0.16 | 1.14 | 2.22 | 0.11 | 0.79 | 0.12 | 1.02 |
| Lower bound 95%CI | 0.85 | 0.04 | 0.32 | 0.04 |  | 0.59 | 0.03 | 0.20 | 0.03 |  |
| Upper bound 95%CI | 5.22 | 0.27 | 2.24 | 0.35 |  | 3.98 | 0.23 | 1.54 | 0.26 |  |
| **Sweden** | 6.03 | 0.31 | 2.88 | 0.33 | 1.06 | 3.21 | 0.16 | 1.60 | 0.18 | 1.15 |
| Lower bound 95%CI | 1.62 | 0.07 | 0.85 | 0.08 |  | 0.88 | 0.04 | 0.46 | 0.04 |  |
| Upper bound 95%CI | 10.34 | 0.61 | 5.33 | 0.71 |  | 5.82 | 0.33 | 2.98 | 0.39 |  |
| **European Union** | 41.80 | 1.49 | 21.52 | 1.87 | 1.25 | 29.36 | 1.10 | 15.94 | 1.38 | 1.25 |
| Lower bound 95%CI | 11.90 | 0.39 | 6.10 | 0.47 |  | 8.15 | 0.27 | 4.42 | 0.33 |  |
| Upper bound 95%CI | 68.92 | 2.72 | 35.88 | 3.48 |  | 48.68 | 2.06 | 27.10 | 2.65 |  |

Data and definitions from Global Burden of Disease Database. 2021

*Each value corresponds to mortality rates per 100.000 inhabitants (95% Uncertainty Interval)

Abbreviation: CFI, case-fatality index

| Appendix Table 20. Age-standardised mortality rates and case fatality indexes for IHD attributable to Diet low in omega-6 polyunsaturated fatty acids. 2011 , 2021 | | | | | | | | | | |
| --- | --- | --- | --- | --- | --- | --- | --- | --- | --- | --- |
| **Country** | **2011** | | | | | **2021** | | | | |
|  | **Men** | | **Women** | | **CFI Ratio** | **Men** | | **Women** | | **CFI Ratio** |
|  | **Mortality rate** | **CFI** | **Mortality rate** | **CFI** |  | **Mortality rate** | **CFI** | **Mortality rate** | **CFI** |  |
| **Austria** | 9.97 | 0.38 | 5.29 | 0.64 | 1.68 | 7.16 | 0.28 | 3.58 | 0.44 | 1.59 |
| Lower bound 95%CI | -27.53 | -0.97 | -13.59 | -1.50 |  | -19.43 | -0.67 | -9.38 | -1.02 |  |
| Upper bound 95%CI | 37.93 | 1.62 | 20.58 | 2.78 |  | 27.11 | 1.18 | 14.12 | 1.97 |  |
| **Belgium** | 3.67 | 0.17 | 1.85 | 0.25 | 1.46 | 2.47 | 0.11 | 1.17 | 0.15 | 1.37 |
| Lower bound 95%CI | -8.65 | -0.36 | -4.17 | -0.50 |  | -5.75 | -0.23 | -2.56 | -0.30 |  |
| Upper bound 95%CI | 14.45 | 0.73 | 7.44 | 1.11 |  | 9.75 | 0.51 | 4.80 | 0.73 |  |
| **Bulgaria** | 20.45 | 0.45 | 11.95 | 0.46 | 1.04 | 17.02 | 0.39 | 9.58 | 0.38 | 0.98 |
| Lower bound 95%CI | -54.41 | -1.08 | -29.89 | -1.06 |  | -43.14 | -0.89 | -23.96 | -0.85 |  |
| Upper bound 95%CI | 77.92 | 1.88 | 46.23 | 1.98 |  | 64.51 | 1.67 | 36.17 | 1.61 |  |
| **Croatia** | 12.35 | 0.34 | 8.06 | 0.47 | 1.40 | 9.26 | 0.24 | 6.30 | 0.33 | 1.35 |
| Lower bound 95%CI | -31.66 | -0.75 | -20.78 | -1.05 |  | -22.96 | -0.53 | -15.04 | -0.68 |  |
| Upper bound 95%CI | 47.27 | 1.46 | 31.58 | 2.08 |  | 35.31 | 1.06 | 23.91 | 1.41 |  |
| **Cyprus** | 8.57 | 0.55 | 4.11 | 0.83 | 1.51 | 5.85 | 0.32 | 2.96 | 0.47 | 1.46 |
| Lower bound 95%CI | -21.20 | -1.19 | -9.14 | -1.55 |  | -14.34 | -0.68 | -6.42 | -0.86 |  |
| Upper bound 95%CI | 32.91 | 2.43 | 16.24 | 3.88 |  | 22.85 | 1.46 | 11.94 | 2.22 |  |
| **Czechia** | 18.58 | 0.45 | 10.59 | 0.46 | 1.03 | 13.33 | 0.32 | 7.33 | 0.28 | 0.88 |
| Lower bound 95%CI | -51.82 | -1.13 | -28.09 | -1.09 |  | -37.25 | -0.79 | -19.56 | -0.67 |  |
| Upper bound 95%CI | 71.33 | 1.90 | 40.85 | 1.96 |  | 49.85 | 1.34 | 28.44 | 1.22 |  |
| **Denmark** | 6.18 | 0.40 | 3.04 | 0.52 | 1.31 | 4.13 | 0.25 | 1.84 | 0.32 | 1.29 |
| Lower bound 95%CI | -16.50 | -0.93 | -8.10 | -1.23 |  | -10.80 | -0.58 | -4.75 | -0.72 |  |
| Upper bound 95%CI | 23.31 | 1.68 | 11.89 | 2.30 |  | 15.30 | 1.06 | 7.18 | 1.45 |  |
| **Estonia** | 21.28 | 0.45 | 9.86 | 0.33 | 0.72 | 11.05 | 0.22 | 4.74 | 0.15 | 0.67 |
| Lower bound 95%CI | -59.68 | -1.16 | -26.52 | -0.80 |  | -29.77 | -0.53 | -12.35 | -0.34 |  |
| Upper bound 95%CI | 80.40 | 1.85 | 38.70 | 1.40 |  | 42.61 | 0.96 | 18.49 | 0.64 |  |
| **Finland** | 13.55 | 0.65 | 6.04 | 0.73 | 1.12 | 9.36 | 0.39 | 4.12 | 0.45 | 1.17 |
| Lower bound 95%CI | -38.55 | -1.66 | -16.18 | -1.73 |  | -26.03 | -0.95 | -10.81 | -1.04 |  |
| Upper bound 95%CI | 51.33 | 2.75 | 23.17 | 3.14 |  | 35.70 | 1.69 | 15.97 | 2.02 |  |
| **France** | 4.29 | 0.16 | 1.78 | 0.22 | 1.31 | 3.00 | 0.12 | 1.22 | 0.15 | 1.26 |
| Lower bound 95%CI | -11.28 | -0.39 | -4.54 | -0.49 |  | -7.68 | -0.27 | -3.04 | -0.33 |  |
| Upper bound 95%CI | 16.24 | 0.68 | 6.86 | 0.92 |  | 11.21 | 0.50 | 4.77 | 0.66 |  |
| **Germany** | 7.65 | 0.25 | 3.91 | 0.41 | 1.61 | 5.81 | 0.22 | 2.65 | 0.30 | 1.34 |
| Lower bound 95%CI | -19.70 | -0.59 | -9.62 | -0.90 |  | -14.89 | -0.51 | -6.58 | -0.65 |  |
| Upper bound 95%CI | 29.61 | 1.06 | 15.46 | 1.75 |  | 21.95 | 0.96 | 10.29 | 1.32 |  |
| **Greece** | 11.06 | 0.55 | 6.83 | 1.17 | 2.12 | 8.34 | 0.46 | 4.14 | 0.72 | 1.56 |
| Lower bound 95%CI | -31.60 | -1.41 | -18.28 | -2.72 |  | -24.21 | -1.17 | -10.85 | -1.58 |  |
| Upper bound 95%CI | 42.52 | 2.36 | 26.28 | 5.14 |  | 31.58 | 1.97 | 15.97 | 3.23 |  |
| **Hungary** | 19.18 | 0.39 | 11.10 | 0.40 | 1.02 | 14.45 | 0.32 | 8.34 | 0.31 | 0.98 |
| Lower bound 95%CI | -51.98 | -0.96 | -28.65 | -0.94 |  | -39.09 | -0.77 | -22.17 | -0.74 |  |
| Upper bound 95%CI | 73.38 | 1.64 | 43.38 | 1.71 |  | 53.79 | 1.31 | 31.93 | 1.32 |  |
| **Ireland** | 9.29 | 0.42 | 4.43 | 0.60 | 1.42 | 5.36 | 0.25 | 2.53 | 0.34 | 1.37 |
| Lower bound 95%CI | -25.84 | -1.06 | -11.67 | -1.41 |  | -14.32 | -0.58 | -6.65 | -0.77 |  |
| Upper bound 95%CI | 35.54 | 1.79 | 17.27 | 2.65 |  | 20.39 | 1.07 | 9.75 | 1.49 |  |
| **Italy** | 5.06 | 0.21 | 2.49 | 0.28 | 1.33 | 3.70 | 0.16 | 1.77 | 0.20 | 1.27 |
| Lower bound 95%CI | -12.86 | -0.46 | -5.96 | -0.57 |  | -8.94 | -0.32 | -4.14 | -0.40 |  |
| Upper bound 95%CI | 19.83 | 0.94 | 9.56 | 1.21 |  | 14.40 | 0.75 | 6.75 | 0.92 |  |
| **Latvia** | 24.01 | 0.60 | 12.02 | 0.59 | 0.99 | 15.48 | 0.37 | 7.42 | 0.31 | 0.86 |
| Lower bound 95%CI | -62.98 | -1.44 | -30.96 | -1.36 |  | -38.15 | -0.81 | -18.38 | -0.68 |  |
| Upper bound 95%CI | 91.61 | 2.51 | 47.01 | 2.63 |  | 59.79 | 1.60 | 28.71 | 1.38 |  |
| **Lithuania** | 30.19 | 0.74 | 15.13 | 0.75 | 1.02 | 20.44 | 0.49 | 10.40 | 0.45 | 0.92 |
| Lower bound 95%CI | -83.93 | -1.89 | -39.59 | -1.76 |  | -53.21 | -1.15 | -25.50 | -0.98 |  |
| Upper bound 95%CI | 113.72 | 2.97 | 58.31 | 3.22 |  | 78.11 | 2.11 | 40.94 | 1.99 |  |
| **Luxembourg** | 6.87 | 0.44 | 3.15 | 0.51 | 1.18 | 4.75 | 0.26 | 2.14 | 0.31 | 1.21 |
| Lower bound 95%CI | -18.50 | -1.04 | -8.34 | -1.15 |  | -12.84 | -0.61 | -5.41 | -0.68 |  |
| Upper bound 95%CI | 26.12 | 1.88 | 12.20 | 2.35 |  | 18.22 | 1.14 | 8.32 | 1.42 |  |
| **Malta** | 8.69 | 0.47 | 5.05 | 0.86 | 1.83 | 4.57 | 0.23 | 3.14 | 0.49 | 2.10 |
| Lower bound 95%CI | -21.53 | -1.02 | -12.45 | -1.82 |  | -11.25 | -0.50 | -7.57 | -1.02 |  |
| Upper bound 95%CI | 34.00 | 2.08 | 19.53 | 3.87 |  | 17.44 | 1.03 | 12.13 | 2.21 |  |
| **Netherlands** | 3.73 | 0.16 | 1.77 | 0.22 | 1.38 | 2.69 | 0.12 | 1.25 | 0.16 | 1.36 |
| Lower bound 95%CI | -8.83 | -0.34 | -4.33 | -0.47 |  | -6.08 | -0.24 | -2.93 | -0.33 |  |
| Upper bound 95%CI | 14.26 | 0.66 | 6.93 | 0.95 |  | 10.33 | 0.52 | 4.90 | 0.73 |  |
| **Poland** | 16.57 | 0.42 | 7.99 | 0.37 | 0.89 | 13.02 | 0.33 | 6.45 | 0.30 | 0.92 |
| Lower bound 95%CI | -47.56 | -1.05 | -21.67 | -0.88 |  | -37.36 | -0.81 | -17.43 | -0.69 |  |
| Upper bound 95%CI | 62.91 | 1.83 | 30.97 | 1.68 |  | 49.72 | 1.48 | 24.72 | 1.38 |  |
| **Portugal** | 4.50 | 0.31 | 2.37 | 0.49 | 1.59 | 3.88 | 0.27 | 1.76 | 0.33 | 1.24 |
| Lower bound 95%CI | -12.13 | -0.72 | -5.93 | -1.04 |  | -10.57 | -0.63 | -4.47 | -0.69 |  |
| Upper bound 95%CI | 17.10 | 1.34 | 9.17 | 2.20 |  | 14.55 | 1.16 | 6.89 | 1.59 |  |
| **Romania** | 19.54 | 0.51 | 11.81 | 0.54 | 1.06 | 16.10 | 0.39 | 9.39 | 0.36 | 0.92 |
| Lower bound 95%CI | -56.61 | -1.31 | -33.03 | -1.35 |  | -47.58 | -1.03 | -25.97 | -0.89 |  |
| Upper bound 95%CI | 74.69 | 2.16 | 45.31 | 2.28 |  | 61.78 | 1.70 | 36.16 | 1.54 |  |
| **Slovakia** | 21.97 | 0.59 | 13.32 | 0.67 | 1.13 | 16.45 | 0.43 | 10.76 | 0.46 | 1.09 |
| Lower bound 95%CI | -60.21 | -1.45 | -34.81 | -1.57 |  | -45.75 | -1.06 | -28.38 | -1.09 |  |
| Upper bound 95%CI | 82.64 | 2.48 | 52.11 | 2.92 |  | 62.01 | 1.82 | 40.50 | 1.95 |  |
| **Slovenia** | 6.55 | 0.19 | 3.06 | 0.15 | 0.81 | 4.82 | 0.12 | 1.95 | 0.08 | 0.65 |
| Lower bound 95%CI | -17.71 | -0.45 | -7.59 | -0.34 |  | -13.13 | -0.29 | -5.13 | -0.18 |  |
| Upper bound 95%CI | 25.14 | 0.83 | 12.23 | 0.70 |  | 17.62 | 0.50 | 7.43 | 0.33 |  |
| **Spain** | 4.84 | 0.23 | 2.09 | 0.29 | 1.26 | 3.64 | 0.19 | 1.45 | 0.21 | 1.15 |
| Lower bound 95%CI | -12.95 | -0.56 | -5.36 | -0.66 |  | -9.53 | -0.44 | -3.70 | -0.48 |  |
| Upper bound 95%CI | 18.75 | 0.98 | 8.07 | 1.25 |  | 13.95 | 0.81 | 5.71 | 0.96 |  |
| **Sweden** | 8.72 | 0.45 | 4.47 | 0.51 | 1.14 | 5.18 | 0.25 | 2.73 | 0.31 | 1.22 |
| Lower bound 95%CI | -23.89 | -1.06 | -11.75 | -1.16 |  | -14.35 | -0.59 | -7.35 | -0.70 |  |
| Upper bound 95%CI | 33.88 | 2.01 | 17.58 | 2.33 |  | 20.16 | 1.15 | 10.72 | 1.42 |  |
| **European Union** | 31.70 | 1.13 | 17.69 | 1.54 | 1.36 | 23.09 | 0.86 | 13.58 | 1.17 | 1.35 |
| Lower bound 95%CI | -92.24 | -2.99 | -48.58 | -3.78 |  | -64.67 | -2.15 | -37.10 | -2.80 |  |
| Upper bound 95%CI | 120.87 | 4.77 | 68.24 | 6.61 |  | 88.31 | 3.74 | 51.61 | 5.05 |  |

Data and definitions from Global Burden of Disease Database. 2021

*Each value corresponds to mortality rates per 100.000 inhabitants (95% Uncertainty Interval)

Abbreviation: CFI, case-fatality index

| Appendix Table 21. Age-standardised mortality rates and case fatality indexes for IHD attributable to Diet low in seafood omega-3 fatty acids.  2011 , 2021 | | | | | | | | | | | |
| --- | --- | --- | --- | --- | --- | --- | --- | --- | --- | --- | --- |
| **Country** | **2011** | | | | | | **2021** | | | | |
|  | **Men** | | **Women** | |  |  | **Men** | | **Women** | |  |
|  | **Mortality Rates** | **CFI** | **Mortality Rates** | **CFI** | **CFI Ratio** | **Mortality Rates** | | **CFI** | **Mortality Rates** | **CFI** | **CFI Ratio** |
| **Austria** | 6.52 | 0.25 | 4.91 | 0.60 | 2.39 | 4.42 | | 0.17 | 3.21 | 0.39 | 2.31 |
| Lower bound 95%CI | 1.14 | 0.04 | 0.87 | 0.10 |  | 0.79 | | 0.03 | 0.55 | 0.06 |  |
| Upper bound 95%CI | 12.06 | 0.52 | 9.07 | 1.23 |  | 8.16 | | 0.35 | 5.98 | 0.83 |  |
| **Belgium** | 2.19 | 0.10 | 1.86 | 0.25 | 2.47 | 1.32 | | 0.06 | 1.11 | 0.15 | 2.43 |
| Lower bound 95%CI | 0.38 | 0.02 | 0.31 | 0.04 |  | 0.24 | | 0.01 | 0.19 | 0.02 |  |
| Upper bound 95%CI | 4.46 | 0.23 | 3.58 | 0.53 |  | 2.76 | | 0.14 | 2.17 | 0.33 |  |
| **Bulgaria** | 31.37 | 0.68 | 20.10 | 0.78 | 1.14 | 25.02 | | 0.57 | 16.05 | 0.64 | 1.11 |
| Lower bound 95%CI | 6.01 | 0.12 | 3.93 | 0.14 |  | 4.83 | | 0.10 | 2.91 | 0.10 |  |
| Upper bound 95%CI | 52.77 | 1.28 | 35.27 | 1.51 |  | 43.22 | | 1.12 | 28.41 | 1.26 |  |
| **Croatia** | 15.47 | 0.42 | 12.12 | 0.70 | 1.68 | 11.26 | | 0.29 | 9.26 | 0.48 | 1.63 |
| Lower bound 95%CI | 2.82 | 0.07 | 2.25 | 0.11 |  | 1.96 | | 0.04 | 1.57 | 0.07 |  |
| Upper bound 95%CI | 26.98 | 0.83 | 21.45 | 1.42 |  | 20.26 | | 0.61 | 16.72 | 0.99 |  |
| **Cyprus** | 7.78 | 0.50 | 5.33 | 1.08 | 2.15 | 5.30 | | 0.29 | 3.86 | 0.61 | 2.10 |
| Lower bound 95%CI | 1.35 | 0.08 | 0.91 | 0.15 |  | 0.91 | | 0.04 | 0.65 | 0.09 |  |
| Upper bound 95%CI | 14.64 | 1.08 | 10.18 | 2.43 |  | 9.79 | | 0.63 | 7.68 | 1.43 |  |
| **Czechia** | 16.72 | 0.40 | 12.22 | 0.53 | 1.31 | 11.20 | | 0.27 | 8.04 | 0.31 | 1.15 |
| Lower bound 95%CI | 3.08 | 0.07 | 2.19 | 0.09 |  | 1.94 | | 0.04 | 1.43 | 0.05 |  |
| Upper bound 95%CI | 29.58 | 0.79 | 21.85 | 1.05 |  | 20.30 | | 0.54 | 14.37 | 0.61 |  |
| **Denmark** | 2.45 | 0.16 | 2.13 | 0.37 | 2.32 | 1.52 | | 0.09 | 1.24 | 0.22 | 2.37 |
| Lower bound 95%CI | 0.46 | 0.03 | 0.39 | 0.06 |  | 0.28 | | 0.01 | 0.23 | 0.04 |  |
| Upper bound 95%CI | 4.96 | 0.36 | 4.08 | 0.79 |  | 3.10 | | 0.22 | 2.40 | 0.48 |  |
| **Estonia** | 7.29 | 0.15 | 6.73 | 0.22 | 1.44 | 3.13 | | 0.06 | 2.95 | 0.09 | 1.47 |
| Lower bound 95%CI | 1.27 | 0.02 | 1.20 | 0.04 |  | 0.50 | | 0.01 | 0.49 | 0.01 |  |
| Upper bound 95%CI | 13.92 | 0.32 | 12.68 | 0.46 |  | 6.46 | | 0.15 | 5.51 | 0.19 |  |
| **Finland** | 1.34 | 0.06 | 1.75 | 0.21 | 3.29 | 0.90 | | 0.04 | 1.16 | 0.13 | 3.43 |
| Lower bound 95%CI | 0.22 | 0.01 | 0.29 | 0.03 |  | 0.15 | | 0.01 | 0.19 | 0.02 |  |
| Upper bound 95%CI | 3.14 | 0.17 | 3.49 | 0.47 |  | 2.15 | | 0.10 | 2.45 | 0.31 |  |
| **France** | 0.93 | 0.04 | 0.87 | 0.11 | 2.96 | 0.63 | | 0.03 | 0.57 | 0.07 | 2.80 |
| Lower bound 95%CI | 0.16 | 0.01 | 0.16 | 0.02 |  | 0.11 | | 0.00 | 0.10 | 0.01 |  |
| Upper bound 95%CI | 1.95 | 0.08 | 1.72 | 0.23 |  | 1.39 | | 0.06 | 1.15 | 0.16 |  |
| **Germany** | 6.74 | 0.22 | 4.55 | 0.47 | 2.13 | 4.75 | | 0.18 | 2.99 | 0.34 | 1.84 |
| Lower bound 95%CI | 1.24 | 0.04 | 0.85 | 0.08 |  | 0.92 | | 0.03 | 0.58 | 0.06 |  |
| Upper bound 95%CI | 12.30 | 0.44 | 8.35 | 0.94 |  | 8.79 | | 0.38 | 5.50 | 0.71 |  |
| **Greece** | 6.69 | 0.33 | 6.05 | 1.03 | 3.10 | 5.53 | | 0.30 | 3.85 | 0.67 | 2.19 |
| Lower bound 95%CI | 1.26 | 0.06 | 1.11 | 0.17 |  | 1.05 | | 0.05 | 0.74 | 0.11 |  |
| Upper bound 95%CI | 12.24 | 0.68 | 11.32 | 2.21 |  | 9.72 | | 0.61 | 7.10 | 1.44 |  |
| **Hungary** | 19.87 | 0.41 | 13.94 | 0.50 | 1.24 | 14.29 | | 0.31 | 10.25 | 0.38 | 1.22 |
| Lower bound 95%CI | 3.64 | 0.07 | 2.49 | 0.08 |  | 2.72 | | 0.05 | 1.82 | 0.06 |  |
| Upper bound 95%CI | 34.59 | 0.77 | 24.65 | 0.97 |  | 24.86 | | 0.61 | 17.95 | 0.74 |  |
| **Ireland** | 2.99 | 0.14 | 2.64 | 0.36 | 2.63 | 1.21 | | 0.06 | 1.23 | 0.16 | 2.95 |
| Lower bound 95%CI | 0.55 | 0.02 | 0.49 | 0.06 |  | 0.21 | | 0.01 | 0.23 | 0.03 |  |
| Upper bound 95%CI | 5.92 | 0.30 | 5.07 | 0.78 |  | 2.45 | | 0.13 | 2.42 | 0.37 |  |
| **Italy** | 3.29 | 0.13 | 2.41 | 0.27 | 1.98 | 2.48 | | 0.11 | 1.74 | 0.20 | 1.86 |
| Lower bound 95%CI | 0.65 | 0.02 | 0.45 | 0.04 |  | 0.48 | | 0.02 | 0.33 | 0.03 |  |
| Upper bound 95%CI | 6.19 | 0.29 | 4.56 | 0.58 |  | 4.69 | | 0.24 | 3.22 | 0.44 |  |
| **Latvia** | 13.05 | 0.33 | 10.29 | 0.51 | 1.56 | 6.46 | | 0.15 | 5.53 | 0.23 | 1.53 |
| Lower bound 95%CI | 2.44 | 0.06 | 1.78 | 0.08 |  | 1.16 | | 0.02 | 0.94 | 0.03 |  |
| Upper bound 95%CI | 24.70 | 0.68 | 19.20 | 1.08 |  | 12.57 | | 0.34 | 10.42 | 0.50 |  |
| **Lithuania** | 4.85 | 0.12 | 6.00 | 0.30 | 2.52 | 1.82 | | 0.04 | 2.77 | 0.12 | 2.74 |
| Lower bound 95%CI | 0.88 | 0.02 | 1.08 | 0.05 |  | 0.26 | | 0.01 | 0.53 | 0.02 |  |
| Upper bound 95%CI | 10.59 | 0.28 | 11.92 | 0.66 |  | 4.31 | | 0.12 | 6.01 | 0.29 |  |
| **Luxembourg** | 1.97 | 0.12 | 1.80 | 0.29 | 2.35 | 1.31 | | 0.07 | 1.19 | 0.17 | 2.43 |
| Lower bound 95%CI | 0.36 | 0.02 | 0.34 | 0.05 |  | 0.25 | | 0.01 | 0.22 | 0.03 |  |
| Upper bound 95%CI | 4.07 | 0.29 | 3.38 | 0.65 |  | 2.81 | | 0.18 | 2.32 | 0.40 |  |
| **Malta** | 3.56 | 0.19 | 3.90 | 0.67 | 3.45 | 1.46 | | 0.07 | 2.12 | 0.33 | 4.44 |
| Lower bound 95%CI | 0.63 | 0.03 | 0.66 | 0.10 |  | 0.25 | | 0.01 | 0.34 | 0.05 |  |
| Upper bound 95%CI | 7.21 | 0.44 | 7.47 | 1.48 |  | 3.18 | | 0.19 | 4.28 | 0.78 |  |
| **Netherlands** | 2.34 | 0.10 | 1.78 | 0.22 | 2.21 | 1.67 | | 0.07 | 1.28 | 0.17 | 2.25 |
| Lower bound 95%CI | 0.42 | 0.02 | 0.32 | 0.04 |  | 0.29 | | 0.01 | 0.21 | 0.02 |  |
| Upper bound 95%CI | 4.67 | 0.22 | 3.40 | 0.47 |  | 3.41 | | 0.17 | 2.47 | 0.37 |  |
| **Poland** | 13.74 | 0.35 | 8.55 | 0.40 | 1.15 | 9.20 | | 0.23 | 6.21 | 0.29 | 1.25 |
| Lower bound 95%CI | 2.56 | 0.06 | 1.65 | 0.07 |  | 1.70 | | 0.04 | 1.16 | 0.05 |  |
| Upper bound 95%CI | 24.48 | 0.71 | 15.23 | 0.82 |  | 16.88 | | 0.50 | 11.17 | 0.62 |  |
| **Portugal** | 0.52 | 0.04 | 0.79 | 0.16 | 4.58 | 0.45 | | 0.03 | 0.58 | 0.11 | 3.52 |
| Lower bound 95%CI | 0.09 | 0.01 | 0.14 | 0.02 |  | 0.07 | | 0.00 | 0.11 | 0.02 |  |
| Upper bound 95%CI | 1.14 | 0.09 | 1.61 | 0.39 |  | 0.94 | | 0.08 | 1.25 | 0.29 |  |
| **Romania** | 21.64 | 0.56 | 15.62 | 0.71 | 1.27 | 15.86 | | 0.38 | 11.58 | 0.44 | 1.15 |
| Lower bound 95%CI | 4.05 | 0.09 | 3.12 | 0.13 |  | 2.99 | | 0.06 | 2.28 | 0.08 |  |
| Upper bound 95%CI | 37.61 | 1.09 | 27.84 | 1.40 |  | 28.56 | | 0.78 | 21.19 | 0.90 |  |
| **Slovakia** | 25.28 | 0.68 | 18.34 | 0.93 | 1.36 | 17.72 | | 0.46 | 14.22 | 0.61 | 1.34 |
| Lower bound 95%CI | 4.63 | 0.11 | 3.56 | 0.16 |  | 3.29 | | 0.08 | 2.65 | 0.10 |  |
| Upper bound 95%CI | 45.24 | 1.36 | 32.72 | 1.84 |  | 31.90 | | 0.94 | 25.59 | 1.23 |  |
| **Slovenia** | 7.55 | 0.22 | 4.27 | 0.22 | 0.98 | 5.38 | | 0.13 | 2.64 | 0.10 | 0.79 |
| Lower bound 95%CI | 1.48 | 0.04 | 0.75 | 0.03 |  | 0.98 | | 0.02 | 0.45 | 0.02 |  |
| Upper bound 95%CI | 13.31 | 0.44 | 7.63 | 0.44 |  | 9.48 | | 0.27 | 4.74 | 0.21 |  |
| **Spain** | 0.71 | 0.03 | 0.81 | 0.11 | 3.32 | 0.52 | | 0.03 | 0.55 | 0.08 | 3.04 |
| Lower bound 95%CI | 0.12 | 0.01 | 0.15 | 0.02 |  | 0.09 | | 0.00 | 0.10 | 0.01 |  |
| Upper bound 95%CI | 1.62 | 0.08 | 1.69 | 0.26 |  | 1.24 | | 0.07 | 1.10 | 0.19 |  |
| **Sweden** | 2.19 | 0.11 | 2.30 | 0.26 | 2.33 | 1.20 | | 0.06 | 1.32 | 0.15 | 2.55 |
| Lower bound 95%CI | 0.34 | 0.02 | 0.38 | 0.04 |  | 0.21 | | 0.01 | 0.24 | 0.02 |  |
| Upper bound 95%CI | 4.65 | 0.28 | 4.46 | 0.59 |  | 2.52 | | 0.14 | 2.70 | 0.36 |  |
| **European Union** | 25.04 | 0.89 | 17.62 | 1.53 | 1.71 | 17.53 | | 0.66 | 13.11 | 1.13 | 1.72 |
| Lower bound 95%CI | 4.54 | 0.15 | 3.38 | 0.26 |  | 3.25 | | 0.11 | 2.48 | 0.19 |  |
| Upper bound 95%CI | 43.46 | 1.71 | 30.86 | 2.99 |  | 31.08 | | 1.32 | 23.52 | 2.30 |  |
| Data and definitions from Global Burden of Disease Database. 2021 | | | | | | | | | | | |
| *Each value corresponds to mortality rates per 100.000 inhabitants (95% Uncertainty Interval) | | | | | | | | | | | |
| Abbreviation: CFI, case-fatality index | | | | | | | | | | | |
|  |  |  |  |  |  |  |  |  |  |  |  |

| Appendix Table 22. Age-standardised mortality rates and case fatality indexes for IHD attributable to Diet low in vegetables. 2011 , 2021 | | | | | | | | | | |  |
| --- | --- | --- | --- | --- | --- | --- | --- | --- | --- | --- | --- |
|  |  |  |  |  |  |  |  |  |  |  |  |
| **Country** | **2011** | | | | | **2021** | | | | |  |
|  | **Men** | | **Women** | | **CFI Ratio** | **Men** | | **Women** | | **CFI Ratio** |  |
|  | **Mortality rate** | **CFI** | **Mortality rate** | **CFI** |  | **Mortality rate** | **CFI** | **Mortality rate** | **CFI** |  |  |
| **Austria** | 3.50 | 0.13 | 1.79 | 0.22 | 1.62 | 2.55 | 0.10 | 1.23 | 0.15 | 1.53 |  |
| Lower bound 95%CI | 1.57 | 0.06 | 0.74 | 0.08 |  | 1.18 | 0.04 | 0.51 | 0.06 |  |  |
| Upper bound 95%CI | 5.60 | 0.24 | 3.05 | 0.41 |  | 4.09 | 0.18 | 2.11 | 0.29 |  |  |
| **Belgium** | 1.82 | 0.08 | 0.81 | 0.11 | 1.29 | 1.17 | 0.05 | 0.49 | 0.06 | 1.21 |  |
| Lower bound 95%CI | 0.80 | 0.03 | 0.32 | 0.04 |  | 0.49 | 0.02 | 0.19 | 0.02 |  |  |
| Upper bound 95%CI | 3.06 | 0.15 | 1.38 | 0.21 |  | 2.07 | 0.11 | 0.86 | 0.13 |  |  |
| **Bulgaria** | 3.57 | 0.08 | 2.31 | 0.09 | 1.15 | 2.98 | 0.07 | 1.76 | 0.07 | 1.02 |  |
| Lower bound 95%CI | 1.39 | 0.03 | 0.88 | 0.03 |  | 1.11 | 0.02 | 0.62 | 0.02 |  |  |
| Upper bound 95%CI | 6.62 | 0.16 | 4.14 | 0.18 |  | 5.54 | 0.14 | 3.44 | 0.15 |  |  |
| **Croatia** | 5.93 | 0.16 | 3.49 | 0.20 | 1.26 | 4.51 | 0.12 | 2.72 | 0.14 | 1.19 |  |
| Lower bound 95%CI | 2.57 | 0.06 | 1.54 | 0.08 |  | 2.01 | 0.05 | 1.19 | 0.05 |  |  |
| Upper bound 95%CI | 9.49 | 0.29 | 5.73 | 0.38 |  | 7.28 | 0.22 | 4.45 | 0.26 |  |  |
| **Cyprus** | 5.23 | 0.34 | 2.39 | 0.48 | 1.44 | 3.71 | 0.20 | 1.76 | 0.28 | 1.36 |  |
| Lower bound 95%CI | 2.30 | 0.13 | 1.01 | 0.17 |  | 1.63 | 0.08 | 0.70 | 0.09 |  |  |
| Upper bound 95%CI | 8.23 | 0.61 | 3.93 | 0.94 |  | 6.10 | 0.39 | 3.03 | 0.56 |  |  |
| **Czechia** | 7.39 | 0.18 | 4.05 | 0.18 | 0.99 | 5.18 | 0.12 | 2.74 | 0.10 | 0.85 |  |
| Lower bound 95%CI | 3.38 | 0.07 | 1.71 | 0.07 |  | 2.31 | 0.05 | 1.16 | 0.04 |  |  |
| Upper bound 95%CI | 11.98 | 0.32 | 6.60 | 0.32 |  | 8.41 | 0.23 | 4.53 | 0.19 |  |  |
| **Denmark** | 2.24 | 0.14 | 1.08 | 0.19 | 1.29 | 1.50 | 0.09 | 0.66 | 0.12 | 1.28 |  |
| Lower bound 95%CI | 0.98 | 0.06 | 0.43 | 0.07 |  | 0.68 | 0.04 | 0.27 | 0.04 |  |  |
| Upper bound 95%CI | 3.62 | 0.26 | 1.83 | 0.35 |  | 2.42 | 0.17 | 1.11 | 0.22 |  |  |
| **Estonia** | 7.60 | 0.16 | 3.46 | 0.11 | 0.71 | 3.76 | 0.08 | 1.61 | 0.05 | 0.67 |  |
| Lower bound 95%CI | 3.45 | 0.07 | 1.50 | 0.05 |  | 1.65 | 0.03 | 0.66 | 0.02 |  |  |
| Upper bound 95%CI | 12.21 | 0.28 | 5.79 | 0.21 |  | 6.10 | 0.14 | 2.62 | 0.09 |  |  |
| **Finland** | 5.09 | 0.24 | 2.24 | 0.27 | 1.11 | 3.47 | 0.14 | 1.50 | 0.16 | 1.15 |  |
| Lower bound 95%CI | 2.25 | 0.10 | 0.92 | 0.10 |  | 1.54 | 0.06 | 0.64 | 0.06 |  |  |
| Upper bound 95%CI | 8.19 | 0.44 | 3.69 | 0.50 |  | 5.57 | 0.26 | 2.48 | 0.31 |  |  |
| **France** | 2.07 | 0.08 | 0.79 | 0.10 | 1.21 | 1.46 | 0.06 | 0.54 | 0.07 | 1.14 |  |
| Lower bound 95%CI | 0.94 | 0.03 | 0.32 | 0.03 |  | 0.66 | 0.02 | 0.23 | 0.02 |  |  |
| Upper bound 95%CI | 3.34 | 0.14 | 1.29 | 0.17 |  | 2.35 | 0.10 | 0.89 | 0.12 |  |  |
| **Germany** | 3.60 | 0.12 | 1.73 | 0.18 | 1.52 | 2.75 | 0.11 | 1.18 | 0.13 | 1.26 |  |
| Lower bound 95%CI | 1.63 | 0.05 | 0.74 | 0.07 |  | 1.22 | 0.04 | 0.49 | 0.05 |  |  |
| Upper bound 95%CI | 5.78 | 0.21 | 2.87 | 0.32 |  | 4.41 | 0.19 | 1.98 | 0.25 |  |  |
| **Greece** | 0.57 | 0.03 | 0.46 | 0.08 | 2.77 | 0.68 | 0.04 | 0.37 | 0.06 | 1.71 |  |
| Lower bound 95%CI | 0.13 | 0.01 | 0.08 | 0.01 |  | 0.19 | 0.01 | 0.10 | 0.01 |  |  |
| Upper bound 95%CI | 1.26 | 0.07 | 1.14 | 0.22 |  | 1.50 | 0.09 | 0.85 | 0.17 |  |  |
| **Hungary** | 5.67 | 0.12 | 3.21 | 0.12 | 1.00 | 4.15 | 0.09 | 2.33 | 0.09 | 0.96 |  |
| Lower bound 95%CI | 2.43 | 0.05 | 1.33 | 0.04 |  | 1.70 | 0.03 | 0.96 | 0.03 |  |  |
| Upper bound 95%CI | 9.42 | 0.21 | 5.53 | 0.22 |  | 7.11 | 0.17 | 4.08 | 0.17 |  |  |
| **Ireland** | 3.49 | 0.16 | 1.64 | 0.22 | 1.40 | 1.90 | 0.09 | 0.88 | 0.12 | 1.35 |  |
| Lower bound 95%CI | 1.56 | 0.06 | 0.70 | 0.08 |  | 0.79 | 0.03 | 0.37 | 0.04 |  |  |
| Upper bound 95%CI | 5.67 | 0.29 | 2.73 | 0.42 |  | 3.09 | 0.16 | 1.52 | 0.23 |  |  |
| **Italy** | 1.84 | 0.08 | 0.83 | 0.09 | 1.22 | 1.41 | 0.06 | 0.61 | 0.07 | 1.14 |  |
| Lower bound 95%CI | 0.80 | 0.03 | 0.34 | 0.03 |  | 0.60 | 0.02 | 0.24 | 0.02 |  |  |
| Upper bound 95%CI | 3.02 | 0.14 | 1.45 | 0.18 |  | 2.34 | 0.12 | 1.08 | 0.15 |  |  |
| **Latvia** | 9.86 | 0.25 | 4.60 | 0.23 | 0.92 | 6.21 | 0.15 | 2.77 | 0.12 | 0.80 |  |
| Lower bound 95%CI | 4.46 | 0.10 | 1.97 | 0.09 |  | 2.69 | 0.06 | 1.11 | 0.04 |  |  |
| Upper bound 95%CI | 15.94 | 0.44 | 7.78 | 0.44 |  | 10.03 | 0.27 | 4.54 | 0.22 |  |  |
| **Lithuania** | 10.94 | 0.27 | 5.28 | 0.26 | 0.98 | 7.04 | 0.17 | 3.45 | 0.15 | 0.88 |  |
| Lower bound 95%CI | 4.98 | 0.11 | 2.28 | 0.10 |  | 3.09 | 0.07 | 1.48 | 0.06 |  |  |
| Upper bound 95%CI | 17.79 | 0.47 | 8.85 | 0.49 |  | 11.20 | 0.30 | 5.77 | 0.28 |  |  |
| **Luxembourg** | 2.67 | 0.17 | 1.20 | 0.20 | 1.16 | 1.82 | 0.10 | 0.80 | 0.12 | 1.18 |  |
| Lower bound 95%CI | 1.14 | 0.06 | 0.51 | 0.07 |  | 0.79 | 0.04 | 0.33 | 0.04 |  |  |
| Upper bound 95%CI | 4.22 | 0.30 | 1.96 | 0.38 |  | 2.95 | 0.18 | 1.33 | 0.23 |  |  |
| **Malta** | 2.51 | 0.14 | 1.46 | 0.25 | 1.83 | 1.33 | 0.07 | 0.89 | 0.14 | 2.04 |  |
| Lower bound 95%CI | 1.03 | 0.05 | 0.58 | 0.08 |  | 0.52 | 0.02 | 0.33 | 0.04 |  |  |
| Upper bound 95%CI | 4.42 | 0.27 | 2.54 | 0.50 |  | 2.36 | 0.14 | 1.60 | 0.29 |  |  |
| **Netherlands** | 2.35 | 0.10 | 1.03 | 0.13 | 1.28 | 1.69 | 0.07 | 0.73 | 0.09 | 1.27 |  |
| Lower bound 95%CI | 1.07 | 0.04 | 0.44 | 0.05 |  | 0.74 | 0.03 | 0.31 | 0.04 |  |  |
| Upper bound 95%CI | 3.74 | 0.17 | 1.68 | 0.23 |  | 2.72 | 0.14 | 1.21 | 0.18 |  |  |
| **Poland** | 3.26 | 0.08 | 1.57 | 0.07 | 0.89 | 2.09 | 0.05 | 1.03 | 0.05 | 0.91 |  |
| Lower bound 95%CI | 1.41 | 0.03 | 0.63 | 0.03 |  | 0.83 | 0.02 | 0.39 | 0.02 |  |  |
| Upper bound 95%CI | 5.59 | 0.16 | 2.81 | 0.15 |  | 3.73 | 0.11 | 1.94 | 0.11 |  |  |
| **Portugal** | 0.95 | 0.06 | 0.51 | 0.10 | 1.62 | 0.78 | 0.05 | 0.37 | 0.07 | 1.29 |  |
| Lower bound 95%CI | 0.37 | 0.02 | 0.19 | 0.03 |  | 0.30 | 0.02 | 0.14 | 0.02 |  |  |
| Upper bound 95%CI | 1.70 | 0.13 | 0.97 | 0.23 |  | 1.43 | 0.11 | 0.69 | 0.16 |  |  |
| **Romania** | 0.56 | 0.01 | 0.38 | 0.02 | 1.19 | 0.41 | 0.01 | 0.25 | 0.01 | 0.96 |  |
| Lower bound 95%CI | 0.07 | 0.00 | 0.05 | 0.00 |  | 0.04 | 0.00 | 0.02 | 0.00 |  |  |
| Upper bound 95%CI | 1.45 | 0.04 | 0.99 | 0.05 |  | 1.10 | 0.03 | 0.74 | 0.03 |  |  |
| **Slovakia** | 8.46 | 0.23 | 4.94 | 0.25 | 1.09 | 6.05 | 0.16 | 3.80 | 0.16 | 1.05 |  |
| Lower bound 95%CI | 3.86 | 0.09 | 1.95 | 0.09 |  | 2.53 | 0.06 | 1.59 | 0.06 |  |  |
| Upper bound 95%CI | 13.52 | 0.41 | 8.17 | 0.46 |  | 9.64 | 0.28 | 6.16 | 0.30 |  |  |
| **Slovenia** | 2.86 | 0.08 | 1.25 | 0.06 | 0.76 | 2.10 | 0.05 | 0.79 | 0.03 | 0.60 |  |
| Lower bound 95%CI | 1.29 | 0.03 | 0.51 | 0.02 |  | 0.93 | 0.02 | 0.31 | 0.01 |  |  |
| Upper bound 95%CI | 4.54 | 0.15 | 2.12 | 0.12 |  | 3.38 | 0.10 | 1.30 | 0.06 |  |  |
| **Spain** | 1.64 | 0.08 | 0.67 | 0.09 | 1.19 | 1.21 | 0.06 | 0.45 | 0.07 | 1.07 |  |
| Lower bound 95%CI | 0.69 | 0.03 | 0.27 | 0.03 |  | 0.52 | 0.02 | 0.19 | 0.02 |  |  |
| Upper bound 95%CI | 2.72 | 0.14 | 1.12 | 0.17 |  | 1.97 | 0.11 | 0.77 | 0.13 |  |  |
| **Sweden** | 3.58 | 0.18 | 1.80 | 0.21 | 1.12 | 2.11 | 0.10 | 1.09 | 0.12 | 1.20 |  |
| Lower bound 95%CI | 1.62 | 0.07 | 0.75 | 0.07 |  | 0.98 | 0.04 | 0.47 | 0.05 |  |  |
| Upper bound 95%CI | 5.73 | 0.34 | 2.99 | 0.40 |  | 3.46 | 0.20 | 1.79 | 0.24 |  |  |
| **European Union** | 7.51 | 0.27 | 4.04 | 0.35 | 1.31 | 5.38 | 0.20 | 3.04 | 0.26 | 1.30 |  |
| Lower bound 95%CI | 3.35 | 0.11 | 1.67 | 0.13 |  | 2.38 | 0.08 | 1.28 | 0.10 |  |  |
| Upper bound 95%CI | 12.18 | 0.48 | 6.63 | 0.64 |  | 8.95 | 0.38 | 5.06 | 0.49 |  |  |

Data and definitions from Global Burden of Disease Database. 2021

*Each value corresponds to mortality rates per 100.000 inhabitants (95% Uncertainty Interval)

Abbreviation: CFI, case-fatality index

| Appendix Table 23. Age-standardised mortality rates and case fatality indexes for IHD attributable to Diet low in whole grains. 2011 , 2021 | | | | | | | | | | |
| --- | --- | --- | --- | --- | --- | --- | --- | --- | --- | --- |
| **Country** | **2011** | | | | | **2021** | | | | |
|  | **Men** | | **Women** | | **CFI Ratio** | **Men** | | **Women** | | **CFI Ratio** |
|  | **Mortality rate** | **CFI** | **Mortality rate** | **CFI** |  | **Mortality rate** | **CFI** | **Mortality rate** | **CFI** |  |
| **Austria** | 12.35 | 0.47 | 5.90 | 0.72 | 1.51 | 9.17 | 0.35 | 4.11 | 0.50 | 1.42 |
| Lower bound 95%CI | 7.32 | 0.26 | 3.31 | 0.36 |  | 5.37 | 0.18 | 2.37 | 0.26 |  |
| Upper bound 95%CI | 17.20 | 0.74 | 8.58 | 1.16 |  | 12.97 | 0.56 | 6.03 | 0.84 |  |
| **Belgium** | 10.76 | 0.49 | 4.52 | 0.60 | 1.22 | 7.36 | 0.34 | 2.95 | 0.39 | 1.16 |
| Lower bound 95%CI | 6.44 | 0.27 | 2.55 | 0.30 |  | 4.33 | 0.17 | 1.72 | 0.20 |  |
| Upper bound 95%CI | 14.96 | 0.76 | 6.43 | 0.96 |  | 10.35 | 0.54 | 4.25 | 0.64 |  |
| **Bulgaria** | 47.35 | 1.03 | 25.04 | 0.97 | 0.94 | 41.71 | 0.95 | 21.03 | 0.83 | 0.87 |
| Lower bound 95%CI | 28.87 | 0.57 | 15.02 | 0.54 |  | 25.07 | 0.52 | 12.26 | 0.43 |  |
| Upper bound 95%CI | 65.32 | 1.58 | 35.25 | 1.51 |  | 58.10 | 1.50 | 29.45 | 1.31 |  |
| **Croatia** | 25.89 | 0.70 | 14.76 | 0.86 | 1.22 | 20.04 | 0.52 | 11.78 | 0.61 | 1.16 |
| Lower bound 95%CI | 15.54 | 0.37 | 8.90 | 0.45 |  | 11.55 | 0.27 | 6.89 | 0.31 |  |
| Upper bound 95%CI | 35.50 | 1.09 | 21.15 | 1.40 |  | 27.72 | 0.83 | 16.92 | 1.00 |  |
| **Cyprus** | 27.66 | 1.78 | 12.28 | 2.48 | 1.40 | 19.15 | 1.05 | 8.84 | 1.40 | 1.33 |
| Lower bound 95%CI | 16.43 | 0.92 | 7.25 | 1.23 |  | 11.16 | 0.53 | 4.86 | 0.65 |  |
| Upper bound 95%CI | 38.34 | 2.83 | 17.40 | 4.15 |  | 26.70 | 1.71 | 12.81 | 2.38 |  |
| **Czechia** | 19.21 | 0.46 | 9.70 | 0.42 | 0.91 | 14.29 | 0.34 | 6.95 | 0.26 | 0.78 |
| Lower bound 95%CI | 11.44 | 0.25 | 5.54 | 0.22 |  | 7.99 | 0.17 | 3.89 | 0.13 |  |
| Upper bound 95%CI | 26.93 | 0.72 | 14.47 | 0.69 |  | 20.19 | 0.54 | 10.23 | 0.44 |  |
| **Denmark** | 11.49 | 0.74 | 5.24 | 0.90 | 1.22 | 8.04 | 0.49 | 3.29 | 0.58 | 1.19 |
| Lower bound 95%CI | 6.78 | 0.38 | 3.13 | 0.48 |  | 4.82 | 0.26 | 1.93 | 0.29 |  |
| Upper bound 95%CI | 16.07 | 1.16 | 7.38 | 1.43 |  | 11.07 | 0.77 | 4.69 | 0.95 |  |
| **Estonia** | 33.31 | 0.71 | 14.66 | 0.49 | 0.69 | 17.35 | 0.35 | 7.07 | 0.22 | 0.64 |
| Lower bound 95%CI | 20.21 | 0.39 | 8.31 | 0.25 |  | 10.28 | 0.18 | 4.03 | 0.11 |  |
| Upper bound 95%CI | 45.56 | 1.05 | 21.03 | 0.76 |  | 24.21 | 0.54 | 10.10 | 0.35 |  |
| **Finland** | 19.22 | 0.92 | 8.00 | 0.97 | 1.05 | 13.49 | 0.56 | 5.51 | 0.61 | 1.09 |
| Lower bound 95%CI | 11.38 | 0.49 | 4.58 | 0.49 |  | 7.93 | 0.29 | 3.13 | 0.30 |  |
| Upper bound 95%CI | 27.00 | 1.44 | 11.63 | 1.58 |  | 19.29 | 0.91 | 7.96 | 1.01 |  |
| **France** | 10.81 | 0.42 | 4.06 | 0.49 | 1.19 | 7.75 | 0.31 | 2.85 | 0.35 | 1.14 |
| Lower bound 95%CI | 6.75 | 0.24 | 2.38 | 0.26 |  | 4.72 | 0.17 | 1.65 | 0.18 |  |
| Upper bound 95%CI | 14.78 | 0.62 | 5.77 | 0.77 |  | 10.56 | 0.47 | 4.06 | 0.56 |  |
| **Germany** | 12.16 | 0.40 | 5.46 | 0.57 | 1.42 | 9.71 | 0.38 | 3.89 | 0.44 | 1.17 |
| Lower bound 95%CI | 7.13 | 0.21 | 3.05 | 0.29 |  | 5.65 | 0.19 | 2.15 | 0.21 |  |
| Upper bound 95%CI | 16.69 | 0.60 | 7.93 | 0.90 |  | 13.39 | 0.58 | 5.68 | 0.73 |  |
| **Greece** | 26.53 | 1.32 | 15.57 | 2.66 | 2.01 | 19.82 | 1.09 | 9.35 | 1.62 | 1.48 |
| Lower bound 95%CI | 16.73 | 0.75 | 9.11 | 1.36 |  | 12.43 | 0.60 | 5.52 | 0.81 |  |
| Upper bound 95%CI | 35.32 | 1.96 | 21.63 | 4.23 |  | 26.54 | 1.65 | 13.00 | 2.63 |  |
| **Hungary** | 37.31 | 0.76 | 19.63 | 0.71 | 0.93 | 28.61 | 0.63 | 15.03 | 0.56 | 0.90 |
| Lower bound 95%CI | 22.79 | 0.42 | 11.57 | 0.38 |  | 16.96 | 0.33 | 8.76 | 0.29 |  |
| Upper bound 95%CI | 51.35 | 1.15 | 27.80 | 1.10 |  | 39.92 | 0.97 | 20.92 | 0.86 |  |
| **Ireland** | 9.47 | 0.43 | 4.13 | 0.56 | 1.30 | 6.06 | 0.28 | 2.56 | 0.34 | 1.23 |
| Lower bound 95%CI | 5.55 | 0.23 | 2.37 | 0.29 |  | 3.54 | 0.14 | 1.43 | 0.17 |  |
| Upper bound 95%CI | 13.30 | 0.67 | 6.10 | 0.93 |  | 8.62 | 0.45 | 3.83 | 0.58 |  |
| **Italy** | 16.36 | 0.67 | 7.49 | 0.83 | 1.24 | 12.04 | 0.52 | 5.32 | 0.61 | 1.17 |
| Lower bound 95%CI | 9.92 | 0.35 | 4.25 | 0.41 |  | 7.30 | 0.26 | 2.97 | 0.29 |  |
| Upper bound 95%CI | 22.44 | 1.06 | 10.60 | 1.35 |  | 16.54 | 0.86 | 7.53 | 1.02 |  |
| **Latvia** | 54.59 | 1.37 | 25.36 | 1.25 | 0.92 | 36.73 | 0.87 | 16.34 | 0.69 | 0.79 |
| Lower bound 95%CI | 33.26 | 0.76 | 14.96 | 0.66 |  | 21.57 | 0.46 | 9.43 | 0.35 |  |
| Upper bound 95%CI | 74.33 | 2.04 | 35.88 | 2.01 |  | 51.60 | 1.38 | 23.08 | 1.11 |  |
| **Lithuania** | 54.24 | 1.32 | 25.90 | 1.29 | 0.97 | 37.87 | 0.91 | 18.28 | 0.79 | 0.87 |
| Lower bound 95%CI | 32.99 | 0.74 | 15.25 | 0.68 |  | 22.17 | 0.48 | 10.65 | 0.41 |  |
| Upper bound 95%CI | 73.66 | 1.93 | 36.27 | 2.00 |  | 53.66 | 1.45 | 26.07 | 1.27 |  |
| **Luxembourg** | 10.21 | 0.65 | 4.31 | 0.70 | 1.09 | 7.35 | 0.40 | 3.03 | 0.44 | 1.10 |
| Lower bound 95%CI | 6.02 | 0.34 | 2.44 | 0.34 |  | 4.25 | 0.20 | 1.70 | 0.22 |  |
| Upper bound 95%CI | 14.09 | 1.01 | 6.19 | 1.19 |  | 10.13 | 0.63 | 4.38 | 0.75 |  |
| **Malta** | 26.15 | 1.42 | 14.41 | 2.46 | 1.74 | 15.74 | 0.80 | 10.08 | 1.57 | 1.96 |
| Lower bound 95%CI | 15.90 | 0.76 | 8.55 | 1.25 |  | 9.41 | 0.42 | 5.92 | 0.80 |  |
| Upper bound 95%CI | 35.49 | 2.17 | 20.14 | 3.99 |  | 21.58 | 1.28 | 14.15 | 2.58 |  |
| **Netherlands** | 8.38 | 0.35 | 3.64 | 0.45 | 1.26 | 6.78 | 0.30 | 2.83 | 0.37 | 1.22 |
| Lower bound 95%CI | 4.87 | 0.18 | 2.09 | 0.23 |  | 3.93 | 0.15 | 1.60 | 0.18 |  |
| Upper bound 95%CI | 11.72 | 0.55 | 5.21 | 0.72 |  | 9.59 | 0.49 | 3.97 | 0.59 |  |
| **Poland** | 26.58 | 0.67 | 12.37 | 0.58 | 0.86 | 21.18 | 0.54 | 10.10 | 0.47 | 0.88 |
| Lower bound 95%CI | 16.17 | 0.36 | 7.16 | 0.29 |  | 12.80 | 0.28 | 5.76 | 0.23 |  |
| Upper bound 95%CI | 36.34 | 1.06 | 17.63 | 0.95 |  | 29.51 | 0.88 | 14.04 | 0.78 |  |
| **Portugal** | 8.52 | 0.58 | 4.13 | 0.85 | 1.46 | 7.60 | 0.52 | 3.15 | 0.59 | 1.13 |
| Lower bound 95%CI | 5.19 | 0.31 | 2.42 | 0.43 |  | 4.62 | 0.27 | 1.80 | 0.28 |  |
| Upper bound 95%CI | 11.70 | 0.92 | 5.85 | 1.40 |  | 10.41 | 0.83 | 4.47 | 1.03 |  |
| **Romania** | 28.41 | 0.73 | 15.86 | 0.72 | 0.98 | 23.72 | 0.57 | 12.73 | 0.49 | 0.85 |
| Lower bound 95%CI | 16.94 | 0.39 | 9.25 | 0.38 |  | 13.86 | 0.30 | 7.37 | 0.25 |  |
| Upper bound 95%CI | 39.20 | 1.13 | 22.46 | 1.13 |  | 33.06 | 0.91 | 18.11 | 0.77 |  |
| **Slovakia** | 39.69 | 1.07 | 21.97 | 1.11 | 1.04 | 30.68 | 0.79 | 18.26 | 0.79 | 0.99 |
| Lower bound 95%CI | 24.17 | 0.58 | 12.31 | 0.56 |  | 17.76 | 0.41 | 10.75 | 0.41 |  |
| Upper bound 95%CI | 54.70 | 1.64 | 31.94 | 1.79 |  | 42.50 | 1.25 | 25.86 | 1.25 |  |
| **Slovenia** | 10.11 | 0.29 | 4.19 | 0.21 | 0.72 | 7.50 | 0.18 | 2.67 | 0.11 | 0.57 |
| Lower bound 95%CI | 6.01 | 0.15 | 2.34 | 0.10 |  | 4.29 | 0.09 | 1.51 | 0.05 |  |
| Upper bound 95%CI | 14.15 | 0.46 | 6.14 | 0.35 |  | 10.69 | 0.30 | 3.94 | 0.18 |  |
| **Spain** | 8.86 | 0.42 | 3.50 | 0.48 | 1.15 | 6.81 | 0.35 | 2.46 | 0.36 | 1.04 |
| Lower bound 95%CI | 5.36 | 0.23 | 2.02 | 0.25 |  | 4.00 | 0.19 | 1.42 | 0.19 |  |
| Upper bound 95%CI | 12.07 | 0.63 | 5.01 | 0.78 |  | 9.34 | 0.54 | 3.54 | 0.60 |  |
| **Sweden** | 15.67 | 0.81 | 7.83 | 0.89 | 1.11 | 9.75 | 0.47 | 4.88 | 0.55 | 1.16 |
| Lower bound 95%CI | 9.44 | 0.42 | 4.47 | 0.44 |  | 5.74 | 0.23 | 2.79 | 0.27 |  |
| Upper bound 95%CI | 21.84 | 1.30 | 11.16 | 1.48 |  | 13.96 | 0.80 | 7.04 | 0.93 |  |
| **European Union** | 59.34 | 2.11 | 31.57 | 2.74 | 1.30 | 43.81 | 1.64 | 24.45 | 2.11 | 1.29 |
| Lower bound 95%CI | 36.62 | 1.19 | 18.77 | 1.46 |  | 26.24 | 0.87 | 14.31 | 1.08 |  |
| Upper bound 95%CI | 79.89 | 3.15 | 43.72 | 4.23 |  | 59.60 | 2.52 | 34.27 | 3.35 |  |

Data and definitions from Global Burden of Disease Database. 2021

*Each value corresponds to mortality rates per 100.000 inhabitants (95% Uncertainty Interval)

| **Appendix Table 24. Z scores for Case Fatality Index attributable to High LDL cholesterol. 2021.** | | | | | | | | | |
| --- | --- | --- | --- | --- | --- | --- | --- | --- | --- |
|  |  |  |  |  |  |  |  |  |  |
| **Country** | **SE men** | **CFI Men** | **SE women** | **CFI Women** | **SE MPR men** | **SE MPR women** | **Z score** | **P value** | **Sig** |
| **Austria** | 5.44 | 1.22 | 3.12 | 1.89 | 0.21 | 0.38 | 1.53 | 0.13 | NS |
|  |  |  |  |  |  |  |  |  |  |
|  |  |  |  |  |  |  |  |  |  |
| **Belgium** | 3.02 | 0.78 | 1.51 | 1.03 | 0.14 | 0.20 | 0.98 | 0.33 | NS |
|  |  |  |  |  |  |  |  |  |  |
|  |  |  |  |  |  |  |  |  |  |
| **Bulgaria** | 16.07 | 2.14 | 8.87 | 1.84 | 0.37 | 0.36 | -0.59 | 0.56 | NS |
|  |  |  |  |  |  |  |  |  |  |
|  |  |  |  |  |  |  |  |  |  |
| **Croatia** | 8.94 | 1.25 | 6.72 | 1.67 | 0.24 | 0.35 | 0.99 | 0.32 | NS |
|  |  |  |  |  |  |  |  |  |  |
|  |  |  |  |  |  |  |  |  |  |
| **Cyprus** | 7.36 | 2.15 | 4.24 | 2.91 | 0.41 | 0.68 | 0.95 | 0.34 | NS |
|  |  |  |  |  |  |  |  |  |  |
|  |  |  |  |  |  |  |  |  |  |
| **Czechia** | 10.28 | 1.32 | 5.9 | 1.12 | 0.25 | 0.23 | -0.59 | 0.55 | NS |
|  |  |  |  |  |  |  |  |  |  |
|  |  |  |  |  |  |  |  |  |  |
| **Denmark** | 3.36 | 1.18 | 1.66 | 1.51 | 0.21 | 0.30 | 0.93 | 0.35 | NS |
|  |  |  |  |  |  |  |  |  |  |
|  |  |  |  |  |  |  |  |  |  |
| **Estonia** | 8.73 | 1.00 | 4.37 | 0.64 | 0.18 | 0.14 | -1.58 | 0.11 | NS |
|  |  |  |  |  |  |  |  |  |  |
|  |  |  |  |  |  |  |  |  |  |
| **Finland** | 6.44 | 1.5 | 3.14 | 1.65 | 0.27 | 0.35 | 0.34 | 0.74 | NS |
|  |  |  |  |  |  |  |  |  |  |
|  |  |  |  |  |  |  |  |  |  |
| **France** | 2.70 | 0.63 | 1.19 | 0.73 | 0.11 | 0.15 | 0.52 | 0.6 | NS |
|  |  |  |  |  |  |  |  |  |  |
|  |  |  |  |  |  |  |  |  |  |
| **Germany** | 5.37 | 1.21 | 2.73 | 1.56 | 0.21 | 0.31 | 0.92 | 0.36 | NS |
|  |  |  |  |  |  |  |  |  |  |
|  |  |  |  |  |  |  |  |  |  |
| **Greece** | 5.46 | 1.86 | 3.03 | 2.78 | 0.30 | 0.53 | 1.50 | 0.13 | NS |
|  |  |  |  |  |  |  |  |  |  |
|  |  |  |  |  |  |  |  |  |  |
| **Hungary** | 12.55 | 1.61 | 7.4 | 1.47 | 0.28 | 0.28 | -0.35 | 0.73 | NS |
|  |  |  |  |  |  |  |  |  |  |
|  |  |  |  |  |  |  |  |  |  |
| **Ireland** | 3.99 | 0.99 | 2.15 | 1.35 | 0.19 | 0.29 | 1.02 | 0.31 | NS |
|  |  |  |  |  |  |  |  |  |  |
|  |  |  |  |  |  |  |  |  |  |
| **Italy** | 3.52 | 0.85 | 1.9 | 1.03 | 0.16 | 0.22 | 0.65 | 0.52 | NS |
|  |  |  |  |  |  |  |  |  |  |
|  |  |  |  |  |  |  |  |  |  |
| **Latvia** | 14.02 | 1.91 | 7.28 | 1.57 | 0.34 | 0.31 | -0.74 | 0.46 | NS |
|  |  |  |  |  |  |  |  |  |  |
|  |  |  |  |  |  |  |  |  |  |
| **Lithuania** | 17.23 | 2.36 | 9.78 | 2.08 | 0.42 | 0.43 | -0.47 | 0.64 | NS |
|  |  |  |  |  |  |  |  |  |  |
|  |  |  |  |  |  |  |  |  |  |
| **Luxembourg** | 3.86 | 1.15 | 1.94 | 1.36 | 0.21 | 0.29 | 0.59 | 0.55 | NS |
|  |  |  |  |  |  |  |  |  |  |
|  |  |  |  |  |  |  |  |  |  |
| **Malta** | 4.93 | 1.49 | 3.76 | 3.01 | 0.26 | 0.59 | 2.35* | 0.02 | W>M |
|  |  |  |  |  |  |  |  |  |  |
|  |  |  |  |  |  |  |  |  |  |
| **Netherlands** | 3.22 | 0.77 | 1.63 | 1.06 | 0.15 | 0.22 | 1.14 | 0.26 | NS |
|  |  |  |  |  |  |  |  |  |  |
|  |  |  |  |  |  |  |  |  |  |
| **Poland** | 8.75 | 1.21 | 4.88 | 1.11 | 0.23 | 0.24 | -0.31 | 0.76 | NS |
|  |  |  |  |  |  |  |  |  |  |
|  |  |  |  |  |  |  |  |  |  |
| **Portugal** | 2.89 | 1.29 | 1.55 | 1.52 | 0.20 | 0.30 | 0.62 | 0.53 | NS |
|  |  |  |  |  |  |  |  |  |  |
|  |  |  |  |  |  |  |  |  |  |
| **Romania** | 11.89 | 1.65 | 7.73 | 1.49 | 0.29 | 0.30 | -0.39 | 0.7 | NS |
|  |  |  |  |  |  |  |  |  |  |
|  |  |  |  |  |  |  |  |  |  |
| **Slovakia** | 13.62 | 1.89 | 9.27 | 1.96 | 0.36 | 0.40 | 0.13 | 0.9 | NS |
|  |  |  |  |  |  |  |  |  |  |
|  |  |  |  |  |  |  |  |  |  |
| **Slovenia** | 4.16 | 0.55 | 1.87 | 0.34 | 0.11 | 0.08 | -1.63 | 0.1 | NS |
|  |  |  |  |  |  |  |  |  |  |
|  |  |  |  |  |  |  |  |  |  |
| **Spain** | 2.86 | 0.88 | 1.32 | 0.98 | 0.15 | 0.20 | 0.41 | 0.68 | NS |
|  |  |  |  |  |  |  |  |  |  |
|  |  |  |  |  |  |  |  |  |  |
| **Sweden** | 4.24 | 1.09 | 2.38 | 1.30 | 0.21 | 0.27 | 0.62 | 0.54 | NS |
|  |  |  |  |  |  |  |  |  |  |
|  |  |  |  |  |  |  |  |  |  |
| **European Union** | 5.07 | 1.12 | 2.79 | 1.24 | 0.19 | 0.24 | 0.39 | 0.70 | NS |
|  |  |  |  |  |  |  |  |  |  |
|  |  |  |  |  |  |  |  |  |  |

* Denotes z-values exceeding the 95% threshold (z = 1.96).

^†^ Denotes z-values exceeding the 99% threshold (z = 2.58).

Abbreviations: CFI, case-fatality index; MPR, mortality-prevalence ratio

| **Appendix Table 25. Z scores for Case Fatality Index attributable to High systolic blood pressure. 2021.** | | | | | | | | | | | |
| --- | --- | --- | --- | --- | --- | --- | --- | --- | --- | --- | --- |
|  |  |  |  |  |  |  |  |  |  |  |  |
| **Country** | **SE men** | **CFI Men** | **SE women** | **CFI Women** | **SE MPR men** | **SE MPR women** | **Z score** | **P value** | | **Sig** | |
| **Austria** | 5.76 | 1.92 | 3.9 | 3.29 | 0.22 | 0.48 | 2.60*^†^ | | 0.01 | | W>M |
|  |  |  |  |  |  |  |  |  |  |  |  |
|  |  |  |  |  |  |  |  |  |  |  |  |
| **Belgium** | 3.07 | 1.36 | 1.78 | 1.95 | 0.14 | 0.24 | 2.09* | | 0.04 | | W>M |
|  |  |  |  |  |  |  |  |  |  |  |  |
|  |  |  |  |  |  |  |  |  |  |  |  |
| **Bulgaria** | 15.99 | 3.46 | 10.84 | 3.56 | 0.37 | 0.43 | 0.19 | | 0.85 | | NS |
|  |  |  |  |  |  |  |  |  |  |  |  |
|  |  |  |  |  |  |  |  |  |  |  |  |
| **Croatia** | 10.01 | 2.35 | 7.93 | 3.31 | 0.27 | 0.41 | 1.95 | | 0.05 | | NS |
|  |  |  |  |  |  |  |  |  |  |  |  |
|  |  |  |  |  |  |  |  |  |  |  |  |
| **Cyprus** | 7.56 | 3.43 | 5.17 | 5.21 | 0.42 | 0.82 | 1.93 | | 0.05 | | NS |
|  |  |  |  |  |  |  |  |  |  |  |  |
|  |  |  |  |  |  |  |  |  |  |  |  |
| **Czechia** | 10.2 | 2.18 | 7.58 | 2.11 | 0.25 | 0.29 | -0.20 | | 0.84 | | NS |
|  |  |  |  |  |  |  |  |  |  |  |  |
|  |  |  |  |  |  |  |  |  |  |  |  |
| **Denmark** | 3.48 | 1.89 | 2.03 | 2.66 | 0.22 | 0.36 | 1.82 | | 0.07 | | NS |
|  |  |  |  |  |  |  |  |  |  |  |  |
|  |  |  |  |  |  |  |  |  |  |  |  |
| **Estonia** | 8.65 | 1.61 | 4.91 | 1.23 | 0.18 | 0.16 | -1.62 | | 0.11 | | NS |
|  |  |  |  |  |  |  |  |  |  |  |  |
|  |  |  |  |  |  |  |  |  |  |  |  |
| **Finland** | 6.81 | 2.49 | 4.01 | 3.19 | 0.29 | 0.45 | 1.33 | | 0.18 | | NS |
|  |  |  |  |  |  |  |  |  |  |  |  |
|  |  |  |  |  |  |  |  |  |  |  |  |
| **France** | 2.70 | 0.96 | 1.46 | 1.23 | 0.11 | 0.18 | 1.24 | | 0.21 | | NS |
|  |  |  |  |  |  |  |  |  |  |  |  |
|  |  |  |  |  |  |  |  |  |  |  |  |
| **Germany** | 5.39 | 1.9 | 3.31 | 3.01 | 0.21 | 0.38 | 2.54* | | 0.01 | | W>M |
|  |  |  |  |  |  |  |  |  |  |  |  |
|  |  |  |  |  |  |  |  |  |  |  |  |
| **Greece** | 5.13 | 2.38 | 3.45 | 4.47 | 0.29 | 0.6 | 3.12*^†^ | | 0 | | W>M |
|  |  |  |  |  |  |  |  |  |  |  |  |
|  |  |  |  |  |  |  |  |  |  |  |  |
| **Hungary** | 12.4 | 2.79 | 8.41 | 3.03 | 0.27 | 0.32 | 0.56 | | 0.57 | | NS |
|  |  |  |  |  |  |  |  |  |  |  |  |
|  |  |  |  |  |  |  |  |  |  |  |  |
| **Ireland** | 4.49 | 1.81 | 2.73 | 2.65 | 0.21 | 0.37 | 1.97* | | 0.05 | | W>M |
|  |  |  |  |  |  |  |  |  |  |  |  |
|  |  |  |  |  |  |  |  |  |  |  |  |
| **Italy** | 3.61 | 1.23 | 2.26 | 1.6 | 0.16 | 0.27 | 1.21 | | 0.23 | | NS |
|  |  |  |  |  |  |  |  |  |  |  |  |
|  |  |  |  |  |  |  |  |  |  |  |  |
| **Latvia** | 15.32 | 3.2 | 8.59 | 3.04 | 0.37 | 0.37 | -0.30 | | 0.76 | | NS |
|  |  |  |  |  |  |  |  |  |  |  |  |
|  |  |  |  |  |  |  |  |  |  |  |  |
| **Lithuania** | 16.27 | 3.91 | 10.88 | 3.95 | 0.39 | 0.48 | 0.08 | | 0.94 | | NS |
|  |  |  |  |  |  |  |  |  |  |  |  |
|  |  |  |  |  |  |  |  |  |  |  |  |
| **Luxembourg** | 3.82 | 1.98 | 2.27 | 2.47 | 0.21 | 0.34 | 1.23 | | 0.22 | | NS |
|  |  |  |  |  |  |  |  |  |  |  |  |
|  |  |  |  |  |  |  |  |  |  |  |  |
| **Malta** | 5.06 | 2.24 | 4.9 | 5.05 | 0.26 | 0.77 | 3.46*^†^ | | 0 | | W>M |
|  |  |  |  |  |  |  |  |  |  |  |  |
|  |  |  |  |  |  |  |  |  |  |  |  |
| **Netherlands** | 3.26 | 1.31 | 1.87 | 1.73 | 0.15 | 0.25 | 1.43 | | 0.15 | | NS |
|  |  |  |  |  |  |  |  |  |  |  |  |
|  |  |  |  |  |  |  |  |  |  |  |  |
| **Poland** | 8.38 | 1.94 | 5.99 | 1.96 | 0.22 | 0.29 | 0.04 | | 0.97 | | NS |
|  |  |  |  |  |  |  |  |  |  |  |  |
|  |  |  |  |  |  |  |  |  |  |  |  |
| **Portugal** | 2.74 | 1.76 | 1.72 | 2.27 | 0.19 | 0.33 | 1.31 | | 0.19 | | NS |
|  |  |  |  |  |  |  |  |  |  |  |  |
|  |  |  |  |  |  |  |  |  |  |  |  |
| **Romania** | 11.82 | 2.79 | 8.16 | 2.95 | 0.29 | 0.31 | 0.37 | | 0.71 | | NS |
|  |  |  |  |  |  |  |  |  |  |  |  |
|  |  |  |  |  |  |  |  |  |  |  |  |
| **Slovakia** | 14.84 | 3.34 | 11.49 | 3.94 | 0.39 | 0.5 | 0.95 | | 0.34 | | NS |
|  |  |  |  |  |  |  |  |  |  |  |  |
|  |  |  |  |  |  |  |  |  |  |  |  |
| **Slovenia** | 4.63 | 0.98 | 2.29 | 0.69 | 0.12 | 0.09 | -1.95 | | 0.05 | | NS |
|  |  |  |  |  |  |  |  |  |  |  |  |
|  |  |  |  |  |  |  |  |  |  |  |  |
| **Spain** | 3.03 | 1.38 | 1.71 | 1.66 | 0.16 | 0.26 | 0.94 | | 0.35 | | NS |
|  |  |  |  |  |  |  |  |  |  |  |  |
|  |  |  |  |  |  |  |  |  |  |  |  |
| **Sweden** | 4.62 | 1.76 | 2.98 | 2.33 | 0.23 | 0.34 | 1.37 | | 0.17 | | NS |
|  |  |  |  |  |  |  |  |  |  |  |  |
|  |  |  |  |  |  |  |  |  |  |  |  |
| **European Union** | 4.46 | 1.76 | 2.81 | 2.25 | 0.17 | 0.25 | 1.65 | | 0.10 | | NS |
|  |  |  |  |  |  |  |  |  |  |  |  |
|  |  |  |  |  |  |  |  |  |  |  |  |

* Denotes z-values exceeding the 95% threshold (z = 1.96).

^†^ Denotes z-values exceeding the 99% threshold (z = 2.58).

Abbreviations: CFI, case-fatality index; MPR, mortality-prevalence ratio

| **Appendix Table 26. Z scores for Case Fatality Index attributable to High body-mass index. 2021.** | | | | | | | | | |
| --- | --- | --- | --- | --- | --- | --- | --- | --- | --- |
|  |  |  |  |  |  |  |  |  |  |
| **Country** | **SE men** | **CFI Men** | **SE women** | **CFI Women** | **SE MPR men** | **SE MPR women** | **Z score** | **P value** | **Sig** |
| **Austria** | 3.55 | 0.4 | 2.04 | 0.70 | 0.14 | 0.25 | 1.04 | 0.30 | NS |
|  |  |  |  |  |  |  |  |  |  |
|  |  |  |  |  |  |  |  |  |  |
| **Belgium** | 2.14 | 0.29 | 1.02 | 0.40 | 0.1 | 0.14 | 0.67 | 0.51 | NS |
|  |  |  |  |  |  |  |  |  |  |
|  |  |  |  |  |  |  |  |  |  |
| **Bulgaria** | 13.57 | 0.94 | 7.67 | 0.90 | 0.31 | 0.31 | -0.10 | 0.92 | NS |
|  |  |  |  |  |  |  |  |  |  |
|  |  |  |  |  |  |  |  |  |  |
| **Croatia** | 7.58 | 0.61 | 5.22 | 0.79 | 0.20 | 0.27 | 0.53 | 0.59 | NS |
|  |  |  |  |  |  |  |  |  |  |
|  |  |  |  |  |  |  |  |  |  |
| **Cyprus** | 4.60 | 0.74 | 2.38 | 1.05 | 0.26 | 0.38 | 0.69 | 0.49 | NS |
|  |  |  |  |  |  |  |  |  |  |
|  |  |  |  |  |  |  |  |  |  |
| **Czechia** | 8.30 | 0.60 | 4.83 | 0.53 | 0.200 | 0.19 | -0.27 | 0.79 | NS |
|  |  |  |  |  |  |  |  |  |  |
|  |  |  |  |  |  |  |  |  |  |
| **Denmark** | 2.08 | 0.38 | 0.96 | 0.49 | 0.13 | 0.17 | 0.53 | 0.6 | NS |
|  |  |  |  |  |  |  |  |  |  |
|  |  |  |  |  |  |  |  |  |  |
| **Estonia** | 6.36 | 0.39 | 3.22 | 0.28 | 0.13 | 0.10 | -0.65 | 0.51 | NS |
|  |  |  |  |  |  |  |  |  |  |
|  |  |  |  |  |  |  |  |  |  |
| **Finland** | 4.52 | 0.57 | 2.16 | 0.7 | 0.19 | 0.24 | 0.43 | 0.67 | NS |
|  |  |  |  |  |  |  |  |  |  |
|  |  |  |  |  |  |  |  |  |  |
| **France** | 1.78 | 0.22 | 0.7 | 0.25 | 0.07 | 0.09 | 0.32 | 0.75 | NS |
|  |  |  |  |  |  |  |  |  |  |
|  |  |  |  |  |  |  |  |  |  |
| **Germany** | 3.95 | 0.46 | 1.71 | 0.60 | 0.16 | 0.20 | 0.59 | 0.56 | NS |
|  |  |  |  |  |  |  |  |  |  |
|  |  |  |  |  |  |  |  |  |  |
| **Greece** | 4.77 | 0.82 | 2.38 | 1.24 | 0.27 | 0.42 | 0.83 | 0.40 | NS |
|  |  |  |  |  |  |  |  |  |  |
|  |  |  |  |  |  |  |  |  |  |
| **Hungary** | 10.88 | 0.75 | 6.00 | 0.69 | 0.24 | 0.23 | -0.16 | 0.87 | NS |
|  |  |  |  |  |  |  |  |  |  |
|  |  |  |  |  |  |  |  |  |  |
| **Ireland** | 3.18 | 0.44 | 1.47 | 0.59 | 0.15 | 0.2 | 0.57 | 0.57 | NS |
|  |  |  |  |  |  |  |  |  |  |
|  |  |  |  |  |  |  |  |  |  |
| **Italy** | 2.48 | 0.31 | 1.2 | 0.39 | 0.12 | 0.14 | 0.43 | 0.67 | NS |
|  |  |  |  |  |  |  |  |  |  |
|  |  |  |  |  |  |  |  |  |  |
| **Latvia** | 11.35 | 0.80 | 6.36 | 0.80 | 0.27 | 0.27 | 0.01 | 0.99 | NS |
|  |  |  |  |  |  |  |  |  |  |
|  |  |  |  |  |  |  |  |  |  |
| **Lithuania** | 13.64 | 1.00 | 7.54 | 0.97 | 0.33 | 0.33 | -0.06 | 0.96 | NS |
|  |  |  |  |  |  |  |  |  |  |
|  |  |  |  |  |  |  |  |  |  |
| **Luxembourg** | 2.73 | 0.45 | 1.34 | 0.56 | 0.15 | 0.20 | 0.46 | 0.65 | NS |
|  |  |  |  |  |  |  |  |  |  |
|  |  |  |  |  |  |  |  |  |  |
| **Malta** | 3.26 | 0.50 | 2.48 | 1.16 | 0.17 | 0.39 | 1.56 | 0.12 | NS |
|  |  |  |  |  |  |  |  |  |  |
|  |  |  |  |  |  |  |  |  |  |
| **Netherlands** | 1.89 | 0.25 | 0.97 | 0.37 | 0.09 | 0.13 | 0.81 | 0.42 | NS |
|  |  |  |  |  |  |  |  |  |  |
|  |  |  |  |  |  |  |  |  |  |
| **Poland** | 6.86 | 0.52 | 3.97 | 0.56 | 0.18 | 0.19 | 0.15 | 0.88 | NS |
|  |  |  |  |  |  |  |  |  |  |
|  |  |  |  |  |  |  |  |  |  |
| **Portugal** | 2.16 | 0.45 | 1.14 | 0.62 | 0.15 | 0.23 | 0.59 | 0.56 | NS |
|  |  |  |  |  |  |  |  |  |  |
|  |  |  |  |  |  |  |  |  |  |
| **Romania** | 9.73 | 0.73 | 5.81 | 0.65 | 0.24 | 0.23 | -0.23 | 0.82 | NS |
|  |  |  |  |  |  |  |  |  |  |
|  |  |  |  |  |  |  |  |  |  |
| **Slovakia** | 10.98 | 0.88 | 7.86 | 1.02 | 0.29 | 0.34 | 0.31 | 0.76 | NS |
|  |  |  |  |  |  |  |  |  |  |
|  |  |  |  |  |  |  |  |  |  |
| **Slovenia** | 3.68 | 0.27 | 1.44 | 0.16 | 0.09 | 0.06 | -0.96 | 0.34 | NS |
|  |  |  |  |  |  |  |  |  |  |
|  |  |  |  |  |  |  |  |  |  |
| **Spain** | 2.65 | 0.41 | 1.08 | 0.47 | 0.14 | 0.16 | 0.29 | 0.77 | NS |
|  |  |  |  |  |  |  |  |  |  |
|  |  |  |  |  |  |  |  |  |  |
| **Sweden** | 2.46 | 0.36 | 1.45 | 0.47 | 0.13 | 0.17 | 0.52 | 0.60 | NS |
|  |  |  |  |  |  |  |  |  |  |
|  |  |  |  |  |  |  |  |  |  |
| **European Union** | 3.95 | 0.45 | 2.07 | 0.54 | 0.15 | 0.18 | 0.36 | 0.72 | NS |
|  |  |  |  |  |  |  |  |  |  |
|  |  |  |  |  |  |  |  |  |  |

* Denotes z-values exceeding the 95% threshold (z = 1.96).

^†^ Denotes z-values exceeding the 99% threshold (z = 2.58).

Abbreviations: CFI, case-fatality index

MPR, mortality-prevalence ratio

| **Appendix Table 27. Z scores for Case Fatality Index attributable to High fasting plasma glucose. 2021.** | | | | | | | | | |
| --- | --- | --- | --- | --- | --- | --- | --- | --- | --- |
|  |  |  |  |  |  |  |  |  |  |
| **Country** | **SE men** | **CFI Men** | **SE women** | **CFI Women** | **SE MPR men** | **SE MPR women** | **Z score** | **P value** | **Sig** |
| **Austria** | 0.86 | 0.34 | 0.58 | 0.57 | 0.04 | 0.07 | 2.80*^†^ | 0.01 | W>M |
|  |  |  |  |  |  |  |  |  |  |
|  |  |  |  |  |  |  |  |  |  |
| **Belgium** | 0.74 | 0.35 | 0.38 | 0.43 | 0.04 | 0.06 | 1.15 | 0.25 | NS |
|  |  |  |  |  |  |  |  |  |  |
|  |  |  |  |  |  |  |  |  |  |
| **Bulgaria** | 4.31 | 1.05 | 2.45 | 1.04 | 0.10 | 0.10 | -0.05 | 0.96 | NS |
|  |  |  |  |  |  |  |  |  |  |
|  |  |  |  |  |  |  |  |  |  |
| **Croatia** | 2.61 | 0.76 | 1.89 | 1.03 | 0.07 | 0.10 | 2.15* | 0.03 | W>M |
|  |  |  |  |  |  |  |  |  |  |
|  |  |  |  |  |  |  |  |  |  |
| **Cyprus** | 1.84 | 1.03 | 1.08 | 1.48 | 0.11 | 0.18 | 2.16* | 0.03 | W>M |
|  |  |  |  |  |  |  |  |  |  |
|  |  |  |  |  |  |  |  |  |  |
| **Czechia** | 3.26 | 0.82 | 1.97 | 0.76 | 0.08 | 0.08 | -0.58 | 0.56 | NS |
|  |  |  |  |  |  |  |  |  |  |
|  |  |  |  |  |  |  |  |  |  |
| **Denmark** | 0.64 | 0.40 | 0.31 | 0.45 | 0.04 | 0.06 | 0.72 | 0.47 | NS |
|  |  |  |  |  |  |  |  |  |  |
|  |  |  |  |  |  |  |  |  |  |
| **Estonia** | 1.81 | 0.37 | 0.97 | 0.26 | 0.04 | 0.03 | -2.05 | 0.04 | M>W |
|  |  |  |  |  |  |  |  |  |  |
|  |  |  |  |  |  |  |  |  |  |
| **Finland** | 1.62 | 0.77 | 0.95 | 0.99 | 0.07 | 0.11 | 1.68 | 0.09 | NS |
|  |  |  |  |  |  |  |  |  |  |
|  |  |  |  |  |  |  |  |  |  |
| **France** | 0.53 | 0.22 | 0.21 | 0.21 | 0.02 | 0.03 | -0.13 | 0.9 | NS |
|  |  |  |  |  |  |  |  |  |  |
|  |  |  |  |  |  |  |  |  |  |
| **Germany** | 1.19 | 0.56 | 0.79 | 0.8 | 0.05 | 0.09 | 2.28* | 0.02 | W>M |
|  |  |  |  |  |  |  |  |  |  |
|  |  |  |  |  |  |  |  |  |  |
| **Greece** | 1.11 | 0.74 | 0.75 | 1.21 | 0.07 | 0.14 | 3.17*^†^ | 0 | W>M |
|  |  |  |  |  |  |  |  |  |  |
|  |  |  |  |  |  |  |  |  |  |
| **Hungary** | 3.43 | 0.79 | 1.83 | 0.76 | 0.08 | 0.07 | -0.32 | 0.75 | NS |
|  |  |  |  |  |  |  |  |  |  |
|  |  |  |  |  |  |  |  |  |  |
| **Ireland** | 0.99 | 0.48 | 0.57 | 0.62 | 0.05 | 0.08 | 1.51 | 0.13 | NS |
|  |  |  |  |  |  |  |  |  |  |
|  |  |  |  |  |  |  |  |  |  |
| **Italy** | 0.81 | 0.40 | 0.55 | 0.52 | 0.04 | 0.07 | 1.44 | 0.15 | NS |
|  |  |  |  |  |  |  |  |  |  |
|  |  |  |  |  |  |  |  |  |  |
| **Latvia** | 2.89 | 0.66 | 1.63 | 0.64 | 0.07 | 0.07 | -0.22 | 0.83 | NS |
|  |  |  |  |  |  |  |  |  |  |
|  |  |  |  |  |  |  |  |  |  |
| **Lithuania** | 2.79 | 0.72 | 1.87 | 0.73 | 0.07 | 0.08 | 0.09 | 0.93 | NS |
|  |  |  |  |  |  |  |  |  |  |
|  |  |  |  |  |  |  |  |  |  |
| **Luxembourg** | 1.01 | 0.64 | 0.51 | 0.77 | 0.06 | 0.08 | 1.28 | 0.2 | NS |
|  |  |  |  |  |  |  |  |  |  |
|  |  |  |  |  |  |  |  |  |  |
| **Malta** | 1.17 | 0.7 | 0.98 | 1.46 | 0.06 | 0.16 | 4.49*^†^ | 0 | W>M |
|  |  |  |  |  |  |  |  |  |  |
|  |  |  |  |  |  |  |  |  |  |
| **Netherlands** | 0.66 | 0.32 | 0.35 | 0.38 | 0.03 | 0.05 | 0.87 | 0.38 | NS |
|  |  |  |  |  |  |  |  |  |  |
|  |  |  |  |  |  |  |  |  |  |
| **Poland** | 2.40 | 0.72 | 1.57 | 0.76 | 0.07 | 0.08 | 0.32 | 0.75 | NS |
|  |  |  |  |  |  |  |  |  |  |
|  |  |  |  |  |  |  |  |  |  |
| **Portugal** | 0.67 | 0.62 | 0.49 | 0.89 | 0.05 | 0.10 | 2.33* | 0.02 | W>M |
|  |  |  |  |  |  |  |  |  |  |
|  |  |  |  |  |  |  |  |  |  |
| **Romania** | 2.84 | 0.70 | 1.86 | 0.7 | 0.07 | 0.07 | -0.04 | 0.97 | NS |
|  |  |  |  |  |  |  |  |  |  |
|  |  |  |  |  |  |  |  |  |  |
| **Slovakia** | 3.65 | 0.93 | 2.97 | 1.11 | 0.1 | 0.13 | 1.11 | 0.27 | NS |
|  |  |  |  |  |  |  |  |  |  |
|  |  |  |  |  |  |  |  |  |  |
| **Slovenia** | 1.20 | 0.30 | 0.59 | 0.2 | 0.03 | 0.03 | -2.37* | 0.02 | M>W |
|  |  |  |  |  |  |  |  |  |  |
|  |  |  |  |  |  |  |  |  |  |
| **Spain** | 0.71 | 0.46 | 0.44 | 0.55 | 0.04 | 0.07 | 1.18 | 0.24 | NS |
|  |  |  |  |  |  |  |  |  |  |
|  |  |  |  |  |  |  |  |  |  |
| **Sweden** | 1.13 | 0.52 | 0.67 | 0.57 | 0.06 | 0.08 | 0.50 | 0.61 | NS |
|  |  |  |  |  |  |  |  |  |  |
|  |  |  |  |  |  |  |  |  |  |
| **European Union** | 1.01 | 0.52 | 0.66 | 0.63 | 0.04 | 0.06 | 1.53 | 0.13 | NS |
|  |  |  |  |  |  |  |  |  |  |
|  |  |  |  |  |  |  |  |  |  |

* Denotes z-values exceeding the 95% threshold (z = 1.96).

^†^ Denotes z-values exceeding the 99% threshold (z = 2.58).

Abbreviations: CFI, case-fatality index MPR, mortality-prevalence ratio

| **Appendix Table 28. Z scores for Case Fatality Index attributable to Tobacco. 2021.** | | | | | | | | | |
| --- | --- | --- | --- | --- | --- | --- | --- | --- | --- |
|  |  |  |  |  |  |  |  |  |  |
| **Country** | **SE men** | **CFI Men** | **SE women** | **CFI Women** | **SE MPR men** | **SE MPR women** | **Z score** | **P value** | **Sig?** |
| **Austria** | 2.00 | 0.76 | 0.58 | 0.49 | 0.08 | 0.08 | -2.42* | 0.02 | M>W |
|  |  |  |  |  |  |  |  |  |  |
|  |  |  |  |  |  |  |  |  |  |
| **Belgium** | 1.25 | 0.56 | 0.36 | 0.34 | 0.06 | 0.05 | -2.70*^†^ | 0.01 | M>W |
|  |  |  |  |  |  |  |  |  |  |
|  |  |  |  |  |  |  |  |  |  |
| **Bulgaria** | 7.21 | 1.42 | 2.33 | 0.61 | 0.17 | 0.10 | -4.20*^†^ | 0 | M>W |
|  |  |  |  |  |  |  |  |  |  |
|  |  |  |  |  |  |  |  |  |  |
| **Croatia** | 5.08 | 1.11 | 2.73 | 0.82 | 0.14 | 0.15 | -1.43 | 0.15 | NS |
|  |  |  |  |  |  |  |  |  |  |
|  |  |  |  |  |  |  |  |  |  |
| **Cyprus** | 3.7 | 1.7 | 0.82 | 0.74 | 0.21 | 0.14 | -3.84*^†^ | 0 | M>W |
|  |  |  |  |  |  |  |  |  |  |
|  |  |  |  |  |  |  |  |  |  |
| **Czechia** | 4.27 | 0.85 | 1.76 | 0.41 | 0.1 | 0.07 | -3.52*^†^ | 0 | M>W |
|  |  |  |  |  |  |  |  |  |  |
|  |  |  |  |  |  |  |  |  |  |
| **Denmark** | 1.52 | 0.86 | 0.5 | 0.65 | 0.1 | 0.09 | -1.51 | 0.13 | NS |
|  |  |  |  |  |  |  |  |  |  |
|  |  |  |  |  |  |  |  |  |  |
| **Estonia** | 3.21 | 0.56 | 0.67 | 0.13 | 0.07 | 0.02 | -6.02*^†^ | 0 | M>W |
|  |  |  |  |  |  |  |  |  |  |
|  |  |  |  |  |  |  |  |  |  |
| **Finland** | 1.80 | 0.67 | 0.39 | 0.29 | 0.08 | 0.05 | -4.06*^†^ | 0 | M>W |
|  |  |  |  |  |  |  |  |  |  |
|  |  |  |  |  |  |  |  |  |  |
| **France** | 0.86 | 0.33 | 0.18 | 0.17 | 0.04 | 0.03 | -3.57*^†^ | 0 | M>W |
|  |  |  |  |  |  |  |  |  |  |
|  |  |  |  |  |  |  |  |  |  |
| **Germany** | 1.94 | 0.67 | 0.51 | 0.41 | 0.08 | 0.06 | -2.61*^†^ | 0.01 | M>W |
|  |  |  |  |  |  |  |  |  |  |
|  |  |  |  |  |  |  |  |  |  |
| **Greece** | 2.72 | 1.71 | 0.91 | 1.18 | 0.15 | 0.17 | -2.34* | 0.02 | M>W |
|  |  |  |  |  |  |  |  |  |  |
|  |  |  |  |  |  |  |  |  |  |
| **Hungary** | 4.36 | 0.9 | 1.61 | 0.41 | 0.1 | 0.06 | -4.20*^†^ | 0 | M>W |
|  |  |  |  |  |  |  |  |  |  |
|  |  |  |  |  |  |  |  |  |  |
| **Ireland** | 1.47 | 0.6 | 0.67 | 0.54 | 0.07 | 0.09 | -0.48 | 0.63 | NS |
|  |  |  |  |  |  |  |  |  |  |
|  |  |  |  |  |  |  |  |  |  |
| **Italy** | 1.26 | 0.51 | 0.31 | 0.24 | 0.06 | 0.04 | -3.55*^†^ | 0 | M>W |
|  |  |  |  |  |  |  |  |  |  |
|  |  |  |  |  |  |  |  |  |  |
| **Latvia** | 5.53 | 1.26 | 1.47 | 0.32 | 0.13 | 0.07 | -6.29*^†^ | 0 | M>W |
|  |  |  |  |  |  |  |  |  |  |
|  |  |  |  |  |  |  |  |  |  |
| **Lithuania** | 6.85 | 1.52 | 1.31 | 0.29 | 0.17 | 0.06 | -6.94*^†^ | 0 | M>W |
|  |  |  |  |  |  |  |  |  |  |
|  |  |  |  |  |  |  |  |  |  |
| **Luxembourg** | 1.70 | 0.69 | 0.47 | 0.40 | 0.1 | 0.07 | -2.35* | 0.02 | M>W |
|  |  |  |  |  |  |  |  |  |  |
|  |  |  |  |  |  |  |  |  |  |
| **Malta** | 1.80 | 0.83 | 0.73 | 0.70 | 0.1 | 0.12 | -0.84 | 0.4 | NS |
|  |  |  |  |  |  |  |  |  |  |
|  |  |  |  |  |  |  |  |  |  |
| **Netherlands** | 1.14 | 0.45 | 0.4 | 0.36 | 0.06 | 0.06 | -1.12 | 0.26 | NS |
|  |  |  |  |  |  |  |  |  |  |
|  |  |  |  |  |  |  |  |  |  |
| **Poland** | 3.35 | 0.81 | 0.95 | 0.32 | 0.09 | 0.05 | -4.70*^†^ | 0 | M>W |
|  |  |  |  |  |  |  |  |  |  |
|  |  |  |  |  |  |  |  |  |  |
| **Portugal** | 0.86 | 0.65 | 0.21 | 0.23 | 0.06 | 0.05 | -5.05*^†^ | 0 | M>W |
|  |  |  |  |  |  |  |  |  |  |
|  |  |  |  |  |  |  |  |  |  |
| **Romania** | 4.24 | 0.95 | 1.55 | 0.34 | 0.11 | 0.06 | -4.97*^†^ | 0 | M>W |
|  |  |  |  |  |  |  |  |  |  |
|  |  |  |  |  |  |  |  |  |  |
| **Slovakia** | 6.52 | 1.32 | 2.32 | 0.48 | 0.17 | 0.10 | -4.20*^†^ | 0 | M>W |
|  |  |  |  |  |  |  |  |  |  |
|  |  |  |  |  |  |  |  |  |  |
| **Slovenia** | 1.62 | 0.33 | 0.42 | 0.10 | 0.04 | 0.02 | -4.56*^†^ | 0 | M>W |
|  |  |  |  |  |  |  |  |  |  |
|  |  |  |  |  |  |  |  |  |  |
| **Spain** | 1.21 | 0.61 | 0.25 | 0.22 | 0.07 | 0.04 | -5.02*^†^ | 0 | M>W |
|  |  |  |  |  |  |  |  |  |  |
|  |  |  |  |  |  |  |  |  |  |
| **Sweden** | 1.77 | 0.63 | 0.69 | 0.48 | 0.09 | 0.08 | -1.24 | 0.22 | NS |
|  |  |  |  |  |  |  |  |  |  |
|  |  |  |  |  |  |  |  |  |  |
| **European Union** | 1.64 | 0.70 | 0.44 | 0.34 | 0.07 | 0.04 | -4.56*^†^ | 0 | M>W |
|  |  |  |  |  |  |  |  |  |  |
|  |  |  |  |  |  |  |  |  |  |

* Denotes z-values exceeding the 95% threshold (z = 1.96).

^†^ Denotes z-values exceeding the 99% threshold (z = 2.58).

Abbreviation: CFI, case-fatality index MPR, mortality-prevalence ratio

| **Appendix Table 29. Z scores for Case Fatality Index attributable to Low physical activity. 2021.** | | | | | | | | | |
| --- | --- | --- | --- | --- | --- | --- | --- | --- | --- |
|  |  |  |  |  |  |  |  |  |  |
| **Country** | **SE men** | **CFR Men** | **SE women** | **CFR Women** | **SE MPR men** | **SE MPR women** | **Z score** | **P value** | **Sig** |
| **Austria** | 0.64 | 0.04 | 0.94 | 0.24 | 0.03 | 0.12 | 1.64 | 0.1 | NS |
|  |  |  |  |  |  |  |  |  |  |
|  |  |  |  |  |  |  |  |  |  |
| **Belgium** | 0.46 | 0.04 | 0.47 | 0.14 | 0.03 | 0.07 | 1.34 | 0.18 | NS |
|  |  |  |  |  |  |  |  |  |  |
|  |  |  |  |  |  |  |  |  |  |
| **Bulgaria** | 1.08 | 0.05 | 1.89 | 0.19 | 0.03 | 0.08 | 1.65 | 0.1 | NS |
|  |  |  |  |  |  |  |  |  |  |
|  |  |  |  |  |  |  |  |  |  |
| **Croatia** | 1.20 | 0.06 | 2.06 | 0.26 | 0.04 | 0.11 | 1.71 | 0.09 | NS |
|  |  |  |  |  |  |  |  |  |  |
|  |  |  |  |  |  |  |  |  |  |
| **Cyprus** | 1.13 | 0.11 | 1.14 | 0.35 | 0.07 | 0.19 | 1.18 | 0.24 | NS |
|  |  |  |  |  |  |  |  |  |  |
|  |  |  |  |  |  |  |  |  |  |
| **Czechia** | 1.25 | 0.06 | 1.68 | 0.17 | 0.03 | 0.07 | 1.47 | 0.14 | NS |
|  |  |  |  |  |  |  |  |  |  |
|  |  |  |  |  |  |  |  |  |  |
| **Denmark** | 0.4 | 0.04 | 0.46 | 0.17 | 0.03 | 0.09 | 1.43 | 0.15 | NS |
|  |  |  |  |  |  |  |  |  |  |
|  |  |  |  |  |  |  |  |  |  |
| **Estonia** | 0.86 | 0.03 | 0.99 | 0.08 | 0.02 | 0.03 | 1.05 | 0.29 | NS |
|  |  |  |  |  |  |  |  |  |  |
|  |  |  |  |  |  |  |  |  |  |
| **Finland** | 0.89 | 0.07 | 0.9 | 0.2 | 0.04 | 0.10 | 1.14 | 0.26 | NS |
|  |  |  |  |  |  |  |  |  |  |
|  |  |  |  |  |  |  |  |  |  |
| **France** | 0.42 | 0.03 | 0.34 | 0.10 | 0.02 | 0.05 | 1.28 | 0.2 | NS |
|  |  |  |  |  |  |  |  |  |  |
|  |  |  |  |  |  |  |  |  |  |
| **Germany** | 0.41 | 0.03 | 0.7 | 0.17 | 0.02 | 0.08 | 1.67 | 0.1 | NS |
|  |  |  |  |  |  |  |  |  |  |
|  |  |  |  |  |  |  |  |  |  |
| **Greece** | 0.44 | 0.05 | 0.82 | 0.31 | 0.03 | 0.15 | 1.75 | 0.08 | NS |
|  |  |  |  |  |  |  |  |  |  |
|  |  |  |  |  |  |  |  |  |  |
| **Hungary** | 1.29 | 0.06 | 1.85 | 0.18 | 0.03 | 0.07 | 1.45 | 0.15 | NS |
|  |  |  |  |  |  |  |  |  |  |
|  |  |  |  |  |  |  |  |  |  |
| **Ireland** | 0.82 | 0.08 | 0.73 | 0.23 | 0.04 | 0.10 | 1.44 | 0.15 | NS |
|  |  |  |  |  |  |  |  |  |  |
|  |  |  |  |  |  |  |  |  |  |
| **Italy** | 0.63 | 0.05 | 0.63 | 0.18 | 0.04 | 0.08 | 1.43 | 0.15 | NS |
|  |  |  |  |  |  |  |  |  |  |
|  |  |  |  |  |  |  |  |  |  |
| **Latvia** | 0.94 | 0.05 | 1.21 | 0.12 | 0.03 | 0.06 | 1.19 | 0.24 | NS |
|  |  |  |  |  |  |  |  |  |  |
|  |  |  |  |  |  |  |  |  |  |
| **Lithuania** | 2.46 | 0.13 | 2.56 | 0.27 | 0.06 | 0.11 | 1.06 | 0.29 | NS |
|  |  |  |  |  |  |  |  |  |  |
|  |  |  |  |  |  |  |  |  |  |
| **Luxembourg** | 0.53 | 0.05 | 0.54 | 0.16 | 0.03 | 0.08 | 1.24 | 0.21 | NS |
|  |  |  |  |  |  |  |  |  |  |
|  |  |  |  |  |  |  |  |  |  |
| **Malta** | 0.88 | 0.09 | 1.18 | 0.45 | 0.05 | 0.19 | 1.87 | 0.06 | NS |
|  |  |  |  |  |  |  |  |  |  |
|  |  |  |  |  |  |  |  |  |  |
| **Netherlands** | 0.3 | 0.02 | 0.32 | 0.08 | 0.02 | 0.05 | 1.16 | 0.24 | NS |
|  |  |  |  |  |  |  |  |  |  |
|  |  |  |  |  |  |  |  |  |  |
| **Poland** | 1.25 | 0.07 | 1.41 | 0.17 | 0.04 | 0.07 | 1.22 | 0.22 | NS |
|  |  |  |  |  |  |  |  |  |  |
|  |  |  |  |  |  |  |  |  |  |
| **Portugal** | 0.41 | 0.06 | 0.46 | 0.19 | 0.03 | 0.10 | 1.26 | 0.21 | NS |
|  |  |  |  |  |  |  |  |  |  |
|  |  |  |  |  |  |  |  |  |  |
| **Romania** | 1.04 | 0.06 | 1.63 | 0.16 | 0.03 | 0.07 | 1.45 | 0.15 | NS |
|  |  |  |  |  |  |  |  |  |  |
|  |  |  |  |  |  |  |  |  |  |
| **Slovakia** | 1.21 | 0.07 | 1.95 | 0.19 | 0.03 | 0.09 | 1.36 | 0.17 | NS |
|  |  |  |  |  |  |  |  |  |  |
|  |  |  |  |  |  |  |  |  |  |
| **Slovenia** | 0.44 | 0.02 | 0.44 | 0.04 | 0.02 | 0.02 | 0.69 | 0.49 | NS |
|  |  |  |  |  |  |  |  |  |  |
|  |  |  |  |  |  |  |  |  |  |
| **Spain** | 0.41 | 0.05 | 0.39 | 0.12 | 0.02 | 0.06 | 1.13 | 0.26 | NS |
|  |  |  |  |  |  |  |  |  |  |
|  |  |  |  |  |  |  |  |  |  |
| **Sweden** | 0.37 | 0.03 | 0.46 | 0.11 | 0.03 | 0.06 | 1.22 | 0.22 | NS |
|  |  |  |  |  |  |  |  |  |  |
|  |  |  |  |  |  |  |  |  |  |
| **European Union** | 0.38 | 0.04 | 0.57 | 0.15 | 0.02 | 0.05 | 1.96 | 0.05 | NS |
|  |  |  |  |  |  |  |  |  |  |
|  |  |  |  |  |  |  |  |  |  |

* Denotes z-values exceeding the 95% threshold (z = 1.96).

^†^ Denotes z-values exceeding the 99% threshold (z = 2.58).

Abbreviation: CFI, case-fatality index MPR, mortality-prevalence ratio

| **Appendix Table 30. Z scores for Case Fatality Index attributable to Air pollution. 2021.** | | | | | | | | | |
| --- | --- | --- | --- | --- | --- | --- | --- | --- | --- |
|  |  |  |  |  |  |  |  |  |  |
| **Country** | **SE men** | **CFI Men** | **SE women** | **CFI Women** | **SE MPR men** | **SE MPR women** | **Z score** | **P value** | **Sig?** |
| **Austria** | 1.72 | 0.37 | 1 | 0.63 | 0.07 | 0.13 | 1.81 | 0.07 | NS |
|  |  |  |  |  |  |  |  |  |  |
|  |  |  |  |  |  |  |  |  |  |
| **Belgium** | 1 | 0.25 | 0.51 | 0.34 | 0.05 | 0.07 | 0.96 | 0.34 | NS |
|  |  |  |  |  |  |  |  |  |  |
|  |  |  |  |  |  |  |  |  |  |
| **Bulgaria** | 8.91 | 1.12 | 5.31 | 1.15 | 0.21 | 0.21 | 0.10 | 0.92 | NS |
|  |  |  |  |  |  |  |  |  |  |
|  |  |  |  |  |  |  |  |  |  |
| **Croatia** | 3.78 | 0.65 | 2.86 | 0.9 | 0.1 | 0.15 | 1.37 | 0.17 | NS |
|  |  |  |  |  |  |  |  |  |  |
|  |  |  |  |  |  |  |  |  |  |
| **Cyprus** | 2.99 | 0.94 | 1.69 | 1.45 | 0.17 | 0.27 | 1.58 | 0.11 | NS |
|  |  |  |  |  |  |  |  |  |  |
|  |  |  |  |  |  |  |  |  |  |
| **Czechia** | 3.66 | 0.54 | 2.17 | 0.51 | 0.09 | 0.09 | -0.24 | 0.81 | NS |
|  |  |  |  |  |  |  |  |  |  |
|  |  |  |  |  |  |  |  |  |  |
| **Denmark** | 1.03 | 0.28 | 0.52 | 0.38 | 0.07 | 0.1 | 0.86 | 0.39 | NS |
|  |  |  |  |  |  |  |  |  |  |
|  |  |  |  |  |  |  |  |  |  |
| **Estonia** | 2.68 | 0.13 | 1.31 | 0.09 | 0.06 | 0.04 | -0.45 | 0.66 | NS |
|  |  |  |  |  |  |  |  |  |  |
|  |  |  |  |  |  |  |  |  |  |
| **Finland** | 1.26 | 0.1 | 0.61 | 0.13 | 0.06 | 0.07 | 0.29 | 0.77 | NS |
|  |  |  |  |  |  |  |  |  |  |
|  |  |  |  |  |  |  |  |  |  |
| **France** | 0.76 | 0.15 | 0.35 | 0.19 | 0.03 | 0.05 | 0.79 | 0.43 | NS |
|  |  |  |  |  |  |  |  |  |  |
|  |  |  |  |  |  |  |  |  |  |
| **Germany** | 1.64 | 0.33 | 0.83 | 0.46 | 0.07 | 0.10 | 1.11 | 0.27 | NS |
|  |  |  |  |  |  |  |  |  |  |
|  |  |  |  |  |  |  |  |  |  |
| **Greece** | 2.13 | 0.77 | 1.25 | 1.38 | 0.12 | 0.22 | 2.39* | 0.02 | W>M |
|  |  |  |  |  |  |  |  |  |  |
|  |  |  |  |  |  |  |  |  |  |
| **Hungary** | 5.79 | 0.65 | 3.83 | 0.69 | 0.13 | 0.15 | 0.19 | 0.85 | NS |
|  |  |  |  |  |  |  |  |  |  |
|  |  |  |  |  |  |  |  |  |  |
| **Ireland** | 1.14 | 0.19 | 0.59 | 0.28 | 0.06 | 0.08 | 0.88 | 0.38 | NS |
|  |  |  |  |  |  |  |  |  |  |
|  |  |  |  |  |  |  |  |  |  |
| **Italy** | 1.38 | 0.38 | 0.75 | 0.48 | 0.07 | 0.09 | 0.91 | 0.36 | NS |
|  |  |  |  |  |  |  |  |  |  |
|  |  |  |  |  |  |  |  |  |  |
| **Latvia** | 5.95 | 0.67 | 3.27 | 0.62 | 0.14 | 0.14 | -0.26 | 0.79 | NS |
|  |  |  |  |  |  |  |  |  |  |
|  |  |  |  |  |  |  |  |  |  |
| **Lithuania** | 5.55 | 0.59 | 3.09 | 0.59 | 0.14 | 0.14 | 0 | 1 | NS |
|  |  |  |  |  |  |  |  |  |  |
|  |  |  |  |  |  |  |  |  |  |
| **Luxembourg** | 1.07 | 0.23 | 0.51 | 0.29 | 0.06 | 0.08 | 0.61 | 0.54 | NS |
|  |  |  |  |  |  |  |  |  |  |
|  |  |  |  |  |  |  |  |  |  |
| **Malta** | 1.71 | 0.51 | 1.33 | 1.11 | 0.09 | 0.21 | 2.60*^†^ | 0.01 | W>M |
|  |  |  |  |  |  |  |  |  |  |
|  |  |  |  |  |  |  |  |  |  |
| **Netherlands** | 0.99 | 0.25 | 0.5 | 0.34 | 0.05 | 0.07 | 1.09 | 0.28 | NS |
|  |  |  |  |  |  |  |  |  |  |
|  |  |  |  |  |  |  |  |  |  |
| **Poland** | 4.55 | 0.74 | 2.63 | 0.76 | 0.12 | 0.13 | 0.08 | 0.93 | NS |
|  |  |  |  |  |  |  |  |  |  |
|  |  |  |  |  |  |  |  |  |  |
| **Portugal** | 0.79 | 0.2 | 0.42 | 0.28 | 0.06 | 0.09 | 0.73 | 0.46 | NS |
|  |  |  |  |  |  |  |  |  |  |
|  |  |  |  |  |  |  |  |  |  |
| **Romania** | 5.62 | 0.71 | 3.68 | 0.73 | 0.14 | 0.14 | 0.06 | 0.95 | NS |
|  |  |  |  |  |  |  |  |  |  |
|  |  |  |  |  |  |  |  |  |  |
| **Slovakia** | 5.73 | 0.91 | 4.05 | 1.04 | 0.15 | 0.18 | 0.57 | 0.57 | NS |
|  |  |  |  |  |  |  |  |  |  |
|  |  |  |  |  |  |  |  |  |  |
| **Slovenia** | 1.64 | 0.23 | 0.75 | 0.16 | 0.04 | 0.03 | -1.34 | 0.18 | NS |
|  |  |  |  |  |  |  |  |  |  |
|  |  |  |  |  |  |  |  |  |  |
| **Spain** | 0.86 | 0.21 | 0.4 | 0.25 | 0.05 | 0.06 | 0.59 | 0.55 | NS |
|  |  |  |  |  |  |  |  |  |  |
|  |  |  |  |  |  |  |  |  |  |
| **Sweden** | 0.91 | 0.1 | 0.51 | 0.13 | 0.05 | 0.06 | 0.36 | 0.72 | NS |
|  |  |  |  |  |  |  |  |  |  |
|  |  |  |  |  |  |  |  |  |  |
| **European Union** | 1.75 | 0.4 | 1.06 | 0.51 | 0.07 | 0.10 | 0.96 | 0.34 | NS |
|  |  |  |  |  |  |  |  |  |  |
|  |  |  |  |  |  |  |  |  |  |

* Denotes z-values exceeding the 95% threshold (z = 1.96).

^†^ Denotes z-values exceeding the 99% threshold (z = 2.58).

Abbreviation: CFI, case-fatality index MPR, mortality-prevalence ratio

| **Table 31: Z values for Case Fatality Index attributable to the 7 Dietary risk factors** | | | | | | | | |
| --- | --- | --- | --- | --- | --- | --- | --- | --- |
| **Country** | **Diet high in processed meat** | **Diet low in fiber** | **Diet low in whole grain** | **Diet low in vegetables** | **Diet low in nuts and seeds** | **Diet low in seafood omega-3 fatty acids** | **Diet low in fruits** | **Diet high in sodium** |
| **Austria** | 3.01 | 3.12 | -5.12 | NS | NS | 3.21 | NS | NS |
| **Belgium** | NS | NS | -5.89 | NS | NS | 2.89 | NS | NS |
| **Bulgaria** | -4.67 | -4.12 | -5.23 | NS | NS | NS | NS | 2.89 |
| **Croatia** | NS | -3.78 | -4.78 | NS | NS | NS | NS | 2.45 |
| **Cyprus** | 2.45 | -4.56 | -5.23 | NS | 3.56 | 3.56 | NS | NS |
| **Czechia** | -4.21 | -4.01 | -5.89 | NS | NS | NS | NS | NS |
| **Denmark** | 3.12 | 3.56 | -6.23 | NS | NS | NS | NS | NS |
| **Estonia** | -5.92 | -5.01 | -7.12 | NS | NS | 2.89 | NS | NS |
| **Finland** | NS | NS | -6.23 | NS | NS | NS | NS | NS |
| **France** | NS | NS | -7.01 | NS | NS | 3.29 | NS | NS |
| **Germany** | NS | NS | -6.78 | NS | NS | 2.78 | NS | NS |
| **Greece** | 3.05 | 4.67 | -5.56 | 2.23 | 2.89 | 4.12 | NS | NS |
| **Hungary** | -4.89 | -4.23 | -5.01 | NS | NS | 3.56 | NS | 2.58 |
| **Ireland** | NS | NS | -6.12 | NS | NS | NS | NS | NS |
| **Italy** | NS | NS | -5.89 | NS | NS | NS | NS | NS |
| **Latvia** | -4.87 | -4.89 | -5.89 | NS | NS | NS | NS | NS |
| **Lithuania** | -5.01 | -4.78 | -5.45 | NS | NS | NS | NS | NS |
| **Luxembourg** | NS | 3.01 | -6.23 | NS | NS | NS | NS | NS |
| **Malta** | NS | 4.92 | -4.12 | 3.12 | 4.12 | 4.67 | NS | NS |
| **Netherlands** | NS | 2.68 | -6.34 | NS | NS | 3.21 | NS | NS |
| **Poland** | -3.4 | -4.12 | -5.34 | NS | NS | NS | NS | NS |
| **Portugal** | NS | NS | -6.45 | NS | NS | 3.56 | NS | NS |
| **Romania** | -4.56 | -3.45 | -5.12 | NS | NS | NS | NS | NS |
| **Slovakia** | -3.98 | -3.78 | -4.67 | NS | NS | NS | NS | -2.45 |
| **Slovenia** | -4.12 | -5.01 | -6.45 | -2.23 | -2.78 | NS | NS | NS |
| **Spain** | NS | NS | -6.78 | NS | NS | NS | NS | NS |
| **Sweden** | NS | NS | -5.34 | NS | NS | NS | NS | NS |

Abbreviations: NS, not significant

| **Appendix Table 32. Z values for Case Fatality Index attributable to Diet high in processed meat. 2021.** | | | | | | | |
| --- | --- | --- | --- | --- | --- | --- | --- |
| **Country** | **CFI**  **Women** | **95% UI (Women)** | **CFI**  **Men** | **95% UI (Men)** | **Z-Score** | **p-value** | **Sig.** |
| **Austria** | 0.096 | 0.036–0.167 | 0.053 | 0.020–0.093 | 3.01*^†^ | 0.003 | W>M |
| **Belgium** | 0.11 | 0.042–0.182 | 0.083 | 0.032–0.138 | 1.23 | 0.219 | NS |
| **Bulgaria** | 0.079 | 0.030–0.135 | 0.199 | 0.077–0.341 | -4.67*^†^ | <0.001 | M>W |
| **Croatia** | 0.004 | 0.002–0.007 | 0.003 | 0.001–0.005 | 1.87 | 0.061 | NS |
| **Cyprus** | 0.084 | 0.032–0.156 | 0.053 | 0.020–0.099 | 2.45*^†^ | 0.014 | W>M |
| **Czechia** | 0.028 | 0.011–0.050 | 0.074 | 0.028–0.133 | -4.21*^†^ | <0.001 | M>W |
| **Denmark** | 0.141 | 0.053–0.231 | 0.08 | 0.031–0.131 | 3.12*^†^ | 0.002 | W>M |
| **Estonia** | 0.128 | 0.048–0.210 | 0.409 | 0.154–0.669 | -5.92*^†^ | <0.001 | M>W |
| **Finland** | 0.099 | 0.037–0.173 | 0.09 | 0.034–0.157 | 0.37 | 0.711 | NS |
| **France** | 0.07 | 0.026–0.117 | 0.057 | 0.021–0.095 | 1.02 | 0.307 | NS |
| **Germany** | 0.209 | 0.078–0.352 | 0.158 | 0.062–0.259 | 2.14* | 0.032 | W>M |
| **Greece** | 0.086 | 0.032–0.147 | 0.05 | 0.019–0.085 | 3.05*^†^ | 0.002 | W>M |
| **Hungary** | 0.047 | 0.018–0.080 | 0.127 | 0.049–0.214 | -4.89*^†^ | <0.001 | M>W |
| **Ireland** | 0.084 | 0.032–0.143 | 0.063 | 0.024–0.107 | 1.45 | 0.147 | NS |
| **Italy** | 0.074 | 0.029–0.120 | 0.065 | 0.025–0.105 | 0.55 | 0.582 | NS |
| **Latvia** | 0.393 | 0.149–0.627 | 0.942 | 0.375–1.503 | -4.87*^†^ | <0.001 | M>W |
| **Lithuania** | 0.503 | 0.193–0.822 | 1.194 | 0.462–1.990 | -5.01*^†^ | <0.001 | M>W |
| **Luxembourg** | 0.186 | 0.070–0.310 | 0.128 | 0.048–0.213 | 1.89 | 0.059 | NS |
| **Malta** | 0.403 | 0.151–0.661 | 0.259 | 0.098–0.425 | 2.31* | 0.021 | W>M |
| **Netherlands** | 0.053 | 0.020–0.093 | 0.041 | 0.015–0.072 | 1.08 | 0.28 | NS |
| **Poland** | 0.018 | 0.007–0.030 | 0.038 | 0.014–0.062 | -3.45*^†^ | <0.001 | M>W |
| **Portugal** | 0.026 | 0.010–0.046 | 0.02 | 0.008–0.034 | 2.45* | 0.014 | W>M |
| **Romania** | 0.062 | 0.024–0.103 | 0.163 | 0.063–0.271 | -4.56*^†^ | <0.001 | M>W |
| **Slovakia** | 0.068 | 0.026–0.120 | 0.158 | 0.060–0.278 | -3.98*^†^ | <0.001 | M>W |
| **Slovenia** | 0.037 | 0.014–0.064 | 0.095 | 0.036–0.164 | -4.12*^†^ | <0.001 | M>W |
| **Spain** | 0.091 | 0.035–0.154 | 0.081 | 0.030–0.135 | 0.58 | 0.562 | NS |
| **Sweden** | 0.204 | 0.080–0.341 | 0.166 | 0.063–0.267 | 1.12 | 0.263 | NS |

* Denotes z-values exceeding the 95% threshold (z = 1.96).

^†^ Denotes z-values exceeding the 99% threshold (z = 2.58).

Abbreviation: CFI, case-fatality index

| **Appendix Table 33. Z values for Case Fatality Index attributable to Diet low in fiber. 2021.** | | | | | | | |
| --- | --- | --- | --- | --- | --- | --- | --- |
| **Country** | **CFI**  **Women** | **95% UI Women** | **CFI**  **Men** | **95% UI Men** | **Z-Score** | **p-value** | **Sig.** |
| **Austria** | 0.21 | 0.10–0.36 | 0.13 | 0.06–0.22 | 3.12*^†^ | 0.002 | W>M |
| **Belgium** | 0.19 | 0.09–0.30 | 0.14 | 0.07–0.21 | 2.45* | 0.014 | W>M |
| **Bulgaria** | 0.46 | 0.22–0.73 | 0.84 | 0.40–1.30 | -4.12*^†^ | <0.001 | M>W |
| **Croatia** | 0.4 | 0.19–0.64 | 0.64 | 0.32–1.01 | -3.78*^†^ | <0.001 | M>W |
| **Cyprus** | 0.22 | 0.10–0.34 | 0.44 | 0.21–0.68 | -4.56*^†^ | <0.001 | M>W |
| **Czechia** | 0.23 | 0.11–0.37 | 0.41 | 0.20–0.63 | -4.01*^†^ | <0.001 | M>W |
| **Denmark** | 0.23 | 0.10–0.38 | 0.13 | 0.06–0.21 | 3.56*^†^ | <0.001 | W>M |
| **Estonia** | 0.05 | 0.02–0.09 | 0.12 | 0.05–0.19 | -5.01*^†^ | <0.001 | M>W |
| **Finland** | 0.31 | 0.15–0.51 | 0.25 | 0.12–0.39 | 1.89 | 0.059 | NS |
| **France** | 0.16 | 0.07–0.24 | 0.13 | 0.06–0.20 | 1.56 | 0.119 | NS |
| **Germany** | 0.25 | 0.12–0.39 | 0.19 | 0.09–0.29 | 2.33* | 0.02 | W>M |
| **Greece** | 0.35 | 0.16–0.58 | 0.17 | 0.08–0.29 | 4.67*^†^ | <0.001 | W>M |
| **Hungary** | 0.28 | 0.14–0.45 | 0.48 | 0.24–0.74 | -4.23*^†^ | <0.001 | M>W |
| **Ireland** | 0.17 | 0.08–0.30 | 0.14 | 0.07–0.23 | 1.42 | 0.156 | NS |
| **Italy** | 0.16 | 0.08–0.26 | 0.13 | 0.06–0.21 | 1.62 | 0.105 | NS |
| **Latvia** | 0.24 | 0.11–0.39 | 0.5 | 0.24–0.79 | -4.89*^†^ | <0.001 | M>W |
| **Lithuania** | 0.12 | 0.05–0.23 | 0.24 | 0.10–0.40 | -4.78*^†^ | <0.001 | M>W |
| **Luxembourg** | 0.26 | 0.12–0.41 | 0.16 | 0.08–0.26 | 3.01*^†^ | 0.003 | W>M |
| **Malta** | 0.36 | 0.16–0.61 | 0.17 | 0.08–0.25 | 4.92*^†^ | <0.001 | W>M |
| **Netherlands** | 0.2 | 0.09–0.32 | 0.14 | 0.07–0.22 | 2.68*^†^ | 0.007 | W>M |
| **Poland** | 0.09 | 0.04–0.15 | 0.16 | 0.07–0.27 | -4.12*^†^ | <0.001 | M>W |
| **Portugal** | 0.21 | 0.09–0.35 | 0.16 | 0.07–0.26 | 1.89 | 0.059 | NS |
| **Romania** | 0.09 | 0.04–0.16 | 0.14 | 0.06–0.23 | -3.45*^†^ | <0.001 | M>W |
| **Slovakia** | 0.52 | 0.25–0.79 | 0.81 | 0.42–1.25 | -3.78*^†^ | <0.001 | M>W |
| **Slovenia** | 0.05 | 0.02–0.09 | 0.12 | 0.05–0.19 | -5.01*^†^ | <0.001 | M>W |
| **Spain** | 0.19 | 0.09–0.30 | 0.14 | 0.07–0.22 | 2.45* | 0.014 | W>M |
| **Sweden** | 0.24 | 0.11–0.41 | 0.18 | 0.08–0.29 | 2.01* | 0.044 | W>M |

* Denotes z-values exceeding the 95% threshold (z = 1.96).

^†^ Denotes z-values exceeding the 99% threshold (z = 2.58).

Abbreviation: CFI, case-fatality index

| **Appendix Table 34. Z values for Case Fatality Index attributable to Diet low in whole grain. 2021.** | | | | | | | |
| --- | --- | --- | --- | --- | --- | --- | --- |
| **Country** | **CFI**  **Women** | **95% UI Women** | **CFI**  **Men** | **95% UI Men** | **Z-Score** | **p-value** | **Sig.** |
| **Austria** | 0.5 | 0.29–0.74 | 1.12 | 0.66–1.65 | -5.12*^†^ | <0.001 | M>W |
| **Belgium** | 0.39 | 0.23–0.54 | 0.97 | 0.57–1.35 | -5.89*^†^ | <0.001 | M>W |
| **Bulgaria** | 0.83 | 0.49–1.17 | 1.66 | 0.99–2.31 | -5.23*^†^ | <0.001 | M>W |
| **Croatia** | 0.61 | 0.36–0.88 | 1.04 | 0.60–1.52 | -4.78*^†^ | <0.001 | M>W |
| **Cyprus** | 0.37 | 0.19–0.51 | 0.76 | 0.44–1.06 | -5.23*^†^ | <0.001 | M>W |
| **Czechia** | 0.26 | 0.15–0.39 | 0.57 | 0.33–0.81 | -5.89*^†^ | <0.001 | M>W |
| **Denmark** | 0.13 | 0.08–0.19 | 0.32 | 0.19–0.45 | -6.23*^†^ | <0.001 | M>W |
| **Estonia** | 0.22 | 0.13–0.32 | 0.69 | 0.41–0.99 | -7.12*^†^ | <0.001 | M>W |
| **Finland** | 0.22 | 0.13–0.32 | 0.56 | 0.33–0.79 | -6.23*^†^ | <0.001 | M>W |
| **France** | 0.11 | 0.06–0.16 | 0.31 | 0.18–0.44 | -7.01*^†^ | <0.001 | M>W |
| **Germany** | 0.15 | 0.09–0.22 | 0.39 | 0.23–0.55 | -6.78*^†^ | <0.001 | M>W |
| **Greece** | 0.38 | 0.22–0.52 | 0.79 | 0.47–1.05 | -5.56*^†^ | <0.001 | M>W |
| **Hungary** | 0.56 | 0.33–0.78 | 1.06 | 0.63–1.49 | -5.01*^†^ | <0.001 | M>W |
| **Ireland** | 0.1 | 0.06–0.15 | 0.24 | 0.14–0.35 | -6.12*^†^ | <0.001 | M>W |
| **Italy** | 0.21 | 0.12–0.30 | 0.48 | 0.29–0.66 | -5.89*^†^ | <0.001 | M>W |
| **Latvia** | 0.69 | 0.40–0.98 | 1.56 | 0.91–2.18 | -5.89*^†^ | <0.001 | M>W |
| **Lithuania** | 0.79 | 0.47–1.16 | 1.64 | 0.96–2.39 | -5.45*^†^ | <0.001 | M>W |
| **Luxembourg** | 0.12 | 0.07–0.18 | 0.29 | 0.17–0.41 | -6.23*^†^ | <0.001 | M>W |
| **Malta** | 1.57 | 0.92–2.20 | 2.45 | 1.47–3.36 | -4.12*^†^ | <0.001 | M>W |
| **Netherlands** | 0.11 | 0.06–0.16 | 0.26 | 0.15–0.38 | -6.34*^†^ | <0.001 | M>W |
| **Poland** | 0.47 | 0.27–0.66 | 0.99 | 0.57–1.41 | -5.34*^†^ | <0.001 | M>W |
| **Portugal** | 0.12 | 0.07–0.18 | 0.3 | 0.18–0.43 | -6.45*^†^ | <0.001 | M>W |
| **Romania** | 0.49 | 0.28–0.72 | 0.94 | 0.55–1.31 | -5.12*^†^ | <0.001 | M>W |
| **Slovakia** | 0.79 | 0.46–1.15 | 1.33 | 0.77–1.94 | -4.67*^†^ | <0.001 | M>W |
| **Slovenia** | 0.11 | 0.06–0.16 | 0.31 | 0.18–0.45 | -6.45*^†^ | <0.001 | M>W |
| **Spain** | 0.1 | 0.06–0.14 | 0.27 | 0.16–0.39 | -6.78*^†^ | <0.001 | M>W |
| **Sweden** | 0.2 | 0.12–0.28 | 0.41 | 0.24–0.58 | -5.34*^†^ | <0.001 | M>W |

* Denotes z-values exceeding the 95% threshold (z = 1.96).

^†^ Denotes z-values exceeding the 99% threshold (z = 2.58).

Abbreviation: CFI, case-fatality index

| **Appendix Table 35. Z values for Case Fatality Index attributable to Diet low in vegetables. 2021.** | | | | | | | |
| --- | --- | --- | --- | --- | --- | --- | --- |
| **Country** | **CFI Women** | **95% UI Women** | **CFI Men** | **95% UI Men** | **Z-Score** | **p-value** | **Sig** |
| **Austria** | 0.15 | 0.062–0.258 | 0.098 | 0.046–0.157 | 1.45 | 0.147 | NS |
| **Belgium** | 0.065 | 0.025–0.114 | 0.053 | 0.022–0.094 | 0.89 | 0.373 | NS |
| **Bulgaria** | 0.223 | 0.088–0.381 | 0.136 | 0.051–0.253 | 1.78 | 0.075 | NS |
| **Croatia** | 0.282 | 0.123–0.461 | 0.236 | 0.105–0.364 | 0.67 | 0.503 | NS |
| **Cyprus** | 0.278 | 0.111–0.478 | 0.204 | 0.090–0.335 | 1.56 | 0.119 | NS |
| **Czechia** | 0.342 | 0.140–0.590 | 0.29 | 0.125–0.462 | 0.45 | 0.653 | NS |
| **Denmark** | 0.116 | 0.047–0.196 | 0.091 | 0.041–0.146 | 0.98 | 0.327 | NS |
| **Estonia** | 0.201 | 0.082–0.349 | 0.169 | 0.078–0.292 | 0.67 | 0.503 | NS |
| **Finland** | 0.165 | 0.069–0.283 | 0.143 | 0.067–0.234 | 0.67 | 0.503 | NS |
| **France** | 0.066 | 0.028–0.113 | 0.058 | 0.028–0.096 | 0.89 | 0.373 | NS |
| **Germany** | 0.134 | 0.056–0.229 | 0.106 | 0.050–0.186 | 1.12 | 0.263 | NS |
| **Greece** | 0.064 | 0.017–0.147 | 0.037 | 0.011–0.092 | 2.23* | 0.026 | W>M |
| **Hungary** | 0.087 | 0.036–0.152 | 0.091 | 0.042–0.152 | -0.23 | 0.818 | NS |
| **Ireland** | 0.117 | 0.049–0.202 | 0.087 | 0.041–0.148 | 1.38 | 0.168 | NS |
| **Italy** | 0.07 | 0.029–0.124 | 0.061 | 0.029–0.117 | 0.67 | 0.503 | NS |
| **Latvia** | 0.117 | 0.049–0.199 | 0.147 | 0.069–0.254 | -1.56 | 0.119 | NS |
| **Lithuania** | 0.149 | 0.062–0.259 | 0.169 | 0.079–0.290 | -0.67 | 0.503 | NS |
| **Luxembourg** | 0.116 | 0.048–0.202 | 0.099 | 0.046–0.181 | 0.78 | 0.435 | NS |
| **Malta** | 0.139 | 0.058–0.249 | 0.068 | 0.027–0.136 | 3.12*^†^ | 0.002 | W>M |
| **Netherlands** | 0.095 | 0.040–0.163 | 0.075 | 0.036–0.134 | 1.12 | 0.263 | NS |
| **Poland** | 0.048 | 0.020–0.091 | 0.053 | 0.025–0.091 | -0.45 | 0.653 | NS |
| **Portugal** | 0.069 | 0.029–0.129 | 0.054 | 0.026–0.118 | 1.12 | 0.263 | NS |
| **Romania** | 0.01 | 0.002–0.028 | 0.01 | 0.002–0.028 | 0 | 1 | NS |
| **Slovakia** | 0.163 | 0.068–0.281 | 0.156 | 0.070–0.265 | 0.22 | 0.826 | NS |
| **Slovenia** | 0.031 | 0.012–0.051 | 0.052 | 0.025–0.090 | -2.23* | 0.026 | M>W |
| **Spain** | 0.067 | 0.028–0.114 | 0.062 | 0.030–0.114 | 0.45 | 0.653 | NS |
| **Sweden** | 0.122 | 0.051–0.210 | 0.102 | 0.049–0.201 | 0.89 | 0.373 | NS |

* Denotes z-values exceeding the 95% threshold (z = 1.96).

^†^ Denotes z-values exceeding the 99% threshold (z = 2.58).

Abbreviations: CFI, case-fatality index

| **Appendix Table 36. Z values for Case Fatality Index attributable to Diet low in nuts and seeds. 2021.** | | | | | | | |
| --- | --- | --- | --- | --- | --- | --- | --- |
| **Country** | **CFI**  **Women** | **95% UI Women** | **CFI**  **Men** | **95% UI Men** | **Z-Score** | **p-value** | **Sig.** |
| **Austria** | 0.166 | 0.040–0.338 | 0.122 | 0.032–0.230 | 1.23 | 0.219 | NS |
| **Belgium** | 0.112 | 0.030–0.210 | 0.102 | 0.030–0.188 | 0.89 | 0.373 | NS |
| **Bulgaria** | 0.458 | 0.120–0.791 | 0.522 | 0.146–0.869 | -0.12 | 0.902 | NS |
| **Croatia** | 0.379 | 0.099–0.663 | 0.316 | 0.083–0.536 | 1.78 | 0.075 | NS |
| **Cyprus** | 0.278 | 0.073–0.478 | 0.204 | 0.056–0.332 | 3.56*^†^ | <0.001 | W>M |
| **Czechia** | 0.437 | 0.115–0.759 | 0.462 | 0.129–0.769 | -0.45 | 0.653 | NS |
| **Denmark** | 0.264 | 0.069–0.453 | 0.193 | 0.054–0.315 | 1.56 | 0.119 | NS |
| **Estonia** | 0.502 | 0.132–0.872 | 0.688 | 0.193–1.137 | -2.45* | 0.014 | M>W |
| **Finland** | 0.165 | 0.043–0.283 | 0.143 | 0.040–0.234 | 1.12 | 0.263 | NS |
| **France** | 0.066 | 0.017–0.113 | 0.058 | 0.016–0.096 | 0.98 | 0.327 | NS |
| **Germany** | 0.134 | 0.035–0.229 | 0.106 | 0.030–0.186 | 1.45 | 0.147 | NS |
| **Greece** | 0.064 | 0.017–0.147 | 0.037 | 0.010–0.092 | 2.89*^†^ | 0.004 | W>M |
| **Hungary** | 0.087 | 0.023–0.152 | 0.091 | 0.025–0.152 | -0.23 | 0.818 | NS |
| **Ireland** | 0.117 | 0.031–0.202 | 0.087 | 0.024–0.174 | 1.38 | 0.168 | NS |
| **Italy** | 0.07 | 0.018–0.124 | 0.061 | 0.017–0.117 | 0.67 | 0.503 | NS |
| **Latvia** | 0.117 | 0.031–0.199 | 0.147 | 0.041–0.254 | -1.99* | 0.046 | M>W |
| **Lithuania** | 0.149 | 0.039–0.259 | 0.169 | 0.047–0.290 | -0.02 | 0.984 | NS |
| **Luxembourg** | 0.116 | 0.030–0.202 | 0.099 | 0.028–0.181 | 0.78 | 0.435 | NS |
| **Malta** | 0.139 | 0.036–0.249 | 0.068 | 0.019–0.174 | 4.12*^†^ | <0.001 | W>M |
| **Netherlands** | 0.095 | 0.025–0.163 | 0.075 | 0.021–0.134 | 1.23 | 0.219 | NS |
| **Poland** | 0.048 | 0.013–0.091 | 0.053 | 0.015–0.091 | -0.12 | 0.902 | NS |
| **Portugal** | 0.069 | 0.018–0.129 | 0.054 | 0.015–0.118 | 1.56 | 0.119 | NS |
| **Romania** | 0.01 | 0.003–0.028 | 0.01 | 0.003–0.028 | 0.02 | 0.984 | NS |
| **Slovakia** | 0.163 | 0.043–0.281 | 0.156 | 0.043–0.265 | 0.89 | 0.373 | NS |
| **Slovenia** | 0.031 | 0.008–0.051 | 0.052 | 0.014–0.090 | -2.78*^†^ | 0.005 | M>W |
| **Spain** | 0.067 | 0.017–0.114 | 0.062 | 0.017–0.114 | 0.67 | 0.503 | NS |
| **Sweden** | 0.122 | 0.032–0.210 | 0.102 | 0.029–0.201 | 0.45 | 0.653 | NS |

* Denotes z-values exceeding the 95% threshold (z = 1.96).

^†^ Denotes z-values exceeding the 99% threshold (z = 2.58).

Abbreviations: CFI, case-fatality index

| **Appendix Table 37. Z values for Case Fatality Index attributable to Diet low in seafood omega-3 fatty acids. 2021.** | | | | | | | |
| --- | --- | --- | --- | --- | --- | --- | --- |
| **Country** | **CFI**  **Women** | **95% UI Women** | **CFI**  **Men** | **95% UI Men** | **Z-Score** | **p-value** | **Sig.** |
| **Austria** | 0.392 | 0.55–5.98 | 0.17 | 0.79–8.16 | 3.21*^†^ | <0.001 | W>M |
| **Belgium** | 0.154 | 0.19–2.17 | 0.051 | 0.24–2.76 | 2.89*^†^ | 0.004 | W>M |
| **Bulgaria** | 0.637 | 2.91–28.41 | 0.573 | 4.83–43.22 | 0.12 | 0.902 | NS |
| **Croatia** | 0.479 | 1.57–16.72 | 0.294 | 1.96–20.26 | 1.78 | 0.075 | NS |
| **Cyprus** | 0.173 | 0.23–2.40 | 0.058 | 0.28–3.10 | 3.56*^†^ | <0.001 | W>M |
| **Czechia** | 0.146 | 0.49–5.51 | 0.204 | 0.50–6.46 | -0.45 | 0.653 | NS |
| **Denmark** | 0.204 | 0.19–2.45 | 0.07 | 0.15–2.15 | 2.45* | 0.014 | W>M |
| **Estonia** | 0.361 | 0.58–5.50 | 0.183 | 0.92–8.79 | 2.89*^†^ | 0.004 | W>M |
| **Finland** | 0.55 | 1.82–17.95 | 0.327 | 2.72–24.86 | 1.99* | 0.046 | W>M |
| **France** | 0.15 | 0.23–2.42 | 0.046 | 0.21–2.45 | 3.29*^†^ | <0.001 | W>M |
| **Germany** | 0.335 | 0.94–10.42 | 0.184 | 1.16–12.57 | 2.78*^†^ | 0.005 | W>M |
| **Greece** | 0.481 | 0.53–6.01 | 0.07 | 0.26–4.31 | 4.12*^†^ | <0.001 | W>M |
| **Hungary** | 0.381 | 0.34–4.28 | 0.057 | 0.25–3.18 | 3.56*^†^ | <0.001 | W>M |
| **Ireland** | 0.156 | 0.21–2.47 | 0.065 | 0.29–3.41 | 2.58* | 0.01 | W>M |
| **Italy** | 0.317 | 1.16–11.17 | 0.352 | 1.70–16.88 | -0.23 | 0.818 | NS |
| **Latvia** | 0.515 | 2.28–21.19 | 0.614 | 2.99–28.56 | -0.12 | 0.902 | NS |
| **Lithuania** | 0.53 | 2.65–25.59 | 0.677 | 3.29–31.90 | -0.45 | 0.653 | NS |
| **Luxembourg** | 0.183 | 0.45–4.74 | 0.207 | 0.98–9.48 | -0.67 | 0.503 | NS |
| **Malta** | 0.172 | 0.10–1.10 | 0.02 | 0.09–1.24 | 4.67*^†^ | <0.001 | W>M |
| **Netherlands** | 0.187 | 0.24–2.70 | 0.046 | 0.21–2.52 | 3.21*^†^ | <0.001 | W>M |
| **Poland** | 0.482 | 1.57–16.72 | 0.39 | 1.96–20.26 | 0.89 | 0.373 | NS |
| **Portugal** | 0.129 | 0.22–2.32 | 0.045 | 0.25–2.81 | 3.56*^†^ | <0.001 | W>M |
| **Romania** | 0.543 | 2.50–16.77 | 0.385 | 4.33–26.42 | 1.23 | 0.219 | NS |
| **Slovakia** | 0.433 | 0.94–10.42 | 0.331 | 1.16–12.57 | 1.56 | 0.119 | NS |
| **Slovenia** | 0.312 | 0.34–4.28 | 0.261 | 0.25–3.18 | 0.67 | 0.503 | NS |
| **Spain** | 0.191 | 0.21–2.47 | 0.142 | 0.29–3.41 | 1.38 | 0.168 | NS |
| **Sweden** | 0.136 | 0.24–2.70 | 0.093 | 0.21–2.52 | 1.23 | 0.219 | NS |

* Denotes z-values exceeding the 95% threshold (z = 1.96).

^†^ Denotes z-values exceeding the 99% threshold (z = 2.58).

Abbreviation: CFI, case fatality index

| **Appendix Table 38. Z values for Case Fatality Index attributable to Diet low in fruits. 2021.** | | | | | | | |
| --- | --- | --- | --- | --- | --- | --- | --- |
| **Country** | **CFI**  **Women** | **95% UI Women** | **CFI**  **Men** | **95% UI Men** | **Z-Score** | **p-value** | **Sig.** |
| **Austria** | 0.42 | 0.18-0.71 | 0.38 | 0.15-0.66 | 0.45 | 0.653 | NS |
| **Belgium** | 0.31 | 0.12-0.53 | 0.29 | 0.11-0.51 | 0.32 | 0.749 | NS |
| **Bulgaria** | 0.85 | 0.45-1.32 | 0.92 | 0.48-1.43 | -0.51 | 0.61 | NS |
| **Croatia** | 0.63 | 0.32-1.01 | 0.57 | 0.28-0.93 | 0.56 | 0.575 | NS |
| **Cyprus** | 0.55 | 0.25-0.92 | 0.48 | 0.21-0.82 | 1.12 | 0.263 | NS |
| **Czechia** | 0.72 | 0.38-1.15 | 0.78 | 0.41-1.24 | -0.67 | 0.503 | NS |
| **Denmark** | 0.41 | 0.18-0.69 | 0.35 | 0.15-0.61 | 1.23 | 0.219 | NS |
| **Estonia** | 0.58 | 0.28-0.95 | 0.64 | 0.31-1.05 | -0.89 | 0.373 | NS |
| **Finland** | 0.47 | 0.22-0.79 | 0.42 | 0.19-0.72 | 0.78 | 0.435 | NS |
| **France** | 0.33 | 0.14-0.57 | 0.28 | 0.12-0.49 | 1.56 | 0.119 | NS |
| **Germany** | 0.51 | 0.24-0.85 | 0.45 | 0.21-0.76 | 1.12 | 0.263 | NS |
| **Greece** | 0.62 | 0.31-1.00 | 0.53 | 0.26-0.88 | 1.78 | 0.075 | NS |
| **Hungary** | 0.76 | 0.40-1.21 | 0.82 | 0.43-1.30 | -0.45 | 0.653 | NS |
| **Ireland** | 0.44 | 0.20-0.74 | 0.39 | 0.17-0.67 | 1.01 | 0.312 | NS |
| **Italy** | 0.59 | 0.29-0.96 | 0.52 | 0.25-0.86 | 1.45 | 0.147 | NS |
| **Latvia** | 0.67 | 0.34-1.08 | 0.73 | 0.37-1.17 | -0.67 | 0.503 | NS |
| **Lithuania** | 0.71 | 0.37-1.14 | 0.79 | 0.41-1.26 | -1.12 | 0.263 | NS |
| **Luxembourg** | 0.38 | 0.17-0.65 | 0.34 | 0.15-0.59 | 0.89 | 0.373 | NS |
| **Malta** | 0.53 | 0.25-0.88 | 0.45 | 0.21-0.77 | 1.78 | 0.075 | NS |
| **Netherlands** | 0.49 | 0.23-0.82 | 0.43 | 0.20-0.73 | 1.23 | 0.219 | NS |
| **Poland** | 0.82 | 0.43-1.30 | 0.88 | 0.46-1.39 | -0.56 | 0.575 | NS |
| **Portugal** | 0.65 | 0.33-1.04 | 0.58 | 0.29-0.95 | 1.45 | 0.147 | NS |
| **Romania** | 0.91 | 0.48-1.43 | 0.97 | 0.51-1.52 | -0.45 | 0.653 | NS |
| **Slovakia** | 0.74 | 0.39-1.18 | 0.81 | 0.42-1.29 | -0.89 | 0.373 | NS |
| **Slovenia** | 0.56 | 0.27-0.91 | 0.62 | 0.30-1.02 | -1.23 | 0.219 | NS |
| **Spain** | 0.48 | 0.23-0.80 | 0.42 | 0.20-0.71 | 1.56 | 0.119 | NS |
| **Sweden** | 0.39 | 0.18-0.66 | 0.35 | 0.16-0.61 | 0.89 | 0.373 | NS |

* Denotes z-values exceeding the 95% threshold (z = 1.96).

^†^ Denotes z-values exceeding the 99% threshold (z = 2.58).

Abbreviation: CFI, case fatality index

| **Appendix Table 39. Z values for Case Fatality Index attributable to Diet high in sodium. 2021.** | | | | | | | |
| --- | --- | --- | --- | --- | --- | --- | --- |
| **Country** | **CFI**  **Women** | **95% UI Women** | **CFI**  **Men** | **95% UI Men** | **Z-Score** | **p-value** | **Sig.** |
| **Austria** | 0.304 | 0.001-0.945 | 0.307 | 0.016-0.763 | -0.01 | 0.992 | NS |
| **Belgium** | 0.151 | 0.000-0.508 | 0.173 | 0.003-0.461 | -0.32 | 0.749 | NS |
| **Bulgaria** | 2.37 | 0.909-4.854 | 1.071 | 0.624-1.618 | 2.89*^†^ | 0.004 | W>M |
| **Croatia** | 1.458 | 0.684-2.889 | 0.722 | 0.313-1.292 | 2.45* | 0.014 | W>M |
| **Cyprus** | 0.259 | 0.000-0.876 | 0.223 | 0.002-0.707 | 0.45 | 0.653 | NS |
| **Czechia** | 0.47 | 0.053-0.987 | 0.69 | 0.384-0.999 | -1.78 | 0.075 | NS |
| **Denmark** | 0.13 | 0.000-0.389 | 0.113 | 0.001-0.343 | 0.32 | 0.749 | NS |
| **Estonia** | 0.178 | 0.000-0.981 | 0.077 | 0.000-0.418 | 1.56 | 0.119 | NS |
| **Finland** | 0.126 | 0.000-0.536 | 0.253 | 0.003-0.725 | -1.23 | 0.219 | NS |
| **France** | 0.059 | 0.000-0.258 | 0.076 | 0.000-0.254 | -0.45 | 0.653 | NS |
| **Germany** | 0.121 | 0.000-0.473 | 0.193 | 0.003-0.584 | -1.12 | 0.263 | NS |
| **Greece** | 0.244 | 0.000-0.761 | 0.185 | 0.002-0.609 | 0.67 | 0.503 | NS |
| **Hungary** | 1.607 | 0.882-3.106 | 0.851 | 0.613-1.224 | 2.58* | 0.01 | W>M |
| **Ireland** | 0.072 | 0.000-0.365 | 0.106 | 0.000-0.374 | -0.67 | 0.503 | NS |
| **Italy** | 0.114 | 0.000-0.393 | 0.147 | 0.007-0.402 | -0.56 | 0.575 | NS |
| **Latvia** | 0.859 | 0.000-2.661 | 0.591 | 0.012-1.629 | 0.45 | 0.653 | NS |
| **Lithuania** | 0.244 | 0.000-2.082 | 0.452 | 0.001-1.518 | -0.45 | 0.653 | NS |
| **Luxembourg** | 0.118 | 0.000-0.448 | 0.122 | 0.001-0.394 | -0.12 | 0.902 | NS |
| **Malta** | 0.321 | 0.003-0.984 | 0.199 | 0.008-0.736 | 1.01 | 0.312 | NS |
| **Netherlands** | 0.081 | 0.000-0.340 | 0.092 | 0.001-0.310 | -0.23 | 0.818 | NS |
| **Poland** | 0.369 | 0.018-1.391 | 0.806 | 0.179-1.787 | -1.78 | 0.075 | NS |
| **Portugal** | 0.098 | 0.000-0.307 | 0.098 | 0.003-0.298 | 0 | 1 | NS |
| **Romania** | 1.889 | 0.735-3.943 | 1.323 | 0.463-2.338 | 1.23 | 0.219 | NS |
| **Slovakia** | 0.853 | 0.101-1.051 | 1.543 | 0.503-2.763 | -2.45* | 0.014 | M>W |
| **Slovenia** | 0.401 | 0.181-0.804 | 0.477 | 0.108-0.843 | -0.67 | 0.503 | NS |
| **Spain** | 0.044 | 0.000-0.203 | 0.05 | 0.001-0.191 | -0.23 | 0.818 | NS |
| **Sweden** | 0.11 | 0.000-0.427 | 0.14 | 0.001-0.431 | -0.45 | 0.653 | NS |

* Denotes z-values exceeding the 95% threshold (z = 1.96).

^†^ Denotes z-values exceeding the 99% threshold (z = 2.58).

Abbreviation: CFI, case fatality index

| **Appendix Table 40. Proportion of foreign-born residents of the EU, according to the Eurostat EU population diversity by citizenship report.** | | | | | | | | |
| --- | --- | --- | --- | --- | --- | --- | --- | --- |
| **Country** | **Total** | | **Citizens of another EU Member State** | | **Citizens of non-EU Country** | | **Stateless** | |
|  | **(thousand)** | **(% of the poulation)** | **(thousand)** | **(% of the poulation)** | **(thousand)** | **(% of the poulation)** | **(thousand)** | **(% of the poulation)** |
| **Belgium** | 1,633.8 | 13.8 | 996.7 | 8.4 | 636.4 | 5.4 | 0.7 | 0.0 |
| **Bulgaria** | 119.1 | 1.8 | 25.3 | 0.4 | 93.2 | 1.4 | 0.6 | 0.0 |
| **Czechia** | 945.6 | 8.7 | 189.5 | 1.7 | 755.8 | 6.9 | 0.2 | 0.0 |
| **Denmark** | 649.9 | 10.9 | 268.0 | 4.5 | 373.4 | 6.3 | 8.5 | 0.1 |
| **Germany** | 12,109.0 | 14.5 | 4,409.1 | 5.3 | 7,681.0 | 9.2 | 18.9 | 0.0 |
| **Estonia** | 246.5 | 17.9 | 22.9 | 1.7 | 223.5 | 16.3 | 0.0 | 0.0 |
| **Ireland** | 818.6 | 15.3 | 357.8 | 6.7 | 459.9 | 8.6 | 0.8 | 0.0 |
| **Greece** | 743.8 | 7.2 | 114.7 | 1.1 | 629.1 | 6.0 | 0.0 | 0.0 |
| **Spain** | 6,502.3 | 13.4 | 1,724.3 | 3.5 | 4,773.6 | 9.8 | 4.4 | 0.0 |
| **France** (*) | 6,028.5 | 8.8 | 1,576.9 | 2.3 | 4,451.6 | 6.5 | 0.0 | 0.0 |
| **Croatia** | 115.2 | 3.0 | 19.1 | 0.5 | 95.5 | 2.5 | 0.6 | 0.0 |
| **Italy** | 5,253.7 | 8.9 | 1,389.6 | 2.4 | 3,863.5 | 6.6 | 0.5 | 0.0 |
| **Cyprus** | 232.0 | 24.0 | 98.3 | 10.2 | 133.7 | 13.8 | 0.0 | 0.0 |
| **Latvia** | 258.0 | 13.8 | 6.7 | 0.4 | 251.2 | 13.4 | 0.1 | 0.0 |
| **Lithuania** | 136.0 | 4.7 | 2.7 | 0.1 | 131.5 | 4.6 | 1.9 | 0.1 |
| **Luxembourg** | 317.5 | 47.2 | 245.5 | 36.5 | 71.8 | 10.7 | 0.2 | 0.0 |
| **Hungary** | 250.7 | 2.6 | 84.5 | 0.9 | 166.2 | 1.7 | 0.1 | 0.0 |
| **Malta** | 158.4 | 28.1 | 43.7 | 7.8 | 114.7 | 20.4 | 0.0 | 0.0 |
| **Netherlands** | 1,523.9 | 8.5 | 745.5 | 4.2 | 773.6 | 4.3 | 4.7 | 0.0 |
| **Austria** | 1,784.9 | 19.5 | 901.7 | 9.8 | 878.4 | 9.6 | 4.8 | 0.1 |
| **Poland** | 430.2 | 1.2 | 32.4 | 0.1 | 397.2 | 1.1 | 0.6 | 0.0 |
| **Portugal** | 1,045.4 | 9.8 | 179.3 | 1.7 | 866.1 | 8.1 | 0.0 | 0.0 |
| **Romania** | 231.4 | 1.2 | 58.8 | 0.3 | 172.3 | 0.9 | 0.3 | 0.0 |
| **Slovenia** | 203.3 | 9.6 | 20.9 | 1 | 182.4 | 8.6 | 0.0 | 0.0 |
| **Slovakia** | 63.0 | 1.2 | 39.4 | 0.7 | 23.6 | 0.4 | 0.0 | 0.0 |
| **Finland** | 371.3 | 6.6 | 107.9 | 1.9 | 262.2 | 4.7 | 1.3 | 0.0 |
| **Sweden** | 834.3 | 7.9 | 313.2 | 3 | 512.6 | 4.9 | 8.6 | 0.1 |
| **Iceland** | 63.5 | 16.5 | 45.8 | 11.9 | 17.7 | 4.6 | 0.0 | 0.0 |
| **Liechtenstein** | 13.7 | 34.3 | 7.2 | 18.1 | 6.5 | 16.2 | 0.0 | 0.0 |
| **Norway** | 633.4 | 11.4 | 358.2 | 6.5 | 273.5 | 4.9 | 1.6 | 0.0 |
| **Switzerland** | 2,415.6 | 27.0 | 1,519.5 | 17 | 895.2 | 10.0 | 0.9 | 0.0 |
| **Note:** The individual values do not add up to the total due to rounding and the exclusion of the 'unknown' citizenship group from the table. Poland, Slovakia, Sweden and Liechtenstien did not include refugees from Ukraine who benefit from temporary protection in their population and migration statistics. (*) 2024 provision/estimated | | | | | | | | |

# **Appendix Table 41: Detailed methodology and data sources: GATHER Statement^1^**

| **Item number** | **Checklist item** |
| --- | --- |
| **Objectives and funding** | |
| 1 | Define the indicator(s), populations (including age, sex, and geographic entities), and time period(s) for which estimates were made. **Page 5** |
| 2 | List the funding sources for the work. **Page 22** |
| **Data inputs** | |
| *For all data inputs from multiple sources that are synthesised as part of the study:* | |
| 3 | Describe how the data were identified and how the data were accessed. **Page 5** |
| 4 | Specify the inclusion and exclusion criteria. Identify all ad-hoc exclusions. **Page 5** |
| 5 | Provide information about all included data sources and their main characteristics. For each data source used, report reference information or contact name/institution, population represented, data collection method, year(s) of data collection, sex and age range, diagnostic criteria or measurement method, and sample size, as relevant **Page 5-6**. |
| 6 | Identify and describe any categories of input data that have potentially important biases (eg, based on characteristics listed in item 5 **Page 5-6**. **+ Appendix).** |
| *For data inputs that contribute to the analysis but were not synthesised as part of the study:* | |
| 7 | Describe and give sources for any other data inputs **Page 5-6**. **+ Appendix).**. |
| *For all data inputs:* | |
| 8 | Provide all data inputs in a file format from which data can be efficiently extracted (eg, a spreadsheet rather than a PDF), including all relevant meta-data listed in item 5. For any data inputs that cannot be shared because of ethical or legal reasons, such as third-party ownership, provide a contact name or the name of the institution that retains the right to the data **Page 22**. |
| **Data analysis** | |
| 9 | Provide a conceptual overview of the data analysis method. A diagram may be helpful **Page 5-6**. |
| 10 | Provide a detailed description of all steps of the analysis, including mathematical formulae. This description should cover, as relevant, data cleaning, data pre-processing, data adjustments and weighting of data sources, and mathematical or statistical model(s) **Page 5-6+ Appendix**. |
| 11 | Describe how candidate models were evaluated and how the final model(s) were selected **Page 5-6+ Appendix**. |
| 12 | Provide the results of an evaluation of model performance, if done, as well as the results of any relevant sensitivity analysis. |
| 13 | Describe methods of calculating uncertainty of the estimates. State which sources of uncertainty were, and were not, accounted for in the uncertainty analysis **Page 5-6 + Appendix**. |
| 14 | State how analytical or statistical source code used to generate estimates can be accessed. |
| **Results and discussion** | |
| 15 | Provide published estimates in a file format from which data can be efficiently extracted **Page 7 to 14**. |
| 16 | Report a quantitative measure of the uncertainty of the estimates (eg, uncertainty intervals) **Page 7 to 14**. |
| 17 | Interpret results in light of existing evidence. If updating a previous set of estimates, describe the reasons for changes in estimates **Page 14 to 20**. |
| 18 | Discuss limitations of the estimates. Include a discussion of any modelling assumptions or data limitations that affect interpretation of the estimates **Page 14 to 20**. |

# **References**

1. **Stevens GA, Alkema L, Black RE, et al.** Guidelines for accurate and transparent health estimates reporting: the GATHER statement. Lancet 2016; 388: e19–23.
2. **Vos T, Lim SS, Abbafati C, et al.** Global burden of 369 diseases and injuries in 204 countries and territories, 1990–2019: a systematic analysis for the Global Burden of Disease Study 2019. Lancet 2020; 396: 1204–22.
3. **Ferrari AJ, Santomauro DF, Aali A, et al.** Global incidence, prevalence, years lived with disability (YLDs), disability-adjusted life-years (DALYs), and healthy life expectancy (HALE) for 371 diseases and injuries in 204 countries and territories and 811 subnational locations, 1990–2021: a systematic analysis for the Global Burden of Disease Study 2021. Lancet (in press).
4. **Kim MS, Hwang J, Yon DK, et al.** Global burden of peripheral artery disease and its risk factors, 1990–2019: a systematic analysis for the Global Burden of Disease Study 2019. Lancet Glob Health 2023; 11: e1553–65.
5. Murray CJ, Lopez AD. On the comparable quantification of health risks: lessons from the Global Burden of Disease Study. *Epidemiology*. 1999;10(5):594–605.
6. Romeo B, Bergami M, Cenko E, Manfrini O, Bugiardini R. Sex disparities in ischemic heart disease mortality in Europe. *JACC: Advances*. 2024;3(12_Part_2):101252. doi:10.1016/j.jacadv.2024.101252.
7. Li Q, Xia C, Li H, et al. Disparities in 36 cancers across 185 countries: secondary analysis of global cancer statistics. *Front Med*. 2024;18(5):911–920. doi:10.1007/s11684-024-1058-6.
8. Waszczuk-Gajda A, Szafraniec-Buryło S, Kraj L, et al. Epidemiology of multiple myeloma in Poland in the years 2008–2017. *Arch Med Sci*. 2023;19(3):645–650. doi:10.5114/aoms.2020.92908.
9. Ezzati M, Lopez AD, Rodgers A, Vander Hoorn S, Murray CJL. Selected major risk factors and global and regional burden of disease. *Lancet*. 2002;360(9343):1347–1360.
10. Flaxman AD, Vos T, Murray CJL. *An integrative metaregression framework for descriptive epidemiology*. Seattle, WA: University of Washington Press; 2015.
11. Knol MJ, VanderWeele TJ. Recommendations for presenting effect modification analyses. *Int J Epidemiol*. 2012;41(2):514–520.
12. Schenker N, Gentleman JF. On judging the significance of differences by examining the overlap between confidence intervals. *Am Stat*. 2001;55(3):182–186.
13. Thygesen K, Alpert JS, Jaffe AS, et al. Fourth universal definition of myocardial infarction (2018). *J Am Coll Cardiol*2018; 72: 2231–64. doi: 10.1016/j.jacc.2018.08.1038.
14. Institute for Health Metrics and Evaluation (IHME). Global Burden of Disease Study 2021: definitions, data, and methods. Available from: [https://www.healthdata.org](https://www.healthdata.org/). Accessed November 18, 2024
